# Sequential Catalytic Functionalization of Aryltriazenyl Aldehydes for the Synthesis of Complex Benzenes

**DOI:** 10.1021/acscatal.1c01722

**Published:** 2021-05-05

**Authors:** Sangwon Seo, Ming Gao, Eva Paffenholz, Michael C. Willis

**Affiliations:** Department of Chemistry, Chemistry Research Laboratory, University of Oxford, Mansfield Road, Oxford, OX1 3TA, United Kingdom

**Keywords:** hydroacylation, rhodium, triazene, benzene, sequential catalysis

## Abstract

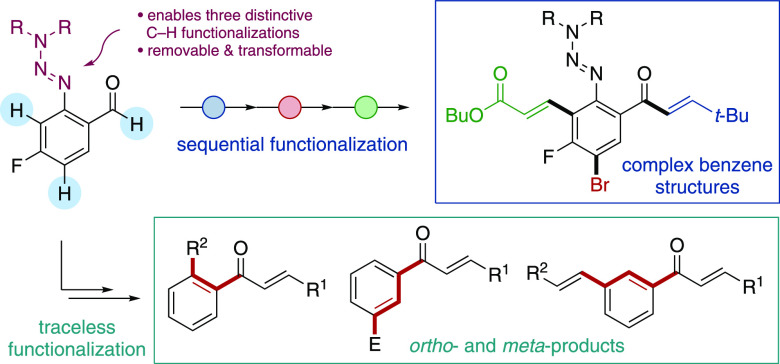

We demonstrate that aryltriazenes can promote three distinctive types of C–H functionalization reactions, allowing the preparation of complex benzene molecules with diverse substitution patterns. 2-Triazenylbenzaldehydes are shown to be efficient substrates for Rh(I)-catalyzed intermolecular alkyne hydroacylation reactions. The resulting triazene-substituted ketone products can then undergo either a Rh(III)-catalyzed C–H activation, or an electrophilic aromatic substitution reaction, achieving multifunctionalization of the benzene core. Subsequent triazene derivatization provides traceless products.

Given the abundance of C–H bonds in organic molecules, the functionalization of these bonds represents an ideal method for chemical manipulation.^1^ Transition-metal catalysis has played a significant role in the advancement of this field, providing powerful methods that are comparable to conventional metal-catalyzed cross-coupling reactions.^2^ In particular, the use of directing group strategies has been the dominant approach to achieve regioselective reactions.^3^ A limitation of such strategies is that the coordinating group, which, by design, is present to direct the metal catalyst to specific C–H bonds of the starting material, will also be present in the final product. This limits synthetic flexibility, and, thus, the ability to remove or transform the directing group to other useful functionalities is advantageous. In addition, it would be beneficial if the coordinating group was able to promote, not only one, but multiple C–H functionalization reactions in a selective way.^4,5^

Metal-catalyzed hydroacylation reactions are examples of C–H functionalizations in which the C–C multiple bond of an alkene or alkyne inserts into the formyl C–H bond of an aldehyde.^6,7^ Despite the advent of several non-chelation-controlled methods for hydroacylation reactions,^8^ intermolecular versions of these processes based on the use of some form of substrate chelation remain the most common.^9^ Aldehydes featuring P-,^10^ O-,^11^ N-,^12^ and S-based chelating groups,^13^ as well as chelating alkenes,^14^ have all been used, and reactions that proceed under mild reaction conditions and encompass broad substrate scopes have been achieved. Regio-^9e,9f,12,15^^f^ and enantioselective^16^ reactions have also been reported,^15b,17^ and applications have been developed.^11b,18^ With these advances in place, strategies to mitigate the issues associated with the presence of chelating-substituents are needed. In this context, approaches have been developed where the chelating group is either incorporated into a target structure,^18f,19^ or transformed to an alternate functionality.^18g,20^ For example, our laboratory has shown that a chelating methyl sulfide employed in hydroacylation reactions can be directly utilized in subsequent Rh-catalyzed carbothiolation, arylation, or reduction reactions (see Scheme 1a).^21^

**Scheme 1:**
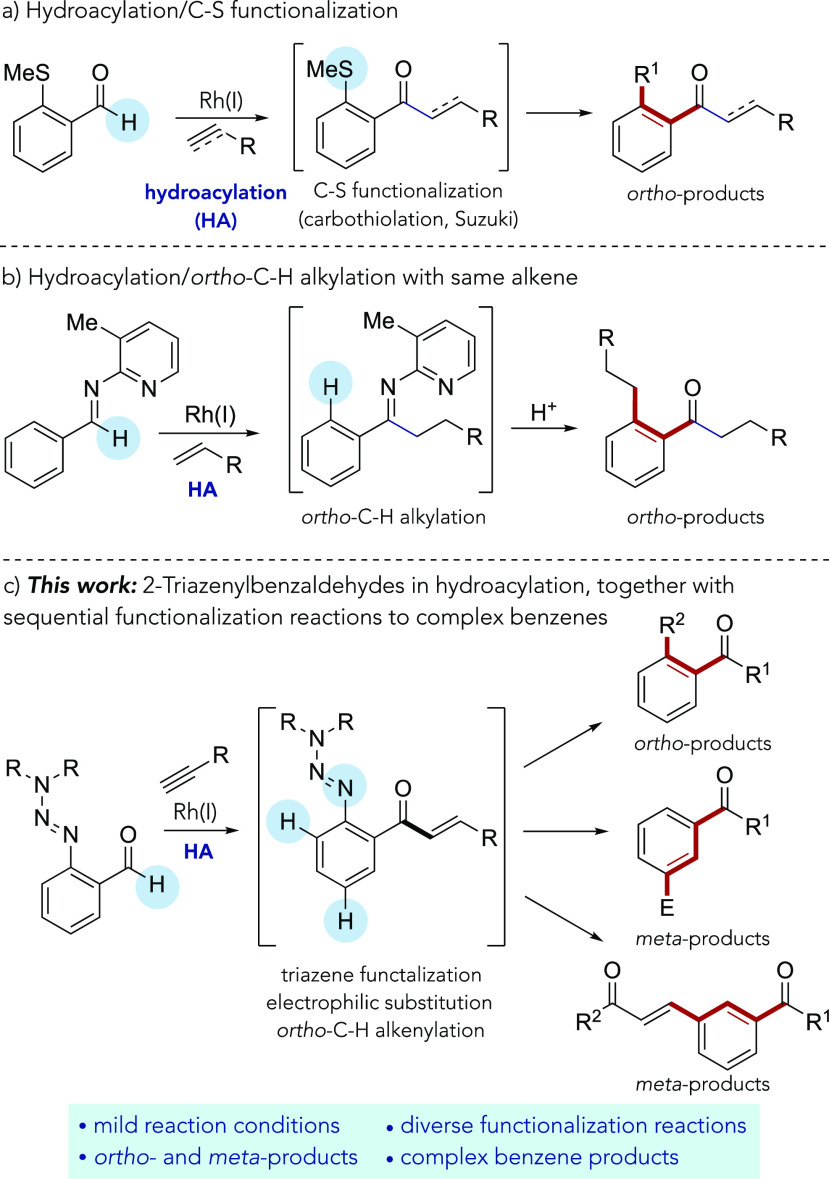
(a) Hydroacylation and Subsequent C–S Functionalization,^21^ (b) Cascade Hydroacylation/*ortho*–C-H Alkylation,^22^ (c) 2-Triazenyl-benzaldehydes in Hydroacylation and Sequential C–H, Triazene Functionalization, and E^+^ Substitution

Building on these prior reports, we aimed to develop an alternative chelating group that would provide complementary transformations and functionalization opportunities. We were particularly interested in an approach in where the coordinating group would be capable of promoting further C–H functionalization reactions, allowing the use of simple substrates, and access to a variety of substitution patterns. In this context, Jun has reported a cascade strategy that uses in-situ-generated picolyl imines for alkene hydroacylation and *ortho*-alkylation of benzaldehydes (Scheme 1b).^22^ This double C–H functionalization is assisted by a single chelating group. However, the harsh reaction conditions (170 °C reaction temperature) result in little regiocontrol, which, in turn, limits the functionalization at both C–H sites to the same coupling partner. The synthetic utility of this approach would be significantly improved if the distinct C–H bonds could be selectively functionalized using *different* coupling partners.^4^ To achieve these aims, we selected aryl aldehydes substituted with 2-triazenyl groups (Scheme 1c).^23^ The triazene group offers many potential advantages: (1) although not previously reported, the triazene group should be capable of acting as a chelating group for metal-catalyzed intermolecular hydroacylation, with the first nitrogen atom positioned to form a stable five-membered acyl-metal-hydride complex;^7d^ (2) catalyst coordination to the second nitrogen atom would direct the metal center to the *ortho*–C–H bond;^24^ (3) the electron-donating properties of the triazene would promote electrophilic aromatic substitution reactions; (4) triazene groups can be easily removed;^25^ and (5) triazenes can be transformed to a wide range of alternative functional groups.^23,24,26^ By exploiting just a selection of these activation modes, it should be possible to access multisubstituted benzenes; these are motifs that remain of considerable worth to medicinal chemists.^27^ Despite the versatility of the triazene group, its use as a directing group in metal-catalyzed C–H functionalization is rare and remains challenging.^24^ This is mainly due to the difficulty of controlling monofunctionalization vs difunctionalization,^24a,28^ which, in turn, limits synthetic applications. However, we were confident that our reaction design, in which a variety of chemically distinct C–H bonds are present, would alleviate these issues. Herein, we show that it is indeed possible to use triazene groups in Rh-catalyzed chelation-controlled alkyne hydroacylation, and in a variety of further functionalization processes, allowing access to complex benzene products.

2-Triazenylbenzaldehyde starting materials were prepared from widely available anthranilic acids using simple procedures.^29^ With the substrates in hand, we began our investigation by evaluating a range of known hydroacylation catalysts. It quickly became apparent that the combination of [Rh(nbd)_2_]BF_4_ (nbd = norbornadiene) and *bis*(diphenylphosphinoethane) (dppe), in dichloromethane solvent at room temperature, was the most efficient catalyst system for the coupling reaction between the piperidine derivative **1a** and a selection of terminal alkynes (see the Supporting Information for further details, as well as Scheme 2a). Excellent conversions and yields were achieved with 1-octyne (**2a**), *t*-Bu-substituted alkyne (**2b**), and phenylacetylene (**2c**), exclusively delivering the linear isomers of the hydroacylation adducts **3a**–**3c**.

**Scheme 2:**
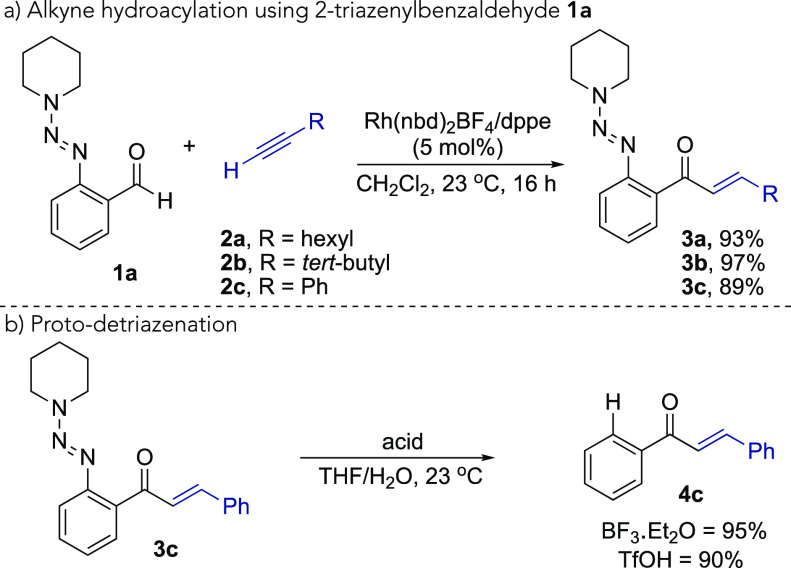
(a) Intermolecular Hydroacylation of 2-Triazenylbenzaldehyde **1a** with Terminal Alkynes, and (b) Removal of the Triazene Group under Acid Conditions

We next explored how readily the triazene group could be removed and replaced with a H-atom (see Scheme 2b). Initial attempts using either known reducing (H-SiCl_3_)^30^ or acidic conditions (TFA)^25^ were not successful. However, we found that by using either BF_3_·OEt_2_ or triflic acid, the triazene group could be efficiently removed (Scheme 2b).^31^ The use of a THF/water solvent mixture was important for the success of these reactions, because it presumably aids solubility of the diazonium salt intermediate.

Next, we examined the scope of sequential hydroacylation/triazene removal, with respect to different alkynes and 2-triazenylbenzaldehydes (Scheme 3). The reaction was generally effective, affording good to excellent yields of the traceless hydroacylation products. Note that both transformations were performed at ambient temperature. Aldehyde **1a** could be combined with a range of terminal alkynes, including those used in Scheme 2 (**4a**–**4c**), as well as cycloalkyl-substituted alkynes (**4d**, **4e**), enyne (**4f**), remote-aryl alkyne (**4g**), and ferrocenyl (**4h**) substrates. The reactions also proceeded well with a variety of different functional groups positioned around the arene core of the aldehydes; 4-chloro (**4i**), 4-trifluoromethyl (**4j**), 5-trifluoromethoxy (**4k**), and 5-fluoro (**4l**) substituents were all well-tolerated.

**Scheme 3:**
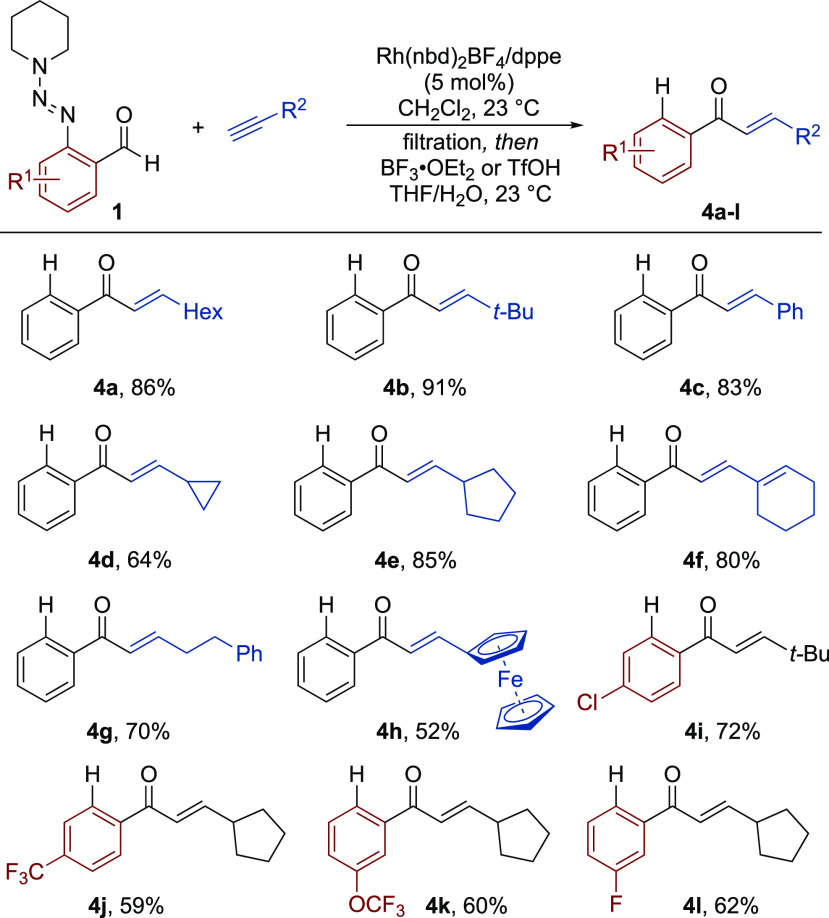
Traceless Hydroacylation via Sequential Alkyne Hydroacylation of 2-Triazenylbenzaldehydes/Removal of the Triazene Group Reaction conditions: **1** (1 equiv), alkyne (1.5 equiv), [Rh(nbd)_2_]BF_4_ (5 mol %), dppe (5 mol %), CH_2_Cl_2_, 23 °C, 16 h; then, silica filtration and BF_3_·OEt_2_ or TfOH (3.3–10 equiv), THF/H_2_O, 23 °C, 1 h. Isolated yields over two steps.

The ability of the coordinating triazene group to facilitate sequential C–H functionalization reactions was evaluated next. Using the conditions developed by Huang for the *ortho*–C–H olefination of aryltriazenes as a starting point,^24a^ we found that a Rh(III)-catalyst system could promote the C–H activation of the initial hydroacylation products. Further optimization showed that the original reaction conditions could be simplified, allowing the reaction to proceed efficiently in the absence of silver co-catalysts and at lower temperatures. With the modified conditions in place, we performed the three-component transformations in a sequential manner, with a simple filtration through a silica pad separating the two steps (see Scheme 4). Using two distinctive catalysts, the combination of 2-triazenylbenzaldehyde **1a** and *t*-Bu-substituted alkyne **2b**, followed by the *ortho*-olefination with butyl acrylate, gave the double C–H functionalization product **5a** in an excellent yield with absolute regiocontrol. A range of other terminal alkynes could also be employed successfully, including 1-octyne (**5b**), and those substituted with alkyl chloride (**5c**), phenyl (**5d**) and 3-thienyl (**5e**) groups. In addition, variation of the aldehyde component was possible; 6-fluoro (**5f**), 5-methyl (**5g**), 5-fluoro (**5h**), 4-chloro (**5i**), and 2-naphthyl (**5j**) substrates all delivered the final products in good to excellent yields. Importantly, the reaction could be performed on increased scale, with the isolation of 1.2 g of benzene **5f** showcasing the excellent practicability of the developed method.

**Scheme 4:**
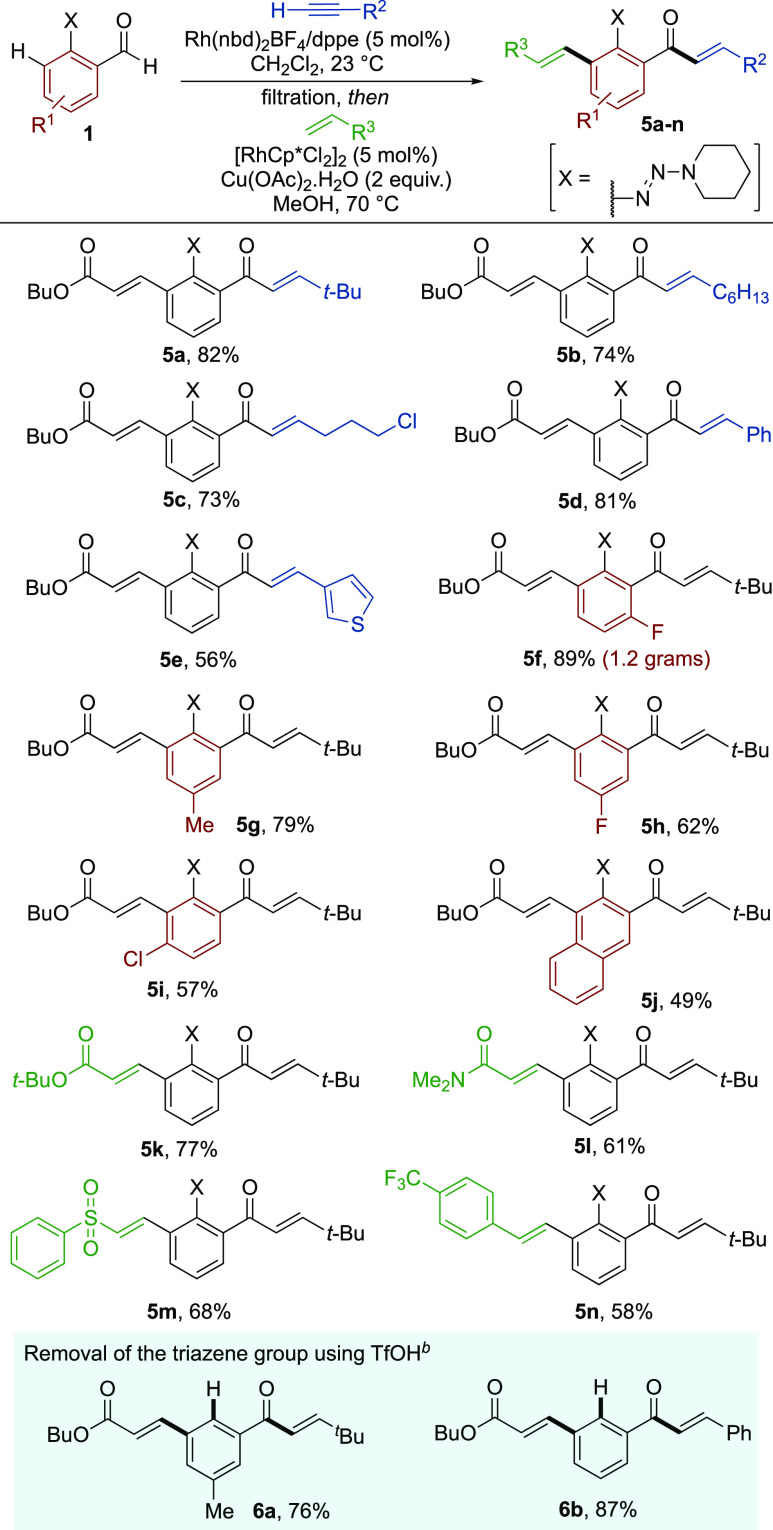
Sequential Hydroacylation/*ortho*–C–H Functionalization Reactions Reaction conditions: **1** (1 equiv), alkyne (1.5 equiv), [Rh(nbd)_2_]BF_4_ (5 mol %), dppe (5 mol %), CH_2_Cl_2_, 23 °C, 16 h; then, silica filtration and alkene (2.5 equiv), [RhCp*Cl_2_]_2_ (5 mol %), Cu(OAc)_2_·H_2_O (2 equiv), MeOH, 70 °C, 16 h. Isolated yields over two steps. *^b^*TfOH (3.3 equiv), THF/H_2_O, 23 °C, 1 h; Isolated yields.

The scope, with respect to the alkene component, was also examined. In addition to *tert*-butyl acrylate (**5k**), a selection of alkenes absent from Huang’s report was also compatible with the sequential process. These compounds included acrylamide (**5l**), phenylsulfone (**5m**), and styrene (**5n**), which afforded the corresponding products in good yields. As previously noted, the triazene group could subsequently be removed using triflic acid, providing the *meta*-substituted products (**6a** and **6b**).

Having explored the utility of the triazene unit as a directing group in metal-catalyzed sequential C–H functionalization reactions, we next turned our attention to its potential use as a controlling substituent in electrophilic aromatic substitution reactions. We envisioned that the electron-donating ability of the triazene should allow simple installation of electrophiles onto the benzene core, which would, when combined with hydroacylation and triazene removal, give access to additional substitution patterns. Attracted by the versatility of aryl bromides in organic synthesis, we selected bromination as the transformation of choice. We found that one-pot addition of NBS to the hydroacylation reaction mixture with stirring for 1 h at room temperature resulted in *para*-selective bromination, relative to the triazene substituent. In situ removal of the triazene could be achieved as observed previously, to afford the *meta*-substituted bromo enone products **7** (see Scheme 5).

**Scheme 5:**
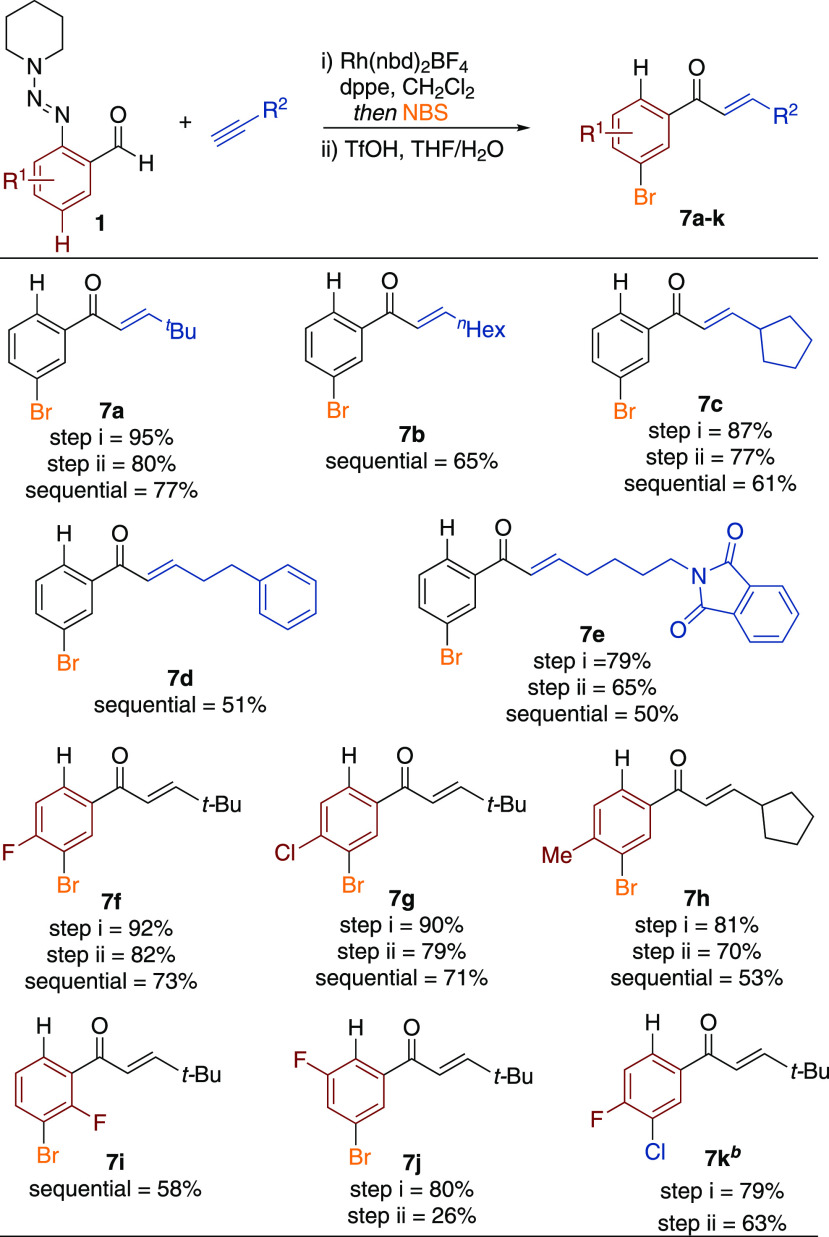
Meta-selective One-Pot Hydroacylation/Bromination, Followed by Removal of the Triazene Group Reaction conditions: (i) **1** (1 equiv), alkynes (1.5 equiv), [Rh(nbd)_2_]BF_4_ (5 mol %), dppe (5 mol %), CH_2_Cl_2_, 23 °C, 16 h; then NBS (1.5 equiv), 23 or 40 °C, 1–1.5 h, (ii) TfOH (3.3 equiv), THF/H_2_O, 23 °C, 1 h; Sequential yields = yields over two steps, obtained using one silica purification. *^b^*1-chloro-1,2-benziodoxol-3-one used in place of NBS.

Bromination using an isolated hydroacylation product confirmed that the process was not metal-catalyzed. In addition, a control reaction established that a simple (*E*)-chalcone was unreactive under these reaction conditions, confirming the requirement for the triazene group. The scope of the one-pot hydroacylation/bromination was general, and a range of alkynes and aldehydes could be employed successfully. *tert*-Butyl (**7a**), 1-octyne (**7b**), and cyclopentyl (**7c**) substrates were efficiently transformed to the corresponding sequential products. Phenyl- (**7d**) and phthalimide-substituted (**7e**) alkyl examples were also compatible. Bromination of the nontriazene-substituted aromatic rings was not observed in these substrates, establishing the high regioselectivity of this reaction. 2-Triazenylbenzaldehydes substituted with 4-fluoro (**7f**), 4-chloro (**7g**), 4-methyl (**7h**), or 6-fluoro (**7i**) were also suitable substrates, affording the *meta*-bromo products in good yields. The 3-fluoro (**7j**) substrate was also well-tolerated for one-pot hydroacylation/bromination, but triazene removal was inefficient. Chlorination could also be achieved if NBS was replaced with 1-chloro-1,2-benziodoxol-3-one,^32^ with *meta*-chloro-variant (**7k**) obtained in good yield. The mild reaction conditions and high yields achieved for these *meta*-halogenated products complements recent metal-catalyzed variants,^33^ which often require forcing reaction conditions and specific electron-poor substrates.

Until this point, functionalization of the triazene substituent had only involved conversion to a H atom. However, the full potential of this group was established by transformation to a diverse set of products.^23^ For example, Pd-catalyzed cross-coupling of triazene-containing hydroacylation adduct **3c** with an aryl boronic acid delivered the arylation product **8** in 88% yield (Scheme 6).^34^ Alternatively, treatment of **3c** with MeI provided the corresponding aryl iodide **9**,^28^ which could either be isolated, or reacted directly in a Pd-catalyzed Sonogashira-coupling reaction to deliver alkyne **10**. Reaction of **3c** with TMS-N_3_ afforded the azide-substituted enone **11** in excellent yield.^28^ The triazene group could also be converted to a deuterium atom via treatment with deuterated TFA and deuterated THF (**12**).^35^

**Scheme 6:**
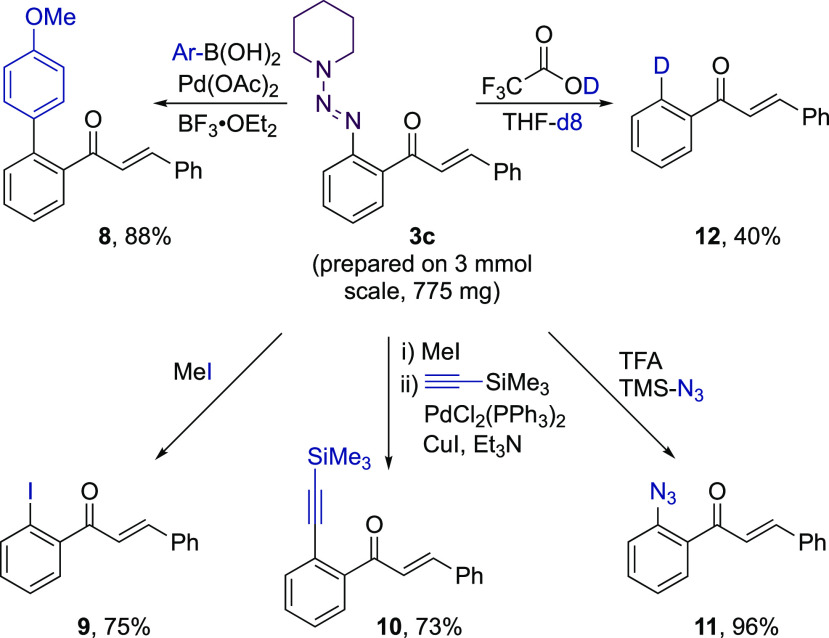
Transformations of the Triazene Group Using Hydroacylation Adduct **3c**

Having established a series of transformations that exploit the triazene substituent of hydroacylation adducts, we then combined several of these reactions with hydroacylation (Scheme 7). Because of the importance of polyaromatic compounds, the Pd-catalyzed detriazenative-arylation reaction was further studied in a sequential manner (Scheme 7a). 4-Methyl (**13a**), 2-methyl (**13b**), and 3-chloro-4-methoxy (**13c**) aryl boronic acids were combined with 3-fluoro, 4-chloro, and 6-fluoro substrates, respectively, following hydroacylation, giving the biaryl products in excellent yields. Although the addition of a Pd catalyst is required for the arylation step, the overall catalyst loading of the Rh(I) complex is reduced, no oxidant is needed, less-costly reagents are used, and at a lower temperature, when compared to the earlier reported cascade C–S activation process.^21a^ Using a single Rh(I) complex, as previously reported by our laboratory, it was possible to achieve sequential alkyne hydroacylation and aryl boronic acid conjugate addition into the enone (Scheme 7b).^18c^ The triazene group remained intact during these one-pot reactions, and it could then be exploited in a Pd-catalyzed coupling reaction with a further aryl boronic acid to afford polyaryl ketone **14** in a selective manner. Finally, alkyne hydroacylation, *para*-bromination, and *ortho*-olefination could be combined to achieve three successive C–H functionalization reactions, delivering complex pentasubstituted benzene **15** in a simple procedure. The Pd-catalyzed Suzuki-coupling of **15** was also possible, and it afforded the arylation product **16**.

**Scheme 7:**
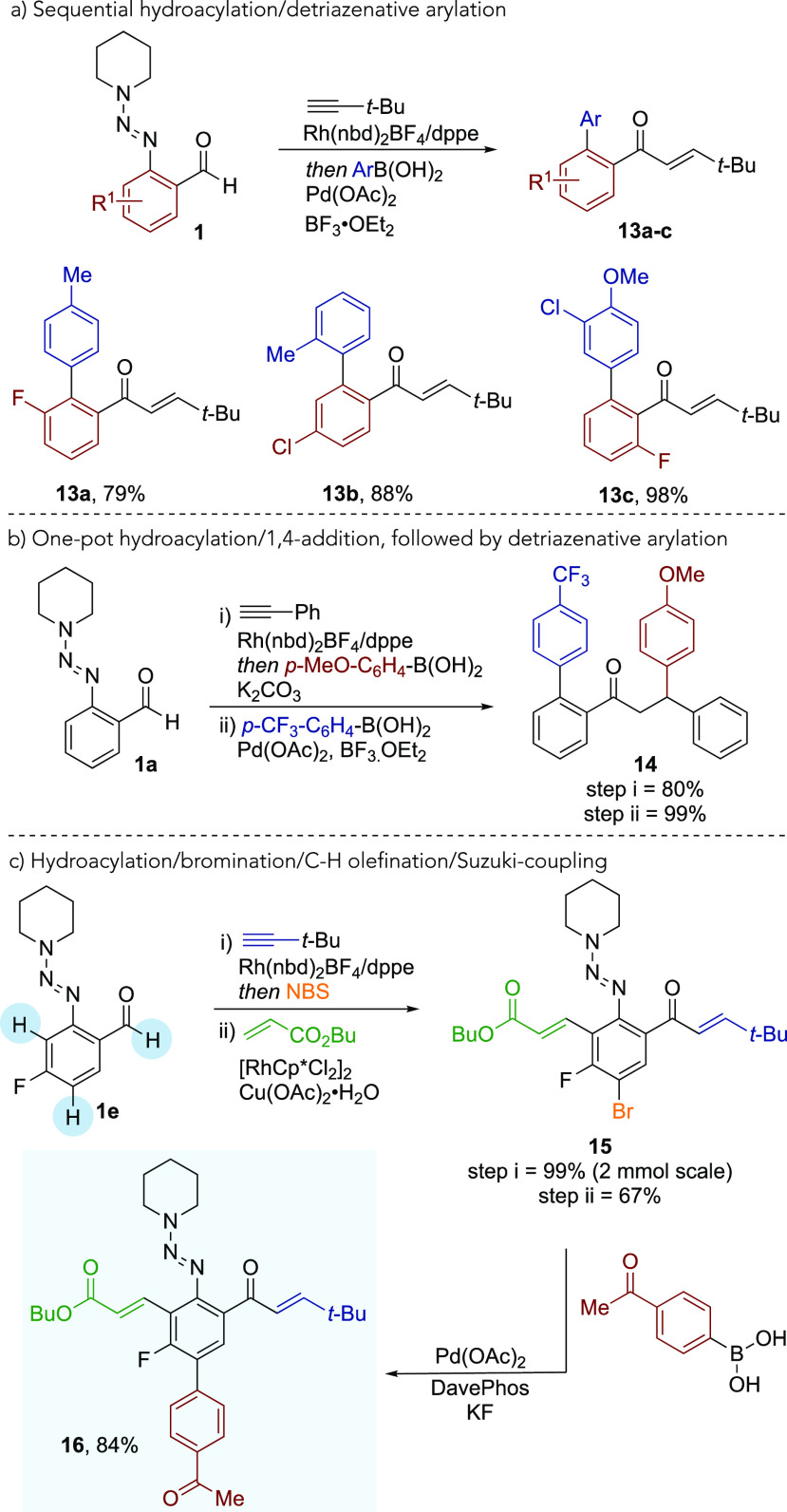
(a) Sequential Hydroacylation/Detriazenative Arylation, (b) One-Pot Hydroacylation/1,4-Conjugate Addition Followed by Arylation, and (c) Multiple C–H Functionalizations Followed by Suzuki Coupling

In summary, we have shown that a dppe-Rh(I) complex can catalyze alkyne hydroacylation of 2-triazenylbenzaldehydes. The versatility of the triazene chelating group enables a variety of sequential transformations, including *ortho*-C–H olefination, *para*-bromination, and a range of detriazenative functionalizations. Each class of sequential reaction utilizes mild reaction conditions and tolerates a broad range of functional groups, delivering traceless-, *ortho*- and *meta*-substituted hydroacylation products. The ability to link together multiple distinct transformations in a selective and efficient manner demonstrates the versatility of triazenyl aldehyde substrates for the preparation of complex benzenes, which remain valuable motifs in drug discovery.^27^

## Supporting Information Available

Experimental procedures and supporting characterization data and spectra (PDF)
