# Supplementary material for: Sequential Catalytic Functionalization of Aryltriazenyl Aldehydes for the Synthesis of Complex Benzenes
Source: ACS Catal. 2021 May 5;11(10):6091–8. doi: 10.1021/acscatal.1c01722 (PMC8291607; doi:10.1021/acscatal.1c01722)
Supplement: Supplementary file 1 — cs1c01722_si_001.pdf [file cs1c01722_si_001.pdf]

Supporting Information

Sequential Catalytic Functionalization of Aryltriazenyl Aldehydes for the Synthesis of Complex Benzenes

Sangwon Seo, Ming Gao, Eva Paffenholz and Michael Willis*

Department of Chemistry, Chemistry Research Laboratory, University of Oxford, Mansfield Road, Oxford, OX1 3TA, UK

*Correspondence to: michael.willis@chem.ox.ac.uk

Contents

I. General Considerations S2

II. Reaction Optimization S3

III. Preparation of Starting Materials S6

IV. Alkyne Hydroacylation using 2-Triazenylbenzaldehydes S16

V. Traceless Hydroacylation S19

VI. Hydroacylation/*ortho*-C–H Alkenylation/Proto-detriazenation S25

VII. Hydroacylation/Bromination/ Proto-detriazenation S36

VIII. Modification of the Triazene Group S47

IX. Sequential Functionalizations S50

X. Reference S56

Appendix *(Spectral Copies of ^{1}H, ^{13}C, and ^{19}F NMR of the Novel Compounds Obtained in this Study)* S57

I. General Considerations

Unless otherwise stated, reagents were purchased from Sigma-Aldrich Co. LLC., Fisher Scientific UK Ltd., Fluorochem Ltd., Tokyo Chemical Industry UK Ltd. or Strem Chemicals Inc. and were used as supplied. Alkenes and alkynes were distilled and stored at –20 ºC. Acetone was distilled over Drierite® under N_2 and degassed by bubbling a flow of N_2 gas for 30 min; 1,2-dichloroethane was distilled over CaH_2 under N_2 and degassed by bubbling a flow of N_2 gas for 30 min. Other solvents were collected fresh from an in-house solvent purification system, which involves passing the solvent through anhydrous alumina columns using an Innovative Technology Inc. PS-400-7 solvent purification system. Petrol refers to the fraction of light petroleum ether boiling in the range 40-60 °C.

Reactions were performed under a nitrogen atmosphere with anhydrous solvent unless otherwise stated. All glassware was oven-dried at > 100 °C, and allowed to cool to room temperature under a positive nitrogen pressure. Cooling to 0 ºC was achieved using ice-water bath. Reactions were monitored by thin layer chromatography (TLC) using pre-coated aluminum-backed silica plates (Merck Kieselgel 60 F254); plates were visualized under ultraviolet light (254 nm) followed by staining with $KMnO_4$. Flash column chromatography was carried out using Geduran® Si 60, 40-63 micron silica gel; the compound to be purified was either loaded as oil or pre-absorbed onto silica. Pressure was applied to the column head by hand bellows.

^{1}H NMR spectra were obtained on a Brüker AVIII400 (400 MHz) spectrometer using the residual solvent as an internal standard. ^{13}C NMR spectra were obtained on a Brüker AVIII400 (101 MHz) spectrometer using the residual solvent as an internal standard. ^{19}F NMR spectra were obtained on a Brüker AVIII400 (377 MHz) spectrometer using a decoupling method. Acquisitions were carried out at room temperature. Chemical shifts (δ) are reported in parts per million (ppm) from the residual solvent peak and coupling constants (*J*) were given in Hertz (Hz) and rounded to the nearest 0.5 Hz. Proton multiplicity is assigned using the following abbreviations: singlet (s), doublet (d), triplet (t), quartet (q), pentet (p), multiplet (m), broad (br.), and apparent (app.).

Low resolution ESI mass spectra were recorded on a Waters LCT Premier spectrometer. High resolution mass spectra were recorded on a Brüker MicroTOF spectrometer under electrospray ionization conditions (ESI) by the internal service at Chemistry Research Laboratory, University of Oxford. Samples for mass spectra were prepared as 1 mg/mL solution in MeOH (LRMS, HRMS-ESI). Values quoted are a ratio of mass to charge in Daltons and relative intensities of peaks observed are quoted as a percentage. High resolution values are calculated to four decimal places from the molecular formula, all found within a tolerance of five ppm. Infrared spectra were recorded as thin films ($CHCl_3$) on a Brüker Tensor 27 FT-IR spectrometer. Melting points were determined using a Stuart Scientific Melting Point Apparatus SMP1.

II. Reaction Optimization

1. Optimization of Conditions for Hydroacylation of Alkynes with Aryltriazenyl Aldehydes

A reaction vial charged with $Rh(nbd)_2BF_4$ (5 or 10 mol%) and ligand (5 or 10 mol%) was evacuated and back-filled with N_2 gas for 3 times, and to this was added solvent (0.25 mL). The resulting solution was bubbled with H_2 gas for 2 min and then with N_2 gas until the volume of the solution had become around 0.1 mL. This solution was then transferred to a N_2-purged reaction vial containing aldehyde (0.2 mmol, 1.0 equiv), with the use of additional 0.1 mL of solvent. Alkyne (1.5 or 3.0 equiv) was added and the reaction mixture was stirred at 23 ºC for 16 h. After completion, the reaction was diluted with Et_2O, filtered through a pad of silica and concentrated under reduced pressure. Product yields were determined by ^1H NMR of the crude mixture using nitromethane as an internal standard.

Table S1. Optimization of Alkyne Hydroacylation with Aryltriazenyl Aldehydes[a]

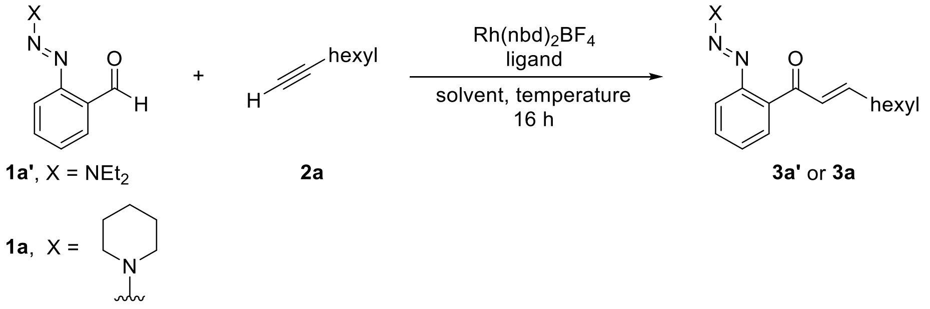

Entry	aldehyde	ligand (mol%)	solvent	T (ºC)	Yield[b] (%)
1	**1a’**	dcpm (10)	acetone	55	**3a’**, 39
2	**1a’**	dppe (10)	acetone	55	**3a’**, 44
3	**1a’**	dcpm (10)	DCE	80	**3a’**, 43
4	**1a’**	dppe (10)	DCE	80	**3a’**, 33
5	**1a’**	dcpm (10)	acetone	23	**3a’**, 70
6	**1a’**	dppe (10)	acetone	23	**3a’**, 93
7	**1a’**	dppe (5)	acetone	23	**3a’**, 33
8[c]	**1a**	dppe (5)	acetone	23	**3a**, 85
9[c]	**1a**	dppe (5)	CH_2Cl_2	23	**3a**, 96

[a]Reaction conditions: aldehyde (1.0 equiv), alkyne (3.0 equiv), $Rh(nbd)_2BF_4$ (as indicated), ligand (as indicated), solvent (1.0 M); nbd, norbornadiene; dcpm, 1,2-bis(dicyclohexylphosphino)ethane; dppe, 1,2-bis(diphenylphosphino)ethane; DCE, 1,2-dichloroethane. [b]Yields determined by ^1H NMR spectroscopy using nitromethane as an internal standard; *anti*-Markovnikov:Markovnikov >50:1 in all cases. [c]Alkyne (1.5 equiv).

2. Optimization of Conditions for the Reductive Removal of the Triazene Group

Table S2. Optimization of the Removal of the Triazene Group[a]

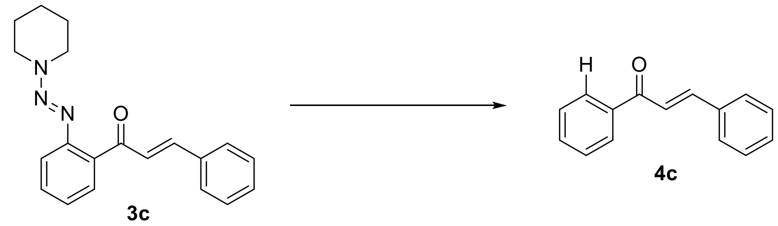

Entry	reaction conditions	Yield[b] (%)
1	$HSiCl_3$ (5.0 equiv), CH_2Cl_2, 23 ºC	0
2	$HSiEt_3$ (5.0 equiv), CH_2Cl_2, 23 ºC	0
3	Cu_2O (2.0 equiv), 1,4-dioxane, CH_2Cl_2, 40 ºC	0
4	TFA (5.0 equiv), MeOH, 65 ºC	0
5	$BF_3.OEt_2$ (5.0 equiv), 1,2-DME, 23 ºC	0
6	$BF_3.OEt_2$ (5.0 equiv), THF, 0 ºC, 1 h, then H_2O (14.0 equiv), 23 ºC, 24 h	95
7	TfOH (3.4 equiv), THF, 0 ºC, 1 h, then H_2O (14.0 equiv), 23 ºC, 24 h	90

[a]Reactions were performed under atmospheric conditions, using **3c** (0.3 mmol), reagents (as indicated) and solvent (as indicated). [b]Isolated yields.

3. Optimization of Conditions for *ortho*-C–H Olefination

Table S3. Optimization of C–H Olefination of Aryltriazenyl Enone[a]

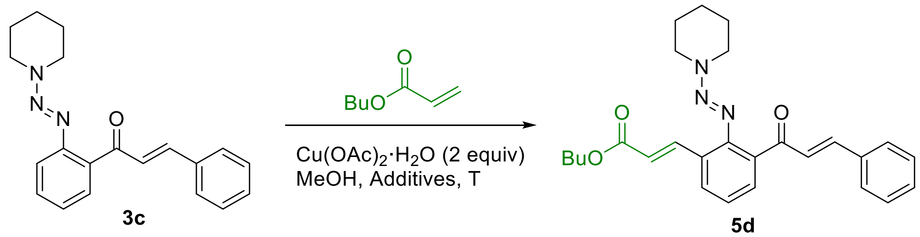

Entry	Rh	Additive	T (ºC)	Yield[b] (%)
1	$[Rh(COD)Cl]_2$ (10 mol%)	-	90	0
2	$[Rh(COD)Cl]_2$ (10 mol%)	$K_2S_2O_8$ (1 equiv)	90	0
3	$[Rh(COD)Cl]_2$ (10 mol%)	AgF (1 equiv)	90	0
4	$Rh(nbd)_2BF_4$/dcpm (10 mol%)	-	90	0
5	$Rh(nbd)_2BF_4$/dcpm (10 mol%)	$K_2S_2O_8$ (1 equiv)	90	0
6	$Rh(nbd)_2BF_4$/dppe (10 mol%)	-	90	0
7	$Rh(nbd)_2BF_4$/dppe (10 mol%)	$K_2S_2O_8$ (1 equiv)	90	0
8	$Rh(nbd)_2BF_4$/dppe (10 mol%)	AgF (1 equiv)	90	0
9	$[Rh(COD)Cl]_2$ (10 mol%)	Cp* (20 mol%)	90	35
10	$[RhCp^*Cl_2]_2$ (5 mol%)	AgOAc (30 mol%)	90	79
11	$[RhCp^*Cl_2]_2$ (5 mol%)	-	90	83
12	$[RhCp^*Cl_2]_2$ (5 mol%)	-	70	91

[a]Reaction conditions: **3c** (0.3 mmol), alkene (2.5 equiv), Rh catalyst (as indicated), $Cu(OAc)_2·H_2O$ (2.0 equiv), additive (as indicated) and MeOH (2.0 mL); COD, 1,5-cyclooctadiene; Cp*, pentamethylcyclopentadienyl. [b]Yields determined by ^1H NMR spectroscopy using nitromethane as an internal standard.

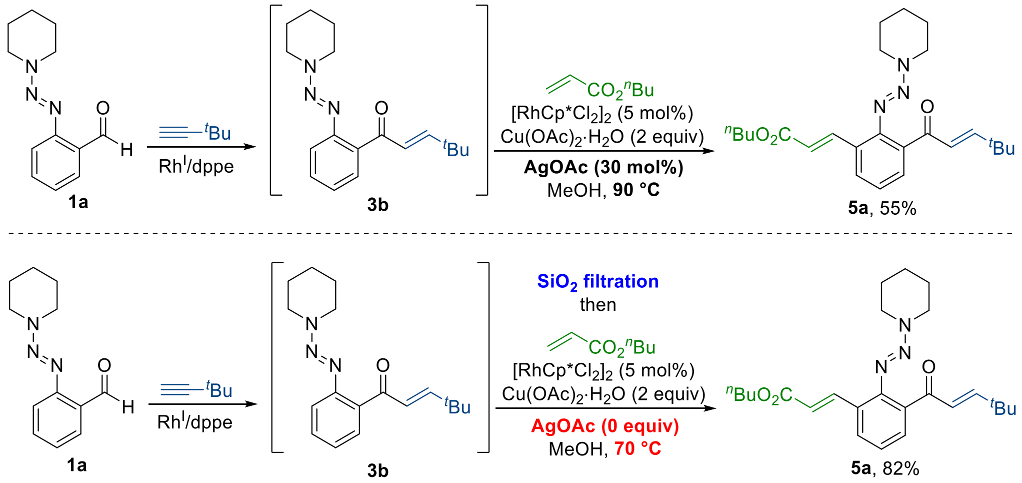

Figure S1. Optimization of Sequential Hydroacylation / *ortho*-C–H Olefination

III. Preparation of Starting Materials

1. Preparation of Weinreb Amides from Anthranilic Acids

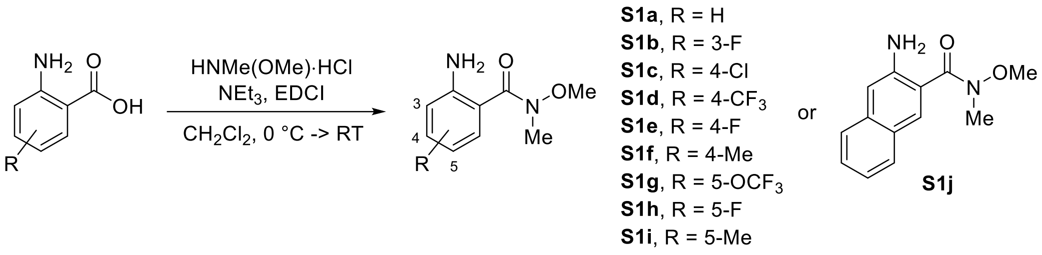

General Procedure A: Triethylamine (1.1 equiv) and *N*,*O*-dimethylhydroxylamine hydrochloride (1.1 equiv) were added to a suspension of anthranilic acid (1.0 equiv) in CH_2Cl_2 (0.1 M) at 0 °C. *N*-(3-Dimethylaminopropyl)-*N'*-ethylcarbodiimide hydrochloride (EDCI) (1.1 equiv) was then added portion-wise to the reaction mixture, and it was stirred at 23 °C until all starting material was consumed. The reaction mixture was washed with saturated solution of $NaHCO_{3(aq)}$ and brine, dried over $MgSO_4$, filtered and concentrated under reduced pressure. Purification by flash column chromatography afforded the corresponding Weinreb Amide.

Data for the obtained Weinreb amides **S1a**,[1] **S1c**,[1] **S1e**,[2] **S1f**,[1] **S1h**,[2] **S1i**,[2] and **S1j**[1] were consistent with those reported in literatures. Characterization data for those not reported previously are presented below:

2-Amino-3-fluoro-*N*-methoxy-*N*-methylbenzamide (S1b)

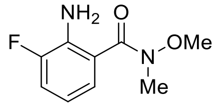

Prepared according to **General Procedure A**, using 2-amino-3-fluorobenzoic acid (1.55 g, 10 mmol), NEt_3 (1.5 mL, 11 mmol), *N*,*O*-dimethylhydroxylamine hydrochloride (1.07 g, 11 mmol), EDCI (2.11 g, 11 mmol) and CH_2Cl_2 (100 mL). Purification by flash column chromatography (CH_2Cl_2/Et_2O, 85:15) afforded the title compound (1.55 g, 78% yield) as a yellow oil. **^{1}H NMR** (400 MHz, $CDCl_3$): δ 7.20 – 7.14 (m, 1H, Ar-*H*), 7.05 – 6.97 (m, 1H, Ar-*H*), 6.64 – 6.56 (m, 1H, Ar-*H*), 4.73 (s, 2H, NH_2), 3.57 (s, 3H, OCH_3), 3.34 (s, 3H, NCH_3); **^{13}C NMR** (101 MHz, $CDCl_3$): δ 168.9, 151.9 (d, $J_{C\text{-}F}$ = 239.0 Hz), 136.0 (d, $J_{C\text{-}F}$ = 14.0 Hz), 124.6 (d, $J_{C\text{-}F}$ = 3.5 Hz), 119.1 (d, $J_{C\text{-}F}$ = 3.5 Hz), 116.7 (d, $J_{C\text{-}F}$ = 18.5 Hz), 116.1 (d, $J_{C\text{-}F}$ = 7.0 Hz), 61.3, 34.2; **^{19}F NMR** (377 MHz, $CDCl_3$): δ –134.8; **IR** ν_{max} (film)/cm^{-1} 3466, 3363, 2973, 2936, 1631, 1590, 1566, 1475, 1419, 1380, 1278, 1226, 1067, 984, 852, 786, 742; **HRMS** (ESI$^+$) calculated for $C_9H_{12}FN_2O_2$ $[M+H]^+$: 199.0877 $[M+H]^+$, found: 199.0878.

2-Amino-*N*-methoxy-*N*-methyl-4-(trifluoromethyl)benzamide (S1d)

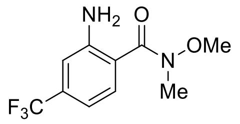

Prepared according to **General Procedure A**, using 2-amino-4-(trifluoromethyl)benzoic acid (3.08 g, 15 mmol), NEt_3 (2.3 mL, 16.5 mmol), *N*,*O*-dimethylhydroxylamine hydrochloride (1.61 g, 16.5 mmol), EDCI (3.16 g, 16.5 mmol) and CH_2Cl_2 (150 mL). Purification by flash column chromatography (gradient CH_2Cl_2/Et_2O, 95:5 to 90:10) afforded the title compound (3.16 g, 85% yield) as a yellow oil. **^{1}H NMR** (400 MHz, $CDCl_3$): δ 7.42 (d, J = 8.0 Hz, 1H, Ar-*H*), 6.92 – 6.90 (m, 1H, Ar-*H*), 6.89 – 6.85 (m, 1H, Ar-*H*), 4.84 (s, 2H, NH_2), 3.53 (s, 3H, OCH_3), 3.32 (s, 3H, NCH_3); **^{13}C NMR** (101 MHz, $CDCl_3$): δ 168.7, 147.0, 133.0 (q, $J_{C\text{-}F}$ = 32.5 Hz), 129.8, 123.8 (q, $J_{C\text{-}F}$ = 272.5 Hz), 120.0, 113.3 – 112.9 (m, 2C), 61.3, 33.7; **^{19}F NMR** (377 MHz, $CDCl_3$): δ –63.4; **IR** ν_{max} (film)/cm^{-1} 3359, 1628, 1597, 1510, 1437, 1337, 1255, 1164, 1118, 1070, 978, 927, 870, 817, 696; **HRMS** (ESI$^+$) calculated for $C_{10}H_{12}F_3N_2O_2$ $[M+H]^+$: 249.0845, found: 249.0845.

2-Amino-*N*-methoxy-*N*-methyl-5-(trifluoromethoxy)benzamide (S1g)

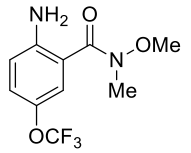

Prepared according to **General Procedure A**, using 2-amino-5-(trifluoromethoxy)benzoic acid (885 mg, 4.0 mmol), NEt_3 (0.61 mL, 4.4 mmol), *N*,*O*-dimethylhydroxylamine hydrochloride (429 mg, 4.4 mmol), EDCI (844 mg, 4.4 mmol) and CH_2Cl_2 (40 mL). Purification by flash column chromatography (gradient CH_2Cl_2/Et_2O, 90:10 to 85:15) afforded the title compound (977 mg, 74% yield) as a yellow oil. **^{1}H NMR** (400 MHz, $CDCl_3$): δ 7.32 – 7.29 (m, 1H, Ar-*H*), 7.06 – 7.01 (m, 1H, Ar-*H*), 6.65 (d, J = 9.0 Hz, 1H, Ar-*H*), 4.78 (s, 2H, NH_2), 3.55 (s, 3H, OCH_3), 3.32 (s, 3H, NCH_3); **^{13}C NMR** (101 MHz, $CDCl_3$): δ 168.6, 146.1, 139.3 (q, $J_{C\text{-}F}$ = 2.0 Hz), 125.0, 122.6, 120.8 (q, $J_{C\text{-}F}$ = 256.0 Hz), 117.3, 116.8, 61.3, 33.8; **^{19}F NMR** (377 MHz, $CDCl_3$): δ –58.8; **IR** ν_{max} (film)/cm^{-1} 3360, 1631, 1593, 1499, 1434, 1383, 1252, 1208, 1147, 985, 957, 890, 825; **HRMS** (ESI$^+$) calculated for $C_{10}H_{12}F_3N_2O_3$ $[M+H]^+$: 265.0795, found: 265.0805.

2. Preparation of Aryltriazenyl Aldehydes via Reduction of Weinreb Amides

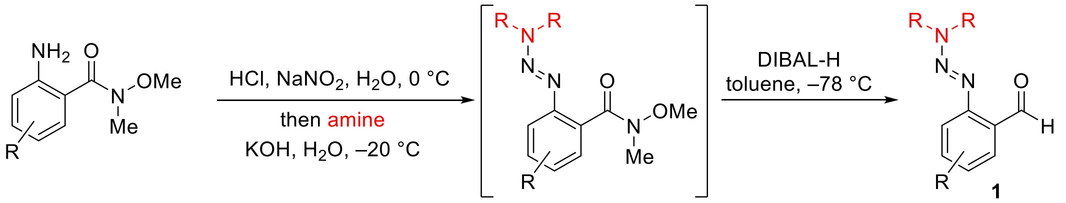

General Procedure B: A cold solution of sodium nitrite (2.0 equiv) in water was slowly added to a suspension of 2-aminobenzamide (1.0 equiv) in conc. $HCl_{(aq)}$ at 0 °C. The mixture was stirred for 10 min before it was poured into a solution of KOH (2.0 equiv) and amine (2.0 equiv) in water at –20 °C. It was then stirred for an additional 10 min, diluted in water and warmed to room temperature. The aqueous phase was extracted with Et_2O, and the combined organic layers were dried over $MgSO_4$, filtered and concentrated under reduced pressure to give aryltriazenyl Weinreb amide, which was used in the reduction step without further purification.

Under N_2 atmosphere, a solution of DIBAL-H (1.0 M in toluene, 1.1 or 1.2 equiv) was added dropwise to a solution of the above obtained amide (1.0 equiv) in toluene (0.1 M) at –78 °C. The reaction mixture was stirred at –78 °C for 3 h. EtOAc and saturated aqueous solution of sodium potassium tartrate were then added, and the mixture was allowed to warm to 25 °C. The organic phase was further washed with saturated aqueous solution of sodium potassium tartrate and brine, dried over $MgSO_4$, filtered and concentrated under reduced pressure. Purification by flash column chromatography afforded the corresponding aryltriazenyl aldehyde.

(*E*)-2-(3,3-Diethyltriaz-1-en-1-yl)benzaldehyde (1a')

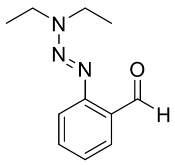

Prepared according to **General Procedure B**, using: i) 2-amino-*N*-methoxy-*N*-methylbenzamide **S1a** (1.80 g, 10 mmol), conc. HCl (3.5 mL), sodium nitrite (1.38 g, 20 mmol, in 2 mL H_2O), KOH (1.12 g, 20 mmol), diethylamine (2.1 mL, 20 mmol) and H_2O (20.0 mL); ii) DIBAL-H (12.0 mL, 12 mmol) and toluene (100 mL). Purification by flash column chromatography (petrol/Et_2O, 9:1) afforded the title compound (1.85 g, 90% yield) as a yellow oil. **^{1}H NMR** (400 MHz, $CDCl_3$): δ 10.87 (d, J = 1.0 Hz, 1H, C*H*O), 7.87 – 7.85 (m, 1H, Ar-*H*), 7.55 – 7.48 (m, 2H, Ar-*H*), 7.21 – 7.16 (m, 1H, Ar-*H*), 3.82 (br. s, 4H, N(C*H*$_2$)$_2$), 1.35 (br. s, 3H, C*H*$_3$), 1.25 (br. s, 3H, C*H*$_3$); **^{13}C NMR** (101 MHz, $CDCl_3$): δ 192.9, 152.9, 134.8, 129.0, 127.0, 125.0, 117.6, 49.5, 42.0, 14.6, 11.3; **IR** ν_{max} (film)/cm^{-1} 3067, 1684, 1593, 1400, 1338, 1193, 1084, 727; **HRMS** (ESI$^+$) calculated for $C_{12}H_{16}N_3O$ [M+H]$^+$: 206.1288, found: 206.1291.

(*E*)-2-(Piperidin-1-yldiazenyl)benzaldehyde (1a)

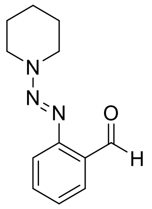

Prepared according to **General Procedure B**, using: i) 2-amino-*N*-methoxy-*N*-methylbenzamide **S1a** (5.40 g, 30 mmol), conc. HCl (10.0 mL), sodium nitrite (4.14 g, 60 mmol, in 5 mL H_2O), KOH (3.37 g, 60 mmol), piperidine (5.9 mL, 60 mmol) and H_2O (50.0 mL); ii) DIBAL-H (36.0 mL, 36 mmol) and toluene (300 mL). Purification by flash column chromatography (petrol/Et_2O, 9:1) afforded the title compound (5.93 g, 92% yield) as a pale yellow solid. **m.p.** (petrol/Et_2O): 33 – 34 ºC; **^{1}H NMR** (400 MHz, $CDCl_3$): δ 10.83 (d, J = 1.0 Hz, 1H, C*H*O), 7.87 – 7.83 (m, 1H, Ar-*H*), 7.55 – 7.47 (m, 2H, Ar-*H*), 7.22 – 7.17 (m, 1H, Ar-*H*), 3.85 (br. s, 4H, N(CH_2)$_2$), 1.71 (br. s, 6H, 3 × CH_2); **^{13}C NMR** (101 MHz, $CDCl_3$): δ 192.6, 152.5, 134.7, 129.0, 127.0, 125.4, 117.6, 53.2, 44.0, 26.4, 24.4, 24.3; **IR** ν_{max} (film)/cm^{-1} 2939, 2856, 1683, 1592, 1413, 1353, 1293, 1187, 1108, 1001, 761; **HRMS** (ESI$^+$) calculated for $C_{12}H_{16}N_3O$ [M+H]$^+$: 218.1288, found: 218.1289.

(*E*)-3-Fluoro-2-(piperidin-1-yldiazenyl)benzaldehyde (1b)

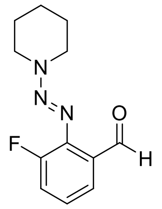

Prepared according to **General Procedure B**, using: i) 2-amino-3-fluoro-*N*-methoxy-*N*-methylbenzamide **S1b** (1.50 g, 7.6 mmol), conc. HCl (2.6 mL), sodium nitrite (1.05 g, 15.2 mmol, in 2 mL H_2O), KOH (853 mg, 15.2 mmol), piperidine (1.5 mL, 15.2 mmol) and H_2O (12 mL); ii) DIBAL-H (8.0 mL, 8.0 mmol) and toluene (67 mL). Purification by flash column chromatography (petrol/Et_2O, 9:1), followed by recrystallization (petrol/CH_2Cl_2), afforded the title compound (0.70 g, 39% yield) as a yellow solid. **m.p.** (petrol/CH_2Cl_2): 66 – 68 ºC; **^{1}H NMR** (400 MHz, $CDCl_3$): δ 10.16 (d, J = 0.5 Hz, 1H, C*H*O), 7.65 – 7.60 (m, 1H, Ar-*H*), 7.34 – 7.28 (m, 1H, Ar-*H*), 7.17 (tdd, J = 8.0, 4.5, 0.5 Hz, 1H, Ar-*H*), 4.00 – 3.80 (2 × br. s, 4H, N(CH_2)$_2$), 1.75 (br. s, 6H, 3 × CH_2); **^{13}C NMR** (101 MHz, $CDCl_3$): δ 190.8 (d, J_{C-F} = 3.0 Hz), 155.4 (d, J_{C-F} = 252.0 Hz), 141.8 (d, J_{C-F} = 9.0 Hz), 131.1, 125.3 (d, J_{C-F} = 8.0 Hz), 123.2 (d, J_{C-F} = 4.0 Hz), 121.2 (d, J_{C-F} = 20.5 Hz), 53.3, 43.9, 26.4, 24.3, 24.2; **^{19}F NMR** (377 MHz, $CDCl_3$): δ –126.3; **IR** ν_{max} (film)/cm^{-1} 2941, 2858, 1684, 1601, 1573, 1415, 1354, 1286, 1250, 1182, 1108, 1014, 968, 853, 789, 765, 733; **HRMS** (ESI$^+$) calculated for $C_{12}H_{15}FN_3O$ [M+H]$^+$: 236.1194, found: 236.1193.

(*E*)-4-Chloro-2-(piperidin-1-yldiazenyl)benzaldehyde (1c)

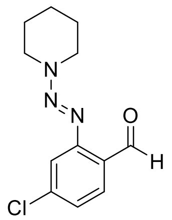

Prepared according to **General Procedure B**, using: i) 2-amino-4-chloro-*N*-methoxy-*N*-methylbenzamide **S1c** (1.25 g, 5.8 mmol), conc. HCl (2.0 mL), sodium nitrite (800 mg, 11.6 mmol, in 2 mL H_2O), KOH (651 mg, 11.6 mmol), piperidine (1.1 mL, 11.6 mmol) and H_2O (8.0 mL); ii) DIBAL-H (6.2 mL, 6.2 mmol) and toluene (52 mL). Purification by flash column chromatography (petrol/Et_2O, 9:1) afforded the title compound (1.20 g, 82% yield) as a pale yellow solid. **m.p.** (petrol/Et_2O): 46 – 47 ºC; **^{1}H NMR** (400 MHz, $CDCl_3$): δ 10.76 (d, J = 1.0 Hz, 1H, C*H*O), 7.78 (d, J = 8.5 Hz, 1H, Ar-*H*), 7.56 (d, J = 2.0 Hz, 1H, Ar-*H*), 7.13 (ddd, J = 8.5, 2.0, 1.0 Hz, 1H, Ar-*H*), 3.88 (br. s, 4H, N(CH_2)$_2$), 1.84 – 1.62 (m, 6H, 3 × CH_2); **^{13}C NMR** (101 MHz, $CDCl_3$): δ 191.5, 153.3, 141.0, 128.5, 127.3, 125.4, 117.4, 53.5, 44.1, 26.5, 24.5, 24.2; **IR** ν_{max} (film)/cm^{-1} 2941, 2857, 1683, 1585, 1421, 1394, 1375, 1354, 1339, 1184, 1111, 831; **HRMS** (ESI$^+$) calculated for $C_{12}H_{15}ClN_3O$ [M+H]$^+$: 252.0898, found: 252.0898.

(*E*)-2-(Piperidin-1-yldiazenyl)-4-(trifluoromethyl)benzaldehyde (1d)

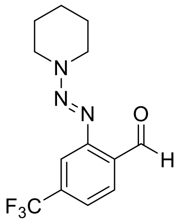

Prepared according to **General Procedure B**, using: i) 2-amino-*N*-methoxy-*N*-methyl-4-(trifluoromethyl)benzamide **S1d** (3.06 g, 12.3 mmol), conc. HCl (4.1 mL), sodium nitrite (1.70 g, 24.6 mmol, in 3 mL H_2O), KOH (1.38 g, 24.6 mmol), piperidine (2.4 mL, 24.6 mmol) and H_2O (21 mL) to afford the corresponding aryltriazenyl amide (3.43 g, 10.0 mmol) as a crude; ii) DIBAL-H (11.0 mL, 11.0 mmol) and toluene (110 mL). Purification by flash column chromatography (petrol/Et_2O, 9:1) afforded the title compound (1.43 g, 41% yield) as a pale yellow solid. **m.p.** (petrol/Et_2O): 108 – 110 ºC; **^{1}H NMR** (400 MHz, $CDCl_3$): δ 10.84 (d, J = 1.0 Hz, 1H, C*H*O), 7.94 (d, J = 8.0 Hz, 1H, Ar-*H*), 7.85 (s, 1H, Ar-*H*), 7.41 (d, J = 8.0 Hz, 1H, Ar-*H*), 3.98 – 3.86 (2 × br. s, 4H, N(CH_2)$_2$), 1.88 – 1.66 (m, 6H, 3 × CH_2); **^{13}C NMR** (101 MHz, $CDCl_3$): δ 191.8, 152.7, 135.8 (q, J_{C-F} = 32.5 Hz), 130.8, 127.9, 123.8 (q, J_{C-F} = 273.0 Hz), 121.3 (q, J_{C-F} = 3.5 Hz), 115.0 (q, J_{C-F} = 4.0 Hz), 53.6, 44.2, 26.6, 24.5, 24.2; **^{19}F NMR** (377 MHz, $CDCl_3$): δ –63.3; **IR** ν_{max} (film)/cm^{-1} 2946, 2863, 1680, 1394, 1327, 1159, 1122, 1065, 903, 832; **HRMS** (ESI$^+$) calculated for $C_{13}H_{15}F_3N_3O$ [M+H]$^+$: 286.1162, found: 286.1161.

(*E*)-4-Fluoro-2-(piperidin-1-yldiazenyl)benzaldehyde (1e)

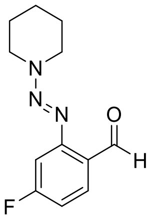

Prepared according to **General Procedure B**, using: i) 2-amino-4-fluoro-*N*-methoxy-*N*-methylbenzamide **S1e** (1.33 g, 6.7 mmol), conc. HCl (2.3 mL), sodium nitrite (925 mg, 13.4 mmol, in 2 mL H_2O), KOH (752 mg, 13.4 mmol), piperidine (1.3 mL, 13.4 mmol) and H_2O (10 mL); ii) DIBAL-H (8.0 mL, 8.0 mmol) and toluene (67 mL). Purification by flash column chromatography (petrol/Et_2O, 9:1) afforded the title compound (1.48 g, 94% yield) as a pale yellow solid. **m.p.** (petrol/Et_2O): 39 – 41 ºC; **^{1}H NMR** (400 MHz, $CDCl_3$): δ 10.73 (d, J = 0.5 Hz, 1H, C*H*O), 7.85 (dd, J = 8.5, 6.5 Hz, 1H, Ar-*H*), 7.22 (dd, J = 11.0, 2.5 Hz, 1H, Ar-*H*), 6.87 – 6.80 (m, 1H, Ar-*H*), 3.86 (br. s, 4H, N(CH_2)$_2$), 1.84 – 1.60 (m, 6H, 3 × CH_2); **^{13}C NMR** (101 MHz, $CDCl_3$): δ 191.0, 166.9 (d, J_{C-F} = 255.0 Hz), 155.0 (d, J_{C-F} = 8.5 Hz), 129.7 (d, J_{C-F} = 10.5 Hz), 125.8 (d, J_{C-F} = 2.5 Hz), 112.7 (d, J_{C-F} = 23.0 Hz), 103.5 (d, J_{C-F} = 23.0 Hz), 53.4, 44.1, 26.5, 24.4, 24.2; **^{19}F NMR** (377 MHz, $CDCl_3$): δ –102.5; **IR** ν_{max} (film)/cm^{-1} 2942, 2858, 1684, 1598, 1581, 1404, 1291, 1240, 1200, 1111, 1004, 864, 812, 656; **HRMS** (ESI$^+$) calculated for $C_{12}H_{15}FN_3O$ [M+H]$^+$: 236.1194, found: 236.1194.

(*E*)-4-Methyl-2-(piperidin-1-yldiazenyl)benzaldehyde (1f)

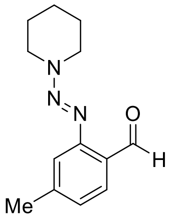

Prepared according to **General Procedure B**, using: i) 2-amino-*N*-methoxy-*N*,4-dimethylbenzamide **S1f** (1.88 g, 9.7 mmol), conc. HCl (3.3 mL), sodium nitrite (1.34 g, 19.4 mmol, in 2 mL H_2O), KOH (1.09 g, 19.4 mmol), piperidine (1.9 mL, 19.4 mmol) and H_2O (16 mL); ii) DIBAL-H (10.6 mL, 10.6 mmol) and toluene (100 mL). Purification by flash column chromatography (petrol/Et_2O, 9:1) afforded the title compound (2.06 g, 92% yield) as a yellow oil. **^{1}H NMR** (400 MHz, $CDCl_3$): δ 10.76 (d, J = 1.0 Hz, 1H, C*H*O), 7.76 (d, J = 8.0 Hz, 1H, Ar-*H*), 7.31 (s, 1H, Ar-*H*), 7.03 – 6.98 (m, 1H, Ar-*H*), 3.83 (br. s, 4H, N(CH_2)$_2$), 2.36 (s, 3H, CH_3), 1.70 (br. s, 6H, 3 × CH_2); **^{13}C NMR** (101 MHz, $CDCl_3$): δ 192.2, 152.4, 145.7, 127.0, 126.9, 126.5, 117.7, 53.1, 44.0, 26.1, 24.4, 24.3, 22.1; **IR** $\boldsymbol{\nu}_{\mathbf{max}}$ (film)/cm^{-1} 2939, 2854, 1679, 1600, 1422, 1353, 1291, 1190, 1096, 1001, 813, 648; **HRMS** (ESI$^+$) calculated for $C_{13}H_{18}N_3O$ [M+H]$^+$: 232.1444, found: 232.1446.

(*E*)-2-(Piperidin-1-yldiazenyl)-5-(trifluoromethoxy)benzaldehyde (1g)

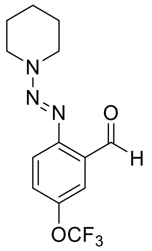

Prepared according to **General Procedure B**, using: i) 2-amino-*N*-methoxy-*N*-methyl-5-(trifluoromethoxy)benzamide **S1g** (876 mg, 3.3 mmol), conc. HCl (1.1 mL), sodium nitrite (458 mg, 6.6 mmol, in 0.8 mL H_2O), KOH (372 mg, 6.6 mmol), piperidine (0.66 mL, 6.6 mmol) and H_2O (6.0 mL) to afford the corresponding aryltriazenyl amide (1.07 g, 3.0 mmol) as a crude; ii) DIBAL-H (3.3 mL, 3.3 mmol) and toluene (30 mL). Purification by flash column chromatography (gradient petrol/Et_2O, 95:5 to 90:10) afforded the title compound (754 mg, 75% yield) as a pale orange solid. **m.p.** (petrol/Et_2O): 35 – 36 ºC; **^{1}H NMR** (400 MHz, $CDCl_3$): δ 10.77 (s, 1H, C*H*O), 7.68 – 7.65 (m, 1H, Ar-*H*), 7.58 (d, J = 9.0 Hz, 1H, Ar-*H*), 7.33 – 7.29 (m, 1H, Ar-*H*), 3.86 (br. s, 4H, N(CH_2)$_2$), 1.73 (br. s, 6H, 3 × CH_2); **^{13}C NMR** (101 MHz, $CDCl_3$): δ 191.1, 151.1, 146.6 (q, J = 2.0 Hz), 129.6, 127.4, 120.6 (q, J = 257.5 Hz), 119.4, 118.8, 53.3, 44.1, 26.5, 24.4, 24.2; **^{19}F NMR** (377 MHz, $CDCl_3$): δ –58.1; **IR** ν_{max} (film)/cm^{-1} 2944, 1686, 1410, 1244, 1212, 1175, 1141, 1108, 1002, 887, 834; **HRMS** (ESI$^+$) calculated for $C_{13}H_{15}F_3N_3O_2$ [M+H]$^+$: 302.1111, found: 302.1110.

(*E*)-5-Fluoro-2-(piperidin-1-yldiazenyl)benzaldehyde (1h)

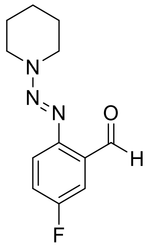

Prepared according to **General Procedure B**, using: i) 2-amino-5-fluoro-*N*-methoxy-*N*-methylbenzamide **S1h** (687 mg, 3.5 mmol), conc. HCl (1.2 mL), sodium nitrite (479 mg, 7.0 mmol, in 0.8 mL H_2O), KOH (389 mg, 7.0 mmol), piperidine (0.69 mL, 7.0 mmol) and H_2O (6.0 mL) to afford the corresponding aryltriazenyl amide (725 mg, 2.5 mmol) as a crude; ii) DIBAL-H (2.7 mL, 2.7 mmol) and toluene (25 mL). Purification by flash column chromatography (gradient petrol/Et_2O, 95:5 to 90:10) afforded the title compound (458 mg, 56% yield) as an orange oil. **^{1}H NMR** (400 MHz, $CDCl_3$): δ 10.76 (d, J = 3.5 Hz, 1H, C*H*O), 7.53 (dd, J = 9.0, 5.0 Hz, 1H, Ar-*H*), 7.48 (dd, J = 8.5, 3.0 Hz, 1H, Ar-*H*), 7.19 (ddd, J = 9.0, 8.0, 3.0 Hz, 1H, Ar-*H*), 3.82 (br. s, 4H, N(CH_2)$_2$), 1.71 (br. s, 6H, 3 × CH_2); **^{13}C NMR** (101 MHz, $CDCl_3$): δ 191.4, 160.6 (d, $J_{C\text{-}F}$ = 246.0 Hz), 149.1 (d, $J_{C\text{-}F}$ = 2.5 Hz), 130.0 (d, $J_{C\text{-}F}$ = 6.0 Hz), 122.0 (d, $J_{C\text{-}F}$ = 23.5 Hz), 119.6 (d, $J_{C\text{-}F}$ = 7.5 Hz), 112.4 (d, $J_{C\text{-}F}$ = 22.5 Hz), 53.1, 44.0, 26.3,

24.4, 24.2; **^{19}F NMR** (377 MHz, $CDCl_3$): δ –117.1; **IR** ν_{max} (film)/cm^{-1} 2941, 2858, 1681, 1478, 1411, 1254, 1106, 1002, 828, 736, 658; **HRMS** (ESI$^+$) calculated for $C_{12}H_{15}FN_3O$ [M+H]$^+$: 236.1194, found: 236.1194.

(*E*)-5-Methyl-2-(piperidin-1-yldiazenyl)benzaldehyde (1i)

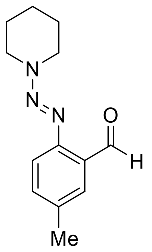

Prepared according to **General Procedure B**, using: i) 2-amino-*N*-methoxy-*N*,5-dimethylbenzamide **S1i** (722 mg, 3.7 mmol), conc. HCl (1.3 mL), sodium nitrite (511 mg, 7.4 mmol, in 2 mL H_2O), KOH (415 mg, 7.4 mmol), piperidine (0.73 mL, 7.4 mmol) and H_2O (6.2 mL) to afford the corresponding aryltriazenyl amide (909 mg, 3.1 mmol) as a crude; ii) DIBAL-H (3.7 mL, 3.7 mmol) and toluene (31 mL). Purification by flash column chromatography (petrol/Et_2O, 9:1) afforded the title compound (665 mg, 78% yield) as a pale yellow solid. **m.p.** (petrol/Et_2O): 50 – 51 ºC; **^{1}H NMR** (400 MHz, $CDCl_3$): δ 10.81 (s, 1H, C*H*O), 7.66 (d, *J* = 1.5 Hz, 1H, Ar-*H*), 7.45 (d, *J* = 8.5 Hz, 1H, Ar-*H*), 7.33 (dd, *J* = 8.5, 2.0 Hz, 1H, Ar-*H*), 3.84 (br. s, 4H, N(CH_2)$_2$), 2.36 (s, 3H, CH_3), 1.73 (br. s, 6H, 3 × CH_2); **^{13}C NMR** (101 MHz, $CDCl_3$): δ 192.7, 150.5, 135.7, 135.1, 128.8, 126.8, 117.5, 52.9, 44.0, 25.9, 24.8, 24.3, 20.9; **IR** ν_{max} (film)/cm^{-1} 2939, 2855, 1681, 1607, 1482, 1426, 1353, 1321, 1220, 1182, 1147, 1100, 1001, 827, 729, 662; **HRMS** (ESI$^+$) calculated for $C_{13}H_{18}N_3O$ [M+H]$^+$: 232.1444, found: 232.1445.

(*E*)-3-(Piperidin-1-yldiazenyl)-2-naphthaldehyde (1j)

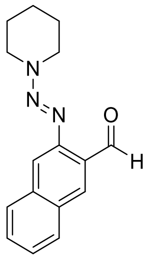

Prepared according to **General Procedure B**, using: i) 3-amino-*N*-methoxy-*N*-methyl-2-naphthamide **S1j** (839 mg, 3.7 mmol), conc. HCl (1.2 mL), sodium nitrite (504 mg, 7.4 mmol, in 1 mL H_2O), KOH (410 mg, 7.4 mmol), piperidine (0.72 mL, 7.4 mmol) and H_2O (6 mL) to afford the corresponding aryltriazenyl amide (937 mg, 2.9 mmol) as a crude; ii) DIBAL-H (3.2 mL, 3.2 mmol) and toluene (29 mL). Purification by flash column chromatography (gradient petrol/Et_2O, 95:5 to 90:10) afforded the title compound (634 mg, 65% yield) as a yellow solid. **m.p.** (petrol/Et_2O): 93 – 95 ºC; **^{1}H NMR** (400 MHz, $CDCl_3$): δ 10.89 (s, 1H, C*H*O), 8.41 (s, 1H, Ar-*H*), 7.90 (d, *J* = 8.0 Hz, 1H, Ar-*H*), 7.84 (s, 1H,

Ar-*H*), 7.81 (d, J = 8.0 Hz, 1H, Ar-*H*), 7.53 – 7.47 (m, 1H, Ar-*H*), 7.43 – 7.37 (m, 1H, Ar-*H*), 3.89 (br. s, 4H, N(CH_2)$_2$), 1.74 (br. s, 6H, 3 × CH_2); **^{13}C NMR** (101 MHz, $CDCl_3$): δ 192.8, 148.4, 137.0, 130.9, 129.9, 129.0, 128.9, 128.7, 128.1, 125.7, 114.4, 53.3, 44.0, 25.9, 25.0, 24.3; **IR** ν_{max} (film)/cm^{-1} 2939, 2856, 1684, 1621, 1587, 1417, 1328, 1189, 1107, 1088, 1003, 886, 747; **HRMS** (ESI$^+$) calculated for $C_{16}H_{18}N_3O$ [M+H]$^+$: 268.1444, found: 268.1444.

3. Preparation of Aryltriazenyl Aldehyde via Oxidation of Aryltriazenyl methanol

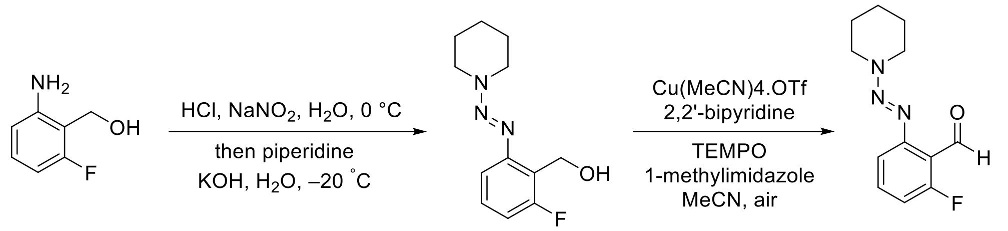

1k

(*E*)-[2-Fluoro-6-(piperidin-1-yldiazenyl)phenyl]methanol

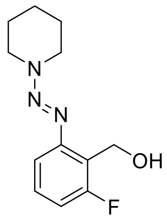

A cold solution of sodium nitrite (1.42 g, 20.6 mmol) in water (2 mL) was slowly added to a suspension of (2-amino-6-fluorophenyl)methanol[3] (1.46 g, 10.3 mmol) in conc. HCl (3.6 mL) at 0 °C. The mixture was stirred for 10 min before it was poured into a solution of KOH (1.16 g, 20.6 mmol) and piperidine (2.0 mL, 20.6 mmol) in water (20 mL) at –20 °C. It was then stirred for an additional 10 min, diluted in water and warmed to room temperature. The aqueous phase was extracted with Et_2O (2×), and the combined organic layers were dried over $MgSO_4$, filtered and concentrated under reduced pressure. Purification by flash column chromatography (petrol/Et_2O, 3:2) afforded the title compound (2.39 g, 98% yield) as a yellow oil. **^{1}H NMR** (400 MHz, $CDCl_3$): δ 7.29 (d, J = 8.0 Hz, 1H, Ar-*H*), 7.18 (dd, J = 14.5, 8.0 Hz, 1H, Ar-*H*), 6.84 (t, J = 8.5 Hz, 1H, Ar-*H*), 4.91 (d, J = 6.5 Hz, 2H, Ar-CH_2), 3.84 – 3.76 (m, 5H, N(CH_2)$_2$ and O*H*), 1.72 (br. s, 6H, 3 × CH_2); **^{13}C NMR** (101 MHz, $CDCl_3$): δ 160.4 (d, $J_{C\text{-}F}$ = 244.0 Hz), 150.7 (d, $J_{C\text{-}F}$ = 6.0 Hz), 128.8 (d, $J_{C\text{-}F}$ = 9.5 Hz), 121.7 (d, $J_{C\text{-}F}$ = 17.0 Hz), 112.4 (d, $J_{C\text{-}F}$ = 3.0 Hz), 112.1 (d, $J_{C\text{-}F}$ = 23.0 Hz), 55.9 (d, $J_{C\text{-}F}$ = 6.5 Hz), 53.1, 43.9, 26.0, 24.2, 24.1; **^{19}F NMR** (377 MHz, $CDCl_3$): δ –119.4; **IR** ν_{max} (film)/cm^{-1} 3433 (br.), 2940, 2857, 1609, 1579, 1419, 1355, 1294, 1242, 1183, 1110, 1011, 853, 835, 786, 745, 719; **HRMS** (ESI$^+$) calculated for $C_{12}H_{17}FN_3O$ [M+H]$^+$: 238.1350, found: 238.1350.

(*E*)-2-Fluoro-6-(piperidin-1-yldiazenyl)benzaldehyde (1k)

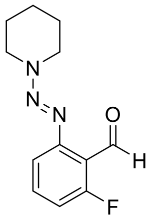

A solution of the above obtained (*E*)-[2-fluoro-6-(piperidin-1-yldiazenyl)phenyl]methanol (2.37 g, 10 mmol), Cu(MeCN)$_4$·OTf (188 mg, 0.5 mmol), 2,2'-bipyridine (78 mg, 0.5 mmol), TEMPO (78 mg, 0.5 mmol) and 1-methylimidazole (80 μL, 1.0 mmol) in acetonitrile (50 mL) was stirred under air for 2.5 h. The mixture was filtered through a pad of SiO_2 and concentrated under reduced pressure. Purification by flash column chromatography (petrol/Et_2O, 9:1) afforded the title compound (2.11 g, 90% yield) as a yellow solid. **m.p.** (petrol/Et_2O): 36 – 38 ºC; **^{1}H NMR** (400 MHz, $CDCl_3$): δ 10.70 (s, 1H, C*H*O), 7.41 (td, J = 8.0, 6.0 Hz, 1H, Ar-*H*), 7.30 (d, J = 8.0 Hz, 1H, Ar-*H*), 6.89 – 6.81 (m, 1H, Ar-*H*), 3.84 (br. s, 4H, N(CH_2)$_2$), 1.82 – 1.62 (m, 6H, 3 × CH_2); **^{13}C NMR** (101 MHz, $CDCl_3$): δ 190.3, 161.6 (d, J_{C-F} = 261.0 Hz), 153.9 (d, J_{C-F} = 3.5 Hz), 134.8 (d, J_{C-F} = 11.0 Hz), 118.0 (d, J_{C-F} = 8.5 Hz), 113.4 (d, J_{C-F} = 3.5 Hz), 112.6 (d, J_{C-F} = 21.5 Hz), 53.3, 44.1, 26.5, 24.4, 24.2; **^{19}F NMR** (377 MHz, $CDCl_3$): δ –116.2; **IR** ν_{max} (film)/cm^{-1} 2941, 2859, 1691, 1602, 1568, 1405, 1296, 1250, 1198, 1180, 1110, 1014, 868, 851, 791, 718; **HRMS** (ESI$^+$) calculated for $C_{12}H_{15}FN_3O$ [M+H]$^+$: 236.1194, found: 236.1193.

IV. Alkyne Hydroacylation using 2-Triazenylbenzaldehydes

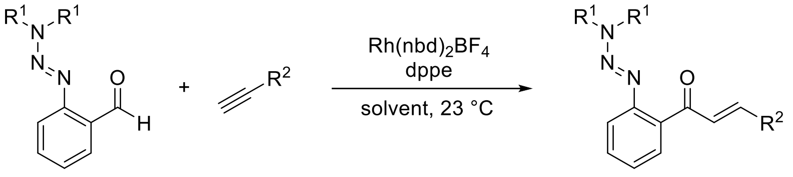

1a' or **1a** **3a'**, **3a–3c**

General Procedure C: A reaction vial charged with $Rh(nbd)_2BF_4$ (5 or 10 mol%) and 1,2-bis(diphenylphosphino)ethane (dppe) (5 or 10 mol%) was evacuated and back-filled with N_2 gas for 3 times, and to this was added solvent (0.25 mL). The resulting solution was bubbled with H_2 gas for 2 min and then with N_2 gas until the volume of the solution had become around 0.1 mL. This solution was then transferred to a N_2-purged reaction vial containing aldehyde (0.2 mmol, 1.0 equiv), with the use of additional 0.1 mL of solvent. Alkyne (1.5 or 3.0 equiv) was added and the reaction mixture was stirred at 23 ºC for 16 h. After completion, the reaction was diluted with Et_2O, filtered through a pad of silica and concentrated under reduced pressure. Purification by flash column chromatography afforded the corresponding hydroacylation product.

(*E*)-1-{2-[(*E*)-3,3-diethyltriaz-1-en-1-yl]phenyl}non-2-en-1-one (3a')

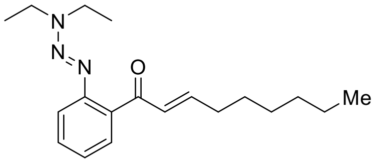

Prepared according to **General Procedure C**, using aldehyde **1a'** (41 mg, 0.2 mmol), 1-octyne (89 µL, 0.6 mmol, 3.0 equiv), $Rh(nbd)_2BF_4$ (7.5 mg, 0.02 mmol, 10 mol%), dppe (8.0 mg, 0.02 mmol, 10 mol%) and acetone. Purification by flash column chromatography (gradient petrol/Et_2O, 10:1) afforded the title compound (56 mg, 89% yield) as a yellow oil. **^{1}H NMR** (400 MHz, $CDCl_3$): δ 7.46 – 7.42 (m, 1H, Ar-*H*), 7.40 – 7.35 (m, 2H, Ar-*H*), 7.17 – 7.12 (m, 1H, Ar-*H*), 6.70 (dt, J = 15.5, 7.0 Hz, 1H, CO-CH=C*H*), 6.45 (dt, J = 15.5, 1.5 Hz, 1H, CO-C*H*=CH), 3.69 (br. s, 4H, $N(CH_2)_2$), 2.20 – 2.13 (m, 2H, CH=CH-CH_2), 1.44 – 1.36 (m, 2H, CH=CH-CH_2-CH_2), 1.34 – 1.09 (m, 12H, 3 × CH_2 & 2 × CH_3), 0.86 (t, J = 7.0 Hz, 3H, CH_3); **^{13}C NMR** (101 MHz, $CDCl_3$): δ 196.9, 149.4, 147.5, 134.7, 131.7, 130.8, 128.6, 124.9, 118.9, 49.0, 41.6, 32.6, 31.7, 29.0, 28.2, 22.6, 14.6, 14.1, 11.3; **Ir** ν_{max} (film)/cm^{-1} 2927, 2861, 1662, 1618, 1402, 1337, 1241, 1087, 977, 761; **HRMS** (ESI$^+$) calculated for $C_{19}H_{30}N_3O$ [M+H]$^+$: 316.2383, found: 316.2381.

(*E*)-1-{2-[(*E*)-Piperidin-1-yldiazenyl]phenyl}non-2-en-1-one (3a)

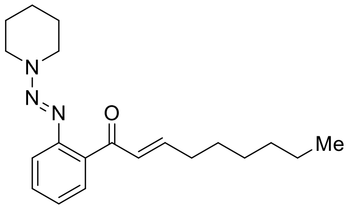

Prepared according to **General Procedure C**, using aldehyde **1a** (43 mg, 0.2 mmol), 1-octyne (44 μL, 0.3 mmol, 1.5 equiv), Rh(nbd)$_2$BF$_4$ (3.7 mg, 0.01 mmol, 5 mol%), dppe (4.0 mg, 0.01 mmol, 5 mol%) and CH_2Cl_2. Purification by flash column chromatography (gradient petrol/Et_2O, 95:5 to 90:10) afforded the title compound (61 mg, 93% yield) as a yellow oil. **^{1}H NMR** (400 MHz, $CDCl_3$): δ 7.48 – 7.44 (m, 1H, Ar-*H*), 7.43 – 7.36 (m, 2H, Ar-*H*), 7.18 (td, *J* = 7.5, 1.0 Hz, 1H, Ar-*H*), 6.70 (dt, *J* = 15.5, 7.0 Hz, 1H, CO-CH=C*H*), 6.45 (dt, *J* = 15.5, 1.5 Hz, 1H, CO-C*H*=CH), 3.74 (br. s, 4H, N(C*H*$_2$)$_2$), 2.21 – 2.14 (m, 2H, CH=CH-C*H*$_2$), 1.67 (br. s, 6H, 3 × C*H*$_2$), 1.46 – 1.37 (m, 2H, CH=CH-CH$_2$-C*H*$_2$), 1.34 – 1.20 (m, 6H, 3 × C*H*$_2$), 0.87 (t, *J* = 7.0 Hz, 3H, C*H*$_3$); **^{13}C NMR** (101 MHz, $CDCl_3$): δ 196.6, 149.0, 147.5, 134.8, 131.8, 130.9, 128.7, 125.3, 118.7, 52.4, 44.4, 32.6, 31.7, 29.0, 28.3, 25.5 (2C), 24.4, 22.6, 14.1; **IR** ν_{max} (film)/cm^{-1} 2925, 2855, 1657, 1618, 1418, 1355, 1293, 1182, 1096, 970, 852, 758; **HRMS** (ESI$^+$) calculated for $C_{20}H_{30}N_3O$ [M+H]$^+$: 328.2383, found: 328.2379.

(*E*)-4,4-Dimethyl-1-{2-[(*E*)-piperidin-1-yldiazenyl]phenyl}pent-2-en-1-one (3b)

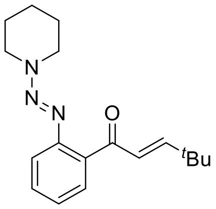

Prepared according to **General Procedure C**, using aldehyde **1a** (43 mg, 0.2 mmol), 3,3-dimethyl-1-butyne (37 μL, 0.3 mmol, 1.5 equiv), Rh(nbd)$_2$BF$_4$ (3.7 mg, 0.01 mmol, 5 mol%), dppe (4.0 mg, 0.01 mmol, 5 mol%) and CH_2Cl_2. Purification by flash column chromatography (petrol/Et_2O, 9:1) afforded the title compound (58 mg, 97% yield) as a colorless oil. **^{1}H NMR** (400 MHz, $CDCl_3$): δ 7.48 – 7.36 (m, 3H, Ar-*H*), 7.17 (td, *J* = 7.5, 1.0 Hz, 1H, Ar-*H*), 6.72 (d, *J* = 16.0 Hz, 1H, CO-CH=C*H*), 6.39 (d, *J* = 16.0 Hz, 1H, CO-C*H*=CH), 3.73 (br. s, 4H, N(C*H*$_2$)$_2$), 1.66 (br. s, 6H, 3 × C*H*$_2$), 1.04 (s, 9H, C(C*H*$_3$)$_3$); **^{13}C NMR** (101 MHz, $CDCl_3$): δ 197.0, 156.4, 149.0, 135.0, 131.0, 128.8, 126.8, 125.4, 118.8, 52.3, 44.1, 33.8, 28.9, 25.4 (2C), 24.3; **IR** ν_{max} (film)/cm^{-1} 2957, 2860, 1656, 1614, 1421, 1357, 1297, 1182, 1096, 1018, 982, 852, 758; **HRMS** (ESI$^+$) calculated for $C_{18}H_{26}N_3O$ [M+H]$^+$: 300.2070, found: 300.2068.

(*E*)-3-Phenyl-1-{2-[(*E*)-piperidin-1-yldiazenyl]phenyl}prop-2-en-1-one (3c)

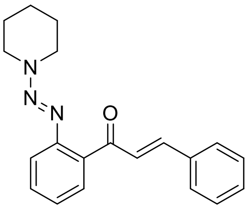

Prepared according to **General Procedure C**, using aldehyde **1a** (43 mg, 0.2 mmol), phenylacetylene (33 µL, 0.3 mmol, 1.5 equiv), $Rh(nbd)_2BF_4$ (3.7 mg, 0.01 mmol, 5 mol%), dppe (4.0 mg, 0.01 mmol, 5 mol%) and CH_2Cl_2. Purification by flash column chromatography (petrol/Et_2O, 4:1) afforded the title compound (57 mg, 89% yield) as a pale yellow solid. **m.p.** (Et_2O): 86 – 88 ºC; **^{1}H NMR** (400 MHz, $CDCl_3$): δ 7.57 (dd, J = 7.5, 1.5 Hz, 1H, Ar-*H*), 7.54 – 7.42 (m, 5H, 4 × Ar-*H* and CO-CH=C*H*), 7.40 – 7.32 (m, 3H, Ar-*H*), 7.22 (td, J = 7.5, 1.5 Hz, 1H, Ar-*H*), 7.15 (d, J = 16.0 Hz, 1H, CO-C*H*=CH), 3.74 – 3.66 (m, 4H, N(CH_2)$_2$), 1.55 (br. s, 6H, 3 × CH_2); **^{13}C NMR** (101 MHz, $CDCl_3$): δ 195.6, 149.4, 141.1, 135.3, 134.6, 131.5, 130.1, 129.1, 128.9, 128.5, 128.2, 125.4, 118.8, 52.8, 44.1, 26.2, 24.4, 24.2; **IR** ν_{max} (film)/cm^{-1} 2940, 2856, 1664, 1600, 1415, 1354, 1329, 1295, 1182, 1095, 1017, 974, 759, 701; **HRMS** (ESI$^+$) calculated for $C_{20}H_{22}N_3O$ [M+H]$^+$: 320.1757, found: 320.1757.

Enone **3c** was also prepared in **3.0 mmol scale**, using aldehyde **1a** (652 mg), phenylacetylene (0.49 mL), $Rh(nbd)_2BF_4$ (56 mg), dppe (60 mg) and acetone (3 mL). The title compound was obtained in 81% yield (775 mg).

V. Traceless Hydroacylation

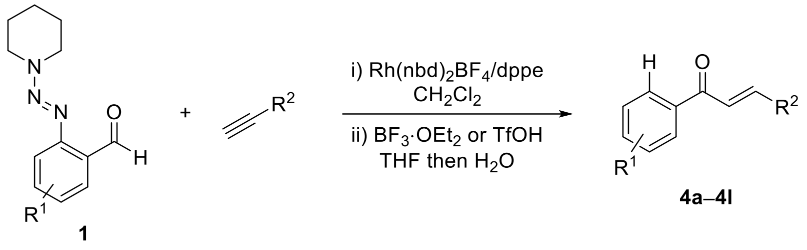

General Procedure D: A reaction vial charged with $Rh(nbd)_2BF_4$ (3.7 mg, 0.01 mmol, 5 mol%) and dppe (4.0 mg, 0.01 mmol, 5 mol%) was evacuated and back-filled with N_2 gas for 3 times, and to this was added CH_2Cl_2 (0.25 mL). The resulting solution was bubbled with H_2 gas for 2 min and then with N_2 gas until the volume of the solution had become around 0.1 mL. This solution was then transferred to a N_2-purged reaction vial containing aldehyde (0.2 mmol, 1.0 equiv), with the use of additional 0.1 mL of CH_2Cl_2. Alkyne (0.30 mmol, 1.5 equiv) was added and the reaction mixture was stirred at 23 ºC for 16 h. The solution was diluted with Et_2O, filtered through a pad of silica to a 25 mL round-bottom flask and concentrated under reduced pressure. The crude was then dissolved in THF (4 mL, 0.05 M) and $BF_3{\cdot}OEt_2$ (1.0 or 2.0 mmol, 5.0 or 10.0 equiv) or triflic acid (0.68 mmol, 3.4 equiv) was added at 0 ºC. The reaction was stirred at 23 ºC until aryltriazenyl enone was all consumed (5-60 min). H_2O (50 µL) was then added and the mixture was further stirred at 23 ºC for 24 h. After completion, the reaction was concentrated under reduced pressure and purified by flash column chromatography to afford the corresponding enone product.

(*E*)-1-Phenylnon-2-en-1-one (4a)

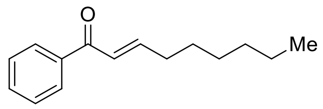

Prepared according to a modified **General Procedure D** on a 0.15 mmol scale, using aldehyde **1a** (33 mg, 0.15 mmol), 1-octyne (33 µL) and $BF_3{\cdot}OEt_2$ (0.19 mL). Purification by flash column chromatography (petrol/Et_2O, 95:5) afforded the title compound (28 mg, 86% yield) as a colorless oil. **^{1}H NMR** (400 MHz, $CDCl_3$): δ 7.95 – 7.90 (m, 2H, Ar-*H*), 7.58 – 7.52 (m, 1H, Ar-*H*), 7.50 – 7.43 (m, 2H, Ar-*H*), 7.07 (dt, J = 15.5, 7.0 Hz, 1H, CO-CH=C*H*), 6.87 (dt, J = 15.5, 1.5 Hz, 1H, CO-C*H*=CH), 2.36 – 2.28 (m, 2H, CH=CH-CH_2), 1.57 – 1.48 (m, 2H, CH=CH-CH_2-CH_2), 1.40 – 1.24 (m, 6H, 3 × CH_2), 0.89 (t, J = 7.0 Hz, 3H, CH_3); **^{13}C NMR** (101 MHz, $CDCl_3$): δ 191.2, 150.3, 138.2, 132.7, 128.7, 128.6, 126.0, 33.0, 31.8, 29.1, 28.3, 22.7, 14.2; **IR** ν_{max} (film)/cm^{-1} 2927, 2856, 1671, 1621, 1448, 1286, 1225, 972, 693; data consistent with those reported in literature.[4]

(*E*)-4,4-Dimethyl-1-phenylpent-2-en-1-one (4b)

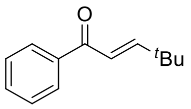

Prepared according to **General Procedure D**, using aldehyde **1a** (43 mg), 3,3-dimethyl-1-butyne (37 μL) and $BF_3·OEt_2$ (0.12 mL). Purification by flash column chromatography (petrol/Et_2O, 95:5) afforded the title compound (34 mg, 91% yield) as a yellow oil. **^{1}H NMR** (400 MHz, $CDCl_3$): δ 7.95 – 7.90 (m, 2H, Ar-*H*), 7.58 – 7.52 (m, 1H, Ar-*H*), 7.50 – 7.43 (m, 2H, Ar-*H*), 7.06 (d, *J* = 15.5 Hz, 1H, CO-CH=C*H*), 6.78 (d, *J* = 15.5 Hz, 1H, CO-C*H*=CH), 1.15 (s, 9H, C(CH_3)$_3$); **^{13}C NMR** (101 MHz, $CDCl_3$): δ 191.7, 159.8, 138.3, 132.7, 128.7, 128.6, 121.1, 34.3, 28.9; **IR** ν_{max} (film)/cm^{-1} 2961, 2868, 1668, 1618, 1448, 1301, 1224, 1018, 985, 841, 776, 696, 656; data consistent with those reported in literature.[5]

(*E*)-Chalcone (4c)

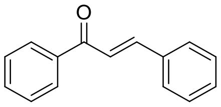

Prepared according to **General Procedure D**, using aldehyde **1a** (43 mg), phenylacetylene (33 μL) and $BF_3·OEt_2$ (0.25 mL). Purification by flash column chromatography (petrol/Et_2O, 95:5) afforded the title compound (35 mg, 83% yield) as a pale yellow solid. **m.p.** (petrol/Et_2O): 55 – 57 ºC; **^{1}H NMR** (400 MHz, $CDCl_3$): δ 8.05 – 8.01 (m, 2H, Ar-*H*), 7.82 (d, *J* = 15.5 Hz, 1H, CO-CH=C*H*), 7.68 – 7.63 (m, 2H, Ar-*H*), 7.62 – 7.57 (m, 1H, Ar-*H*), 7.57 – 7.48 (m, 3H, 2 × Ar-*H* and CO-C*H*=CH), 7.45 – 7.40 (m, 3H, Ar-*H*); **^{13}C NMR** (101 MHz, $CDCl_3$): δ 190.7, 145.0, 138.3, 135.0, 132.9, 130.7, 129.1, 128.8, 128.64, 128.58, 122.2; **IR** ν_{max} (film)/cm^{-1} 3059, 3027, 1662, 1604, 1575, 1449, 1335, 1214, 1016, 978, 745, 688; data consistent with those reported in literature.[6]

(*E*)-3-Cyclopropyl-1-phenylprop-2-en-1-one (4d)

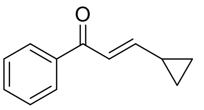

Prepared according to **General Procedure D**, using aldehyde **1a** (43 mg), ethynylcyclopropane (25 μL) and $BF_3·OEt_2$ (0.12 mL). Purification by flash column chromatography (petrol/Et_2O, 9:1) afforded the title compound (22 mg, 64% yield) as a colorless oil. **^{1}H NMR** (400 MHz, $CDCl_3$): δ 7.96 – 7.90 (m, 2H, Ar-*H*), 7.58 – 7.51 (m, 1H, Ar-*H*), 7.50 – 7.43 (m, 2H, Ar-*H*), 7.02 (d, *J* = 15.0 Hz, 1H, CO-C*H*=CH), 6.56 (dd, *J* = 15.0, 10.5 Hz, 1H, CO-CH=C*H*), 1.76 – 1.66 (m, 1H, CH=CH-C*H*), 1.06 – 0.99 (m, 2H, CH_aH$_b$-CH_cH$_d$), 0.77 – 0.71 (m, 2H, CH$_a$$H_b$-CH$_c$$H_d$); **^{13}C NMR** (101 MHz, $CDCl_3$): δ 190.1, 155.4, 138.3, 132.6, 128.6, 128.5, 123.0, 15.5, 9.4; **IR** ν_{max} (film)/cm^{-1} 2980, 2889, 1663, 1607, 1381,

1270, 1155, 1073, 938, 890, 696; **HRMS** (ESI^+) calculated for $C_{12}H_{13}O$ $[M+H]^+$: 173.0961, found: 173.0962; data consistent with those reported in literature.[7]

(*E*)-3-Cyclopentyl-1-phenylprop-2-en-1-one (4e)

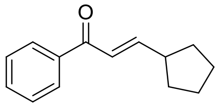

Prepared according to **General Procedure D**, using aldehyde **1a** (43 mg), ethynylcyclopentane (35 μL) and $BF_3 \cdot OEt_2$ (0.12 mL). Purification by flash column chromatography (petrol/CH_2Cl_2, 1:1) afforded the title compound (34 mg, 85% yield) as a yellow oil. **^{1}H NMR** (400 MHz, $CDCl_3$): δ 7.95 – 7.90 (m, 2H, Ar-*H*), 7.58 – 7.52 (m, 1H, Ar-*H*), 7.50 – 7.43 (m, 2H, Ar-*H*), 7.05 (dd, *J* = 15.5, 8.0 Hz, 1H, CO-CH=C*H*), 6.86 (d, *J* = 15.5 Hz, 1H, CO-C*H*=CH), 2.77 – 2.65 (m, 1H, CH=CH-C*H*), 1.94 – 1.85 (m, 2H, Cypent-*H*), 1.79 – 1.58 (m, 4H, Cypent-*H*), 1.53 – 1.42 (m, 2H, Cypent-*H*); **^{13}C NMR** (101 MHz, $CDCl_3$): δ 191.3, 154.4, 138.3, 132.7, 128.64, 128.62, 124.1, 43.6, 32.8, 25.5; **IR** ν_{max} (film)/cm^{-1} 2980, 2869, 1668, 1617, 1448, 1363, 1261, 1218, 1011, 984, 772, 697; data consistent with those reported in literature.[8]

(*E*)-3-(Cyclohex-1-en-1-yl)-1-phenylprop-2-en-1-one (4f)

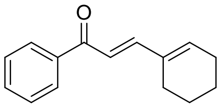

Prepared according to **General Procedure D**, using aldehyde **1a** (43 mg), 1-ethynylcyclohex-1-ene (35 μL) and $BF_3 \cdot OEt_2$ (0.12 mL). Purification by flash column chromatography (petrol/CH_2Cl_2, 1:1) afforded the title compound (34 mg, 80% yield) as a pale yellow solid. **m.p.** (petrol/Et_2O): 70 – 71 ºC; **^{1}H NMR** (400 MHz, $CDCl_3$): δ 7.96 – 7.92 (m, 2H, Ar-*H*), 7.57 – 7.51 (m, 1H, Ar-*H*), 7.49 – 7.38 (m, 3H, 2 × Ar-*H* and CO-CH=C*H*), 6.84 (d, *J* = 15.5 Hz, 1H, CO-C*H*=CH), 6.29 (t, *J* = 3.5 Hz, 1H, C=C*H*), 2.30 – 2.21 (m, 4H, CH_2-C=CH-CH_2), 1.78 – 1.60 (m, 4H, 2 × CH_2); **^{13}C NMR** (101 MHz, $CDCl_3$): δ 191.4, 148.6, 140.9, 138.7, 135.7, 132.5, 128.6, 128.5, 118.9, 26.8, 24.5, 22.2, 22.1; **IR** ν_{max} (film)/cm^{-1} 2981, 2930, 1660, 1586, 1447, 1291, 1210, 1017, 980, 763, 698, 623; data consistent with those reported in literature.[9]

(*E*)-1,5-Diphenylpent-2-en-1-one (4g)

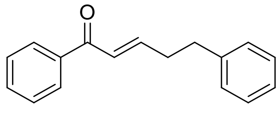

Prepared according to **General Procedure D**, using aldehyde **1a** (43 mg), but-3-yn-1-ylbenzene (42 µL) and $BF_3{\cdot}OEt_2$ (0.12 mL). Purification by flash column chromatography (petrol/CH_2Cl_2, 1:1) afforded the title compound (33 mg, 70% yield) as a yellow oil. **^{1}H NMR** (400 MHz, $CDCl_3$): δ 7.91 – 7.87 (m, 2H, Ar-*H*), 7.59 – 7.53 (m, 1H, Ar-*H*), 7.49 – 7.43 (m, 2H, Ar-*H*), 7.35 – 7.29 (m, 2H, Ar-*H*), 7.25 – 7.19 (m, 3H, Ar-*H*), 7.09 (dt, *J* = 15.5, 7.0 Hz, 1H, CO-CH=C*H*), 6.88 (dt, *J* = 15.5, 1.5 Hz, 1H, CO-C*H*=CH), 2.89 – 2.83 (m, 2H, CH_2-Ph), 2.69 – 2.61 (m, 2H, CH=CH-CH_2); **^{13}C NMR** (101 MHz, $CDCl_3$): δ 191.0, 148.6, 140.9, 138.0, 132.8, 128.7, 128.6 (2C), 128.5, 126.7, 126.3, 34.7, 34.6; $\boldsymbol{\nu}_{\mathbf{max}}$ (film)/cm^{-1} 2981, 1669, 1619, 1448, 1283, 1224, 969, 695; data consistent with literature.[10]

(*E*)-3-Ferrocenyl-1-phenylprop-2-en-1-one (4h)

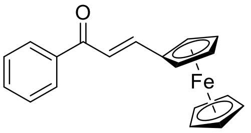

Prepared according to **General Procedure D**, using aldehyde **1a** (43 mg), ethynylferrocene (63 mg) and $BF_3{\cdot}OEt_2$ (0.12 mL). Purification by flash column chromatography (petrol/Et_2O, 4:1) afforded the title compound (33 mg, 52% yield) as a purple solid. **m.p.** (petrol/Et_2O): 135 – 136 ºC; **^{1}H NMR** (400 MHz, $CDCl_3$): δ 8.00 – 7.96 (m, 2H, Ar-*H*), 7.76 (d, *J* = 15.5 Hz, 1H, CO-CH=C*H*), 7.60 – 7.54 (m, 1H, Ar-*H*), 7.52-7.47 (m, 2H, Ar-*H*), 7.13 (d, *J* = 15.5 Hz, 1H, CO-C*H*=CH), 4.60 (t, *J* = 2.0 Hz, 2H, Fc-*H*), 4.49 (t, *J* = 2.0 Hz, 2H, Fc-*H*), 4.18 (s, 5H, Fc-*H*); **^{13}C NMR** (101 MHz, $CDCl_3$): δ 190.0, 147.0, 138.7, 132.5, 128.7, 128.5, 119.3, 79.3, 71.5, 69.9, 69.2; **IR** ν_{max} (film)/cm^{-1} 3657, 2980, 2889, 1655, 1587, 1473, 1462, 1382, 1251, 1152, 1073, 954, 818; **HRMS** (ESI$^+$) calculated for $C_{19}H_{17}FeO$ $[M+H]^+$: 317.0623, found: 317.0622.

(*E*)-1-(4-Chlorophenyl)-4,4-dimethylpent-2-en-1-one (4i)

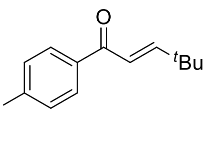

Prepared according to **General Procedure D**, using aldehyde **1c** (50 mg), 3,3-dimethyl-1-butyne (37 µL) and TfOH (0.06 mL). Purification by flash column chromatography (petrol/CH_2Cl_2, 3:2) afforded the title compound (32 mg, 72% yield) as a yellow oil. **^{1}H NMR** (400 MHz, $CDCl_3$): δ 7.86 (d, *J* = 8.5 Hz, 2H, Ar-*H*), 7.43 (d, *J* = 8.5 Hz, 2H, Ar-*H*), 7.06 (d, *J* = 15.5 Hz, 1H, CO-CH=C*H*), 6.73 (d, *J* =

15.5 Hz, 1H, CO-C*H*=CH), 1.15 (s, 9H, C(CH_3)$_3$); **^{13}C NMR** (101 MHz, $CDCl_3$): δ 190.3, 160.3, 139.1, 136.6, 130.1, 128.9, 120.6, 34.4, 28.8; data consistent with literature.[11]

(*E*)-3-Cyclopentyl-1-[4-(trifluoromethyl)phenyl]prop-2-en-1-one (4j)

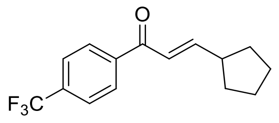

Prepared according to **General Procedure D**, using aldehyde **1d** (57 mg), ethynylcyclopentane (35 µL) and TfOH (0.06 mL). Purification by flash column chromatography (petrol/CH_2Cl_2, 7:3) afforded the title compound (32 mg, 59% yield) as a yellow oil. **^{1}H NMR** (400 MHz, $CDCl_3$): δ 7.99 (d, J = 8.0 Hz, 2H, Ar-*H*), 7.72 (d, J = 8.0 Hz, 2H, Ar-*H*), 7.07 (dd, J = 15.5, 8.0 Hz, 1H, CO-CH=C*H*), 6.81 (d, J = 15.5 Hz, 1H, CO-C*H*=CH), 2.78 – 2.66 (m, 1H, CH=CH-C*H*), 1.96 – 1.86 (m, 2H, Cypent-*H*), 1.79 – 1.59 (m, 4H, Cypent-*H*), 1.53 – 1.42 (m, 2H, Cypent-*H*); **^{13}C NMR** (101 MHz, $CDCl_3$): δ 190.4, 156.0, 141.1, 133.9 (q, $J_{C\text{-}F}$ = 32.5 Hz), 128.9, 125.7 (q, $J_{C\text{-}F}$ = 3.5 Hz), 123.84, 123.82 (q, $J_{C\text{-}F}$ = 272.5 Hz), 43.7, 32.8, 25.5; **^{19}F NMR** (377 MHz, $CDCl_3$): δ –63.0; **IR** ν_{max} (film)/cm^{-1} 2956, 2871, 1672, 1613, 1319, 1167, 1127, 1066, 1013, 830, 689; **HRMS** (ESI$^+$) calculated for $C_{15}H_{16}F_3O$ [M+H]$^+$: 269.1148, found: 269.1149.

(*E*)-3-Cyclopentyl-1-[3-(trifluoromethoxy)phenyl]prop-2-en-1-one (4k)

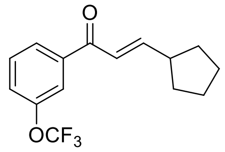

Prepared according to **General Procedure D**, using aldehyde **1g** (60 mg), ethynylcyclopentane (35 µL) and TfOH (0.06 mL). Purification by flash column chromatography (petrol/CH_2Cl_2, 3:1) afforded the title compound (34 mg, 60% yield) as a yellow oil. **^{1}H NMR** (400 MHz, $CDCl_3$): δ 7.84 (d, J = 8.0 Hz, 1H, Ar-*H*), 7.75 (s, 1H, Ar-*H*), 7.50 (t, J = 8.0 Hz, 1H, Ar-*H*), 7.39 (d, J = 8.0 Hz, 1H, Ar-*H*), 7.07 (dd, J = 15.5, 8.0 Hz, 1H, CO-CH=C*H*), 6.80 (d, J = 15.5 Hz, 1H, CO-C*H*=CH), 2.78 – 2.66 (m, 1H, CH=CH-C*H*), 1.95 – 1.85 (m, 2H, Cypent-*H*), 1.79 – 1.59 (m, 4H, Cypent-*H*), 1.53 – 1.41 (m, 2H, Cypent-*H*); **^{13}C NMR** (101 MHz, $CDCl_3$): δ 189.6, 155.7, 149.5, 140.1, 130.2, 126.9, 125.0, 123.6, 121.1, 120.6 (q, $J_{C\text{-}F}$ = 258.0 Hz), 43.7, 32.8, 25.5; **^{19}F NMR** (377 MHz, $CDCl_3$): δ –57.9; **IR** ν_{max} (film)/cm^{-1} 2957, 2871, 1672, 1619, 1585, 1442, 1251, 1212, 1160, 984; **HRMS** (ESI$^+$) calculated for $C_{15}H_{16}F_3O_2$ [M+H]$^+$: 285.1097, found: 285.1098.

(*E*)-3-Cyclopentyl-1-(3-fluorophenyl)prop-2-en-1-one (4l)

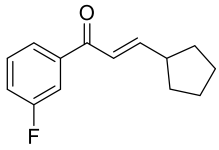

Prepared according to **General Procedure D**, using aldehyde **1h** (47 mg), ethynylcyclopentane (35 μL) and TfOH (0.06 mL). Purification by flash column chromatography (petrol/CH_2Cl_2, 7:3) afforded the title compound (27 mg, 62% yield) as a yellow oil. **^{1}H NMR** (400 MHz, $CDCl_3$): δ 7.70 (d, J = 8.0 Hz, 1H, Ar-*H*), 7.61 (d, J = 9.5 Hz, 1H, Ar-*H*), 7.44 (td, J = 8.0, 5.5 Hz, 1H, Ar-*H*), 7.28 – 7.21 (m, 1H, Ar-*H*), 7.07 (dd, J = 15.5, 8.0 Hz, 1H, CO-CH=C*H*), 6.81 (d, J = 15.5 Hz, 1H, CO-C*H*=CH), 2.77 – 2.65 (m, 1H, CH=CH-C*H*), 1.95 – 1.85 (m, 2H, Cypent-*H*), 1.79 – 1.59 (m, 4H, Cypent-*H*), 1.53 – 1.41 (m, 2H, Cypent-*H*); **^{13}C NMR** (101 MHz, $CDCl_3$): δ 189.8, 162.9 (d, $J_{C\text{-}F}$ = 247.5 Hz), 155.3, 140.4 (d, $J_{C\text{-}F}$ = 6.5 Hz), 130.3 (d, $J_{C\text{-}F}$ = 7.5 Hz), 124.3 (d, $J_{C\text{-}F}$ = 3.0 Hz), 123.6, 119.7 (d, $J_{C\text{-}F}$ = 21.5 Hz), 115.4 (d, $J_{C\text{-}F}$ = 22.5 Hz), 43.6, 32.7, 25.5; **^{19}F NMR** (377 MHz, $CDCl_3$): δ –112.0; **IR** ν_{max} (film)/cm^{-1} 2954, 2869, 1670, 1620, 1586, 1484, 1441, 1361, 1300, 1265, 1151, 983, 894, 791, 723, 673; **HRMS** (ESI$^+$) calculated for $C_{14}H_{16}FO$ [M+H]$^+$: 219.1180, found: 219.1182.

VI. Hydroacylation/*ortho*-C–H Alkenylation/Proto-detriazenation

1. Sequential Hydroacylation/*ortho*-C–H Alkenylation

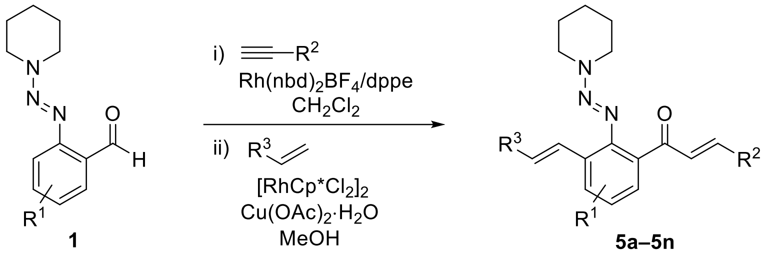

General Procedure E: A reaction vial charged with $Rh(nbd)_2BF_4$ (5.6 mg, 0.015 mmol, 5 mol%) and dppe (6.0 mg, 0.015 mmol, 5 mol%) was evacuated and back-filled with N_2 gas for 3 times, and to this was added CH_2Cl_2 (0.3 mL). The resulting solution was bubbled with H_2 gas for 2 min and then with N_2 gas until the volume of the solution had become around 0.1 mL. This solution was then transferred to a N_2-purged reaction vial containing aldehyde (0.3 mmol, 1.0 equiv), with the use of additional 0.2 mL of CH_2 CH_2Cl_2. Alkyne (0.45 mmol, 1.5 equiv) was added and the reaction mixture was stirred at 23 ºC for 16 h. The mixture was diluted with Et_2O, filtered through a pad of silica to a reaction vial, and concentrated under reduced pressure. To this was added MeOH (2.0 mL, 0.15 M), and the resulting solution was transferred to a reaction vial containing $[RhCp^*Cl_2]_2$ (9.3 mg, 0.015 mmol, 5 mol%) and $Cu(OAc)_2·H_2O$ (120 mg, 0.60 mmol, 2.0 equiv), followed by the addition of alkene (0.75 mmol, 2.5 equiv). The reaction mixture was stirred at 70 ºC. Upon completion (6-16 h), it was cooled to room temperature, diluted with Et_2O, filtered through a pad of silica and concentrated under reduced pressure. Purification by flash column chromatography afforded the corresponding product.

(*E*)-Butyl 3-{3-[(*E*)-4,4-dimethylpent-2-enoyl]-2-[(*E*)-piperidin-1-yldiazenyl]phenyl}acrylate (5a)

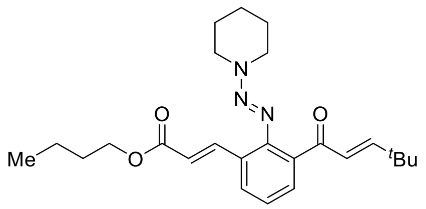

Prepared according to **General Procedure E**, using aldehyde **1a** (65 mg), 3,3-dimethylbut-1-yne (55 μL) and *n*-butyl acrylate (108 μL). Purification by flash column chromatography (petrol/Et_2O, 4:1) afforded the title compound (104 mg, 82% yield) as a yellow oil. **^{1}H NMR** (400 MHz, $CDCl_3$): δ 8.32 (d, J = 16.0 Hz, 1H, CH=C*H*-Ar), 7.67 (dd, J = 8.0, 1.0 Hz, 1H, Ar-*H*), 7.36 (dd, J = 7.5, 1.5 Hz, 1H, Ar-*H*), 7.18 (t, J = 7.5 Hz, 1H, Ar-*H*), 6.61 (d, J = 16.0 Hz, 1H, CO-CH=C*H*), 6.43 (d, J = 16.0 Hz, 1H, C*H*=CH-Ar), 5.90 (d, J = 16.0 Hz, 1H, CO-C*H*=CH), 4.18 (t, J = 6.5 Hz, 2H, CO_2CH_2), 3.90 – 3.60 (2

× br. s, 4H, N(CH_2)$_2$), 1.70 – 1.62 (m, 8H, 4 × CH_2), 1.47 – 1.37 (m, 2H, CH_2-CH_3), 0.99 (s, 9H, C(CH_3)$_3$), 0.93 (t, J = 7.5 Hz, 3H, CH_2-CH_3); **^{13}C NMR** (101 MHz, $CDCl_3$): δ 196.1, 167.3, 155.9, 148.2, 141.3, 133.7, 130.9, 129.1, 128.6, 126.1, 125.1, 118.9, 64.3, 52.6, 44.3, 33.7, 30.8, 28.8, 26.4, 24.3, 24.1, 19.3, 13.8; **IR** ν_{max} (film)/cm^{-1} 2957, 2864, 1710, 1676, 1656, 1622, 1413, 1356, 1315, 1255, 1165, 1108, 1059, 985, 755, 729; **HRMS** (ESI$^+$) calculated for $C_{25}H_{36}N_3O_3$ [M+H]$^+$: 426.2751, found: 426.2744.

(*E*)-Butyl 3-{3-[(*E*)-non-2-enoyl]-2-[(*E*)-piperidin-1-yldiazenyl]phenyl}acrylate (5b)

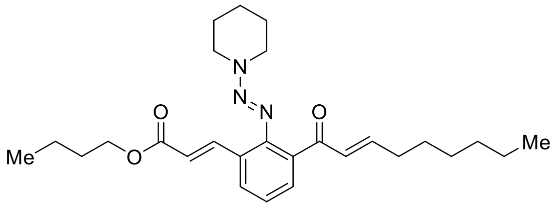

Prepared according to **General Procedure E**, using aldehyde **1a** (65 mg), 1-octyne (66 µL) and *n*-butyl acrylate (108 µL). Purification by flash column chromatography (gradient petrol/Et_2O, 8:2 to 7:3) afforded the title compound (101 mg, 74% yield) as a red oil. **^{1}H NMR** (400 MHz, $CDCl_3$): δ 8.31 (d, J = 16.0 Hz, 1H, CH=CH-Ar), 7.66 (dd, J = 8.0, 1.0 Hz, 1H, Ar-H), 7.34 (dd, J = 7.5, 1.5 Hz, 1H, Ar-H), 7.17 (t, J = 7.5 Hz, 1H, Ar-H), 6.60 (dt, J = 15.5, 7.0 Hz, 1H, CO-CH=CH), 6.42 (d, J = 16.0 Hz, 1H, CH=CH-Ar), 6.01 (dt, J = 15.5, 1.5 Hz, 1H, CO-CH=CH), 4.18 (t, J = 6.5 Hz, 2H, CO_2CH_2), 3.90 – 3.60 (2 × br. s, 4H, N(CH_2)$_2$), 2.14 – 2.06 (m, 2H, CH=CH-CH_2), 1.72 – 1.61 (m, 8H, 4 × CH_2), 1.47 – 1.19 (m, 10H, 5 × CH_2), 0.93 (t, J = 7.5 Hz, 3H, CH_3), 0.84 (t, J = 7.0 Hz, 3H, CH_3); **^{13}C NMR** (101 MHz, $CDCl_3$): δ 195.8, 167.3, 148.2, 146.9, 141.3, 133.5, 131.0, 130.8, 129.1, 128.5, 125.0, 118.9, 64.3, 52.7, 44.0, 32.4, 31.6, 30.8, 28.9, 28.2, 26.5, 24.5, 24.2, 22.5, 19.3, 14.1, 13.8; **IR** ν_{max} (film)/cm^{-1} 2980, 2889, 1710, 1675, 1627, 1463, 1382, 1314, 1254, 1164, 1109, 1071, 968, 754, 728; **HRMS** (ESI$^+$) calculated for $C_{27}H_{40}N_3O_3$ [M+H]$^+$: 454.3064, found: 454.3059.

(*E*)-Butyl 3-{3-[(*E*)-6-chlorohex-2-enoyl]-2-[(*E*)-piperidin-1-yldiazenyl]phenyl}acrylate (5c)

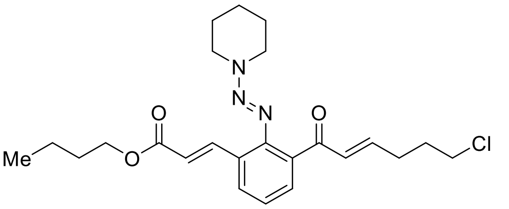

Prepared according to **General Procedure E**, using aldehyde **1a** (65 mg), 5-chloropent-1-yne (48 µL) and *n*-butyl acrylate (108 µL). Purification by flash column chromatography (petrol/Et_2O, 7:3) afforded the title compound (97 mg, 73% yield) as a brown oil. **^{1}H NMR** (400 MHz, $CDCl_3$): δ 8.30 (d, J = 16.0 Hz, 1H, CH=C*H*-Ar), 7.67 (dd, J = 7.5, 1.0 Hz, 1H, Ar-*H*), 7.34 (dd, J = 7.5, 1.5 Hz, 1H, Ar-*H*), 7.18 (t, J = 7.5 Hz, 1H, Ar-*H*), 6.54 (dt, J = 15.5, 7.0 Hz, 1H, CO-CH=C*H*), 6.42 (d, J = 16.0 Hz, 1H, C*H*=CH-Ar), 6.05 (dt, J = 15.5, 1.5 Hz, 1H, CO-C*H*=CH), 4.17 (t, J = 6.5 Hz, 2H, CO_2CH_2), 3.92 – 3.60 (2 × br. s, 4H, $N(CH_2)_2$), 3.47 (t, J = 6.5 Hz, 2H, CH_2-Cl), 2.32 – 2.25 (m, 2H, CH=CH-CH_2), 1.88 – 1.80 (m, 2H, CH_2-CH_2-Cl), 1.72 – 1.61 (m, 8H, 4 × CH_2), 1.47 – 1.36 (m, 2H, CH_2-CH_3), 0.93 (t, J = 7.5 Hz, 3H, CH_3); **^{13}C NMR** (101 MHz, $CDCl_3$): δ 195.2, 167.2, 148.2, 143.9, 141.2, 133.2, 132.1, 130.8, 129.1, 128.7, 125.1, 119.0, 64.3, 52.7, 44.2, 44.0, 30.81, 30.80, 29.2, 26.5, 24.4, 24.1, 19.3, 13.8; **IR** ν_{max} (film)/cm^{-1} 2939, 2860, 1708, 1676, 1627, 1574, 1411, 1355, 1313, 1256, 1167, 1108, 984, 755, 727; **HRMS** (ESI$^+$) calculated for $C_{24}H_{33}ClN_3O_3$ $[M+H]^+$: 446.2205, found: 446.2209.

(*E*)-Butyl 3-{3-cinnamoyl-2-[(*E*)-piperidin-1-yldiazenyl]phenyl}acrylate (5d)

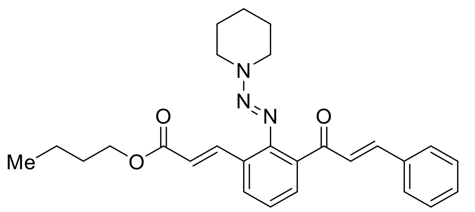

Prepared according to **General Procedure E**, using aldehyde **1a** (65 mg), phenylacetylene (49 µL) and *n*-butyl acrylate (108 µL). Purification by flash column chromatography (petrol/Et_2O, 7:3) afforded the title compound (108 mg, 81% yield) as a red oil. **^{1}H NMR** (400 MHz, $CDCl_3$): δ 8.33 (d, J = 16.0 Hz, 1H, CH=C*H*-Ar), 7.71 (dd, J = 8.0, 1.0 Hz, 1H, Ar-*H*), 7.49 (dd, J = 7.5, 1.5 Hz, 1H, Ar-*H*), 7.47 – 7.41 (m, 3H, CO-CH=C*H* and 2 × Ar-*H*), 7.35 – 7.30 (m, 3H, Ar-*H*), 7.22 (t, J = 7.5 Hz, 1H, Ar-*H*), 6.59 (d, J = 16.0 Hz, 1H, CO-C*H*=CH), 6.47 (d, J = 16.0 Hz, 1H, C*H*=CH-Ar), 4.19 (t, J = 6.5 Hz, 2H, CO_2CH_2), 3.86 – 3.56 (2 × br. s, 4H, $N(CH_2)_2$), 1.71 – 1.63 (m, 2H, CH_2), 1.53 – 1.38 (m, 8H, 4 × CH_2), 0.94 (t, J = 7.5 Hz, 3H, CH_3); **^{13}C NMR** (101 MHz, $CDCl_3$): δ 194.6, 167.2, 148.7, 141.2, 140.9, 135.0, 133.8, 131.1, 130.1, 129.1, 129.0, 128.9, 127.9, 127.7, 125.1, 119.0, 64.3, 52.6, 44.0, 30.8, 26.3, 24.2, 23.9, 19.3, 13.8; **IR** ν_{max} (film)/cm^{-1} 2980, 2889, 1707, 1669, 1631, 1607, 1575, 1462, 1383, 1315, 1253, 1165, 1110, 1071, 954, 762, 729, 699; **HRMS** (ESI$^+$) calculated for $C_{27}H_{32}N_3O_3$ $[M+H]^+$: 446.2438, found: 446.2433.

(*E*)-Butyl 3-{2-[(*E*)-piperidin-1-yldiazenyl]-3-[(*E*)-3-(thiophen-3-yl)acryloyl]phenyl}acrylate (5e)

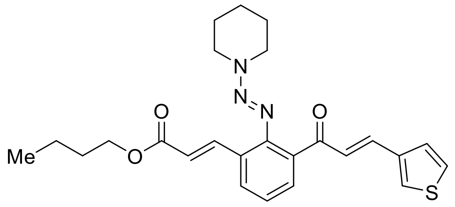

Prepared according to **General Procedure E**, using aldehyde **1a** (65 mg), 3-ethynylthiophene (44 μL) and *n*-butyl acrylate (108 μL). Purification by flash column chromatography (petrol/Et_2O, 7:3) afforded the title compound (76 mg, 56% yield) as a brown oil. **^{1}H NMR** (400 MHz, $CDCl_3$): δ 8.33 (d, J = 16.0 Hz, 1H, CH=C*H*-Ar), 7.72 (d, J = 7.5 Hz, 1H, Ar-*H*), 7.48 (dd, J = 7.5, 1.0 Hz, 1H, Ar-*H*), 7.46 – 7.39 (m, 2H, CO-CH=C*H* and Ar-*H*), 7.33 – 7.29 (m, 1H, Ar-*H*), 7.27 – 7.20 (m, 2H, Ar-*H*), 6.50 – 6.40 (m, 2H, C*H*=CH-Ar and CO-C*H*=CH), 4.21 (t, J = 6.5 Hz, 2H, CO_2CH_2), 3.88 – 3.59 (2 × br. s, 4H, $N(CH_2)_2$), 1.73 – 1.64 (m, 2H, CH_2), 1.58 – 1.39 (m, 8H, 4 × CH_2), 0.96 (t, J = 7.5 Hz, 3H, CH_3); **^{13}C NMR** (101 MHz, $CDCl_3$): δ 195.1, 167.4, 148.8, 141.3, 138.2, 134.9, 133.8, 131.2, 129.2, 129.0, 128.0, 127.7, 127.1, 125.1, 125.0, 119.1, 64.4, 52.7, 44.1, 30.9, 26.4, 24.4, 24.1, 19.3, 13.9; **IR** ν_{max} (film)/cm^{-1} 2939, 2859, 1707, 1667, 1631, 1602, 1409, 1315, 1290, 1254, 1168, 1111, 1051, 984, 867, 781, 755; **HRMS** (ESI$^+$) calculated for $C_{25}H_{30}N_3O_3S$ [M+H]$^+$: 452.2002, found: 452.2001.

(*E*)-Butyl 3-{3-[(*E*)-4,4-dimethylpent-2-enoyl]-4-fluoro-2-[(*E*)-piperidin-1-yl diazenyl]pheny-l}acrylate (5f)

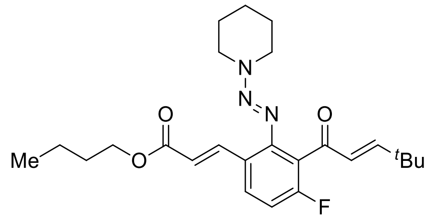

Prepared according to a modified **General Procedure E** on a **3.0 mmol scale**, using aldehyde **1k** (706 mg, 3.0 mmol), 3,3-dimethylbut-1-yne (0.55 mL) $Rh(nbd)_2BF_4$ (56 mg, 0.15 mmol, 5 mol%), dppe (60 mg, 0.15 mmol, 5 mol%), CH_2Cl_2 (3.0 mL), *n*-butyl acrylate (1.1 mL), $[RhCp^*Cl_2]_2$ (93 mg, 0.15 mmol, 5 mol%), $Cu(OAc)_2\cdot H_2O$ (1.20 g, 6.0 mmol, 2.0 equiv) and MeOH (20 mL). Purification by flash column chromatography (petrol/Et_2O, 85:15) afforded the title compound (1.18 g, 89% yield) as an off-white solid. **m.p.** (petrol): 92 – 94 ºC; **^{1}H NMR** (400 MHz, $CDCl_3$): δ 8.27 (d, J = 16.0 Hz, 1H, CH=C*H*-Ar), 7.61 (dd, J = 8.5, 6.0 Hz, 1H, Ar-*H*), 6.89 (t, J = 8.5 Hz, 1H, Ar-*H*), 6.54 (d, J = 16.0 Hz, 1H, CO-CH=C*H*), 6.36 (d, J = 16.0 Hz, 1H, C*H*=CH-Ar), 6.06 (d, J = 16.0 Hz, 1H, CO-C*H*=CH), 4.18 (t, J = 6.5 Hz, 2H, CO_2CH_2), 3.88 – 3.58 (2 × br. s, 4H, $N(CH_2)_2$), 1.71 – 1.61 (m, 8H, 4 × CH_2), 1.47 – 1.36 (m, 2H, CH_2-CH_3), 1.01 (s, 9H, $C(CH_3)_3$), 0.94 (t, J = 7.5 Hz, 3H, CH_2-CH_3); **^{13}C NMR** (101 MHz, $CDCl_3$): δ 192.8, 167.3, 160.8 (d, $J_{C\text{-}F}$ = 253.0 Hz), 158.1, 148.7 (d, $J_{C\text{-}F}$ = 4.0 Hz), 140.9, 128.9 (d, $J_{C\text{-}}$

F = 10.0 Hz), 127.1, 125.4 (d, J_{C-F} = 3.0 Hz), 120.3 (d, J_{C-F} = 18.0 Hz), 118.2, 112.7 (d, J_{C-F} = 23.5 Hz), 64.3, 52.7, 44.1, 33.8, 30.9, 28.7, 26.5, 24.4, 24.1, 19.3, 13.8; **^{19}F NMR** (377 MHz, $CDCl_3$): δ –113.1; **IR** ν_{max} (film)/cm^{-1} 2957, 2864, 1710, 1664, 1627, 1578, 1465, 1429, 1380, 1315, 1247, 1162, 1113, 1066, 1026, 983, 853, 814, 730; **HRMS** (ESI$^+$) calculated for $C_{25}H_{35}FN_3O_3$ [M+H]$^+$: 444.2657, found: 444.2654.

(*E*)-Butyl 3-{3-[(*E*)-4,4-dimethylpent-2-enoyl]-5-methyl-2-[(*E*)-piperidin-1-yl diazenyl]phenyl}acrylate (5g)

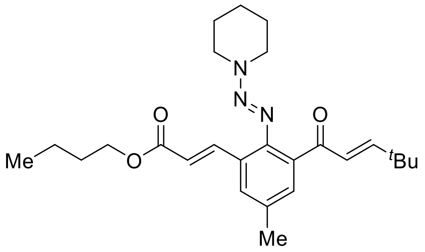

Prepared according to **General Procedure E**, using aldehyde **1i** (69 mg), 3,3-dimethylbut-1-yne (55 μL) and *n*-butyl acrylate (108 μL). Purification by flash column chromatography (petrol/Et_2O, 4:1) afforded the title compound (103 mg, 79% yield) as a brown oil. **^{1}H NMR** (400 MHz, $CDCl_3$): δ 8.30 (d, *J* = 16.0 Hz, 1H, CH=C*H*-Ar), 7.49 (d, *J* = 1.5 Hz, 1H, Ar-*H*), 7.17 (d, *J* = 1.5 Hz, 1H, Ar-*H*), 6.60 (d, *J* = 16.0 Hz, 1H, CO-CH=C*H*), 6.43 (d, *J* = 16.0 Hz, 1H, C*H*=CH-Ar), 5.89 (d, *J* = 16.0 Hz, 1H, CO-C*H*=CH), 4.17 (t, *J* = 6.5 Hz, 2H, CO_2CH_2), 3.90 – 3.56 (2 × br. s, 4H, $N(CH_2)_2$), 2.33 (s, 3H, Ar-CH_3), 1.70 – 1.61 (m, 8H, 4 × CH_2), 1.47 – 1.36 (m, 2H, CH_2-CH_3), 0.99 (s, 9H, $C(CH_3)_3$), 0.93 (t, *J* = 7.5 Hz, 3H, CH_2-CH_3); **^{13}C NMR** (101 MHz, $CDCl_3$): δ 196.2, 167.3, 155.6, 146.1, 141.4, 134.9, 133.6, 131.6, 129.1, 128.9, 126.2, 118.7, 64.3, 52.5, 44.2, 33.6, 30.8, 28.8, 26.3, 24.3, 24.1, 20.9, 19.3, 13.8; **IR** ν_{max} (film)/cm^{-1} 2957, 2863, 1710, 1675, 1655, 1628, 1421, 1329, 1255, 1171, 1107, 983, 918, 855, 730; **HRMS** (ESI$^+$) calculated for $C_{26}H_{38}N_3O_3$ [M+H]$^+$: 440.2908, found: 440.2900.

(*E*)-Butyl 3-{3-[(*E*)-4,4-dimethylpent-2-enoyl]-5-fluoro-2-[(*E*)-piperidin-1-yl diazenyl]phenyl}acrylate (5h)

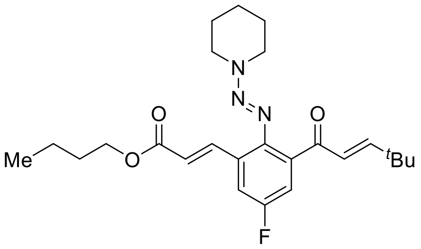

Prepared according to **General Procedure E**, using aldehyde **1h** (71 mg), 3,3-dimethylbut-1-yne (55 μL) and *n*-butyl acrylate (108 μL). Purification by flash column chromatography (petrol/Et_2O, 4:1) afforded the title compound (82 mg, 62% yield) as a yellow oil. **^{1}H NMR** (400 MHz, $CDCl_3$): δ 8.27 (dd, J = 16.0, 1.5 Hz, 1H, CH=C*H*-Ar), 7.36 (dd, J = 9.0, 3.0 Hz, 1H, Ar-*H*), 7.08 (dd, J = 8.0, 3.0 Hz, 1H, Ar-*H*), 6.63 (d, J = 16.0 Hz, 1H, CO-CH=C*H*), 6.40 (d, J = 16.0 Hz, 1H, C*H*=CH-Ar), 5.89 (d, J = 16.0 Hz, 1H, CO-C*H*=CH), 4.19 (t, J = 6.5 Hz, 2H, CO_2CH_2), 3.88 – 3.59 (2 × br. s, 4H, $N(CH_2)_2$), 1.70 – 1.62 (m, 8H, 4 × CH_2), 1.47 – 1.37 (m, 2H, CH_2-CH_3), 1.00 (s, 9H, $C(CH_3)_3$), 0.94 (t, J = 7.5 Hz, 3H, CH_2-CH_3); **^{13}C NMR** (101 MHz, $CDCl_3$): δ 194.7, 167.0, 159.9 (d, $J_{C\text{-}F}$ = 246.0 Hz), 156.7, 144.7 (d, $J_{C\text{-}F}$ = 2.5 Hz), 140.3, 135.2 (d, $J_{C\text{-}F}$ = 6.5 Hz), 130.9 (d, $J_{C\text{-}F}$ = 7.5 Hz), 125.7, 120.0, 117.7 (d, $J_{C\text{-}F}$ = 24.0 Hz), 114.6 (d, $J_{C\text{-}F}$ = 23.0 Hz), 64.5, 52.6, 44.3, 33.8, 30.8, 28.8, 26.4, 24.3, 24.1, 19.3, 13.8; **^{19}F NMR** (377 MHz, $CDCl_3$): δ –117.2; **IR** ν_{max} (film)/cm^{-1} 2958, 2864, 1712, 1676, 1632, 1594, 1417, 1329, 1259, 1170, 1109, 983, 916, 857, 777, 731; **HRMS** (ESI$^+$) calculated for $C_{25}H_{35}FN_3O_3$ $[M+H]^+$: 444.2657, found: 444.2655.

(*E*)-Butyl 3-{6-chloro-3-[(*E*)-4,4-dimethylpent-2-enoyl]-2-[(*E*)-piperidin-1-yl diazenyl]phenyl}acrylate (5i)

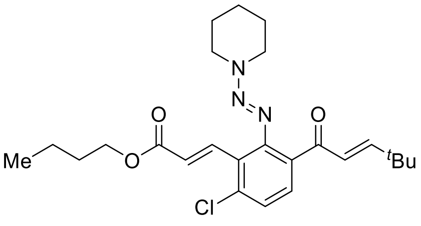

Prepared according to **General Procedure E**, using aldehyde **1c** (75 mg), 3,3-dimethylbut-1-yne (55 μL) and *n*-butyl acrylate (108 μL). Purification by flash column chromatography (petrol/Et_2O, 4:1) afforded the title compound (79 mg, 57% yield) as a yellow oil. **^{1}H NMR** (400 MHz, $CDCl_3$): δ 8.05 (d, J = 16.0 Hz, 1H, CH=C*H*-Ar), 7.26 (br. s, 2H, Ar-*H*), 6.64 (app. dd, J = 16.0, 7.0 Hz, 2H, CO-CH=C*H* and C*H*=CH-Ar), 5.85 (d, J = 16.0 Hz, 1H, CO-C*H*=CH), 4.20 (t, J = 6.5 Hz, 2H, CO_2CH_2), 3.87 – 3.63 (2 × br. s, 4H, $N(CH_2)_2$), 1.72 – 1.63 (m, 8H, 4 × CH_2), 1.47 – 1.37 (m, 2H, CH_2-CH_3), 1.00 (s, 9H, $C(CH_3)_3$), 0.95 (t, J = 7.5 Hz, 3H, CH_2-CH_3); **^{13}C NMR** (101 MHz, $CDCl_3$): δ 195.0, 167.1,

156.2, 150.3, 138.2, 136.6, 132.2, 130.1, 127.3, 126.4, 125.9, 125.8, 64.4, 52.7, 44.3, 33.7, 30.8, 28.7, 26.4, 24.4, 24.0, 19.2, 13.8; **IR** ν_{max} (film)/cm^{-1} 2960, 1714, 1676, 1619, 1567, 1365, 1301, 1257, 1169, 1109, 1064, 982, 854, 814, 728; **HRMS** (ESI$^+$) calculated for $C_{25}H_{35}ClN_3O_3$ [M+H]$^+$: 460.2361, found: 460.2356.

(*E*)-Butyl 3-{3-[(*E*)-4,4-dimethylpent-2-enoyl]-2-[(*E*)-piperidin-1-yldiazenyl]naphthalen-1-yl}acrylate (5j)

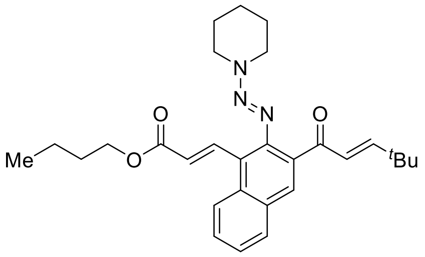

Prepared according to **General Procedure E**, using aldehyde **1j** (80 mg), 3,3-dimethylbut-1-yne (55 µL) and *n*-butyl acrylate (108 µL). Purification by flash column chromatography (gradient petrol/Et_2O, 4:1 to 3:1) afforded the title compound (70 mg, 49% yield) as a brown oil. **^{1}H NMR** (400 MHz, $CDCl_3$): δ 8.45 (d, J = 16.5 Hz, 1H, CH=C*H*-Ar), 8.25 (d, J = 8.5 Hz, 1H, Ar-*H*), 7.87 – 7.83 (m, 2H, Ar-*H*), 7.56 (ddd, J = 8.5, 7.0, 1.5 Hz, 1H, Ar-*H*), 7.46 (ddd, J = 8.0, 7.0, 1.0 Hz, 1H, Ar-*H*), 6.64 (d, J = 16.0 Hz, 1H, CO-CH=C*H*), 6.45 (d, J = 16.5 Hz, 1H, C*H*=CH-Ar), 6.03 (d, J = 16.0 Hz, 1H, CO-C*H*=CH), 4.25 (t, J = 6.5 Hz, 2H, CO_2CH_2), 3.95 – 3.59 (m, 4H, $N(CH_2)_2$), 1.76 – 1.66 (m, 8H, 4 × CH_2), 1.52 – 1.41 (m, 2H, CH_2-CH_3), 1.02 (s, 9H, $C(CH_3)_3$), 0.98 (t, J = 7.5 Hz, 3H, CH_2-CH_3); **^{13}C NMR** (101 MHz, $CDCl_3$): δ 196.2, 167.3, 156.4, 145.5, 140.6, 133.2, 132.6, 131.1, 130.4, 129.4, 128.1, 126.5, 125.8, 125.4, 125.2, 124.8, 64.5, 52.7, 44.2, 33.8, 30.9, 28.9, 26.5, 24.4, 24.3, 19.4, 13.9; **IR** ν_{max} (film)/cm^{-1} 2980, 1712, 1677, 1656, 1623, 1444, 1384, 1359, 1303, 1259, 1170, 1110, 1073, 977, 852, 752; **HRMS** (ESI$^+$) calculated for $C_{29}H_{38}N_3O_3$ [M+H]$^+$: 476.2908, found: 476.2914.

(E)-*tert*-Butyl 3-{3-[(E)-4,4-dimethylpent-2-enoyl]-2-[(E)-piperidin-1-yldiazenyl] phenyl}acrylate (5k)

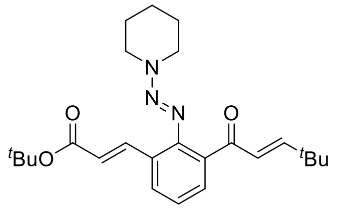

Prepared according to **General Procedure E**, using aldehyde **1a** (65 mg), 3,3-dimethylbut-1-yne (55 µL) and *tert*-butyl acrylate (110 µL). Purification by flash column chromatography (petrol/Et_2O, 85:15) afforded the title compound (98 mg, 77% yield) as a white solid. **m.p.** (Et_2O): 126 – 128 ºC; **^{1}H NMR** (400 MHz, $CDCl_3$): δ 8.24 (d, J = 16.0 Hz, 1H, CH=C*H*-Ar), 7.67 (dd, J = 8.0, 1.0 Hz, 1H, Ar-*H*), 7.34 (dd, J = 7.5, 1.5 Hz, 1H, Ar-*H*), 7.17 (t, J = 7.5 Hz, 1H, Ar-*H*), 6.61 (d, J = 16.0 Hz, 1H, CO-CH=C*H*), 6.37 (d, J = 16.0 Hz, 1H, C*H*=CH-Ar), 5.89 (d, J = 16.0 Hz, 1H, CO-C*H*=CH), 3.90 – 3.60 (2 × br. s, 4H, N(CH_2)$_2$), 1.66 (br. s, 6H, 3 × CH_2), 1.50 (s, 9H, C(CH_3)$_3$), 0.98 (s, 9H, C(CH_3)$_3$); **^{13}C NMR** (101 MHz, $CDCl_3$): δ 196.1, 166.5, 155.8, 148.1, 140.1, 133.7, 130.7, 129.2, 128.4, 126.1, 125.1, 120.7, 80.3, 52.6, 44.2, 33.7, 28.8, 28.3, 26.4, 24.3, 24.1; **IR** ν_{max} (film)/cm^{-1} 2961, 2862, 1705, 1676, 1626, 1413, 1366, 1320, 1292, 1255, 1148, 1108, 983, 917, 852, 729; **HRMS** (ESI$^+$) calculated for $C_{25}H_{36}N_3O_3$ $[M+H]^+$: 426.2751, found: 426.2743.

(E)-3-{3-[(E)-4,4-Dimethylpent-2-enoyl]-2-[(E)-piperidin-1-yldiazenyl]phenyl}-*N*,*N*-dimethylacrylamide (5l)

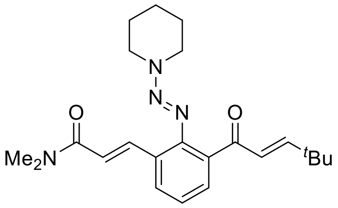

Prepared according to **General Procedure E**, using aldehyde **1a** (65 mg), 3,3-dimethylbut-1-yne (55 µL) and *N,N*-dimethylacrylamide (77 µL). Purification by flash column chromatography (CH_2Cl_2/Et_2O, 4:1) afforded the title compound (72 mg, 61% yield) as a pale yellow solid. **m.p.** (Et_2O): 177 – 178 ºC; **^{1}H NMR** (400 MHz, $CDCl_3$): δ 8.27 (d, J = 15.5 Hz, 1H, CH=C*H*-Ar), 7.68 (dd, J = 7.5, 1.0 Hz, 1H, Ar-*H*), 7.35 (dd, J = 7.5, 1.5 Hz, 1H, Ar-*H*), 7.19 (t, J = 7.5 Hz, 1H, Ar-*H*), 6.90 (d, J = 15.5 Hz, 1H, C*H*=CH-Ar), 6.62 (d, J = 16.0 Hz, 1H, CO-CH=C*H*), 5.91 (d, J = 16.0 Hz, 1H, CO-C*H*=CH), 3.90 – 3.60 (2 × br. s, 4H, N(CH_2)$_2$), 3.17 (s, 3H, N-CH_3), 3.06 (s, 3H, N-CH_3), 1.66 (br. s, 6H, 3 × CH_2), 1.00 (s, 9H, C(CH_3)$_3$); **^{13}C NMR** (101 MHz, $CDCl_3$): δ 196.4, 167.1, 155.9, 148.2, 139.2, 133.8, 130.3, 130.2, 128.6, 126.2, 125.1, 118.5, 52.7, 44.4, 37.6, 36.0, 33.7, 28.9, 26.4, 24.4, 24.2; **IR** ν_{max} (film)/cm^{-1} 2950, 2862, 1647, 1608, 1392, 1298, 1259, 1184, 1140, 1108, 983, 921, 727; **HRMS** (ESI$^+$) calculated for $C_{23}H_{33}N_4O_2$ $[M+H]^+$: 397.2598, found: 397.2596.

(*E*)-4,4-Dimethyl-1-{3-[(*E*)-2-(phenylsulfonyl)vinyl]-2-[(*E*)-piperidin-1-yldiazenyl]phenyl}pent-2-en-1-one (5m)

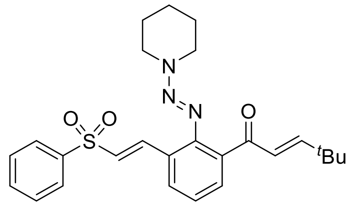

Prepared according to **General Procedure E**, using aldehyde **1a** (65 mg), 3,3-dimethylbut-1-yne (55 μL) and (vinylsulfonyl)benzene (126 mg). Purification by flash column chromatography (petrol/Et_2O, 1:1) afforded the title compound (95 mg, 68% yield) as a pale yellow solid. **m.p.** (Et_2O): 161 – 164 ºC; **^{1}H NMR** (400 MHz, $CDCl_3$): δ 8.23 (d, J = 15.5 Hz, 1H, CH=C*H*-Ar), 7.95 – 7.90 (m, 2H, Ar-*H*), 7.62 – 7.49 (m, 4H, Ar-*H*), 7.37 (dd, J = 7.5, 1.5 Hz, 1H, Ar-*H*), 7.16 (t, J = 7.5 Hz, 1H, Ar-*H*), 6.93 (d, J = 15.5 Hz, 1H, C*H*=CH-Ar), 6.61 (d, J = 16.0 Hz, 1H, CO-CH=C*H*), 5.91 (d, J = 16.0 Hz, 1H, CO-C*H*=CH), 3.85 – 3.62 (2 × br. s, 4H, N(CH_2)$_2$), 1.67 (br. s, 6H, 3 × CH_2), 0.99 (s, 9H, C(CH_3)$_3$); **^{13}C NMR** (101 MHz, $CDCl_3$): δ 195.8, 156.4, 148.6, 140.9, 139.6, 133.9, 133.3, 131.7, 129.7, 129.3, 128.1, 127.7, 126.8, 126.0, 125.1, 52.7, 44.3, 33.7, 28.8, 26.4, 24.3, 24.0; **IR** ν_{max} (film)/cm^{-1} 2952, 2861, 1673, 1654, 1613, 1408, 1305, 1185, 1145, 1110, 1085, 984, 913, 847, 727, 687, 647; **HRMS** (ESI$^+$) calculated for $C_{26}H_{32}N_3O_3S$ [M+H]$^+$: 466.2159, found: 466.2156.

(*E*)-4,4-Dimethyl-1-{2-[(*E*)-piperidin-1-yldiazenyl]-3-[(*E*)-4-(trifluoromethyl)styryl]phenyl}pent-2-en-1-one (5n)

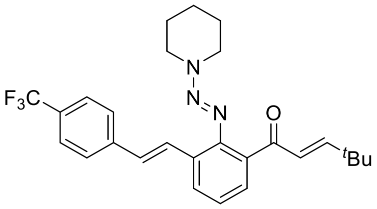

Prepared according to **General Procedure E**, using aldehyde **1a** (65 mg), 3,3-dimethylbut-1-yne (55 μL) and 1-(trifluoromethyl)-4-vinylbenzene (110 μL). Purification by flash column chromatography (petrol/Et_2O, 4:1) afforded the title compound (81 mg, 58% yield) as a yellow oil. **^{1}H NMR** (400 MHz, $CDCl_3$): δ 7.86 (d, J = 16.5 Hz, 1H, CH=C*H*-Ar), 7.77 (dd, J = 7.5, 1.0 Hz, 1H, Ar-*H*), 7.60 (app. s, 4H, Ar-*H*), 7.33 (dd, J = 7.5, 1.5 Hz, 1H, Ar-*H*), 7.23 (t, J = 7.5 Hz, 1H, Ar-*H*), 7.12 (d, J = 16.5 Hz, 1H, C*H*=CH-Ar), 6.69 (d, J = 16.0 Hz, 1H, CO-CH=C*H*), 5.95 (d, J = 16.0 Hz, 1H, CO-C*H*=CH), 3.78 (br. s, 4H, N(CH_2)$_2$), 1.70 (br. s, 6H, 3 × CH_2), 1.03 (s, 9H, C(CH_3)$_3$); **^{13}C NMR** (101 MHz, $CDCl_3$): δ 196.4, 155.8, 147.3, 141.4, 133.8, 131.4, 129.1, 128.9 (q, J_{C-F} = 32.5 Hz), 127.9, 127.8, 127.5, 126.7, 126.3, 125.7 (q, J_{C-F} = 3.5 Hz), 125.3, 124.4 (q, J_{C-F} = 271.5 Hz), 52.4, 44.2, 33.7, 28.9, 26.2, 24.5, 24.2; **^{19}F NMR** (377 MHz, $CDCl_3$): δ –62.4; **IR** ν_{max} (film)/cm^{-1} 2954, 2862, 1674, 1655, 1613, 1416, 1321, 1184,

1163, 1109, 1067, 1015, 979, 913, 826, 752, 734, 681; **HRMS** (ESI^+) $C_{27}H_{31}F_3N_3O$ requires 470.2414 $[M+H]^+$, found 470.2408.

2. Proto-detriazenation of the Hydroacylation/*ortho*-C–H Olefination Product

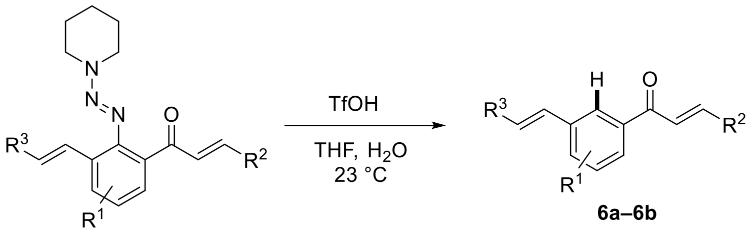

(*E*)-Butyl 3-{3-[(*E*)-4,4-dimethylpent-2-enoyl]-5-methylphenyl}acrylate (6a)

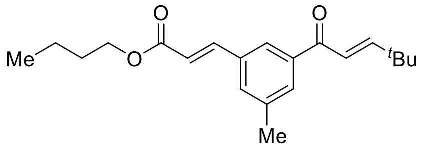

Under N_2 atmosphere, triflic acid (20 μL, 0.23 mmol) was added to a solution of aryltriazenyl enone **5g** (44 mg, 0.1 mmol) in THF (2 mL) at 0 ºC. The reaction mixture was stirred at 23 ºC for 1 h, followed by the addition of H_2O (25 μL). It was then further stirred at 23 ºC for 24 h and concentrated under reduced pressure. Purification by flash column chromatography (gradient petrol/CH_2Cl_2, 7:3 to 5:5) afforded the title compound (24 mg, 76% yield) as a colorless oil. **^{1}H NMR** (400 MHz, $CDCl_3$): δ 7.84 (s, 1H, Ar-*H*), 7.74 – 7.67 (m, 2H, Ar-*H* and CH=C*H*-Ar), 7.52 (s, 1H, Ar-*H*), 7.08 (d, *J* = 15.5 Hz, 1H, CO-CH=C*H*), 6.75 (d, *J* = 15.5 Hz, 1H, CO-C*H*=CH), 6.50 (d, *J* = 16.0 Hz, 1H, C*H*=CH-Ar), 4.22 (t, *J* = 6.5 Hz, 2H, CO_2CH_2), 2.44 (s, 3H, Ar-CH_3), 1.74 – 1.65 (m, 2H, CH_2), 1.50 – 1.39 (m, 2H, CH_2-CH_3), 1.17 (s, 9H, $C(CH_3)_3$), 0.97 (t, *J* = 7.5 Hz, 3H, CH_2-CH_3); **^{13}C NMR** (101 MHz, $CDCl_3$): δ 191.2, 167.0, 160.4, 143.8, 139.2, 139.1, 134.9, 132.5, 130.9, 125.6, 120.9, 119.4, 64.7, 34.4, 30.9, 28.9, 21.5, 19.3, 13.9; **IR** ν_{max} (film)/cm^{-1} 2959, 2871, 1713, 1671, 1640, 1620, 1334, 1272, 1172, 982, 848; **HRMS** (ESI^+) calculated for $C_{21}H_{29}O_3$ $[M+H]^+$: 329.2111, found: 329.2125.

(*E*)-Butyl 3-(3-cinnamoylphenyl)acrylate (6b)

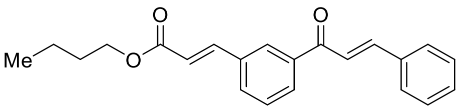

Under N_2 atmosphere, triflic acid (20 µL, 0.23 mmol) was added to a solution of aryltriazenyl enone **5d** (45 mg, 0.1 mmol) in THF (2 mL) at 0 ºC. The reaction mixture was stirred at 23 ºC for 1 h, followed by the addition of H_2O (25 µL). It was then further stirred at 23 ºC for 24 h and concentrated under reduced pressure. Purification by flash column chromatography (petrol/Et_2O, 9:1) afforded the title compound (29 mg, 87% yield) as a pale yellow solid. **m.p.** (Et_2O): 80 – 81 ºC; **^{1}H NMR** (400 MHz, $CDCl_3$): δ 8.15 (s, 1H, Ar-*H*), 8.02 (d, *J* = 7.5 Hz, 1H, Ar-*H*), 7.84 (d, *J* = 15.5 Hz, 1H, CH=C*H*-Ar), 7.78 – 7.71 (m, 2H, CO-CH=C*H* and Ar-*H*), 7.69 – 7.64 (m, 2H, Ar-*H*), 7.56 – 7.50 (m, 2H, Ar-*H* and C*H*=CH-Ar), 7.47 – 7.41 (m, 3H, Ar-*H*), 6.55 (d, *J* = 16.0 Hz, 1H, CO-C*H*=CH), 4.23 (t, *J* = 6.5 Hz, 2H, CO_2CH_2), 1.75 – 1.66 (m, 2H, C*H*$_2$), 1.50 – 1.39 (m, 2H, C*H*$_2$-CH_3), 0.97 (t, *J* = 7.5 Hz, 3H, C*H*$_3$); **^{13}C NMR** (101 MHz, $CDCl_3$): δ 190.0, 166.9, 145.6, 143.5, 139.0, 135.2, 134.8, 132.0, 130.9, 130.1, 129.4, 129.1, 128.7, 128.1, 121.8, 119.8, 64.7, 30.9, 19.3, 13.9; **IR** ν_{max} (film)/cm^{-1} 2959, 2873, 1709, 1665, 1639, 1606, 1575, 1450, 1305, 1162, 1055, 1033, 979, 863, 805, 761, 687; **HRMS** (ESI$^+$) calculated for $C_{22}H_{23}O_3$ $[M+H]^+$: 335.1642, found: 335.1641.

VII. Hydroacylation/Bromination/Proto-detriazenation

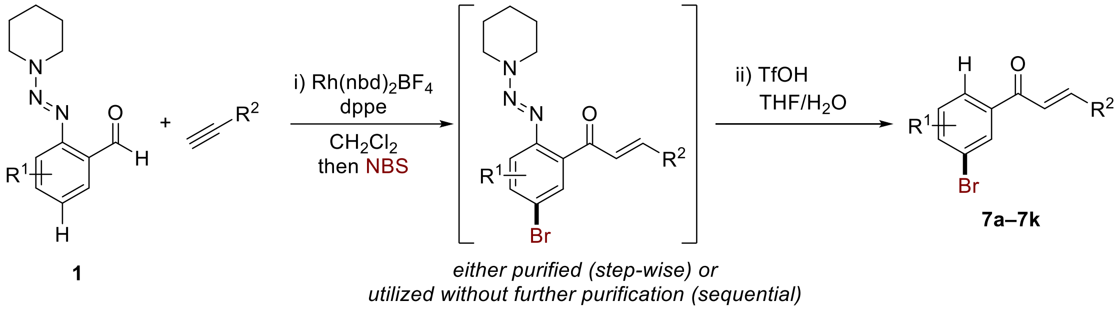

General Procedure F: i) A reaction vial charged with $Rh(nbd)_2BF_4$ (3.7 mg, 0.01 mmol, 5 mol%) and dppe (4.0 mg, 0.01 mmol, 5 mol%) was evacuated and back-filled with N_2 gas for 3 times, and to this was added CH_2Cl_2 (0.25 mL). The resulting solution was bubbled with H_2 gas for 2 min and then with N_2 gas until the volume of the solution had become around 0.1 mL. This solution was then transferred to a N_2-purged reaction vial containing aldehyde (0.2 mmol, 1.0 equiv), with the use of additional 0.1 mL of CH_2Cl_2. Alkyne (0.30 mmol, 1.5 equiv) was added and the reaction mixture was stirred at 23 ºC for 16 h. To this was then added a solution of *N*-bromosuccinimide (NBS) (53.4 mg, 0.3 mmol, 1.5 equiv), and the resulting mixture was further stirred at 23 ºC for 1–2 h. Upon completion, the reaction was diluted with Et_2O, filtered through a pad of silica and concentrated under reduced pressure. The crude was either purified by flash column chromatography or utilized for the next step without further purification.

ii) The above obtained hydroacylation/bromination product (either crude or purified) was dissolved in THF (0.05 M), and to the resulting solution was added triflic acid (3.4 equiv) at 0 ºC. The reaction was stirred at 23 ºC until the hydroacylation/bromination product was all consumed (5-60 min). H_2O (50 µL) was then added and the mixture was further stirred at 23 ºC for 24 h. After completion, the reaction was concentrated under reduced pressure and purified by flash column chromatography to afford the corresponding bromoaryl enone product.

(*E*)-1-{5-Bromo-2-[(*E*)-piperidin-1-yldiazenyl]phenyl}-4,4-dimethylpent-2-en-1-one (S2)

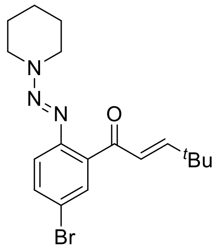

Prepared according to **General Procedure F (i)**, using aldehyde **1a** (43 mg) and 3,3-dimethyl-1-butyne (37 μL). Purification by flash column chromatography (petrol/CH_2Cl_2, 1:1) afforded the title compound (72 mg, 95% yield) as a yellow oil. **^{1}H NMR** (400 MHz, $CDCl_3$): δ 7.53 – 7.45 (m, 2H, Ar-*H*), 7.36 (d, *J* = 8.5 Hz, 1H, Ar-*H*), 6.72 (d, *J* = 16.0 Hz, 1H, CO-CH=C*H*), 6.36 (d, *J* = 16.0 Hz, 1H, CO-C*H*=CH), 3.73 (br. s, 4H, N(C*H*$_2$)$_2$), 1.67 (br. s, 6H, 3 × C*H*$_2$), 1.04 (s, 9H, C(C*H*$_3$)$_3$); **^{13}C NMR** (101 MHz, $CDCl_3$): δ 195.4, 157.4, 147.9, 136.4, 133.8, 131.4, 126.4, 120.3, 118.5, 52.9, 44.0, 33.9, 28.9, 26.2, 24.4, 24.3; **IR** ν_{max} (film)/cm^{-1} 2941, 2859, 1663, 1614, 1422, 1391, 1355, 1297, 1184, 1105, 983, 853, 825; **HRMS** (ESI$^+$) calculated for $C_{18}H_{25}BrN_3O$ [M+H]$^+$: 378.1176, found: 378.1172.

(*E*)-1-(3-Bromophenyl)-4,4-dimethylpent-2-en-1-one (7a)

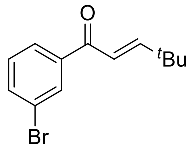

Prepared according to **General Procedure F (ii)**, using aryltriazenyl enone **S2** (72 mg, 0.19 mmol), TfOH (0.06 mL, 0.65 mmol), THF (3.8 mL) and H_2O (48 μL). Purification by flash column chromatography (petrol/CH_2Cl_2, 7:3) afforded the title compound (41 mg, 80% yield) as a yellow oil.

Bromoaryl enone **7a** was also prepared according to **General Procedure F** in a **sequential manner** without the purification of **S2** prior to the second step, using aldehyde **1a** (43 mg) and 3,3-dimethyl-1-butyne (37 μL). Purification by flash column chromatography (petrol/CH_2Cl_2, 7:3) afforded the title compound (41 mg, 77% yield) as a yellow oil.

^{1}H NMR (400 MHz, $CDCl_3$): δ 8.04 (s, 1H, Ar-*H*), 7.83 (d, *J* = 8.0 Hz, 1H, Ar-*H*), 7.67 (d, *J* = 8.0 Hz, 1H, Ar-*H*), 7.35 (t, *J* = 8.0 Hz, 1H, Ar-*H*), 7.08 (d, *J* = 15.5 Hz, 1H, CO-CH=C*H*), 6.71 (d, *J* = 15.5 Hz, 1H, CO-C*H*=CH), 1.15 (s, 9H, C(C*H*$_3$)$_3$); **^{13}C NMR** (101 MHz, $CDCl_3$): δ 190.1, 160.8, 140.1, 135.5, 131.6, 130.2, 127.2, 123.0, 120.6, 34.4, 28.8; **ν_{max}** (film)/cm^{-1} 2961, 1670, 1617, 1565, 1302, 1216, 984, 790, 718, 687; **HRMS** (ESI$^+$) calculated for $C_{13}H_{16}BrO$ [M+H]$^+$: 267.0379, found: 267.0380.

(*E*)-1-(3-Bromophenyl)non-2-en-1-one (7b)

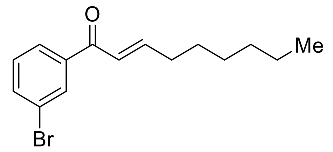

Prepared according to **General Procedure F** in a **sequential manner**, using aldehyde **1a** (43 mg) and 1-octyne (44 μL). Purification by flash column chromatography (petrol/Et_2O, 98:2) afforded the title compound (38 mg, 65% yield) as a yellow oil. **^{1}H NMR** (400 MHz, $CDCl_3$): δ 8.04 (t, J = 2.0 Hz, 1H, Ar-*H*), 7.85 – 7.81 (m, 1H, Ar-*H*), 7.66 (ddd, J = 8.0, 2.0, 1.0 Hz, 1H, Ar-*H*), 7.34 (t, J = 8.0 Hz, 1H, Ar-*H*), 7.08 (dt, J = 15.5, 7.0 Hz, 1H, CO-CH=C*H*), 6.81 (dt, J = 15.5, 1.5 Hz, 1H, CO-C*H*=CH), 2.35 – 2.28 (m, 2H, CH=CH-CH_2), 1.56 – 1.46 (m, 2H, CH=CH-CH_2-CH_2), 1.39 – 1.26 (m, 6H, 3 × CH_2), 0.89 (t, J = 7.0 Hz, 3H, CH_3); **^{13}C NMR** (101 MHz, $CDCl_3$): δ 189.5, 151.4, 139.9, 135.5, 131.7, 130.2, 127.1, 125.5, 122.9, 33.0, 31.7, 29.0, 28.2, 22.7, 14.2; **IR** ν_{max} (film)/cm^{-1} 2926, 2855, 1671, 1619, 1564, 1466, 1420, 1289, 1214, 971, 783, 700; **HRMS** (ESI$^+$) calculated for $C_{15}H_{20}BrO$ [M+H]$^+$: 295.0692, found: 295.0692.

(*E*)-1-{5-Bromo-2-[(*E*)-piperidin-1-yldiazenyl]phenyl}-3-cyclopentylprop-2-en-1-one (S3)

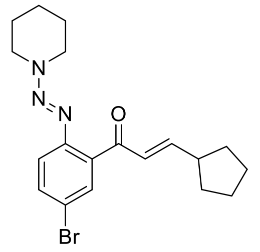

Prepared according to **General Procedure F (i)**, using aldehyde **1a** (43 mg) and ethynylcyclopentane (35 μL). Purification by flash column chromatography (petrol/CH_2Cl_2, 1:1) afforded the title compound (68 mg, 87% yield) as a yellow solid. **m.p.** (petrol): 67 – 69 ºC; **^{1}H NMR** (400 MHz, $CDCl_3$): δ 7.53 – 7.44 (m, 2H, Ar-*H*), 7.35 (d, J = 8.5 Hz, 1H, Ar-*H*), 6.67 (dd, J = 15.5, 8.0 Hz, 1H, CO-CH=C*H*), 6.40 (d, J = 15.5 Hz, 1H, CO-C*H*=CH), 3.73 (br. s, 4H, N(CH_2)$_2$), 2.63 – 2.51 (m, 1H, CH=CH-C*H*), 1.83 – 1.52 (m, 12H, 6 × CH_2), 1.40 – 1.30 (m, 2H, Cypent-*H*); **^{13}C NMR** (101 MHz, $CDCl_3$): δ 195.1, 152.3, 147.9, 136.2, 133.8, 131.4, 129.4, 120.2, 118.4, 53.0, 44.2, 43.2, 32.6, 26.4, 25.4, 24.4, 24.3; **Ir** ν_{max} (film)/cm^{-1} 2942, 2859, 1662, 1615, 1423, 1393, 1355, 1297, 1260, 1184, 1106, 981; **HRMS** (ESI$^+$) calculated for $C_{19}H_{25}BrN_3O$ [M+H]$^+$: 390.1176, found: 390.1173.

(*E*)-1-(3-Bromophenyl)-3-cyclopentylprop-2-en-1-one (7c)

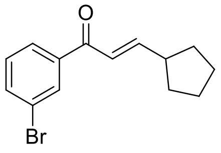

Prepared according to **General Procedure F (ii)**, using aryltriazenyl enone **S3** (66 mg, 0.17 mmol), TfOH (0.05 mL, 0.58 mmol), THF (3.4 mL) and H_2O (43 μL). Purification by flash column chromatography (petrol/CH_2Cl_2, 3:2) afforded the title compound (36 mg, 77% yield) as a yellow oil.

Bromoaryl enone **7c** was also prepared according to **General Procedure F** in a **sequential manner** without the purification of **S3** prior to the second step, using aldehyde **1a** (43 mg), ethynylcyclopentane (35 μL). Purification by flash column chromatography (petrol/CH_2Cl_2, 3:2) afforded the title compound (34 mg, 61% yield) as a yellow oil.

^{1}H NMR (400 MHz, $CDCl_3$): δ 8.03 (s, 1H, Ar-*H*), 7.83 (d, *J* = 8.0 Hz, 1H, Ar-*H*), 7.66 (d, *J* = 8.0 Hz, 1H, Ar-*H*), 7.33 (t, *J* = 8.0 Hz, 1H, Ar-*H*), 7.06 (dd, *J* = 15.5, 8.0 Hz, 1H, CO-CH=C*H*), 6.79 (d, *J* = 15.5 Hz, 1H, CO-C*H*=CH), 2.76 – 2.64 (m, 1H, CH=CH-C*H*), 1.94 – 1.84 (m, 2H, Cypent-*H*), 1.78 – 1.58 (m, 4H, Cypent-*H*), 1.51 – 1.41 (m, 2H, Cypent-*H*); **^{13}C NMR** (101 MHz, $CDCl_3$): δ 189.7, 155.4, 140.0, 135.5, 131.6, 130.2, 127.1, 123.5, 122.9, 43.6, 32.7, 25.5; **IR** ν_{max} (film)/cm^{-1} 2952, 2867, 1669, 1616, 1563, 1421, 1360, 1300, 1254, 1212, 983, 789, 719, 703; **HRMS** (ESI$^+$) calculated for $C_{14}H_{16}BrO$ $[M+H]^+$: 279.0379, found: 279.0381.

(*E*)-1-(3-Bromophenyl)-5-phenylpent-2-en-1-one (7d)

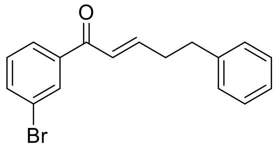

Prepared according to **General Procedure F** in a **sequential manner**, using aldehyde **1a** (43 mg) and 4-phenyl-1-butyne (42 μL). Purification by flash column chromatography (petrol/Et_2O, 98:2) afforded the title compound (32 mg, 51% yield) as a yellow oil. **^{1}H NMR** (400 MHz, $CDCl_3$): δ 7.99 (s, 1H, Ar-*H*), 7.78 (d, *J* = 8.0 Hz, 1H, Ar-*H*), 7.67 (d, *J* = 8.0 Hz, 1H, Ar-*H*), 7.36 – 7.29 (m, 3H, Ar-*H*), 7.26 – 7.19 (m, 3H, Ar-*H*), 7.09 (dt, *J* = 15.5, 7.0 Hz, 1H, CO-CH=C*H*), 6.79 (d, *J* = 15.5 Hz, 1H, CO-C*H*=CH), 2.86 (t, *J* = 7.5 Hz, 2H, C*H*$_2$-Ph), 2.66 (q, *J* = 7.5 Hz, 2H, CH=CH-C*H*$_2$); **^{13}C NMR** (101 MHz, $CDCl_3$): δ 189.5, 149.6, 140.8, 139.8, 135.6, 131.7, 130.2, 128.7, 128.5, 127.2, 126.4, 126.3, 122.9, 34.7, 34.5; **IR** ν_{max} (film)/cm^{-1} 3062, 3026, 2924, 2853, 1670, 1619, 1563, 1420, 1299, 1213, 1068, 1030, 981, 789, 748, 698; **HRMS** (ESI$^+$) calculated for $C_{17}H_{16}BrO$ $[M+H]^+$: 315.0379, found: 315.0380.

2-{(*E*)-7-[5-Bromo-2-((*E*)-piperidin-1-yldiazenyl)phenyl]-7-oxohept-5-en-1-yl}isoindoline-1,3-dione (S4)

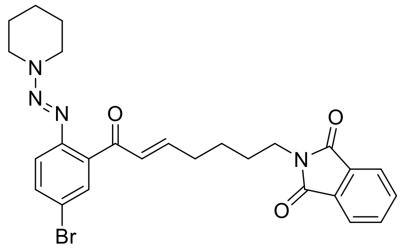

Prepared according to **General Procedure F (i)**, using aldehyde **1a** (43 mg) and 2-(hex-5-yn-1-yl)isoindoline-1,3-dione (68 mg). Purification by flash column chromatography (gradient petrol/Et_2O, 7:3 to 6:4) afforded the title compound (83 mg, 79% yield) as a thick yellow oil. **^{1}H NMR** (400 MHz, $CDCl_3$): δ 7.83 – 7.76 (m, 2H, Ar-*H*), 7.71 – 7.65 (m, 2H, Ar-*H*), 7.49 – 7.41 (m, 2H, Ar-*H*), 7.33 (d, *J* = 8.5 Hz, 1H, Ar-*H*), 6.70 – 6.60 (m, 1H, CO-CH=C*H*), 6.42 (d, *J* = 15.5 Hz, 1H, CO-C*H*=CH), 3.73 – 3.61 (m, 6H, N(CH_2)$_2$ and CH_2-N), 2.26 – 2.18 (m, 2H, CH=CH-CH_2), 1.72 – 1.40 (m, 10H, 5 × CH_2); **^{13}C NMR** (101 MHz, $CDCl_3$): δ 194.6, 168.4, 147.9, 146.9, 135.9, 134.0, 133.8, 132.0, 131.6, 131.2, 123.2, 120.2, 118.3, 52.9, 44.0, 37.6, 32.0, 28.3, 26.3, 25.4, 24.3, 24.2; **IR** ν_{max} (film)/cm^{-1} 2940, 2857, 1708, 1660, 1616, 1394, 1296, 1184, 1106, 1043, 911, 826, 718; **HRMS** (ESI$^+$) calculated for $C_{26}H_{28}BrN_4O_3$ [M+H]$^+$: 523.1339, found: 523.1340.

(*E*)-2-[7-(3-Bromophenyl)-7-oxohept-5-en-1-yl]isoindoline-1,3-dione (7e)

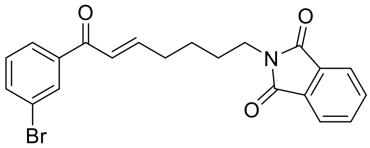

Prepared according to **General Procedure F (ii)**, using aryltriazenyl enone **S4** (80 mg, 0.15 mmol), TfOH (0.05 mL, 0.51 mmol), THF (3.0 mL) and H_2O (38 μL). Purification by flash column chromatography (petrol/Et_2O, 7:3) afforded the title compound (41 mg, 65% yield) as a thick yellow oil.

Bromoaryl enone **7e** was also prepared according to **General Procedure F** in a **sequential manner** without the purification of **S4** prior to the second step, using aldehyde **1a** (43 mg) and 2-(hex-5-yn-1-yl)isoindoline-1,3-dione (68 mg). Purification by flash column chromatography (petrol/Et_2O, 7:3) afforded the title compound (41 mg, 50% yield) as a thick yellow oil.

^{1}H NMR (400 MHz, $CDCl_3$): δ 8.04 (t, *J* = 1.5 Hz, 1H, Ar-*H*), 7.87 – 7.81 (m, 3H, Ar-*H*), 7.74 – 7.64 (m, 3H, Ar-*H*), 7.34 (t, *J* = 8.0 Hz, 1H, Ar-*H*), 7.04 (dt, *J* = 15.5, 7.0 Hz, 1H, CO-CH=C*H*), 6.83 (d, *J* = 15.5 Hz, 1H, CO-C*H*=CH), 3.72 (t, *J* = 7.0 Hz, 2H, CH_2-N), 2.42 – 2.34 (m, 2H, CH=CH-CH_2), 1.80 – 1.71 (m, 2H, CH_2-CH$_2$-N), 1.63 – 1.54 (m, 2H, CH_2); **^{13}C NMR** (101 MHz, $CDCl_3$): δ 189.3, 168.5, 150.0, 139.8, 135.6, 134.1, 132.2, 131.6, 130.2, 127.2, 125.9, 123.3, 123.0, 37.6, 32.4, 28.2, 25.4; **IR**

ν_{max} (film)/cm^{-1} 2938, 2861, 1707, 1670, 1619, 1396, 1370, 1216, 1041, 719; **HRMS** (ESI$^+$) calculated for $C_{21}H_{19}BrNO_3$ [M+H]$^+$: 412.0543, found: 412.0541.

(*E*)-1-{5-Bromo-4-fluoro-2-[(*E*)-piperidin-1-yldiazenyl]phenyl}-4,4-dimethylpent -2-en-1-one (S5)

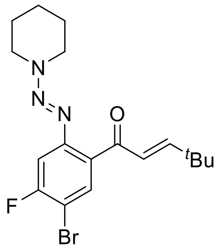

Prepared according to **General Procedure F (i)**, using aldehyde **1e** (47 mg) and 3,3-dimethyl-1-butyne (37 µL). Purification by flash column chromatography (petrol/CH_2Cl_2, 1:1) afforded the title compound (73 mg, 92% yield) as a thick yellow oil. **^{1}H NMR** (400 MHz, $CDCl_3$): δ 7.62 (d, J = 7.5 Hz, 1H, Ar-*H*), 7.27 (d, J = 10.5 Hz, 1H, Ar-*H*), 6.76 (d, J = 16.0 Hz, 1H, CO-CH=C*H*), 6.44 (d, J = 16.0 Hz, 1H, CO-C*H*=CH), 3.76 (br. s, 4H, N(CH_2)$_2$), 1.78 – 1.56 (m, 6H, 3 × CH_2), 1.04 (s, 9H, C(CH_3)$_3$); **^{13}C NMR** (101 MHz, $CDCl_3$): δ 194.1, 160.6 (d, $J_{C\text{-}F}$ = 251.0 Hz), 157.2, 150.2 (d, $J_{C\text{-}F}$ = 7.0 Hz), 133.8 (d, $J_{C\text{-}F}$ = 1.5 Hz), 132.3 (d, $J_{C\text{-}F}$ = 3.0 Hz), 126.4, 105.4 (d, $J_{C\text{-}F}$ = 24.0 Hz), 105.0 (d, $J_{C\text{-}F}$ = 22.5 Hz), 53.2, 44.2, 34.0, 28.9, 26.4, 24.5, 24.2; **^{19}F NMR** (377 MHz, $CDCl_3$): δ −103.4; **IR** ν_{max} (film)/cm^{-1} 2948, 2860, 1663, 1613, 1418, 1381, 1340, 1292, 1249, 1202, 1146, 1108, 983, 853; **HRMS** (ESI$^+$) calculated for $C_{18}H_{24}BrFN_3O$ [M+H]$^+$: 396.1081, found: 396.1077.

(*E*)-1-(3-Bromo-4-fluorophenyl)-4,4-dimethylpent-2-en-1-one (7f)

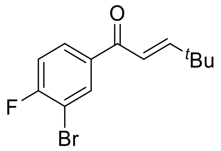

Prepared according to **General Procedure F (ii)**, using aryltriazenyl enone **S5** (70 mg, 0.18 mmol), TfOH (0.05 mL, 0.61 mmol), THF (3.6 mL) and H_2O (44 µL). Purification by flash column chromatography (petrol/CH_2Cl_2, 4:1) afforded the title compound (42 mg, 82% yield) as a yellow oil.

Bromoaryl enone **7f** was also prepared according to **General Procedure F** in a **sequential manner** without the purification of **S5** prior to the second step, using aldehyde **1e** (47 mg) and 3,3-dimethyl-1-butyne (37 µL). Purification by flash column chromatography (petrol/CH_2Cl_2, 4:1) afforded the title compound (41 mg, 73% yield) as a yellow oil.

^{1}H NMR (400 MHz, $CDCl_3$): δ 8.14 (d, J = 6.5 Hz, 1H, Ar-*H*), 7.91-7.85 (m, 1H, Ar-*H*), 7.20 (t, J = 8.5 Hz, 1H, Ar-*H*), 7.09 (d, J = 15.5 Hz, 1H, CO-CH=C*H*), 6.70 (d, J = 15.5 Hz, 1H, CO-C*H*=CH), 1.15 (s, 9H, C(CH_3)$_3$); **^{13}C NMR** (101 MHz, $CDCl_3$): δ 188.6, 161.8 (d, $J_{C\text{-}F}$ = 255.0 Hz), 160.9, 135.8 (d, $J_{C\text{-}F}$ = 3.5 Hz), 134.4 (d, $J_{C\text{-}F}$ = 1.0 Hz), 129.8 (d, $J_{C\text{-}F}$ = 8.5 Hz), 120.1, 116.7 (d, $J_{C\text{-}F}$ = 23.0 Hz),

109.7 (d, $J_{C\text{-}F}$ = 21.5 Hz), 34.5, 28.8; **^{19}F NMR** (377 MHz, $CDCl_3$): δ −100.4; **IR** ν_{max} (film)/cm^{-1} 2962, 2904, 2868, 1672, 1618, 1590, 1491, 1396, 1303, 1260, 1244, 1207, 1049, 984, 826, 732, 671, 615; **HRMS** (ESI$^+$) calculated for $C_{13}H_{15}BrFO$ [M+H]$^+$: 285.0285, found: 285.0286.

(*E*)-1-{5-Bromo-4-chloro-2-[(*E*)-piperidin-1-yldiazenyl]phenyl}-4,4-dimethylpent-2-en-1-one (S6)

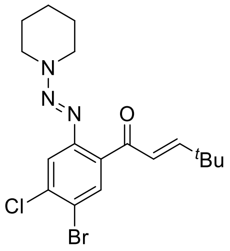

Prepared according to **General Procedure F (i)**, using aldehyde **1c** (50 mg) and 3,3-dimethyl-1-butyne (37 µL). Purification by flash column chromatography (petrol/CH_2Cl_2, 1:1) afforded the title compound (74 mg, 90% yield) as an off-white solid. **m.p.** (Et_2O): 102 – 104 ºC; **^{1}H NMR** (400 MHz, $CDCl_3$): δ 7.65 (s, 1H, Ar-*H*), 7.60 (s, 1H, Ar-*H*), 6.74 (d, *J* = 16.0 Hz, 1H, CO-CH=C*H*), 6.40 (d, *J* = 16.0 Hz, 1H, CO-C*H*=CH), 3.78 – 3.74 (m, 4H, N(CH_2)$_2$), 1.78 – 1.56 (m, 6H, 3 × CH_2), 1.05 (s, 9H, C(CH_3)$_3$); **^{13}C NMR** (101 MHz, $CDCl_3$): δ 194.3, 157.7, 148.8, 136.9, 134.5, 133.6, 126.3, 119.7, 118.1, 53.1, 44.1, 34.0, 28.8, 26.4, 24.4, 24.2; **IR** ν_{max} (film)/cm^{-1} 2941, 2858, 1724, 1666, 1614, 1573, 1416, 1354, 1339, 1293, 1265, 1203, 1108, 729; **HRMS** (ESI$^+$) calculated for $C_{18}H_{24}BrClN_3O$ [M+H]$^+$: 412.0786, found: 412.0782.

(*E*)-1-(3-Bromo-4-chlorophenyl)-4,4-dimethylpent-2-en-1-one (7g)

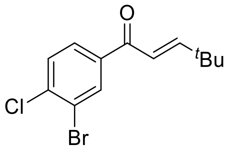

Prepared according to **General Procedure F (ii)**, using aryltriazenyl enone **S6** (70 mg, 0.17 mmol), TfOH (0.05 mL, 0.58 mmol), THF (3.4 mL) and H_2O (43 µL). Purification by flash column chromatography (petrol/CH_2Cl_2, 4:1) afforded the title compound (41 mg, 79% yield) as a yellow oil.

Bromoaryl enone **7g** was also prepared according to **General Procedure F** in a **sequential manner** without the purification of **S6** prior to the second step, using aldehyde **1c** (50 mg) and 3,3-dimethyl-1-butyne (37 µL). Purification by flash column chromatography (petrol/CH_2Cl_2, 4:1) afforded the title compound (42 mg, 71% yield) as a yellow oil.

^{1}H NMR (400 MHz, $CDCl_3$): δ 8.15 (s, 1H, Ar-*H*), 7.79 (d, *J* = 8.5 Hz, 1H, Ar-*H*), 7.54 (d, *J* = 8.5 Hz, 1H, Ar-*H*), 7.09 (d, *J* = 15.5 Hz, 1H, CO-CH=C*H*), 6.69 (d, *J* = 15.5 Hz, 1H, CO-C*H*=CH), 1.15 (s, 9H, C(CH_3)$_3$); **^{13}C NMR** (101 MHz, $CDCl_3$): δ 189.0, 161.2, 139.1, 137.9, 133.9, 130.6, 128.4, 123.1, 120.1, 34.5, 28.8; **IR** ν_{max} (film)/cm^{-1} 2962, 2904, 2867, 1671, 1617, 1583, 1552, 1462, 1369, 1302, 1273,

1212, 1125, 1022, 984, 860, 825, 728, 712, 661; **HRMS** (ESI$^+$) calculated for $C_{13}H_{15}BrClO$ [M+H]$^+$: 300.9989, found: 300.9991.

(*E*)-1-{5-Bromo-4-methyl-2-[(*E*)-piperidin-1-yldiazenyl]phenyl}-3-cyclopentyl prop-2-en-1-one (S7)

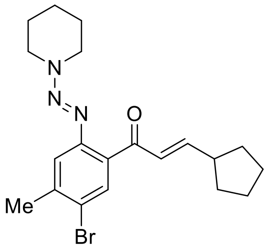

Prepared according to **General Procedure F (i)**, using aldehyde **1f** (46 mg) and ethynylcyclopentane (35 μL). Purification by flash column chromatography (petrol/CH_2Cl_2, 1:1) afforded the title compound (66 mg, 81% yield) as a yellow oil. **^{1}H NMR** (400 MHz, $CDCl_3$): δ 7.59 (s, 1H, Ar-*H*), 7.33 (s, 1H, Ar-*H*), 6.68 (dd, *J* = 15.5, 8.0 Hz, 1H, CO-CH=C*H*), 6.43 (d, *J* = 15.5 Hz, 1H, CO-C*H*=CH), 3.73 (br. s, 4H, N(C*H*$_2$)$_2$), 2.62 – 2.50 (m, 1H, CH=CH-C*H*), 2.39 (s, 3H, C*H*$_3$), 1.84 – 1.51 (m, 12H, 6 × C*H*$_2$), 1.41 – 1.30 (m, 2H, Cypent-*H*); **^{13}C NMR** (101 MHz, $CDCl_3$): δ 194.6, 151.6, 148.1, 140.9, 133.8, 132.4, 129.5, 121.2, 120.6, 52.7, 44.2, 43.2, 32.6, 26.3, 25.4, 24.5, 24.3, 23.2; **IR** ν_{max} (film)/cm^{-1} 2941, 2858, 1661, 1614, 1418, 1353, 1292, 1255, 1191, 1149, 1105, 981, 892, 852; **HRMS** (ESI$^+$) calculated for $C_{20}H_{27}BrN_3O$ [M+H]$^+$: 404.1332, found: 404.1327.

(*E*)-1-(3-Bromo-4-methylphenyl)-3-cyclopentylprop-2-en-1-one (7h)

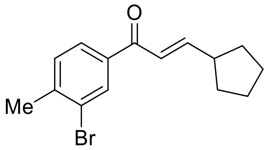

Prepared according to **General Procedure F (ii)**, using aryltriazenyl enone **S7** (66 mg, 0.16 mmol), TfOH (0.05 mL, 0.54 mmol), THF (3.2 mL) and H_2O (40 μL). Purification by flash column chromatography (petrol/Et_2O, 95:5) afforded the title compound (33 mg, 70% yield) as a yellow oil.

Bromoaryl enone **7h** was also prepared according to **General Procedure F** in a **sequential manner** without the purification of **S7** prior to the second step, using aldehyde **1f** (46 mg) and ethynylcyclopentane (35 μL). Purification by flash column chromatography (petrol/Et_2O, 95:5) afforded the title compound (31 mg, 53% yield) as a yellow oil.

^{1}H NMR (400 MHz, $CDCl_3$): δ 8.08 (d, *J* = 1.5 Hz, 1H, Ar-*H*), 7.76 (dd, *J* = 8.0, 1.5 Hz, 1H, Ar-*H*), 7.31 (d, *J* = 8.0 Hz, 1H, Ar-*H*), 7.05 (dd, *J* = 15.5, 8.0 Hz, 1H, CO-CH=C*H*), 6.80 (d, *J* = 15.5 Hz, 1H, CO-C*H*=CH), 2.76 – 2.65 (m, 1H, CH=CH-C*H*), 2.45 (s, 3H, C*H*$_3$), 1.94 – 1.85 (m, 2H, Cypent-*H*), 1.78 – 1.58 (m, 4H, Cypent-*H*), 1.52 – 1.41 (m, 2H, Cypent-*H*); **^{13}C NMR** (101 MHz, $CDCl_3$): δ 189.3,

154.9, 143.1, 137.6, 132.6, 130.9, 127.4, 125.3, 123.5, 43.6, 32.8, 25.5, 23.3; **IR** ν_{max} (film)/cm^{-1} 2953, 2867, 1668, 1617, 1554, 1450, 1381, 1296, 1255, 1208, 1038, 983, 821, 723, 674; **HRMS** (ESI$^+$) calculated for $C_{15}H_{18}BrO$ [M+H]$^+$: 293.0536, found 293.0536.

(*E*)-1-(3-Bromo-2-fluorophenyl)-4,4-dimethylpent-2-en-1-one (7i)

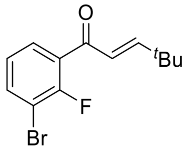

Prepared according to a modified **General Procedure F**, using aldehyde **1k** (47 mg) and 3,3-dimethyl-1-butyne (37 µL). The bromination was carried out at 40 ºC. The purification after the bromination step was inefficient due to an inseparable impurity, therefore, the impure crude was used for the second step. Purification by flash column chromatography (petrol/CH_2Cl_2, 7:3) afforded the title compound (33 mg, 58% yield) as a yellow oil. **^{1}H NMR** (400 MHz, $CDCl_3$): δ 7.72 – 7.67 (m, 1H, Ar-*H*), 7.63 – 7.58 (m, 1H, Ar-*H*), 7.11 (t, *J* = 8.0 Hz, 1H, Ar-*H*), 6.97 (dd, *J* = 16.0, 1.5 Hz, 1H, CO-CH=C*H*), 6.59 (dd, *J* = 16.0, 3.0 Hz, 1H, CO-C*H*=CH), 1.13 (s, 9H, C(C*H*$_3$)$_3$); **^{13}C NMR** (101 MHz, $CDCl_3$): δ 189.6 (d, $J_{C\text{-}F}$ = 3.0 Hz), 161.0, 157.2 (d, $J_{C\text{-}F}$ = 253.0 Hz), 136.9, 130.0 (d, $J_{C\text{-}F}$ = 2.5 Hz), 128.8 (d, $J_{C\text{-}F}$ = 15.0 Hz), 125.4 (d, $J_{C\text{-}F}$ = 4.5 Hz), 124.6 (d, $J_{C\text{-}F}$ = 5.5 Hz), 110.3 (d, $J_{C\text{-}F}$ = 22.5 Hz), 34.4, 28.7; **^{19}F NMR** (377 MHz, $CDCl_3$): δ −105.1; **IR** ν_{max} (film)/cm^{-1} 2962, 2868, 1671, 1616, 1445, 1302, 1243, 1135, 986, 737; **HRMS** (ESI$^+$) calculated for $C_{13}H_{15}BrFO$ [M+H]$^+$: 285.0285, found: 285.0286.

(*E*)-1-{5-Bromo-3-fluoro-2-[(*E*)-piperidin-1-yldiazenyl]phenyl}-4,4-dimethylpent -2-en-1-one (S8)

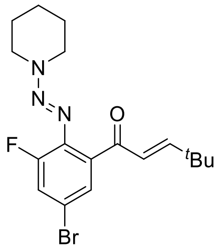

Prepared according to a modified **General Procedure F (i)**, using aldehyde **1b** (47 mg) and 3,3-dimethyl-1-butyne (37 µL) at 40 ºC. Purification by flash column chromatography (petrol/CH_2Cl_2, 1:1) afforded the title compound (63 mg, 80% yield) as a yellow solid. **m.p.** (Et_2O): 53 – 55 ºC; **^{1}H NMR** (400 MHz, $CDCl_3$): δ 7.32 (d, *J* = 10.0 Hz, 1H, Ar-*H*), 7.24 (s, 1H, Ar-*H*), 6.62 (d, *J* = 16.0 Hz, 1H, CO-CH=C*H*), 5.97 (d, *J* = 16.0 Hz, 1H, CO-C*H*=CH), 3.86 – 3.60 (2 × br. s, 4H, N(C*H*$_2$)$_2$), 1.67 (br. s, 6H, 3 × C*H*$_2$), 1.01 (s, 9H, C(C*H*$_3$)$_3$); **^{13}C NMR** (101 MHz, $CDCl_3$): δ 194.0 (d, $J_{C\text{-}F}$ = 2.5 Hz), 157.4, 155.6 (d, $J_{C\text{-}F}$ = 255.5 Hz), 137.5 (d, $J_{C\text{-}F}$ = 9.0 Hz), 136.0, 127.2 (d, $J_{C\text{-}F}$ = 3.5 Hz), 125.6, 121.1 (d, $J_{C\text{-}F}$ = 23.5 Hz), 117.4 (d, $J_{C\text{-}F}$ = 9.0 Hz), 52.8, 44.0, 33.9, 28.8, 26.4, 24.4, 24.1; **^{19}F NMR** (377 MHz, $CDCl_3$): δ −123.1; **IR** ν_{max} (film)/cm^{-1} 2945, 2861, 1662, 1620, 1565, 1420, 1339, 1297, 1255, 1183,

1108, 985, 954, 922, 891, 853, 756, 732, 690; **HRMS** (ESI$^+$) calculated for $C_{18}H_{24}BrFN_3O$ [M+H]$^+$: 396.1081, found: 396.1086.

(*E*)-1-(3-Bromo-5-fluorophenyl)-4,4-dimethylpent-2-en-1-one, (7j)

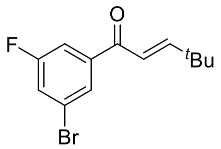

Prepared according to **General Procedure F (ii)**, using aryltriazenyl enone **S8** (52 mg, 0.13 mmol), TfOH (0.04 mL, 0.44 mmol), THF (2.6 mL) and H_2O (33 μL). Purification by flash column chromatography (petrol/CH_2Cl_2, 4:1) afforded the title compound (10 mg, 26% yield) as a yellow oil. **^{1}H NMR** (400 MHz, $CDCl_3$): δ 7.82 (s, 1H, Ar-*H*), 7.54 (d, J = 9.0 Hz, 1H, Ar-*H*), 7.43 (d, J = 7.5 Hz, 1H, Ar-*H*), 7.11 (d, J = 15.5 Hz, 1H, CO-CH=C*H*), 6.67 (d, J = 15.5 Hz, 1H, CO-C*H*=CH), 1.16 (s, 9H, C(C*H*$_3$)$_3$); **^{13}C NMR** (101 MHz, $CDCl_3$): δ 188.7 (d, $J_{C\text{-}F}$ = 2.0 Hz), 162.7 (d, $J_{C\text{-}F}$ = 253.0 Hz), 161.7, 141.4 (d, $J_{C\text{-}F}$ = 6.5 Hz), 127.6 (d, $J_{C\text{-}F}$ = 3.0 Hz), 123.09 (d, $J_{C\text{-}F}$ = 9.0 Hz), 123.08 (d, $J_{C\text{-}F}$ = 24.5 Hz), 120.1, 114.5 (d, $J_{C\text{-}F}$ = 22.0 Hz), 34.6, 28.8; **^{19}F NMR** (377 MHz, $CDCl_3$): δ −109.4; **IR** ν_{max} (film)/cm^{-1} 2963, 2869, 1674, 1619, 1578, 1427, 1303, 1162, 984, 925, 839, 694; **HRMS** (ESI$^+$) calculated for $C_{13}H_{15}BrFO$ [M+H]$^+$: 285.0285, found: 285.0286.

(*E*)-1-{5-Chloro-4-fluoro-2-[(*E*)-piperidin-1-yldiazenyl]phenyl}-4,4-dimethylpent -2-en-1-one (S9)

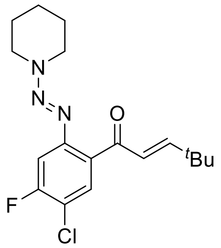

Prepared according to a modified **General Procedure F (i)**, using aldehyde **1e** (47 mg), 3,3-dimethyl-1-butyne (37 μL, 0.30 mmol), 1-chloro-1,2-benziodoxol-3-one (113.0 mg, 0.40 mmol, 2.0 equiv) in replacement of NBS, and at 40 ºC. After completion, the mixture was diluted with 5% aqueous solution of LiCl and extracted with CH_2Cl_2. The combined organic layers were concentrated under reduced pressure and purified by flash column chromatography (petrol/CH_2Cl_2, 3:2) to afford the title compound (56 mg, 79% yield) as a yellow oil. **^{1}H NMR** (400 MHz, $CDCl_3$): δ 7.49 (d, J = 8.0 Hz, 1H, Ar-*H*), 7.31 (d, J = 10.5 Hz, 1H, Ar-*H*), 6.77 (d, J = 16.0 Hz, 1H, CO-CH=C*H*), 6.45 (d, J = 16.0 Hz, 1H, CO-C*H*=CH), 3.77 (br. s, 4H, N(C*H*$_2$)$_2$), 1.82 – 1.56 (m, 6H, 3 × C*H*$_2$), 1.06 (s, 9H, C(C*H*$_3$)$_3$); **^{13}C NMR** (101 MHz, $CDCl_3$): δ 194.2, 159.6 (d, $J_{C\text{-}F}$ = 252.5 Hz), 157.3, 149.4 (d, $J_{C\text{-}F}$ = 6.5 Hz), 131.9 (d, $J_{C\text{-}F}$ = 3.0 Hz), 131.0, 126.4, 117.4 (d, $J_{C\text{-}F}$ = 19.0 Hz), 105.6 (d, $J_{C\text{-}F}$ = 23.0 Hz), 53.2, 44.3, 34.0, 28.9, 26.5, 24.5, 24.2; **^{19}F NMR** (377 MHz, $CDCl_3$): δ −111.3; **IR** ν_{max} (film)/cm^{-1} 2943, 2860, 1664, 1613, 1478,

1420, 1384, 1295, 1252, 1202, 1147, 1108, 1065, 983, 853, 743, 693; **HRMS** (ESI^+) calculated for $C_{18}H_{24}ClFN_3O$ $[M+H]^+$: 352.1586, found: 352.1588.

(*E*)-1-(3-Chloro-4-fluorophenyl)-4,4-dimethylpent-2-en-1-one (7k)

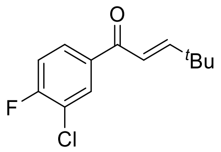

Prepared according to **General Procedure F (ii)**, using aryltriazenyl enone **S9** (56 mg, 0.16 mmol), TfOH (0.05 mL, 0.54 mmol), THF (3.2 mL) and H_2O (40 μL). Purification by flash column chromatography (petrol/CH_2Cl_2, 7:3) afforded the title compound (24 mg, 63% yield) as a yellow oil. **^{1}H NMR** (400 MHz, $CDCl_3$): δ 8.00 (dd, J = 7.0, 1.5 Hz, 1H, Ar-*H*), 7.86-7.81 (m, 1H, Ar-*H*), 7.22 (t, J = 8.5 Hz, 1H, Ar-*H*), 7.09 (d, J = 15.5 Hz, 1H, CO-CH=C*H*), 6.71 (d, J = 15.5 Hz, 1H, CO-C*H*=CH), 1.16 (s, 9H, C(C*H*$_3$)$_3$); **^{13}C NMR** (101 MHz, $CDCl_3$): δ 188.7, 160.89, 160.88 (d, J_{C-F} = 256.5 Hz), 135.4 (d, J_{C-F} = 3.5 Hz), 131.5, 129.0 (d, J_{C-F} = 8.5 Hz), 121.8 (d, J_{C-F} = 18.0 Hz), 120.1, 116.8 (d, J_{C-F} = 21.5 Hz), 34.5, 28.8; **^{19}F NMR** (377 MHz, $CDCl_3$): δ −108.4; **IR** ν_{max} (film)/cm^{-1} 2963, 2869, 1715, 1673, 1617, 1592, 1496, 1397, 1365, 1304, 1264, 1246, 1221, 1208, 1064, 984, 826, 732, 694, 616; **HRMS** (ESI^+) $C_{13}H_{15}ClFO$ $[M+H]^+$: 241.0790, found: 241.0792.

VIII. Modification of the Triazene Group

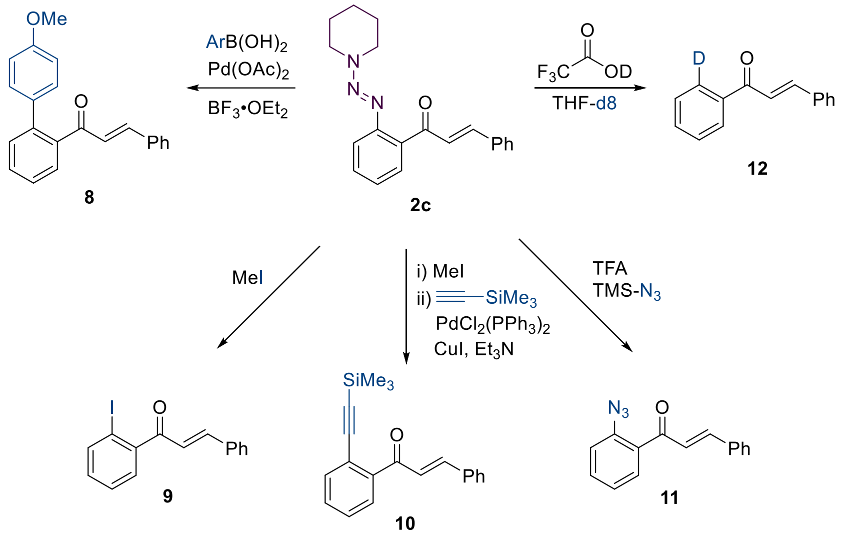

(*E*)-1-(4'-Methoxy-[1,1'-biphenyl]-2-yl)-3-phenylprop-2-en-1-one (8)

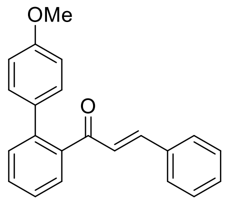

A reaction vial charged with aryltriazenyl enone **2c** (47.9 mg, 0.15 mmol), Pd(OAc)$_2$ (1.7 mg, 0.0075 mmol) and 4-methoxyphenylboronic acid (45.6 mg, 0.30 mmol) was evacuated and back-filled with N_2 gas for 3 times. 1,4-Dioxane (1.5 mL) and BF_3·OEt_2 (0.02 mL, 0.15 mmol) were added and the reaction mixture was stirred at 23 ºC for 24 h. Upon completion, the reaction was concentrated under reduced pressure and purified by flash column chromatography (petrol/Et_2O, 9:1) to afford the title compound (42 mg, 88% yield) as a yellow oil. **^{1}H NMR** (400 MHz, $CDCl_3$): δ 7.55 (d, J = 7.5 Hz, 1H, Ar-*H*), 7.49 – 7.43 (m, 1H, Ar-*H*), 7.39 – 7.32 (m, 2H, Ar-*H*), 7.29 (d, J = 16.0 Hz, 1H, CO-CH=C*H*), 7.25 – 7.14 (m, 7H, Ar-*H*), 6.81 (d, J = 8.0 Hz, 2H, Ar-*H*), 6.47 (d, J = 16.0 Hz, 1H, CO-C*H*=CH), 3.67 (s, 3H, C*H*$_3$); **^{13}C NMR** (101 MHz, $CDCl_3$): δ 196.6, 159.5, 143.5, 140.7, 139.8, 134.9, 132.9, 130.8, 130.5, 130.4, 130.2, 128.9, 128.8, 128.3, 127.1, 127.0, 114.2, 55.4; **IR** ν_{max} (film)/cm^{-1} 3059, 2836, 1664, 1643, 1607, 1575, 1516, 1475, 1448, 1330, 1298, 1245, 1206, 1178, 1034, 978, 834, 763; **HRMS** (ESI$^+$) calculated for $C_{22}H_{19}O_2$ [M+H]$^+$: 315.1380, found: 315.1381.

(*E*)-1-(2-Iodophenyl)-3-phenylprop-2-en-1-one (9)

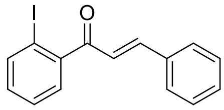

In a sealed reaction vial under N_2 atmosphere, a solution of aryltriazenyl enone **2c** (31.9 mg, 0.1 mmol) in MeI (1 mL) was refluxed at 100 ºC for 16 h. The mixture was concentrated under reduced pressure and purified by flash column chromatography (petrol/Et_2O, 92:8) to afford the title compound (25 mg, 75% yield) as a yellow oil. **^{1}H NMR** (400 MHz, $CDCl_3$): δ 7.94 (d, J = 8.0 Hz, 1H, Ar-*H*), 7.59 – 7.54 (m, 2H, Ar-*H*), 7.48 – 7.36 (m, 6H, 5 × Ar-*H* and CO-CH=C*H*), 7.17 (td, J = 8.0, 1.5 Hz, 1H, Ar-*H*), 7.08 (d, J = 16.0 Hz, 1H, CO-C*H*=CH); **^{13}C NMR** (101 MHz, $CDCl_3$): δ 196.2, 147.2, 144.8, 140.1, 134.5, 131.4, 131.1, 129.1, 128.7, 128.6, 128.1, 125.7, 92.3; **IR** ν_{max} (film)/cm^{-1} 3058, 2981, 1644, 1602, 1449, 1331, 1285, 1210, 1011, 753; data consistent with those reported in literature.[12]

(*E*)-3-Phenyl-1-{2-[(trimethylsilyl)ethynyl]phenyl}prop-2-en-1-one (10)

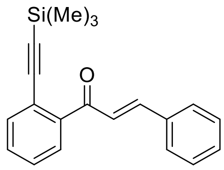

In a sealed reaction vial under N_2 atmosphere, a solution of aryltriazenyl enone **2c** (31.9 mg, 0.1 mmol) in MeI (1 mL) was refluxed at 100 ºC for 16 h. The mixture was concentrated under reduced pressure and back-filled with N_2 gas. To this was added $PdCl_2(PPh_3)_2$ (7.0 mg, 0.01 mmol), CuI (1.9 mg, 0.01 mmol), ethynyltrimethylsilane (28 µL, 0.2 mmol) and NEt_3 (0.5 mL), and the mixture was stirred at 23 ºC for 24 h. It was then concentrated under reduced pressure and purified by flash column chromatography (petrol/CH_2Cl_2, 3:2) to afford the title compound (22 mg, 73% yield) as a yellow oil. **^{1}H NMR** (400 MHz, $CDCl_3$): δ 7.65 – 7.56 (m, 5H, 4 × Ar-*H* and CO-CH=C*H*), 7.49 – 7.37 (m, 6H, 5 × Ar-*H* and CO-C*H*=CH), 0.08 (s, 9H, Si(C*H*$_3$)$_3$); **^{13}C NMR** (101 MHz, $CDCl_3$): δ 194.1, 144.3, 142.7, 135.0, 133.7, 130.8, 130.7, 129.0, 128.8, 128.7 (2C), 126.0, 121.4, 103.1, 101.5, –0.2; **IR** ν_{max} (film)/cm^{-1} 3060, 2981, 2159, 1643, 1607, 1575, 1478, 1448, 1331, 1287, 1206, 1178, 1034, 690; **HRMS** (ESI$^+$) calculated for $C_{20}H_{21}OSi$ [M+H]$^+$: 305.1356, found: 305.1356.

(*E*)-1-(2-Azidophenyl)-3-phenylprop-2-en-1-one (11)

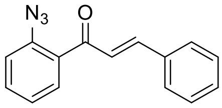

Under N_2 atmosphere, azidotrimethylsilane (66 μL, 0.5 mmol) and trifluoroacetic acid (0.06 mL, 0.8 mmol) were added to a solution of aryltriazenyl enone **2c** (31.9 mg, 0.1 mmol) in CH_2Cl_2 (0.5 mL) at 0 ºC. The reaction mixture was stirred at 23 ºC for 1 h and concentrated under reduced pressure. Purification by flash column chromatography (petrol/Et_2O, 92:8) afforded the title compound (24 mg, 96% yield) as a yellow oil. **^{1}H NMR** (400 MHz, $CDCl_3$): δ 7.56-7.44 (m, 5H, 4 × Ar-*H* and CO-CH=C*H*), 7.37-7.31 (m, 3H, Ar-*H*), 7.22-7.14 (m, 3H, 2 × Ar-*H* and CO-C*H*=CH); **^{13}C NMR** (101 MHz, $CDCl_3$): δ 192.8, 145.2, 138.4, 134.7, 132.4, 132.0, 130.8, 130.2, 129.1, 128.7, 126.4, 124.9, 119.2; **IR** ν_{max} (film)/cm^{-1} 3060, 2981, 2123, 1661, 1646, 1599, 1575, 1480, 1448, 1332, 1287, 1207, 1021, 978, 754, 705; **HRMS** (ESI$^+$) calculated for $C_{15}H_{11}N_3ONa$ [M+Na]$^+$: 272.0794, found: 272.0795.

(*E*)-1-(2-Deuteriophenyl)-3-phenylprop-2-en-1-one (12)

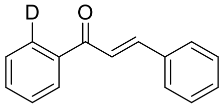

Trifluoroacetic acid-*d* (0.10 mL) was added to a solution of aryltriazenyl enone **2c** (31.9 mg, 0.10 mmol) in THF-d_8 (2 mL) and the mixture was stirred at 40 ºC for 16 h. It was then concentrated under reduced pressure and purified by flash column chromatography (petrol/Et_2O, 92:8) to afford the title compound (8 mg, 40% yield) as a yellow oil. **^{1}H NMR** (400 MHz, $CDCl_3$): δ 8.03 (d, J = 8.0 Hz, 1H, Ar-*H*), 7.82 (d, J = 16.0 Hz, 1H, CO-CH=C*H*), 7.69 – 7.63 (m, 2H, Ar-*H*), 7.62 – 7.48 (m, 4H, 3 × Ar-*H* and CO-C*H*=CH), 7.46 – 7.39 (m, 3H, Ar-*H*); **^{13}C NMR** (101 MHz, $CDCl_3$): δ 190.7, 145.0, 138.2, 135.0, 132.9, 130.7, 129.1, 128.8, 128.7, 128.63, 128.60 (2C, one singlet and one multiplet overlap), 122.2; **IR** ν_{max} (film)/cm^{-1} 3060, 2921, 1663, 1604, 1575, 1449, 1334, 1214, 1018, 745; **HRMS** (ESI$^+$) calculated for $C_{15}H_{12}DO$ [M+H]$^+$: 210.1024, found: 210.1024.

IX. Sequential Functionalizations

1. Sequential Hydroacylation / Detriazenative Arylation

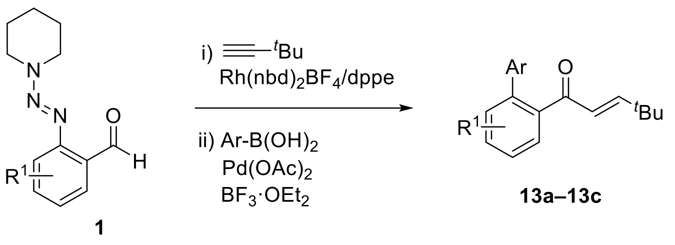

General Procedure G: A reaction vial charged with $Rh(nbd)_2BF_4$ (3.7 mg, 0.01 mmol, 5 mol%) and dppe (4.0 mg, 0.01 mmol, 5 mol%) was evacuated and back-filled with N_2 gas for 3 times, and to this was added CH_2Cl_2 (0.25 mL). The resulting solution was bubbled with H_2 gas for 2 min and then with N_2 gas until the volume of the solution had become around 0.1 mL. This solution was then transferred to a N_2-purged reaction vial containing aldehyde (0.2 mmol, 1.0 equiv), with the use of additional 0.1 mL of CH_2Cl_2. Alkyne (0.30 mmol, 1.5 equiv) was added and the reaction mixture was stirred at 23 ºC for 16 h. To this was added arylboronic acid (0.40 mmol, 2.0 equiv) and $Pd(OAc)_2$ (2.2 mg, 0.01 mmol, 5 mol%). The flask was then evacuated and back-filled with N_2 gas, followed by the addition of 1,4-dioxane (2 mL, 0.1 M) and $BF_3·OEt_2$ (0.05 mL, 0.40 mmol, 2.0 equiv). The reaction mixture was stirred at 23 ºC for 24 h, filtered through a pad of silica and concentrated under reduced pressure. Purification by flash column chromatography afforded the corresponding product.

(*E*)-1-(6-Fluoro-4'-methyl-[1,1'-biphenyl]-2-yl)-4,4-dimethylpent-2-en-1-one (13a)

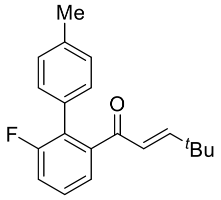

Prepared according to **General Procedure G**, using aldehyde **1b** (47 mg), 3,3-dimethyl-1-butyne (37 µL) and *p*-tolylboronic acid (54 mg). Purification by flash column chromatography (gradient petrol/CH_2Cl_2, 7:3 to 6:4) afforded the title compound (47 mg, 79% yield) as a yellow oil. **^{1}H NMR** (400 MHz, $CDCl_3$): δ 7.44 – 7.34 (m, 2H, Ar-*H*), 7.31 – 7.24 (m, 1H, Ar-*H*), 7.21 (app. s, 4H, Ar-*H*), 6.55 (d, *J* = 16.0 Hz, 1H, CO-CH=C*H*), 5.76 (d, *J* = 16.0 Hz, 1H, CO-C*H*=CH), 2.39 (s, 3H, Ar-C*H*$_3$), 0.83 (s, 9H, C(C*H*$_3$)$_3$); **^{13}C NMR** (101 MHz, $CDCl_3$): δ 196.2 (d, $J_{C\text{-}F}$ = 3.0 Hz), 159.4 (d, $J_{C\text{-}F}$ = 247.5 Hz), 158.8, 142.7 (d, $J_{C\text{-}F}$ = 2.0 Hz), 138.3, 130.5, 130.3 (d, $J_{C\text{-}F}$ = 1.5 Hz), 129.1, 128.9 (d, $J_{C\text{-}F}$ = 8.5 Hz), 128.1 (d, $J_{C\text{-}F}$ = 16.5 Hz), 125.6, 124.3 (d, $J_{C\text{-}F}$ = 3.5 Hz), 117.9 (d, $J_{C\text{-}F}$ = 23.5 Hz), 33.8, 28.5, 21.3; **^{19}F NMR** (377 MHz, $CDCl_3$): δ –116.7; **IR** ν_{max} (film)/cm^{-1} 2961, 2867, 1672, 1654, 1616, 1451, 1300, 1244, 1060, 983, 817, 748; **HRMS** (ESI$^+$) calculated for $C_{20}H_{22}FO$ [M+H]$^+$: 297.1649, found: 297.1651.

(*E*)-1-(5-Chloro-2'-methyl-[1,1'-biphenyl]-2-yl)-4,4-dimethylpent-2-en-1-one (13b)

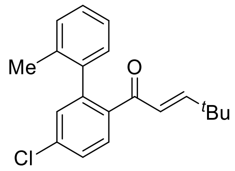

Prepared according to **General Procedure G**, using aldehyde **1c** (50 mg), 3,3-dimethyl-1-butyne (37 μL) and *o*-tolylboronic acid (54 mg). Purification by flash column chromatography (petrol/Et_2O, 94:6) afforded the title compound (55 mg, 88% yield) as a yellow oil. **^{1}H NMR** (400 MHz, $CDCl_3$): δ 7.58 (d, J = 8.5 Hz, 1H, Ar-H), 7.39 (dd, J = 8.5, 2.0 Hz, 1H, Ar-H), 7.27 – 7.21 (m, 2H, Ar-H), 7.20 – 7.13 (m, 2H, Ar-H), 7.07 (d, J = 7.5 Hz, 1H, Ar-H), 6.59 (d, J = 15.5 Hz, 1H, CO-CH=CH), 5.74 (d, J = 15.5 Hz, 1H, CO-CH=CH), 2.11 (s, 3H, Ar-CH_3), 0.78 (s, 9H, C(CH_3)$_3$); **^{13}C NMR** (101 MHz, $CDCl_3$): δ 195.0, 158.0, 142.3, 139.3, 138.8, 136.6, 135.8, 130.6, 130.5, 130.4, 130.0, 128.5, 127.7, 126.0, 124.6, 33.7, 28.5, 20.2; **IR** ν_{max} (film)/cm^{-1} 2961, 2867, 1669, 1617, 1587, 1554, 1463, 1389, 1365, 1297, 1094, 1017, 983, 851, 826, 766, 730; **HRMS** (ESI$^+$) calculated for $C_{20}H_{22}ClO$ [M+H]$^+$: 313.1354, found: 313.1354.

(*E*)-1-(3'-Chloro-3-fluoro-4'-methoxy-[1,1'-biphenyl]-2-yl)-4,4-dimethylpent-2-en-1-one (13c)

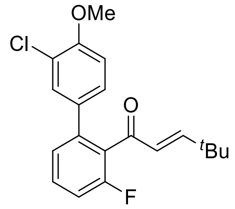

Prepared according to **General Procedure G**, using aldehyde **1k** (47 mg), 3,3-dimethyl-1-butyne (37 μL) and 3-chloro-4-methoxyphenylboronic acid (75 mg). Purification by flash column chromatography (petrol/Et_2O, 9:1) afforded the title compound (68 mg, 98% yield) as a colorless oil. **^{1}H NMR** (400 MHz, $CDCl_3$): δ 7.46 – 7.39 (m, 1H, Ar-H), 7.31 (d, J = 2.0 Hz, 1H, Ar-H), 7.17 – 7.08 (m, 3H, Ar-H), 6.88 (d, J = 8.5 Hz, 1H, Ar-H), 6.43 (d, J = 16.0 Hz, 1H, CO-CH=CH), 5.97 (d, J = 16.0 Hz, 1H, CO-CH=CH), 3.89 (s, 3H, OCH_3), 0.90 (s, 9H, C(CH_3)$_3$); **^{13}C NMR** (101 MHz, $CDCl_3$): δ 194.5, 162.2, 159.4 (d, J_{C-F} = 249.0 Hz), 154.9, 140.7 (d, J_{C-F} = 4.0 Hz), 132.5 (d, J_{C-F} = 2.5 Hz), 130.9 (d, J_{C-F} = 9.0 Hz), 130.6, 128.5, 127.5 (d, J_{C-F} = 17.5 Hz), 127.3, 125.4 (d, J_{C-F} = 3.0 Hz), 122.6, 114.9 (d, J_{C-F} = 22.0 Hz), 111.9, 56.3, 34.1, 28.5; **^{19}F NMR** (377 MHz, $CDCl_3$): δ –115.7; **IR** ν_{max} (film)/cm^{-1} 2962, 1657, 1608, 1508, 1460, 1290, 1253, 1236, 1105, 1066, 1023, 984, 925, 800, 755, 705; **HRMS** (ESI$^+$) calculated for $C_{20}H_{21}ClFO_2$ [M+H]$^+$: 347.1209, found: 347.1208.

2. One-pot Hydroacylation / 1,4-Addition, Followed by Detriazenative Arylation

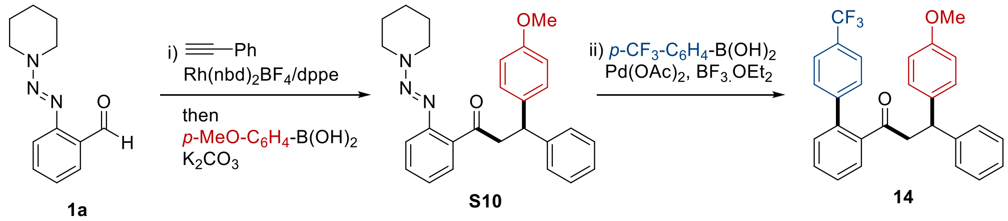

(*E*)-3-(4-Methoxyphenyl)-3-phenyl-1-[2-(piperidin-1-yldiazenyl)phenyl]propan-1-one (S10)

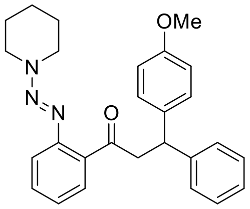

A reaction vial charged with $Rh(nbd)_2BF_4$ (15.0 mg, 0.04 mmol, 10 mol%) and dppe (15.9 mg, 0.04 mmol, 10 mol%) was evacuated and back-filled with N_2 gas for 3 times, and to this was added CH_2Cl_2 (0.5 mL). The resulting solution was bubbled with H_2 gas for 2 min and then with N_2 gas until the volume of the solution had become around 0.2 mL. This solution was then transferred to a N_2-purged reaction vial containing aldehyde **1a** (86.9 mg, 0.4 mmol, 1.0 equiv), with the use of additional 0.2 mL of CH_2Cl_2. Phenylacetylene (66 µL, 0.60 mmol) was added and the reaction mixture was stirred at 23 ºC for 16 h. The mixture was then transferred to a N_2-purged reaction vial containing 4-methoxyphenylboronic acid (121.6 mg, 0.80 mmol) and K_2CO_3 (55.3 mg, 0.40 mmol), followed by additions of acetone (3.6 mL) and MeOH (0.4 mL). The reaction mixture was stirred in a sealed tube at 55 ºC for 24 h. It was then diluted with Et_2O, filtered through a pad of silica and concentrated under reduced pressure. Purification by flash column chromatography (petrol/Et_2O, 4:1) afforded the title compound (137 mg, 80% yield) as a thick yellow oil. **^{1}H NMR** (400 MHz, $CDCl_3$): δ 7.37 (d, J = 8.0 Hz, 1H, Ar-*H*), 7.28 (t, J = 7.5 Hz, 1H, Ar-*H*), 7.16 – 6.96 (m, 9H, Ar-*H*), 6.67 (d, J = 8.5 Hz, 2H, Ar-*H*), 4.53 (t, J = 7.5 Hz, 1H, C*H*), 3.70 – 3.56 (m, 9H, CH-CH_2, N(CH_2)$_2$ and OCH_3), 1.54 (br. s, 6H, 3 × CH_2); **^{13}C NMR** (101 MHz, $CDCl_3$): δ 204.2, 158.0, 148.7, 144.8, 136.5, 134.8, 131.6, 128.9, 128.7, 128.4, 127.9, 126.2, 125.4, 117.7, 113.8, 55.3, 53.0, 51.3, 45.9, 44.1, 26.3, 24.5, 24.3; **IR** ν_{max} (film)/cm^{-1} 2939, 2856, 1674, 1610, 1591, 1510, 1418, 1355, 1295, 1247, 1181, 1110, 1096, 1033, 987, 909, 829, 761, 729, 699; **HRMS** (ESI$^+$) calculated for $C_{27}H_{30}N_3O_2$ [M+H]$^+$: 428.2333, found: 428.2325.

3-(4-Methoxyphenyl)-3-phenyl-1-[4'-(trifluoromethyl)-[1,1'-biphenyl]-2-yl] propan-1-one (14)

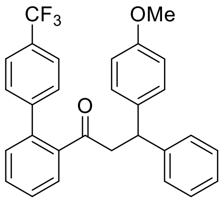

Under N_2 atmosphere, $BF_3·OEt_2$ (0.03 mL, 0.20 mmol) was added to a solution of aryltriazenyl ketone **S10** (85.5 mg, 0.20 mmol), 4-(trifluoromethyl)phenylboronic acid (76.0 mg, 0.40 mmol) and $Pd(OAc)_2$ (2.2 mg, 0.01 mmol) in 1,4-dioxane (2 mL), and the mixture was stirred at 23 ºC for 24 h. It was then diluted with Et_2O, filtered through a pad of silica and concentrated under reduced pressure. Purification by flash column chromatography (petrol/Et_2O, 9:1) afforded the title compound (92 mg, 99% yield) as a thick yellow oil. **^{1}H NMR** (400 MHz, $CDCl_3$): δ 7.58 – 7.51 (m, 3H, Ar-*H*), 7.47 – 7.41 (m, 2H, Ar-*H*), 7.37 (d, J = 7.5 Hz, 1H, Ar-*H*), 7.30 – 7.18 (m, 5H, Ar-*H*), 7.13 (d, J = 7.5 Hz, 2H, Ar-*H*), 7.06 (d, J = 8.5 Hz, 2H, Ar-*H*), 6.82 (d, J = 8.5 Hz, 2H, Ar-*H*), 4.58 (t, J = 7.5 Hz, 1H, C*H*), 3.80 (s, 3H, OCH_3), 3.31 (d, J = 7.5 Hz, 2H, CH_2); **^{13}C NMR** (101 MHz, $CDCl_3$): δ 203.8, 158.2, 144.3, 144.0, 140.3, 139.1, 135.7, 130.9, 130.6, 129.6 (q, $J_{C\text{-}F}$ = 32.5 Hz), 129.2, 128.9, 128.6, 128.1, 128.0, 127.8, 126.5, 125.5 (q, $J_{C\text{-}F}$ = 3.5 Hz), 124.3 (q, $J_{C\text{-}F}$ = 272.0 Hz), 114.0, 55.3, 49.0, 45.5; **^{19}F NMR** (377 MHz, $CDCl_3$): δ –62.3; **IR** ν_{max} (film)/cm^{-1} 3061, 3029, 2934, 2837, 1692, 1613, 1511, 1324, 1248, 1164, 1122, 1069, 1033, 844, 766, 735, 699; **HRMS** (ESI$^+$) calculated for $C_{29}H_{23}F_3O_2Na$ [M+Na]$^+$: 483.1542, found: 483.1540.

3. Multiple C–H Functionalizations Followed by Suzuki-Coupling

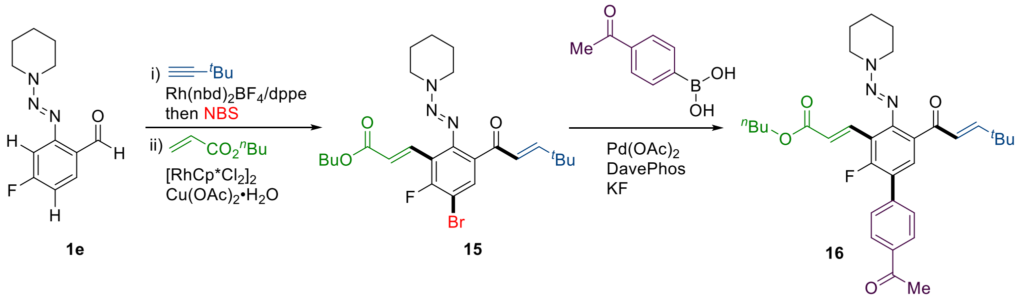

(*E*)-1-{5-Bromo-4-fluoro-2-[(*E*)-piperidin-1-yldiazenyl]phenyl}-4,4-dimethylpent -2-en-1-one (S5)

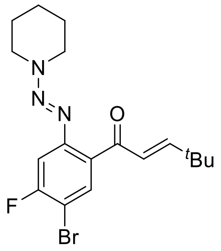

Prepared according to a modified **General Procedure F (i)** on 2.0 mmol scale, using 1) aldehyde **1e** (471 mg) and 3,3-dimethyl-1-butyne (0.37 mL), $Rh(nbd)_2BF_4$ (37 mg), dppe (40 mg), CH_2Cl_2 (2 mL),

16 h, and 2) NBS (534 mg), CH_2Cl_2 (8 mL), 3 h. Purification by flash column chromatography (petrol/CH_2Cl_2, 1:1) afforded the title compound (782 mg, 99% yield) as a thick yellow oil. Data consistent with those obtained from a smaller scale reaction.

(*E*)-Butyl 3-{3-bromo-5-[(*E*)-4,4-dimethylpent-2-enoyl]-2-fluoro-6-[(*E*)-piperidin-1-yldiazenyl]phenyl}acrylate (15)

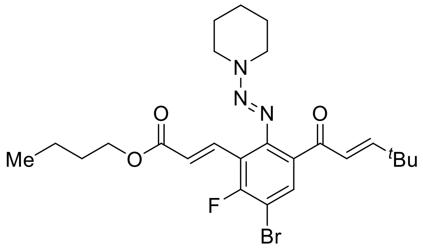

Under N_2 atmosphere, *n*-butyl acrylate (0.72 mL, 5.0 mmol) was added to a suspension of aryltriazenyl enone **S5** (782 mg, 2.0 mmol), $[RhCp*Cl_2]_2$ (62 mg, 0.10 mmol) and $Cu(OAc)_2 \cdot H_2O$ (799 mg, 4.0 mmol) in MeOH (13 mL), and the reaction mixture was stirred at 70 ºC for 20 h. It was then diluted with Et_2O, filtered through a pad of silica and concentrated under reduced pressure. Purification by flash column chromatography (petrol/Et_2O, 85:15) afforded the title compound (695 mg, 67% yield) as a yellow solid. **m.p.** (petrol): 67 – 69 ºC; **^{1}H NMR** (400 MHz, $CDCl_3$): δ 8.10 (d, J = 16.5 Hz, 1H, CH=C*H*-Ar), 7.53 (d, J = 7.0 Hz, 1H, Ar-*H*), 6.71 (d, J = 16.5 Hz, 1H, C*H*=CH-Ar), 6.63 (d, J = 16.0 Hz, 1H, CO-CH=C*H*), 5.84 (d, J = 16.0 Hz, 1H, CO-C*H*=CH), 4.19 (t, J = 6.5 Hz, 2H, CO_2CH_2), 3.90 – 3.64 (2 × br. s, 4H, $N(CH_2)_2$), 1.73 – 1.63 (m, 8H, 4 × CH_2), 1.48 – 1.37 (m, 2H, CH_2-CH_3), 1.00 (s, 9H, $C(CH_3)_3$), 0.94 (t, J = 7.5 Hz, 3H, CH_2-CH_3); **^{13}C NMR** (101 MHz, $CDCl_3$): δ 193.7, 167.4, 158.7 (d, J_{C-F} = 256.0 Hz), 156.6, 149.5 (d, J_{C-F} = 5.0 Hz), 134.4, 134.1 (d, J_{C-F} = 2.0 Hz), 130.3 (d, J_{C-F} = 3.5 Hz), 125.6, 124.4 (d, J_{C-F} = 13.5 Hz), 118.3 (d, J_{C-F} = 13.5 Hz), 105.7 (d, J_{C-F} = 23.0 Hz), 64.6, 52.9, 44.6, 33.8, 30.8, 28.8, 26.5, 24.4, 24.0, 19.3, 13.9; **^{19}F NMR** (377 MHz, $CDCl_3$): δ –101.3; **IR** ν_{max} (film)/cm^{-1} 2957, 2864, 1714, 1674, 1622, 1563, 1417, 1398, 1366, 1302, 1253, 1176, 1109, 986, 874, 851, 726, 653; **HRMS** (ESI$^+$) calculated for $C_{25}H_{34}BrFN_3O_3$ [M+H]$^+$: 522.1762, found: 522.1764.

(*E*)-Butyl 3-{4'-acetyl-5-[(*E*)-4,4-dimethylpent-2-enoyl]-2-fluoro-4-[(*E*)-piperidin-1-yldiazenyl]-[1,1'-biphenyl]-3-yl}acrylate (16)

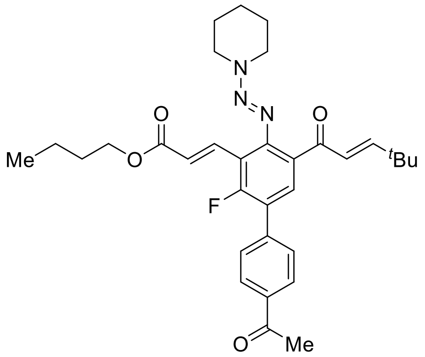

Under N_2 atmosphere, a suspension of compound **15** (52.2 mg, 0.10 mmol), 4-acetylphenyl boronic acid (32.8 mg, 0.20 mmol), $Pd(OAc)_2$ (1.1 mg, 0.005 mmol), 2-dicyclohexylphosphino-2′-(*N*,*N*-dimethylamino)biphenyl (DavePhos, 3.9 mg, 0.01 mmol) and KF (14.5 mg, 0.25 mmol) in THF (0.5 mL) was stirred at 60 ºC for 16 h. It was then diluted with H_2O and extracted with CH_2Cl_2 (3×). The combined organic layers were dried over $MgSO_4$, filtered and concentrated under reduced pressure. Purification by flash column chromatography (petrol/Et_2O, 3:2) afforded the title compound (47 mg, 84% yield) as a yellow solid. **m.p.** (petrol): 153 – 155 ºC; **^{1}H NMR** (400 MHz, $CDCl_3$): δ 8.20 (d, J = 16.5 Hz, 1H, CH=C*H*-Ar), 8.02 (d, J = 8.0 Hz, 2H, Ar-*H*), 7.65 (d, J = 8.0 Hz, 2H, Ar-*H*), 7.48 (d, J = 8.0 Hz, 1H, Ar-*H*), 6.76 (d, J = 16.5 Hz, 1H, C*H*=CH-Ar), 6.69 (d, J = 16.0 Hz, 1H, CO-CH=C*H*), 5.90 (d, J = 16.0 Hz, 1H, CO-C*H*=CH), 4.20 (t, J = 6.5 Hz, 2H, CO_2CH_2), 3.94 – 3.68 (2 × br. s, 4H, $N(CH_2)_2$), 2.63 (s, 3H, CO-CH_3), 1.77 – 1.63 (m, 8H, 4 × CH_2), 1.49 – 1.37 (m, 2H, CH_2-CH_3), 1.02 (s, 9H, $C(CH_3)_3$), 0.95 (t, J = 7.5 Hz, 3H, CH_2-CH_3); **^{13}C NMR** (101 MHz, $CDCl_3$): δ 197.7, 194.7, 167.6, 159.6 (d, J_{C-F} = 259.0 Hz), 156.2, 150.2 (d, J_{C-F} = 5.5 Hz), 139.7, 136.4, 135.0, 132.0 (d, J_{C-F} = 6.0 Hz), 129.9 (d, J_{C-F} = 3.5 Hz), 129.3 (d, J_{C-F} = 3.0 Hz), 128.6, 125.9, 124.9 (d, J_{C-F} = 15.5 Hz), 124.1 (d, J_{C-F} = 13.5 Hz), 117.7 (d, J_{C-F} = 13.5 Hz), 64.5, 53.0, 44.6, 33.8, 30.9, 28.9, 26.8, 26.5, 24.5, 24.0, 19.3, 13.9; **^{19}F NMR** (377 MHz, $CDCl_3$): δ –112.2; **IR** ν_{max} (film)/cm^{-1} 2957, 2917, 2849, 1721, 1683, 1606, 1409, 1267, 1191, 1154, 1091, 732; **HRMS** (ESI$^+$) calculated for $C_{33}H_{41}FN_3O_4$ [M+H]$^+$: 562.3076, found: 562.3074.

X. Reference

(1) Dong, X.; Ma, P.; Zhang, T.; Jalani, H. B.; Li, G.; Lu, H. Iridium-Catalyzed C–H Amination of Weinreb Amides: A Facile Pathway toward Anilines and Quinazolin-2,4-diones. *J. Org. Chem.* **2020**, *85*, 13096–13107.

(2) Sarli, V.; Huemmer, S.; Sunder-Plassmann, N.; Mayer, T. U.; Giannis, A. Synthesis and Biological Evaluation of Novel Eg5 Inhibitors. *ChemBioChem* **2005**, *6*, 2005–2013.

(3) Meier, C.; Görbig, U.; Müller, C.; Balzarini, J. cycloSal-PMEA and cycloAmb-PMEA: Potentially New Phosphonate Prodrugs Based on the cycloSal-Pronucleotide Approach. *J. Med. Chem.* **2005**, *48*, 8079–8086.

(4) Chong, J. M.; Shen, L.; Taylor, N. J. Asymmetric Conjugate Addition of Alkynylboronates to Enones. *J. Am. Chem. Soc.* **2000**, *122*, 1822–1823.

(5) Hooper, J. F.; Young, R. D.; Weller, A. S.; Willis, M. C. Traceless Chelation-Controlled Rhodium-Catalyzed Intermolecular Alkene and Alkyne Hydroacylation. *Chem. Eur. J.* **2013**, *19*, 3125–3130.

(6) Jie, X.; Shang, Y.; Zhang, X.; Su, W. Cu-Catalyzed Sequential Dehydrogenation–Conjugate Addition for *β*-Functionalization of Saturated Ketones: Scope and Mechanism. *J. Am. Chem. Soc.* **2016**, *138*, 5623–5633.

(7) Thomson, C. J.; Barber, D. M.; Dixon, D. J. One-Pot Catalytic Enantioselective Synthesis of 2-Pyrazolines. *Angew. Chem., Int. Ed.* **2019**, *58*, 2469–2473.

(8) Kan, J.; Zhang, M.; Zhang, X.; Lou, X.; Shang, Y.; Xu, B.; Yang, F.; Su, W. Oxidation of Enones for Regioselective [3+2] Cycloaddition through *γ*-Enone Radical Intermediates. *Chem. Eur. J.* **2019**, *25*, 15233–15238.

(9) Kim, H. Y.; Oh, K. 1,3-Dienones and 2*H*-Pyran-2-ones from Soft α-Vinyl Enolization of β-Chlorovinyl Ketones: Defined Roles of Brönsted and Lewis Base. *Org. Lett.* **2015**, *17*, 6254–6257.

(10) Rana, N. K.; Selvakumar, S.; Singh, V. K. Highly Enantioselective Organocatalytic Sulfa-Michael Addition to α,β-Unsaturated Ketones. *J. Org. Chem.* **2010**, *75*, 2089–2091.

(11) Rana, N. K.; Unhale, R.; Singh, V. K. Enantioselective Sulfa-Michael Addition of Thioacids to α,β-Unsaturated Ketones with Bifunctional Organocatalyst. *Tetrahedron Lett.* **2012**, *53*, 2121–2124.

(12) Minatti, A.; Zheng, X.; Buchwald, S. L. Synthesis of Chiral 3-Substituted Indanones via an Enantioselective Reductive-Heck Reaction. *J. Org. Chem.* **2007**, *72*, 9253–9258.

***Appendix** (Spectral Copies of ^{1}H, ^{13}C, and ^{19}F NMR of the Novel Compounds Obtained in this Study)*

2-Amino-3-fluoro-*N*-methoxy-*N*-methylbenzamide (S1b)

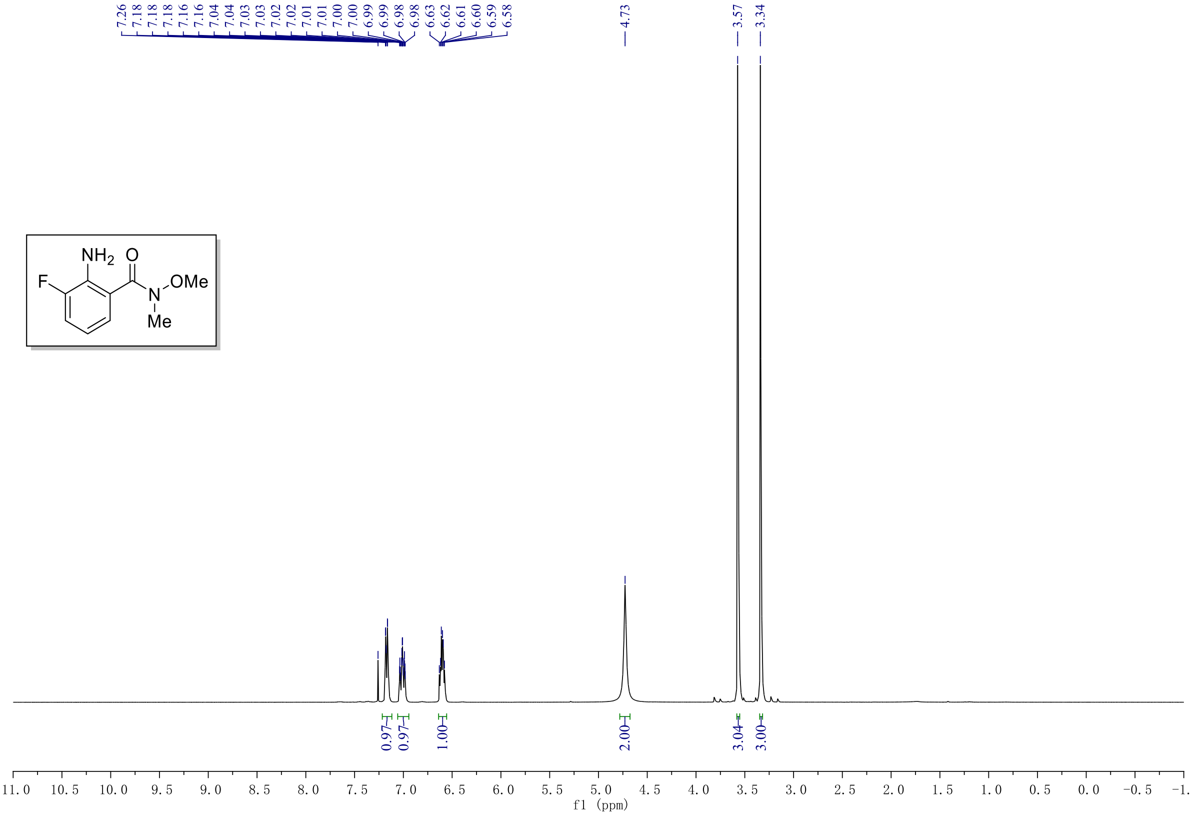

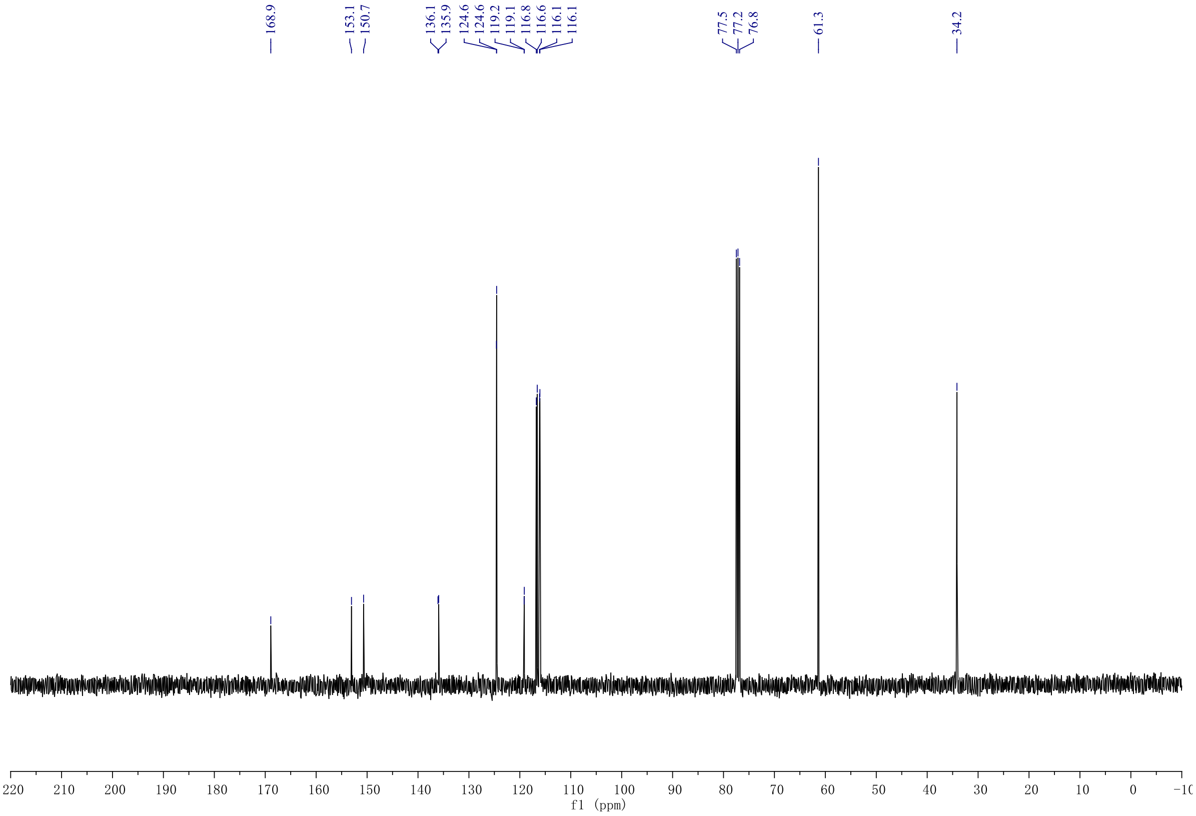

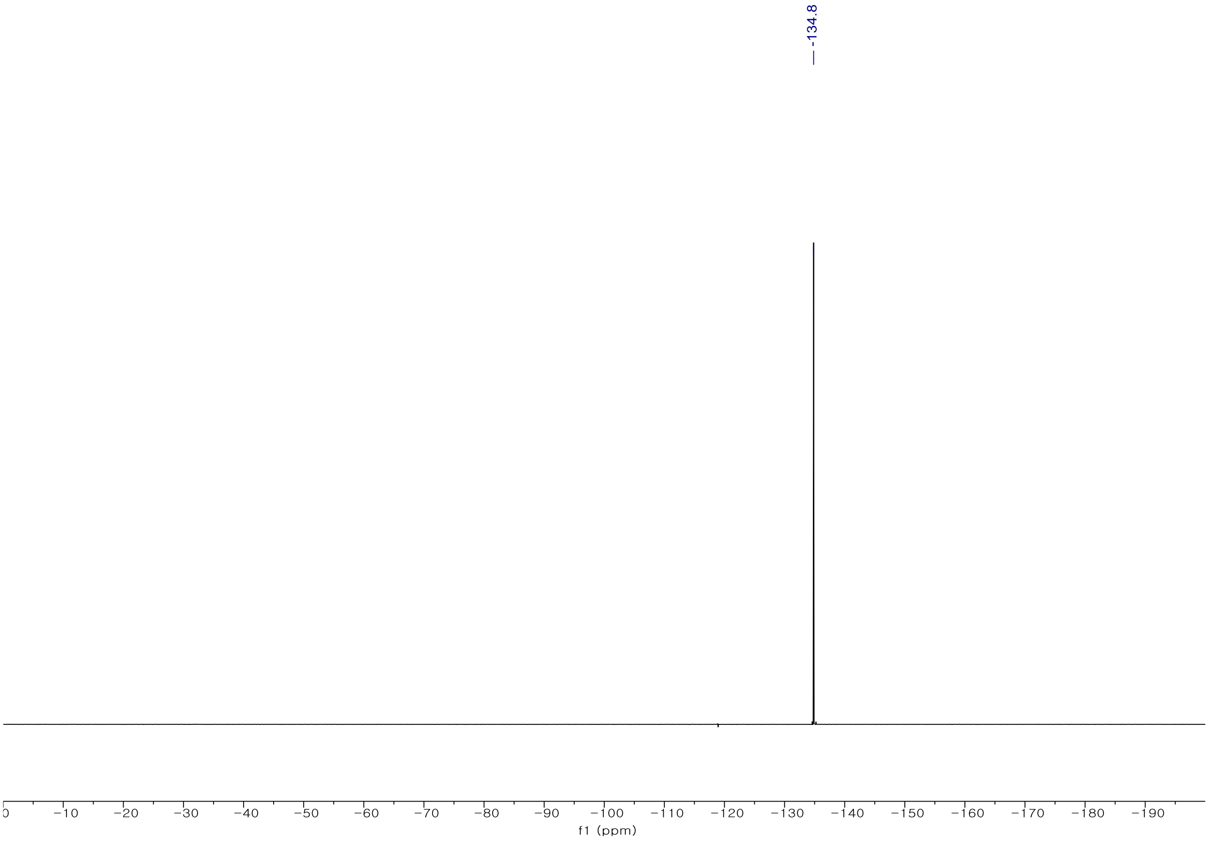
-134.8
0
-10
-20
-30
-40
-50
-60
-70
-80
-90
-100
-110
-120
-130
-140
-150
-160
-170
-180
-190
f1 (ppm)

2-Amino-*N*-methoxy-*N*-methyl-4-(trifluoromethyl)benzamide (S1d)

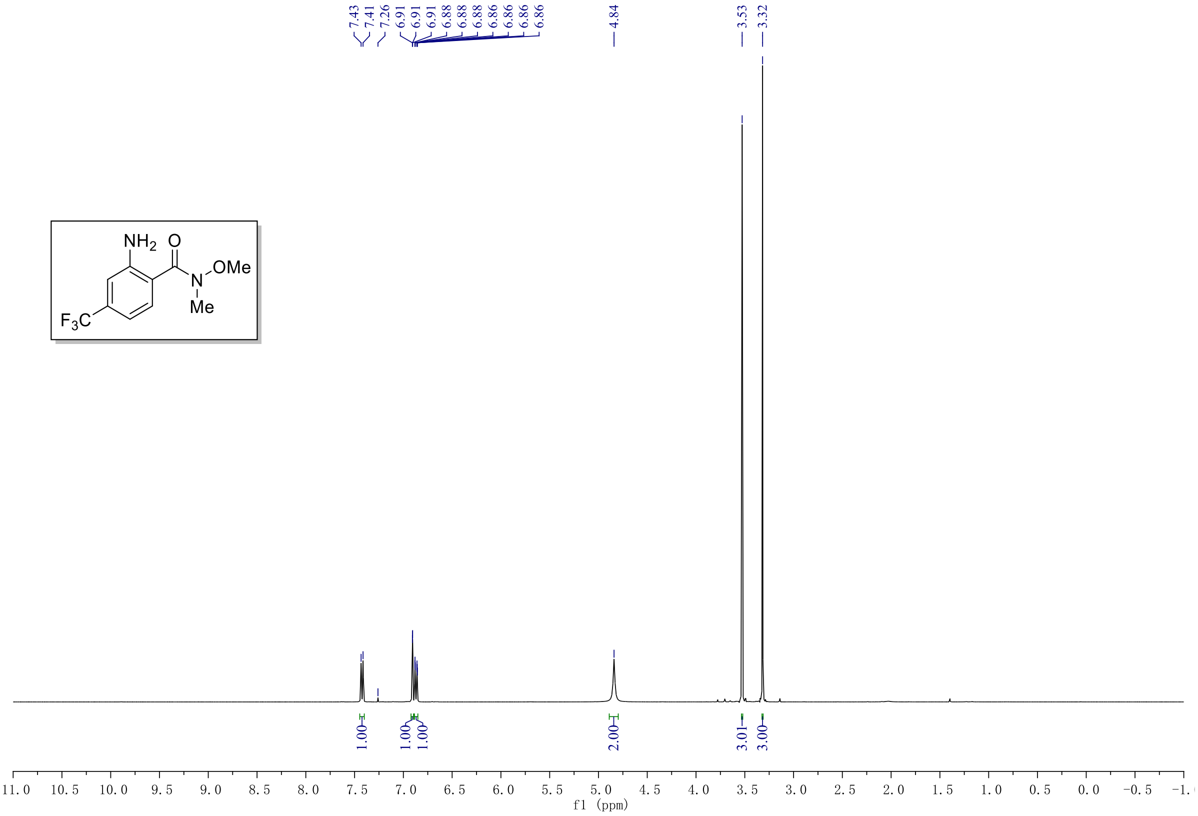

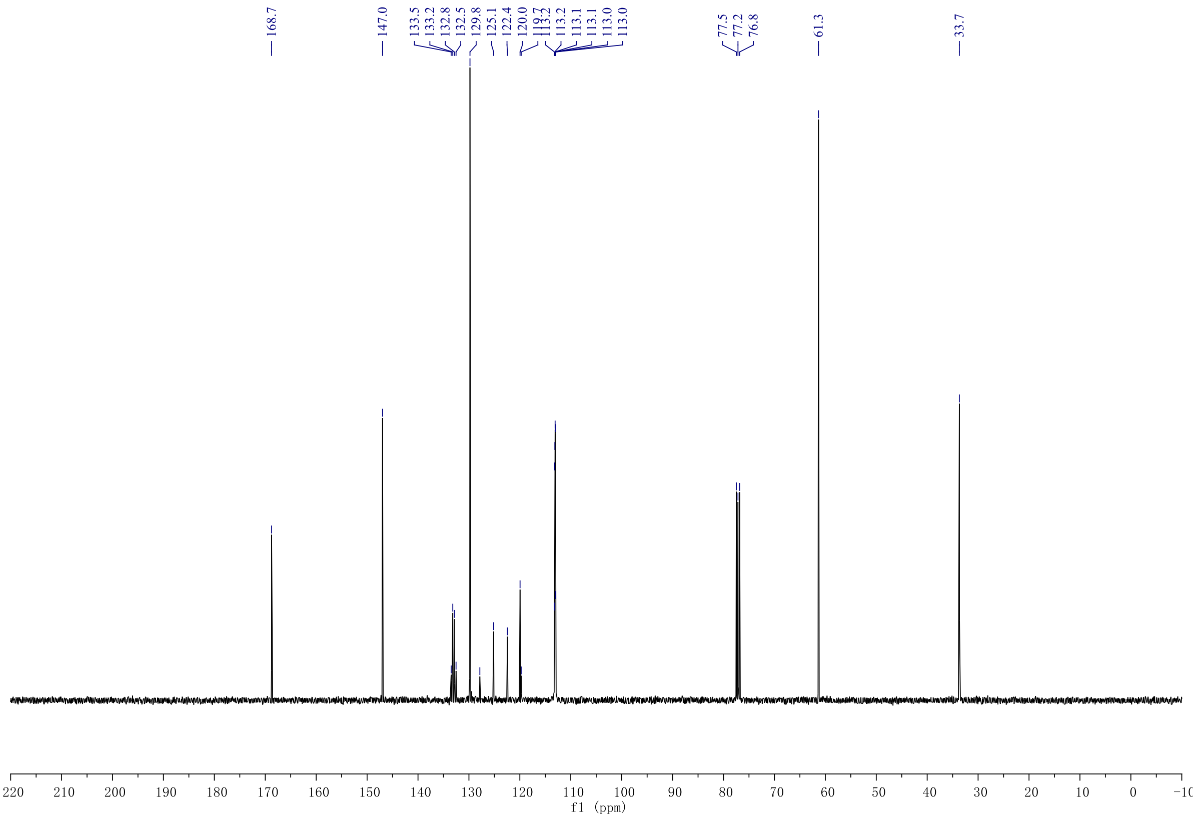

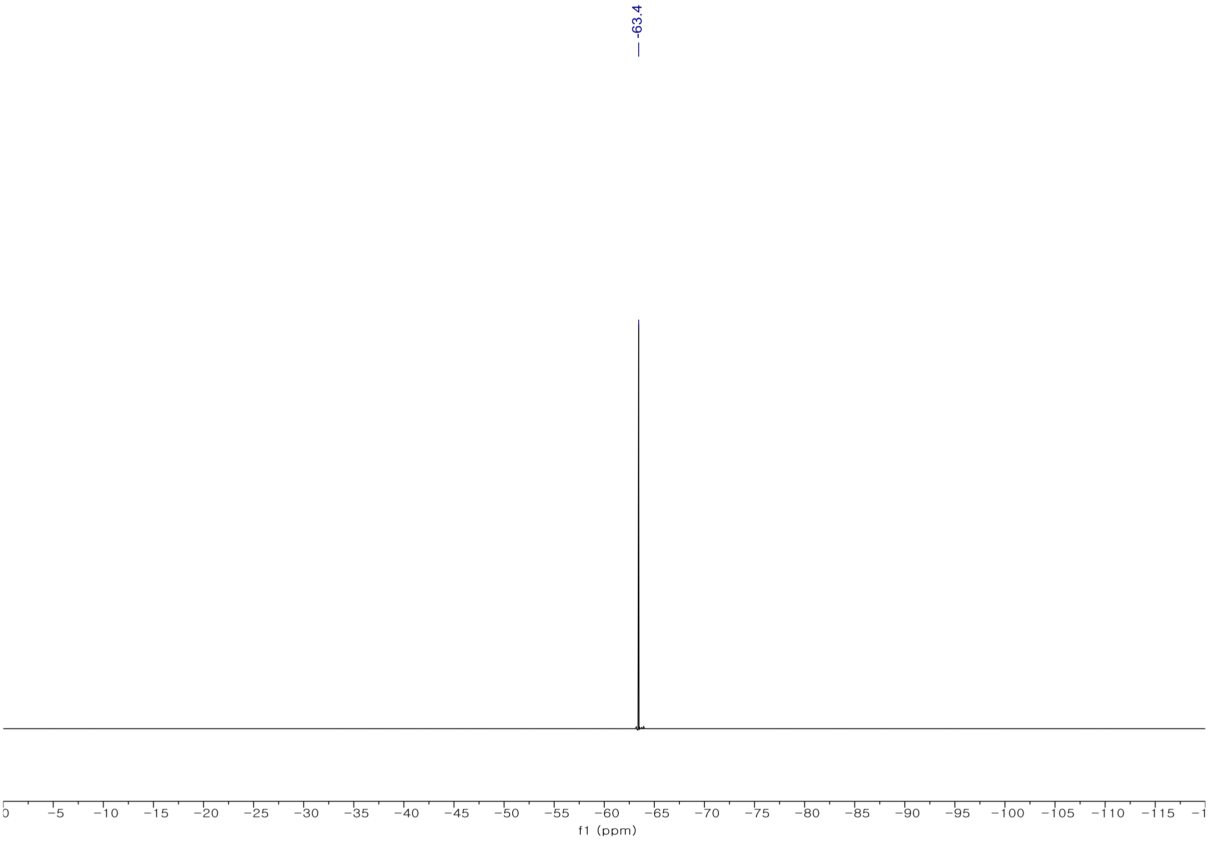
-63.4
0 −5 −10 −15 −20 −25 −30 −35 −40 −45 −50 −55 −60 −65 −70 −75 −80 −85 −90 −95 −100 −105 −110 −115
f1 (ppm)

2-Amino-*N*-methoxy-*N*-methyl-5-(trifluoromethoxy)benzamide (S1g)

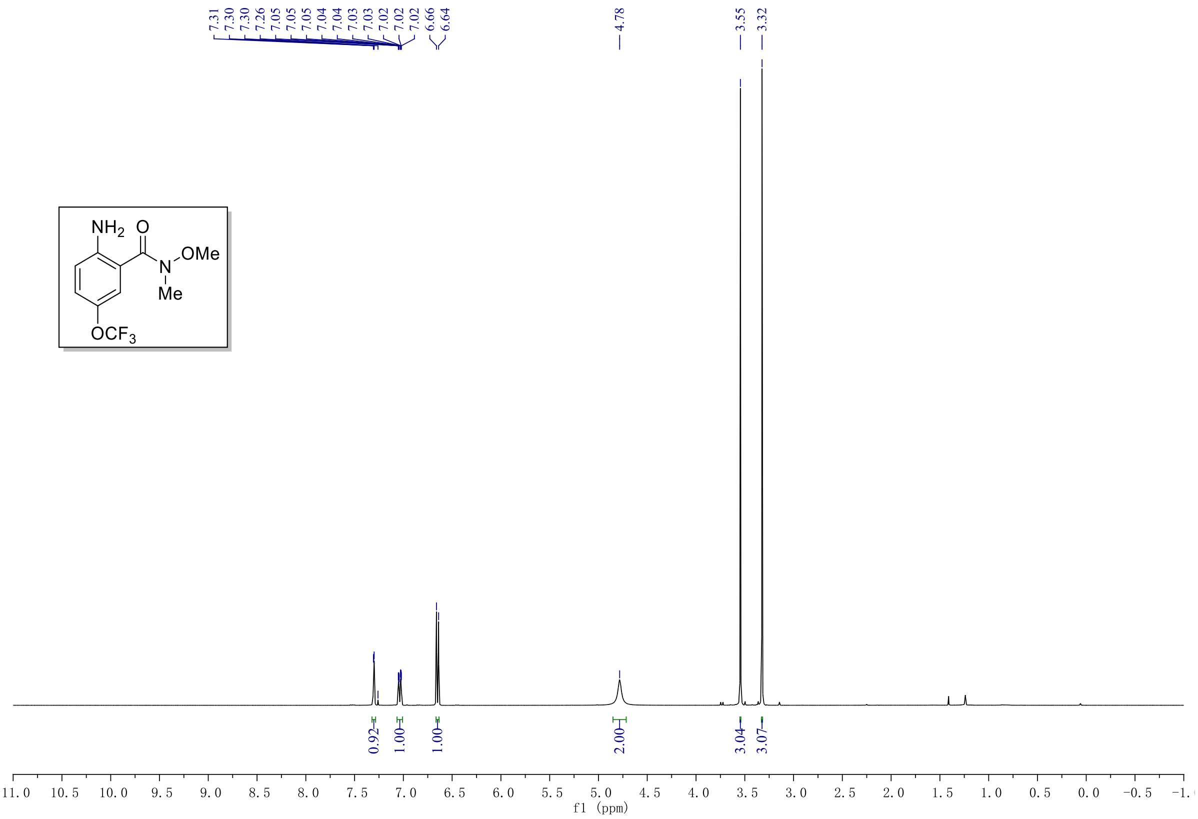

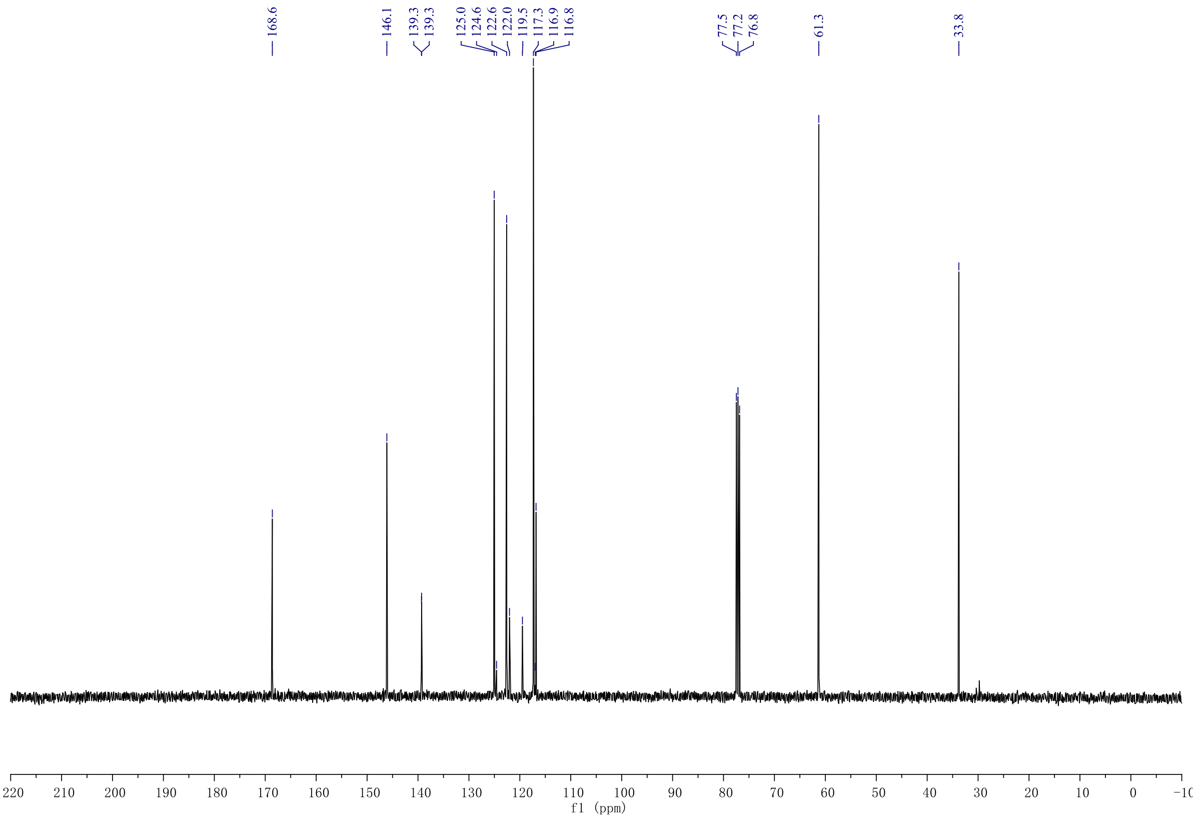

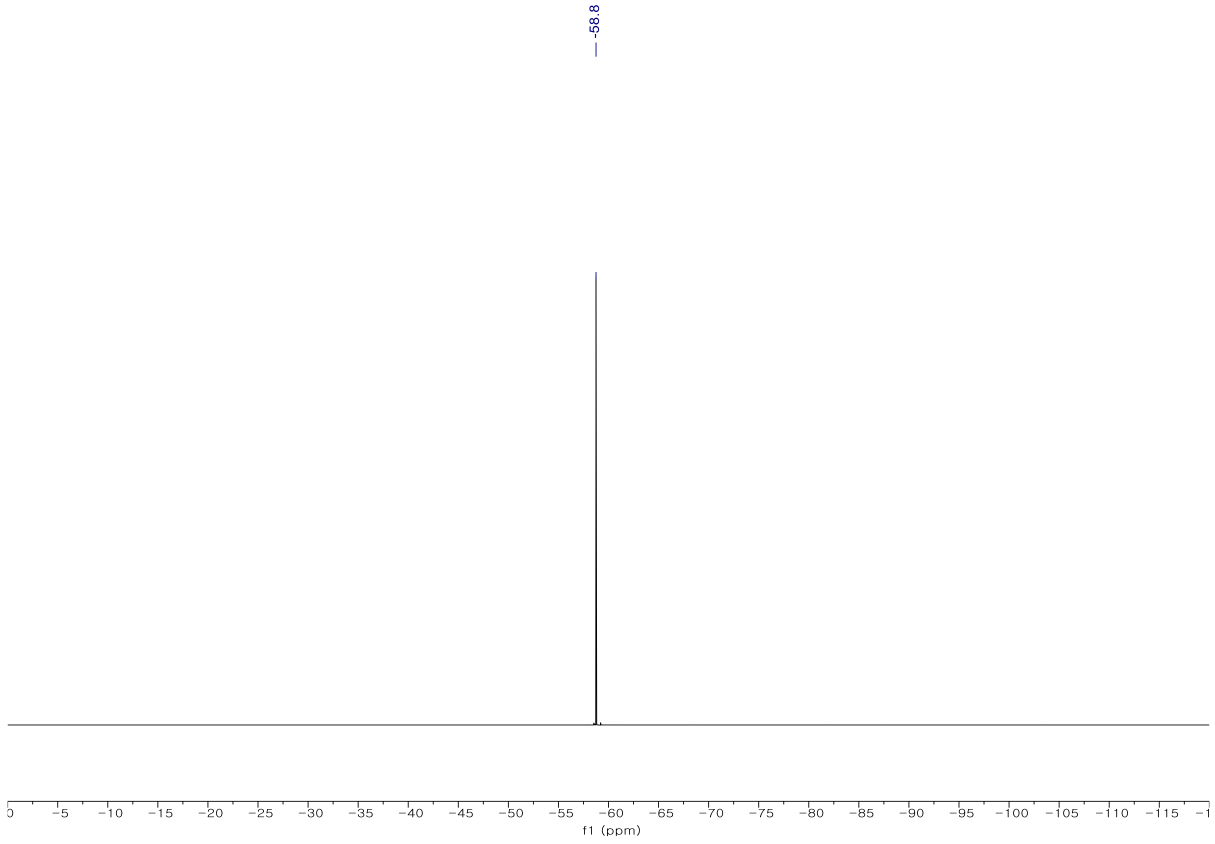

-58.8
0
-5
-10
-15
-20
-25
-30
-35
-40
-45
-50
-55
-60
-65
-70
-75
-80
-85
-90
-95
-100
-105
-110
-115
f1 (ppm)

(*E*)-2-(3,3-Diethyltriaz-1-en-1-yl)benzaldehyde (1a’)

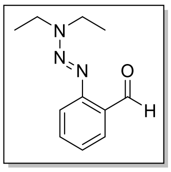

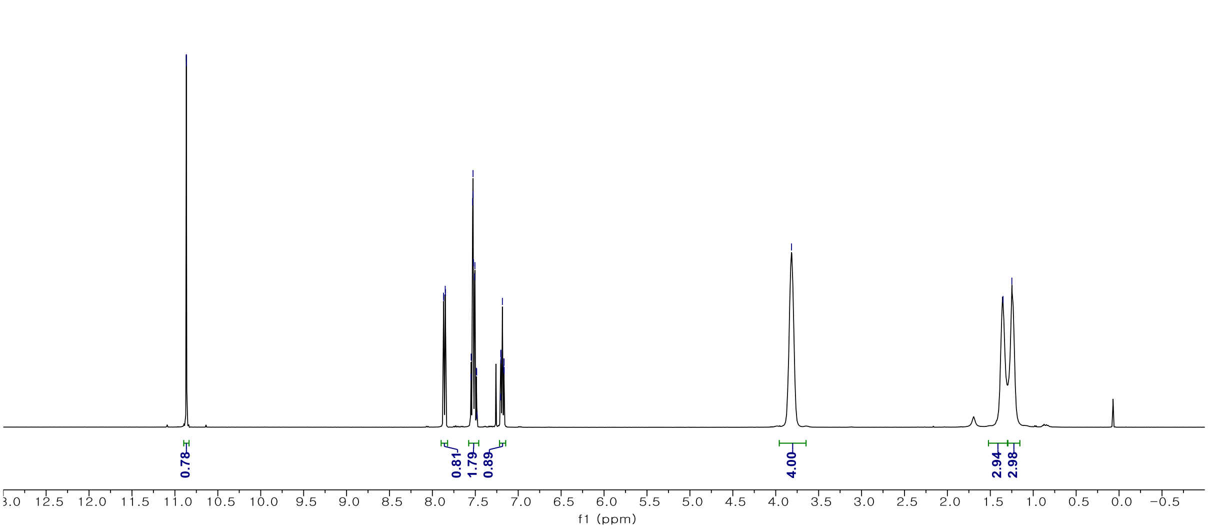

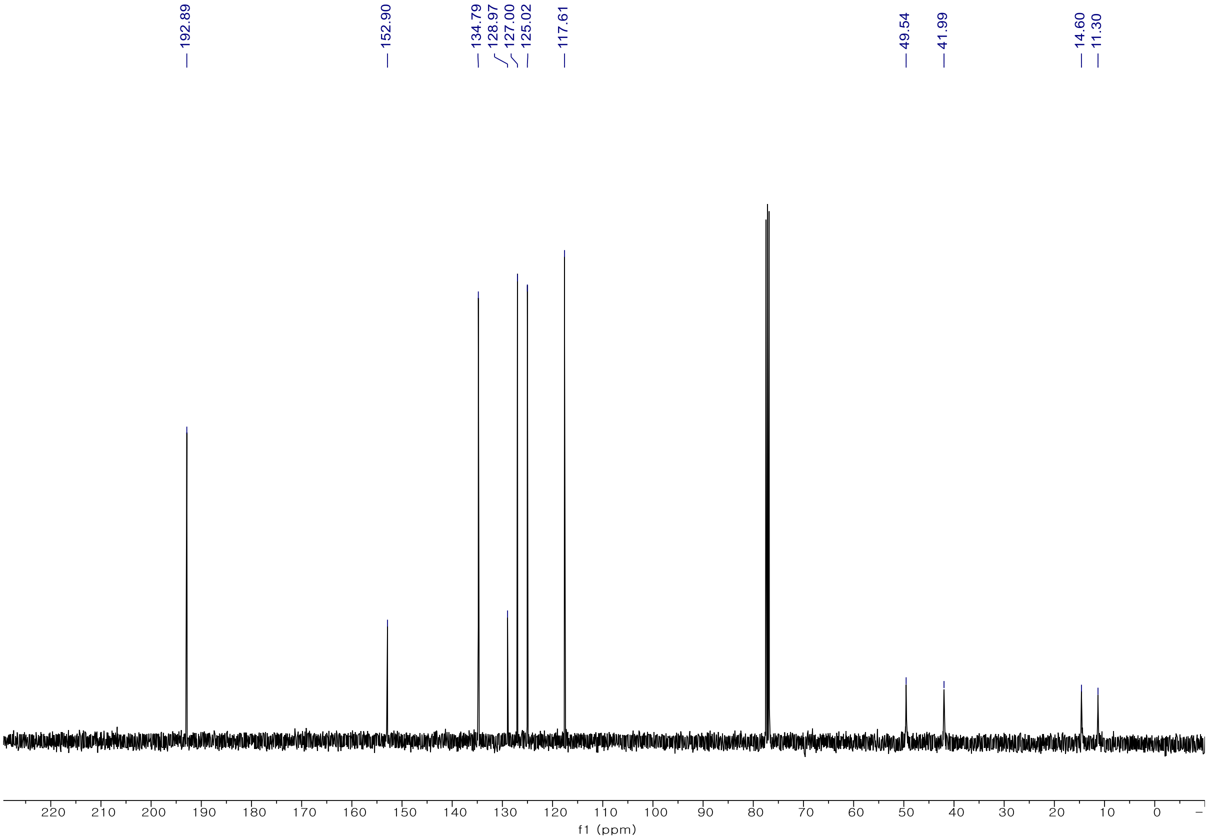

(*E*)-2-(Piperidin-1-yldiazenyl)benzaldehyde (1a)

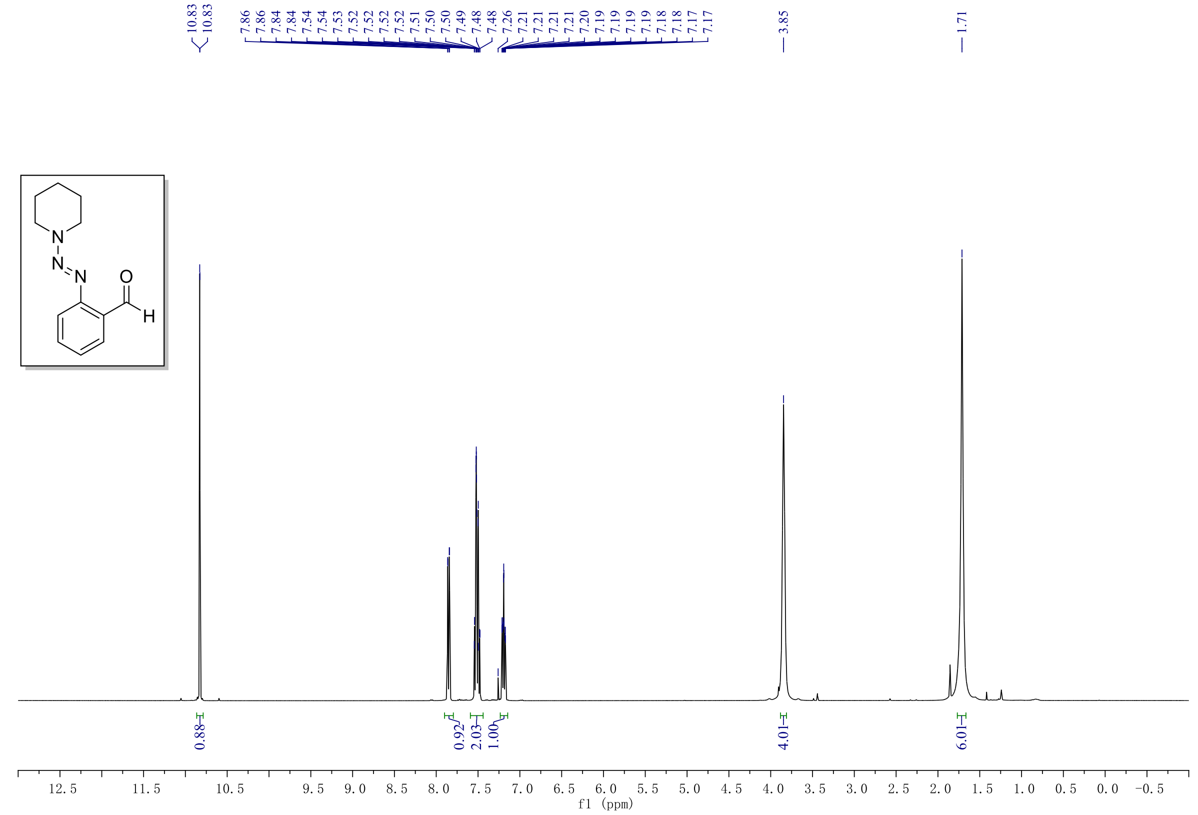

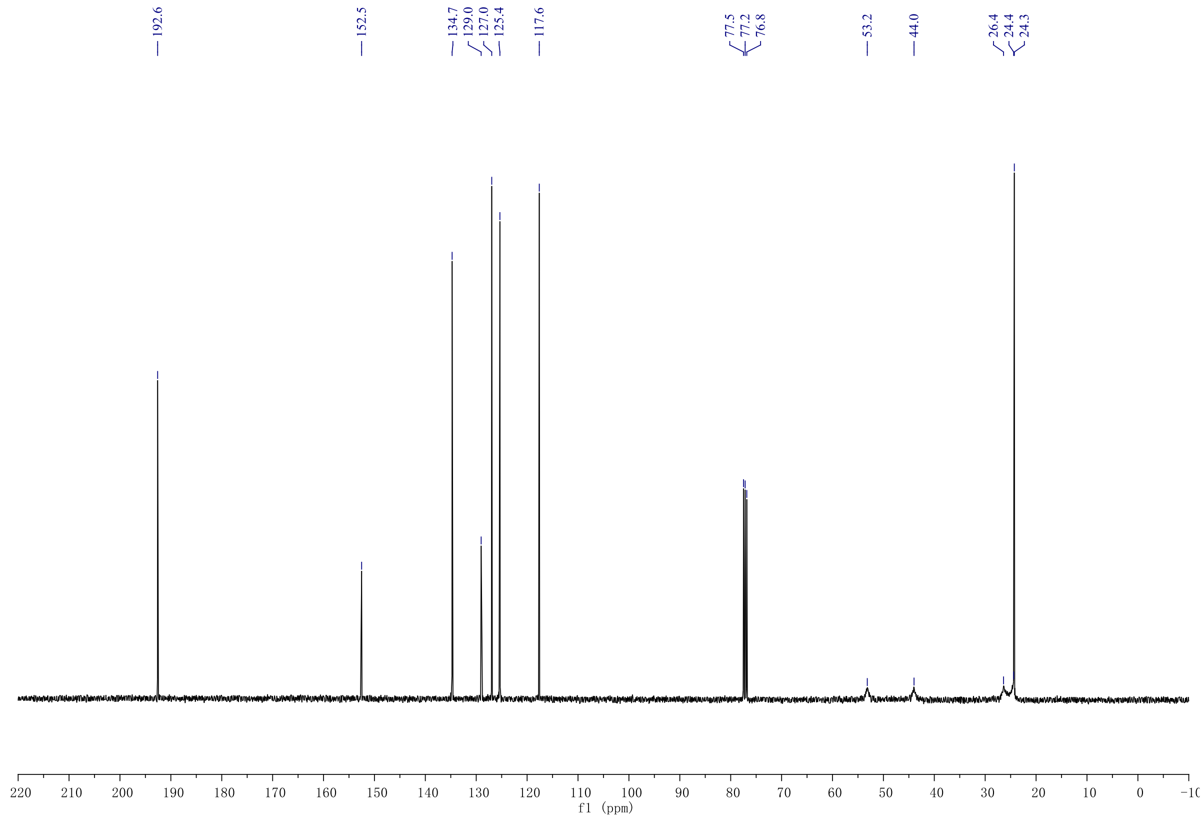

(*E*)-3-Fluoro-2-(piperidin-1-yldiazenyl)benzaldehyde (1b)

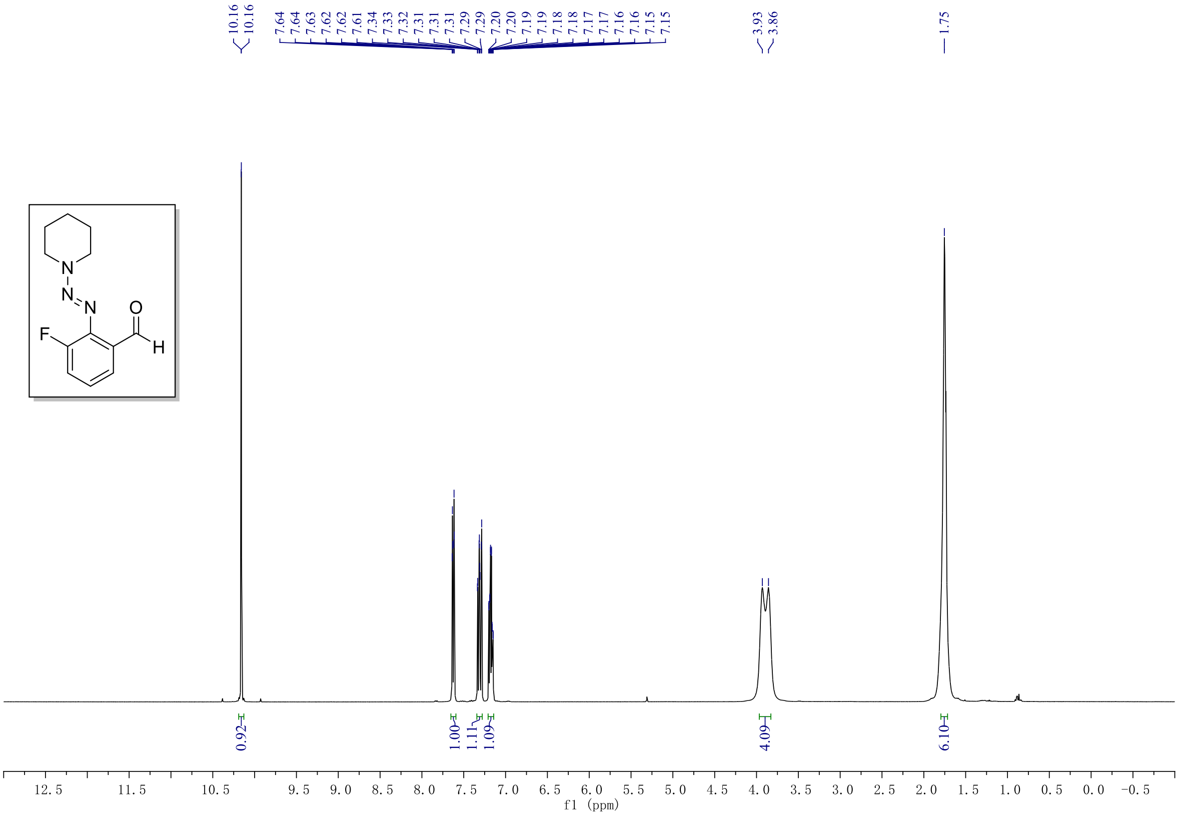

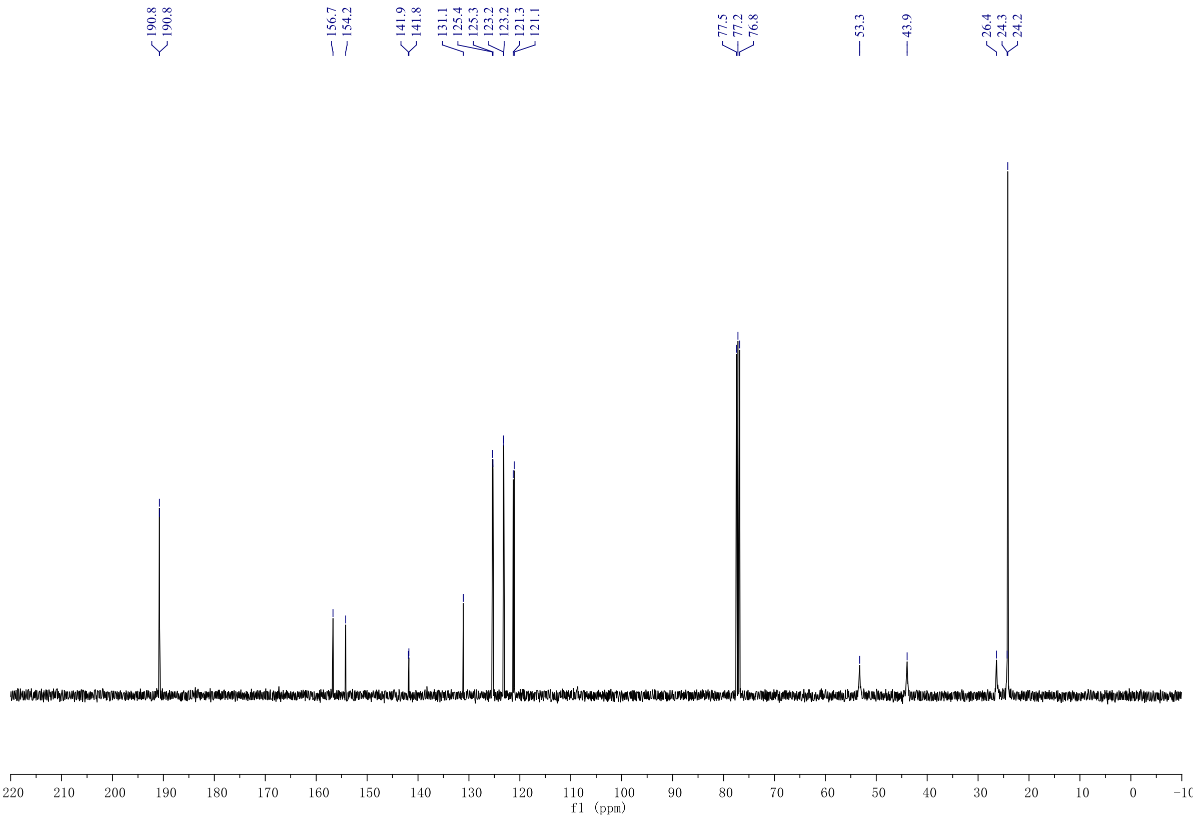

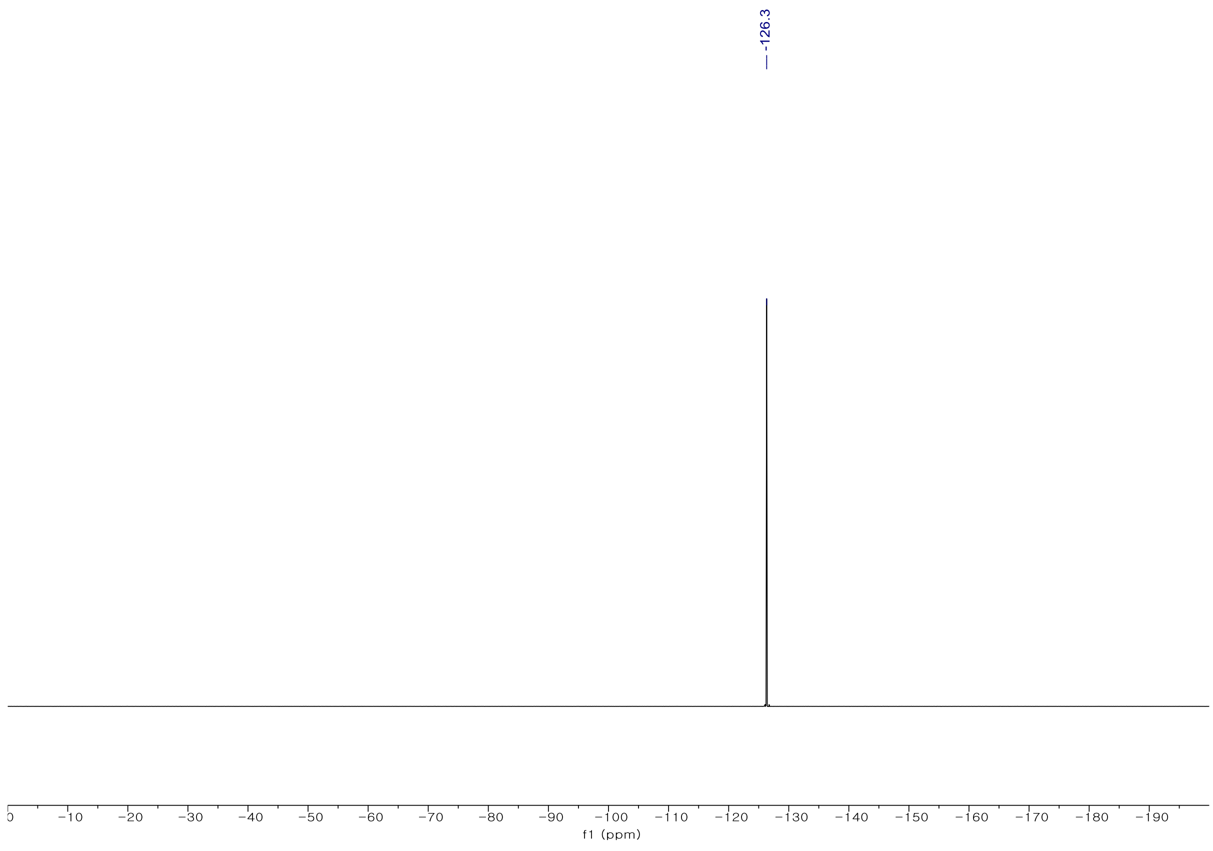
-126.3
0
−10
−20
−30
−40
−50
−60
−70
−80
−90
−100
−110
−120
−130
−140
−150
−160
−170
−180
−190
f1 (ppm)

(*E*)-4-Chloro-2-(piperidin-1-yldiazenyl)benzaldehyde (1c)

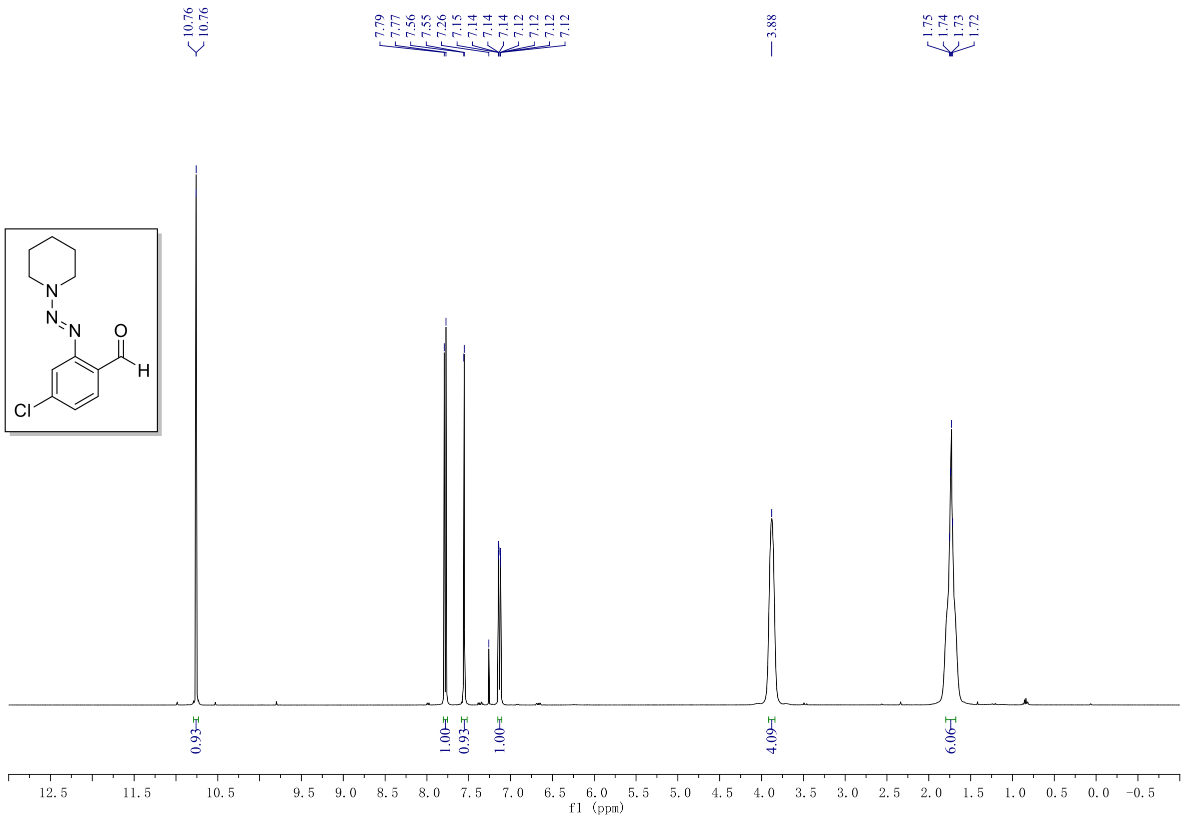

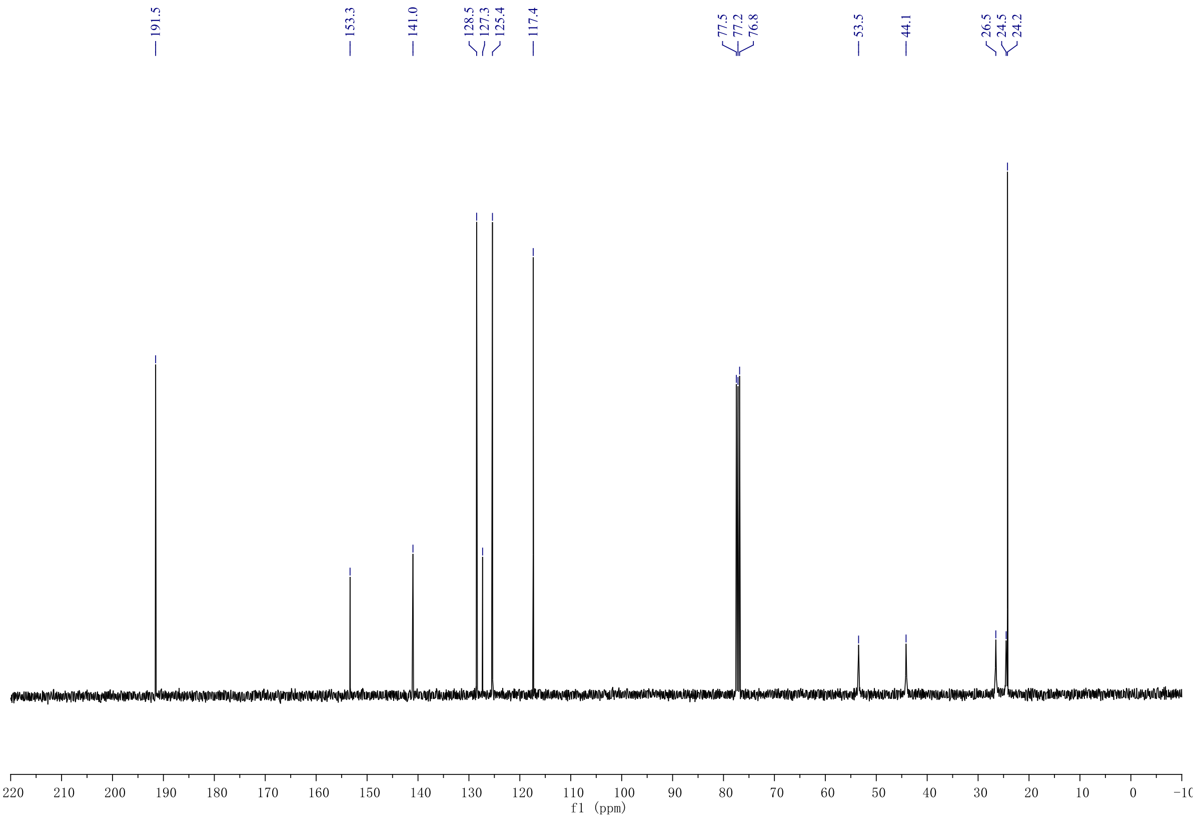

(*E*)-2-(Piperidin-1-yldiazenyl)-4-(trifluoromethyl)benzaldehyde (1d)

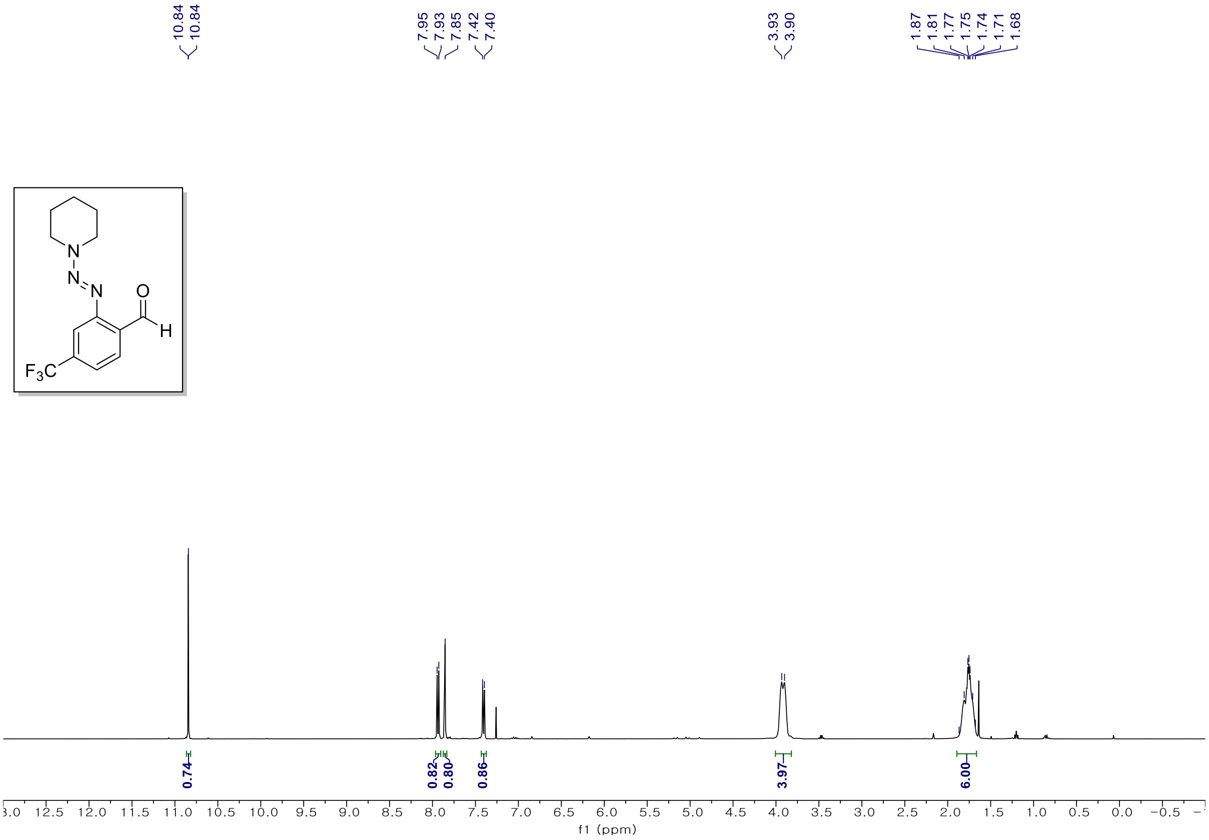

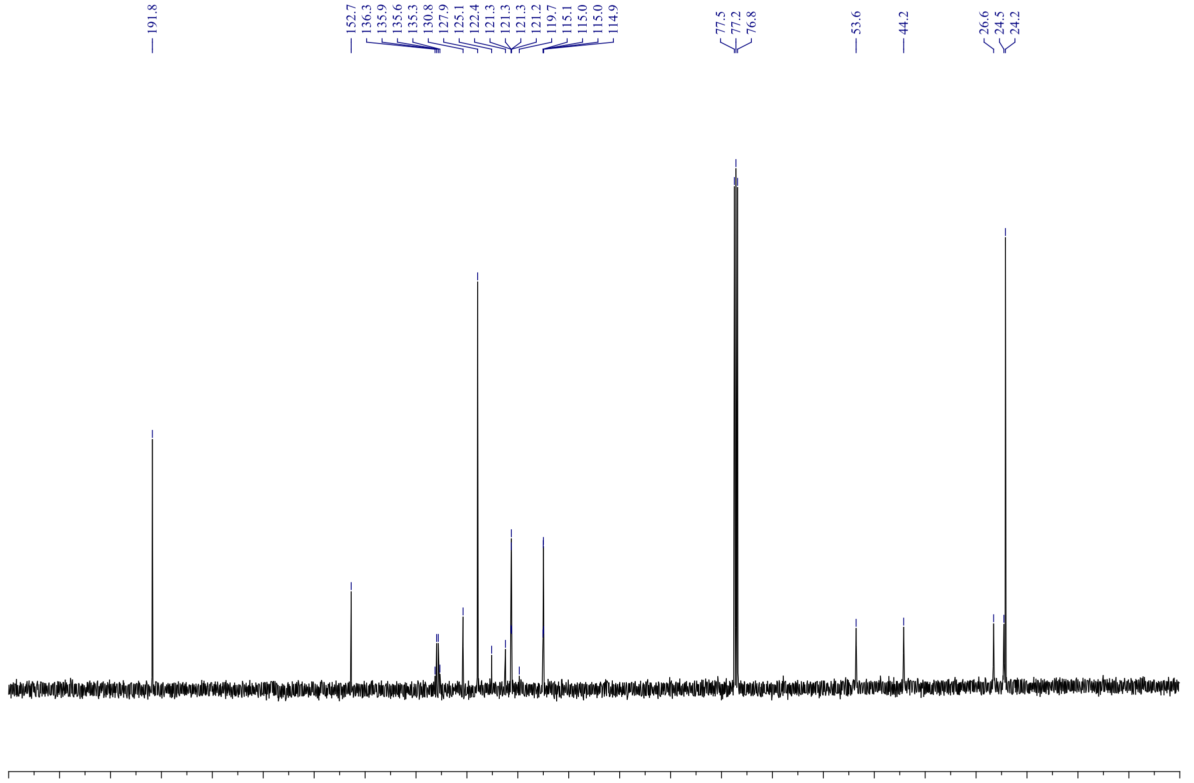

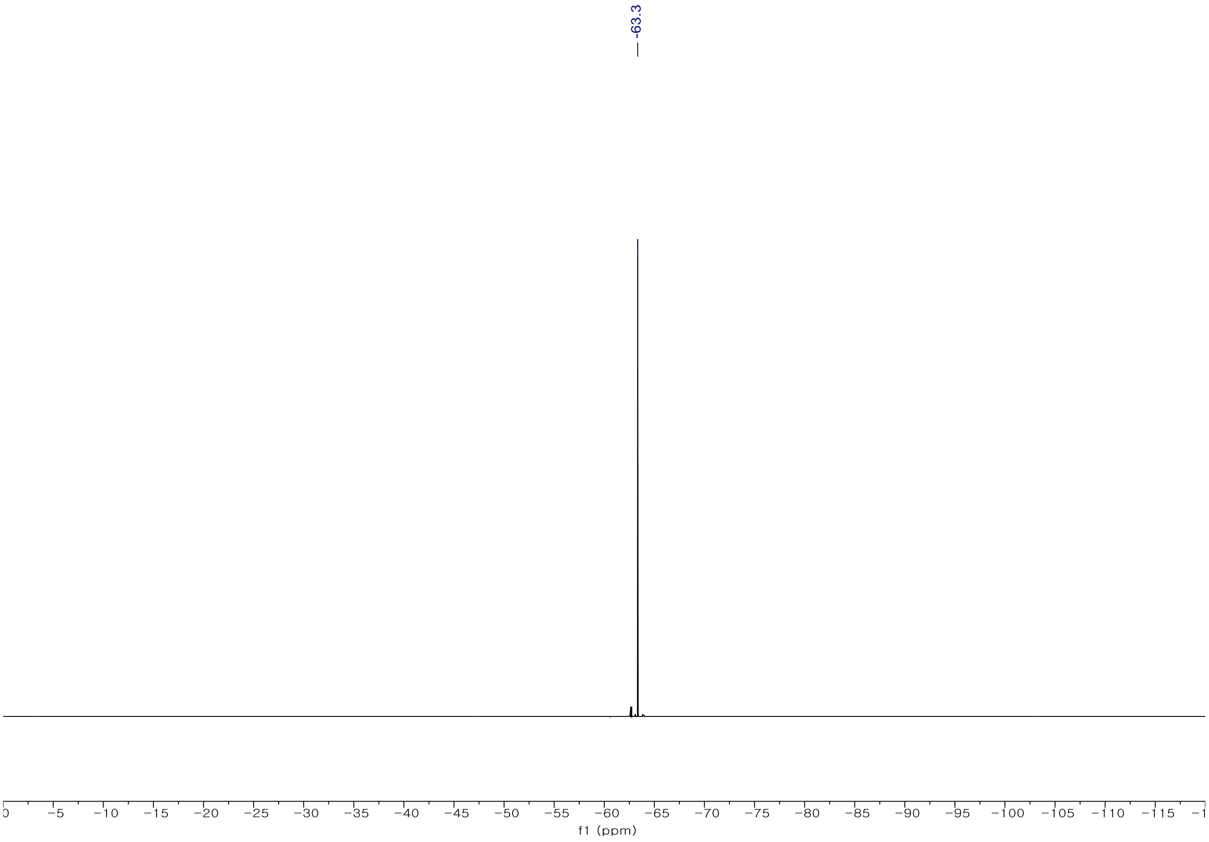
-63.3
f1 (ppm)

(*E*)-4-Fluoro-2-(piperidin-1-yldiazenyl)benzaldehyde (1e)

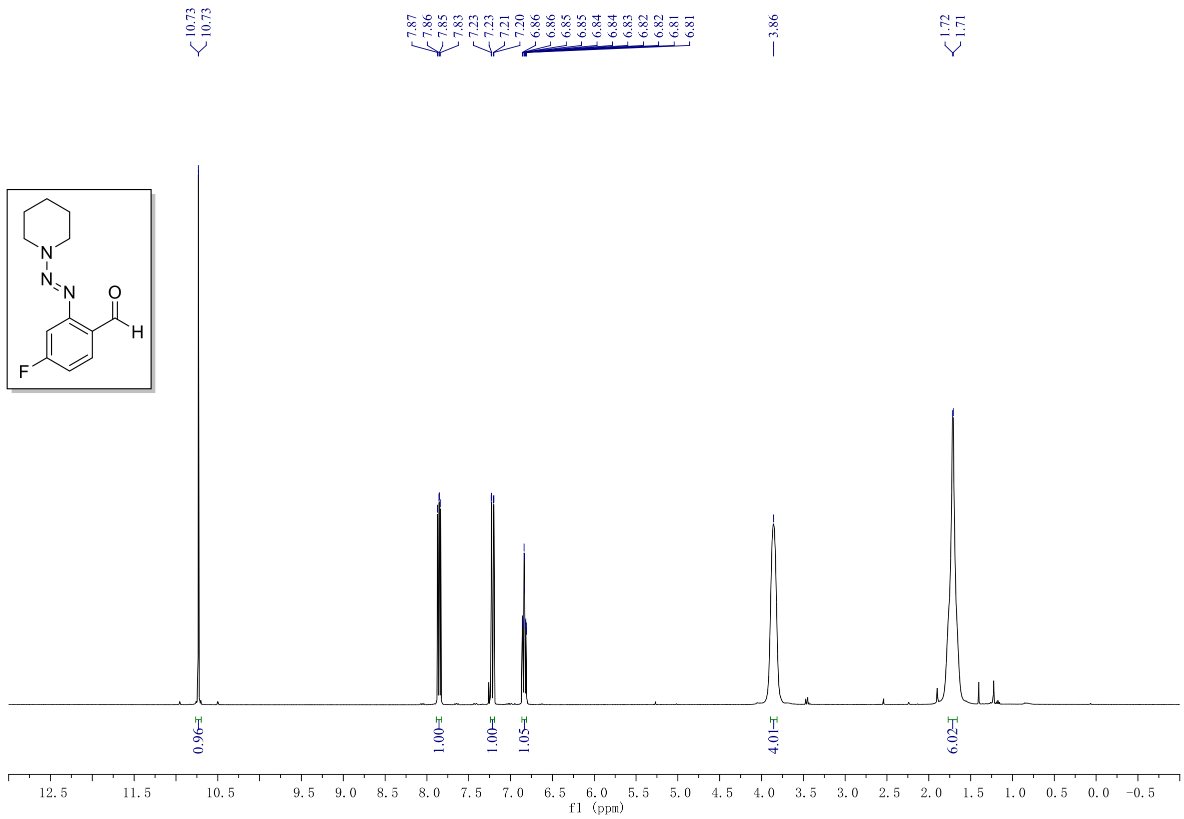

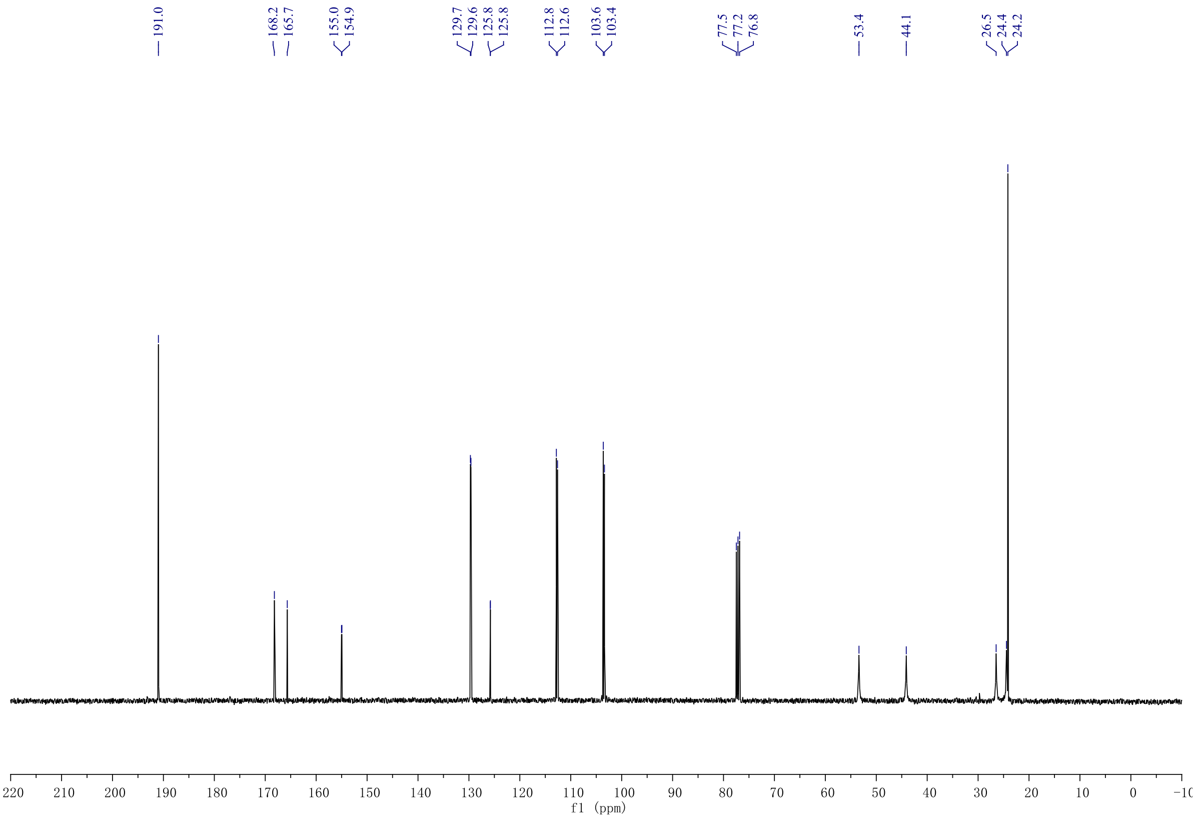

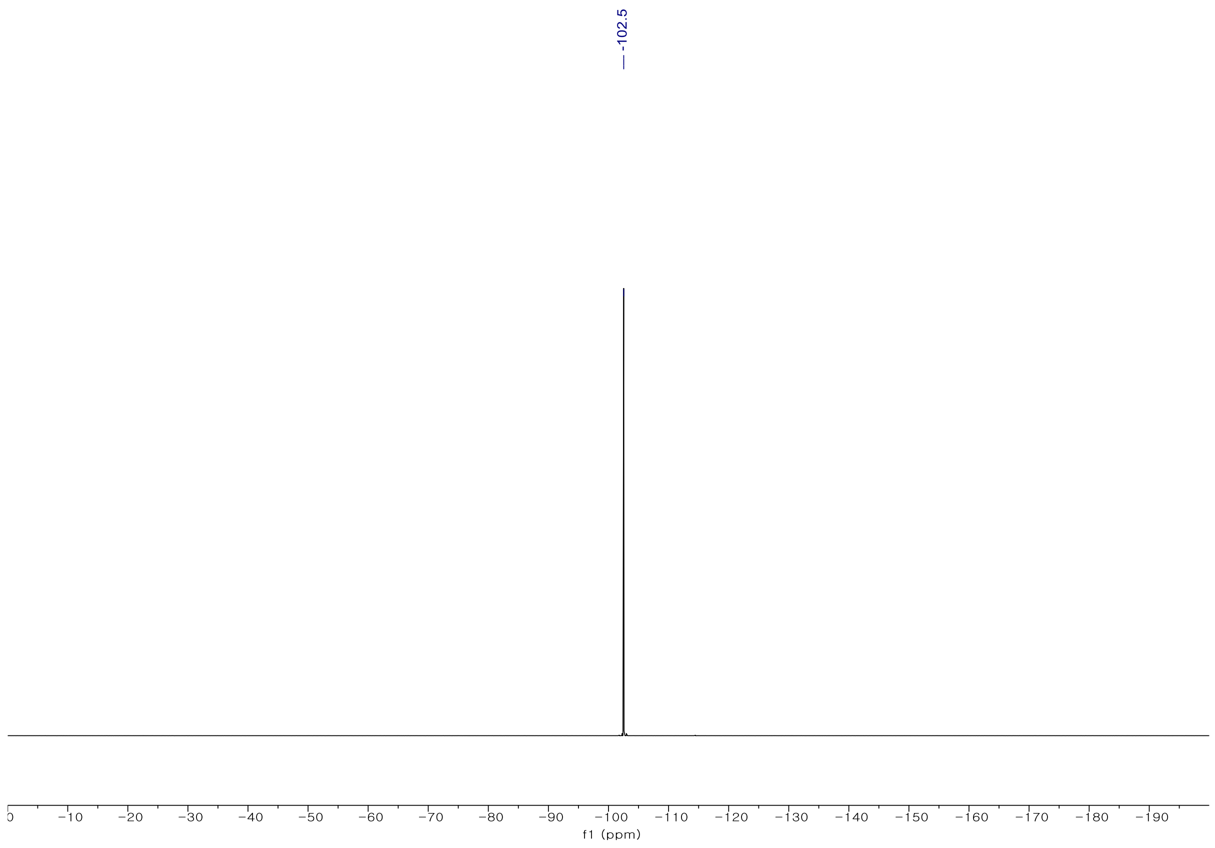
-102.5
0 −10 −20 −30 −40 −50 −60 −70 −80 −90 −100 −110 −120 −130 −140 −150 −160 −170 −180 −190
f1 (ppm)

(*E*)-4-Methyl-2-(piperidin-1-yldiazenyl)benzaldehyde (1f)

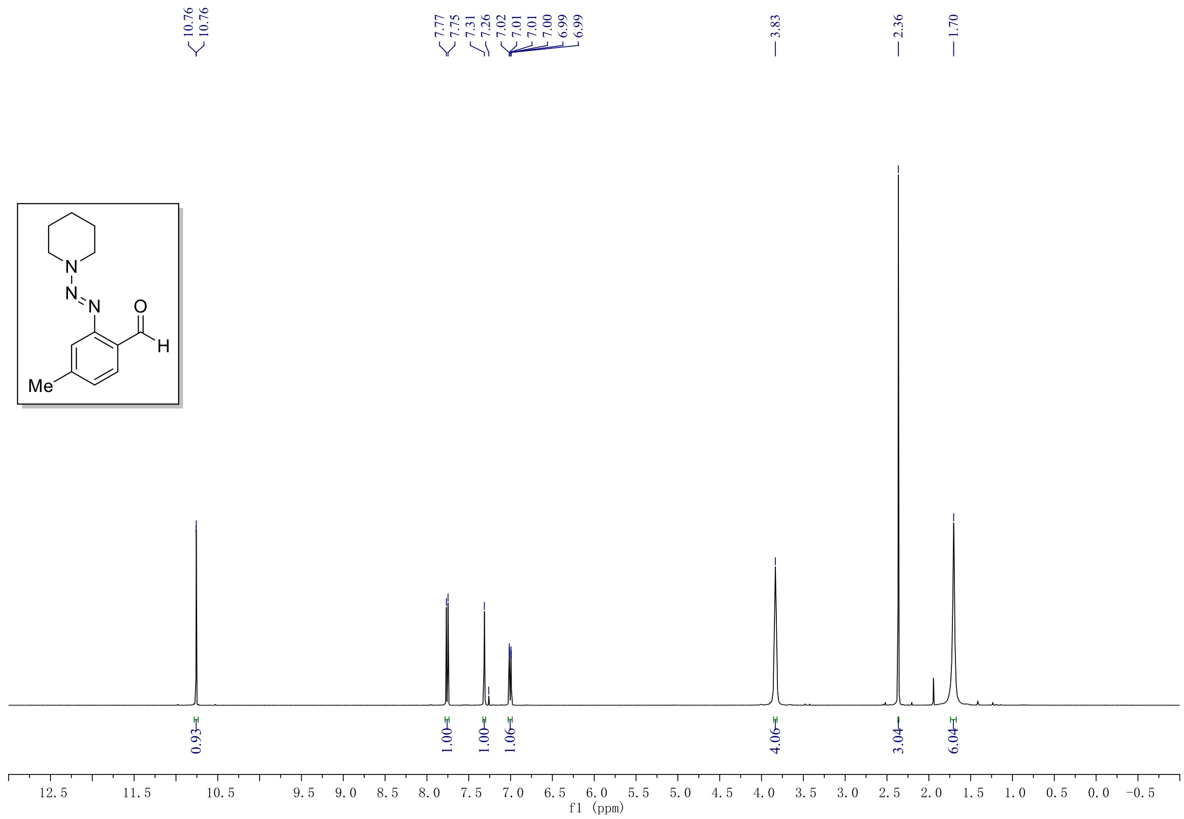

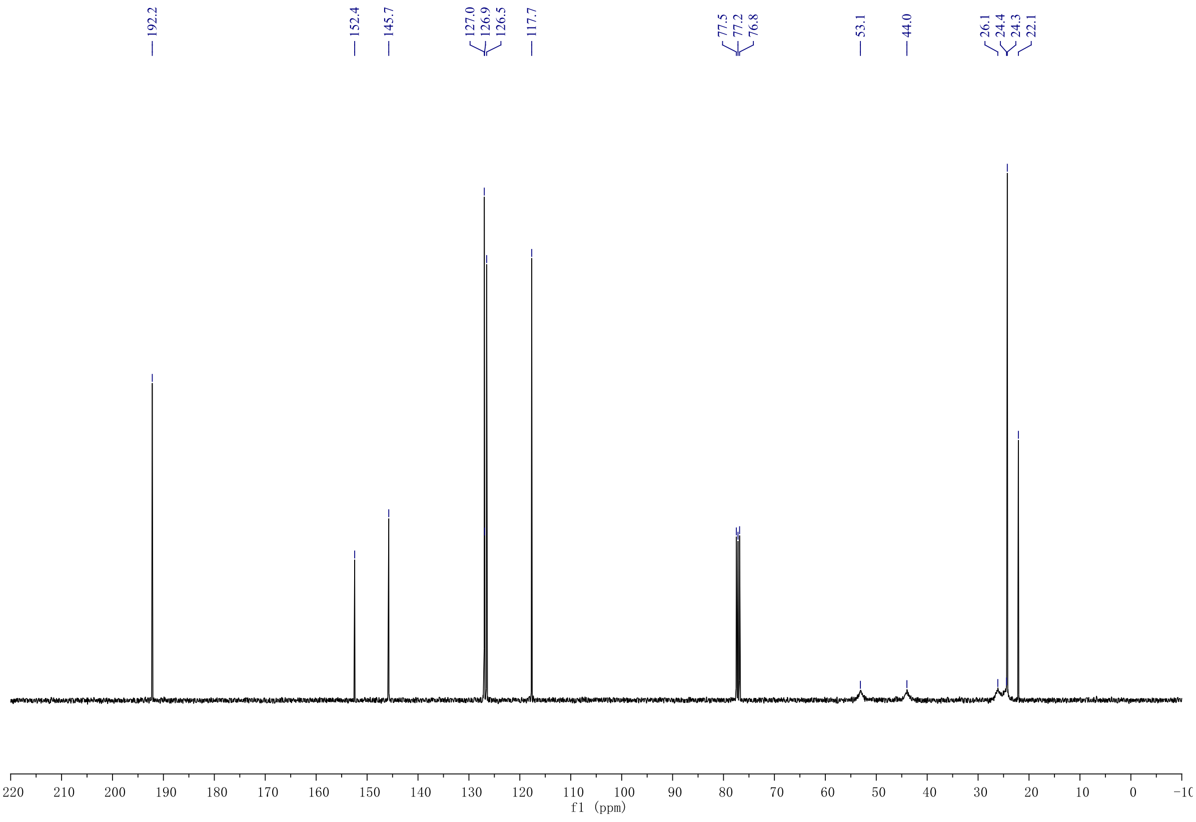

(*E*)-2-(Piperidin-1-yldiazenyl)-5-(trifluoromethoxy)benzaldehyde (1g)

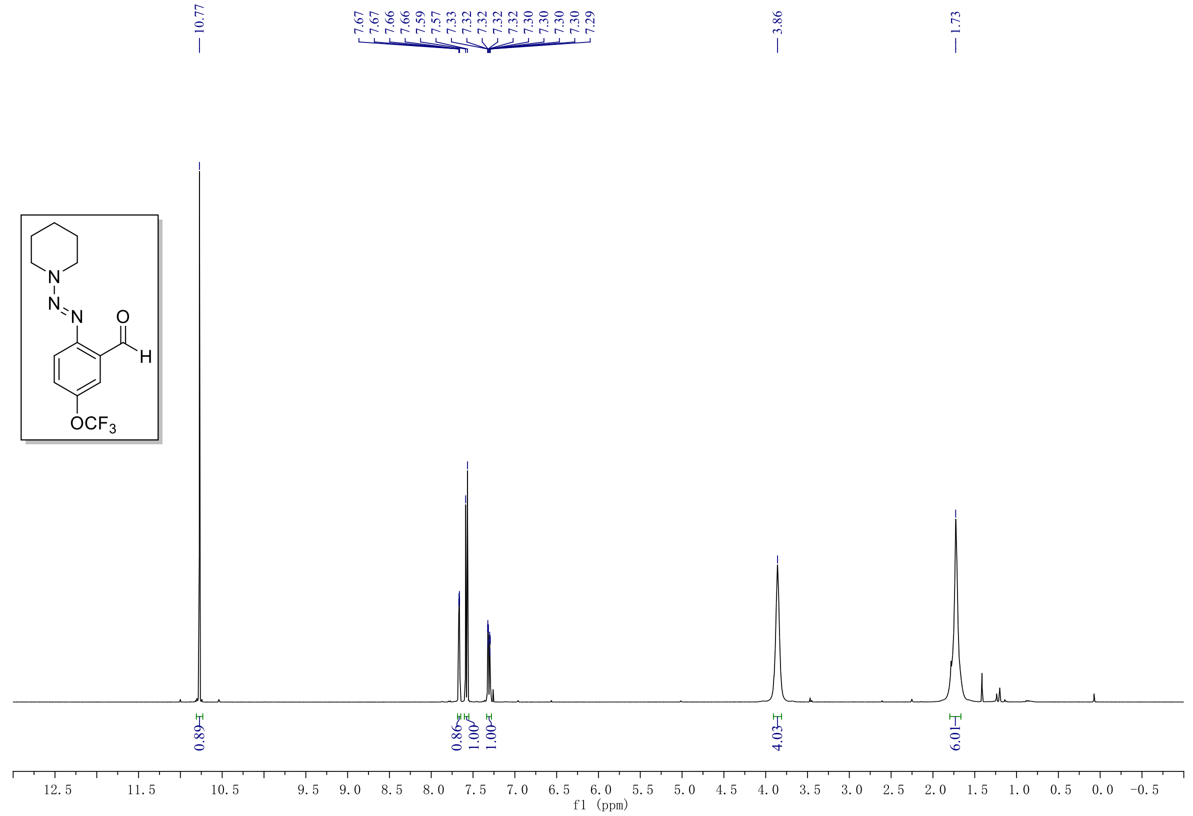

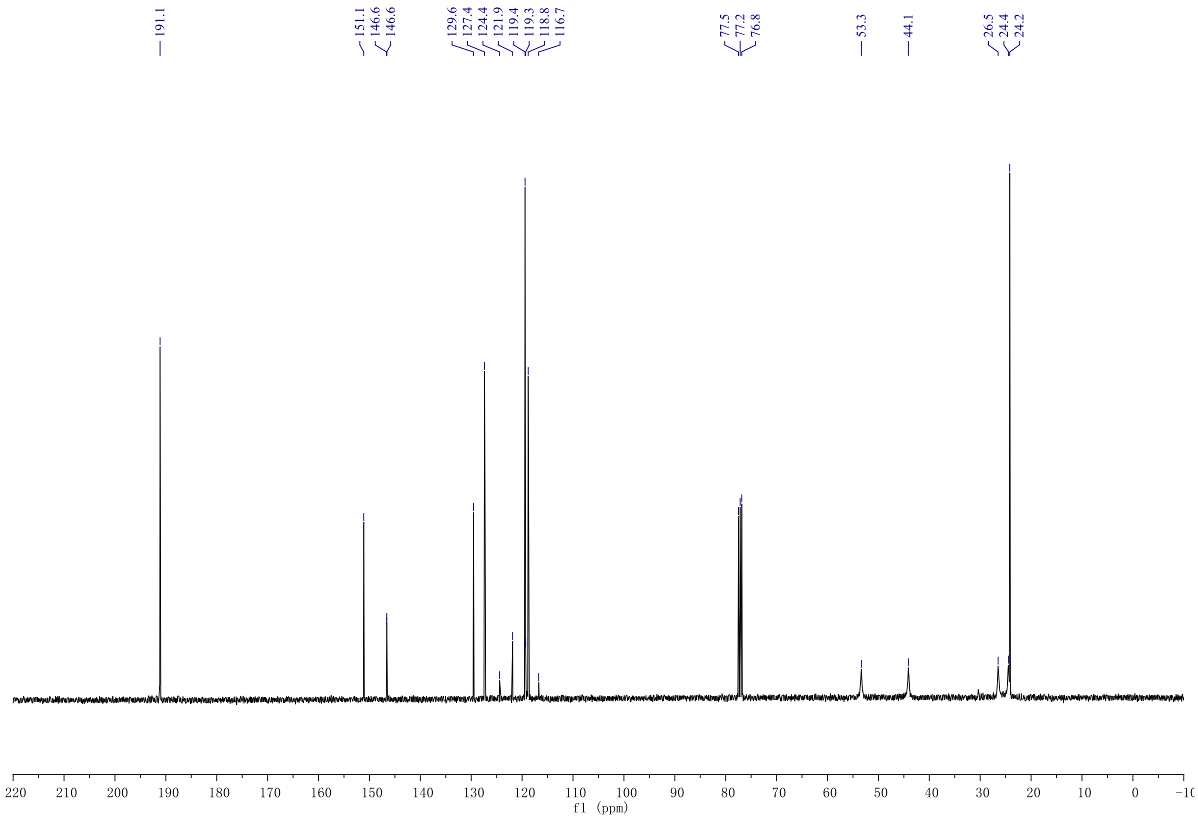

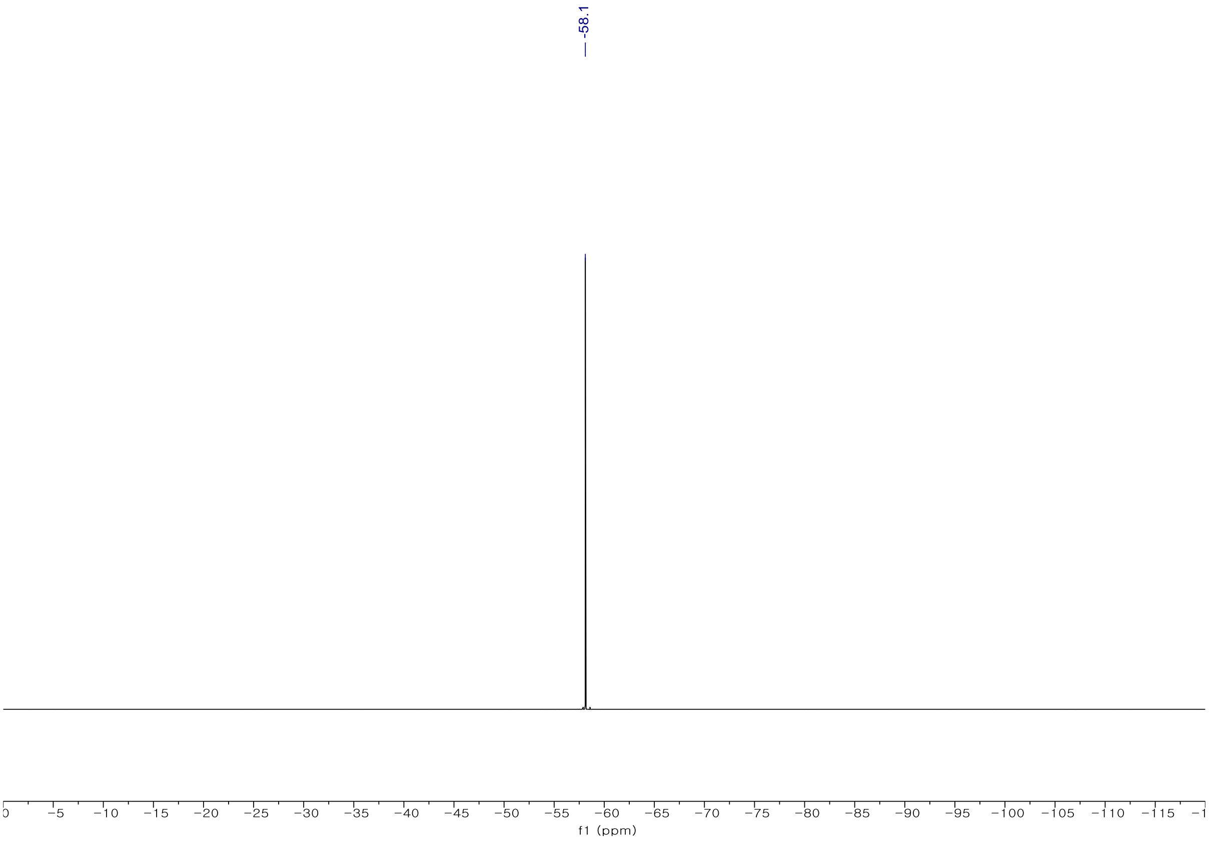
-58.1
f1 (ppm)

(*E*)-5-Fluoro-2-(piperidin-1-yldiazenyl)benzaldehyde (1h)

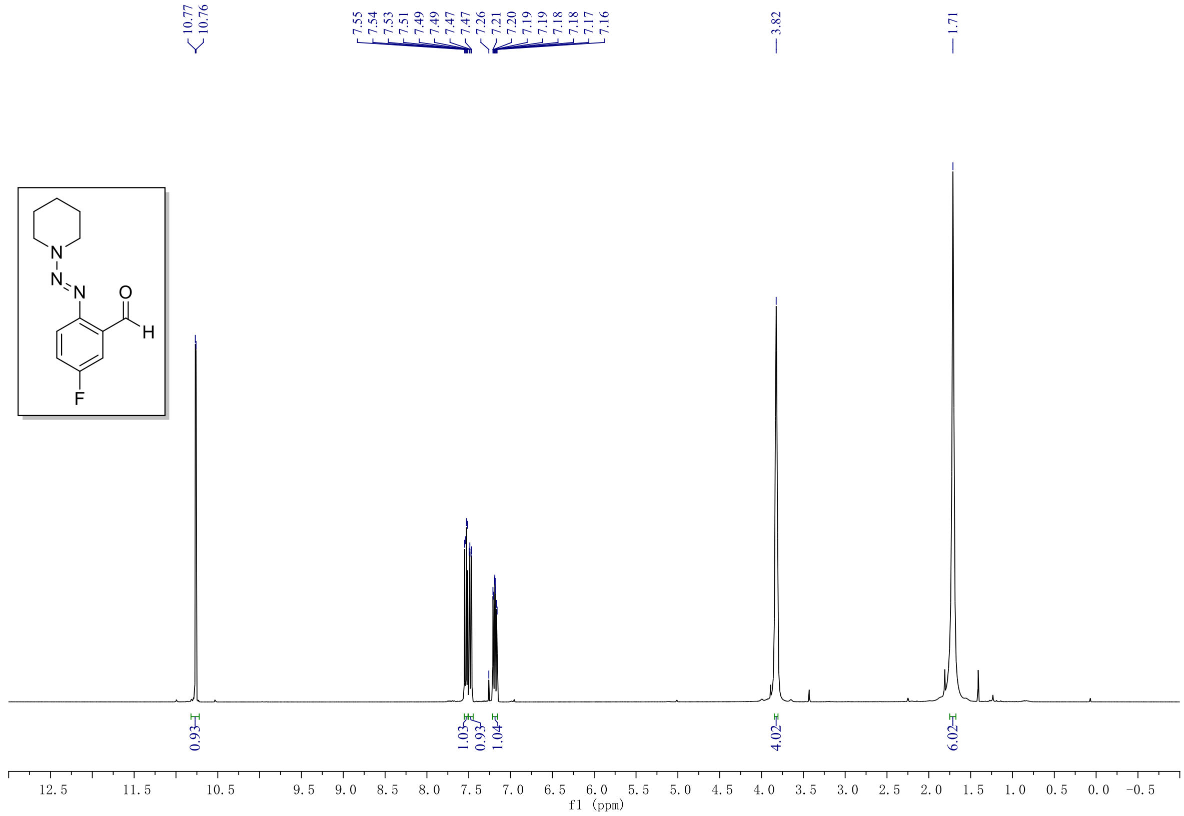

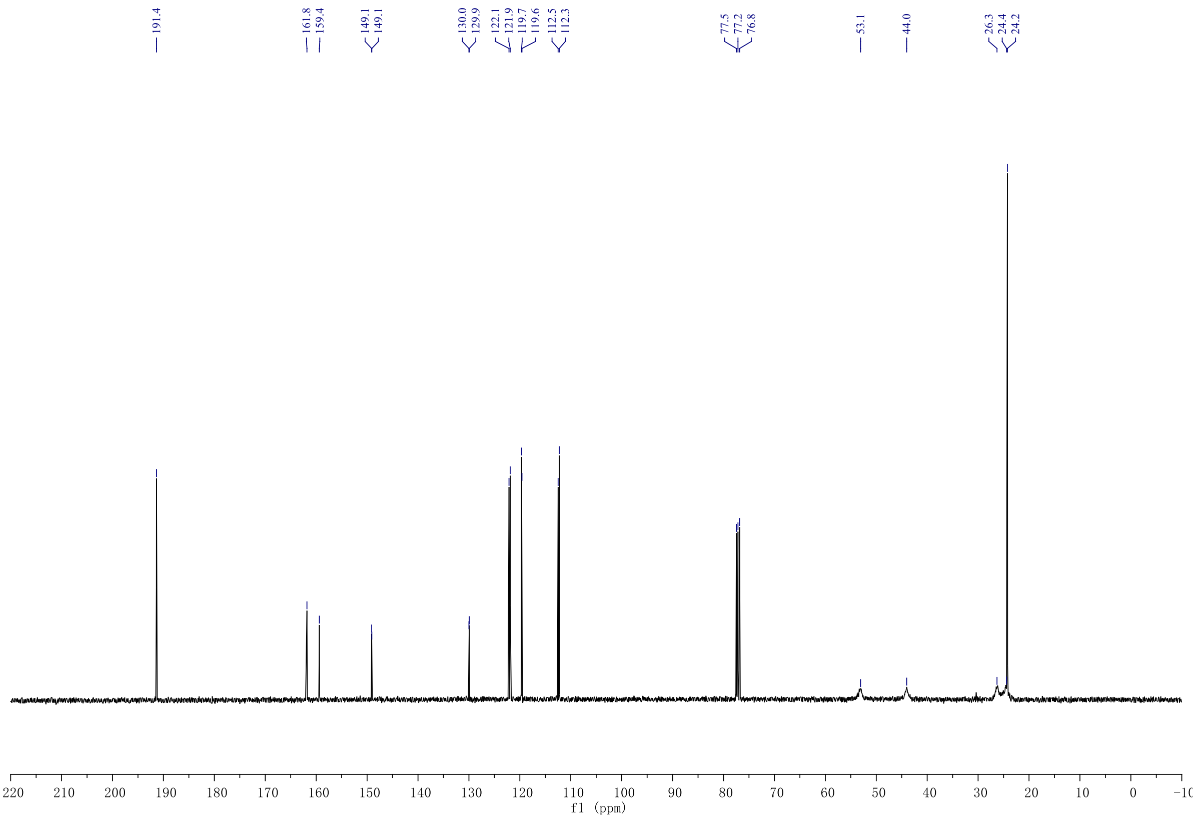

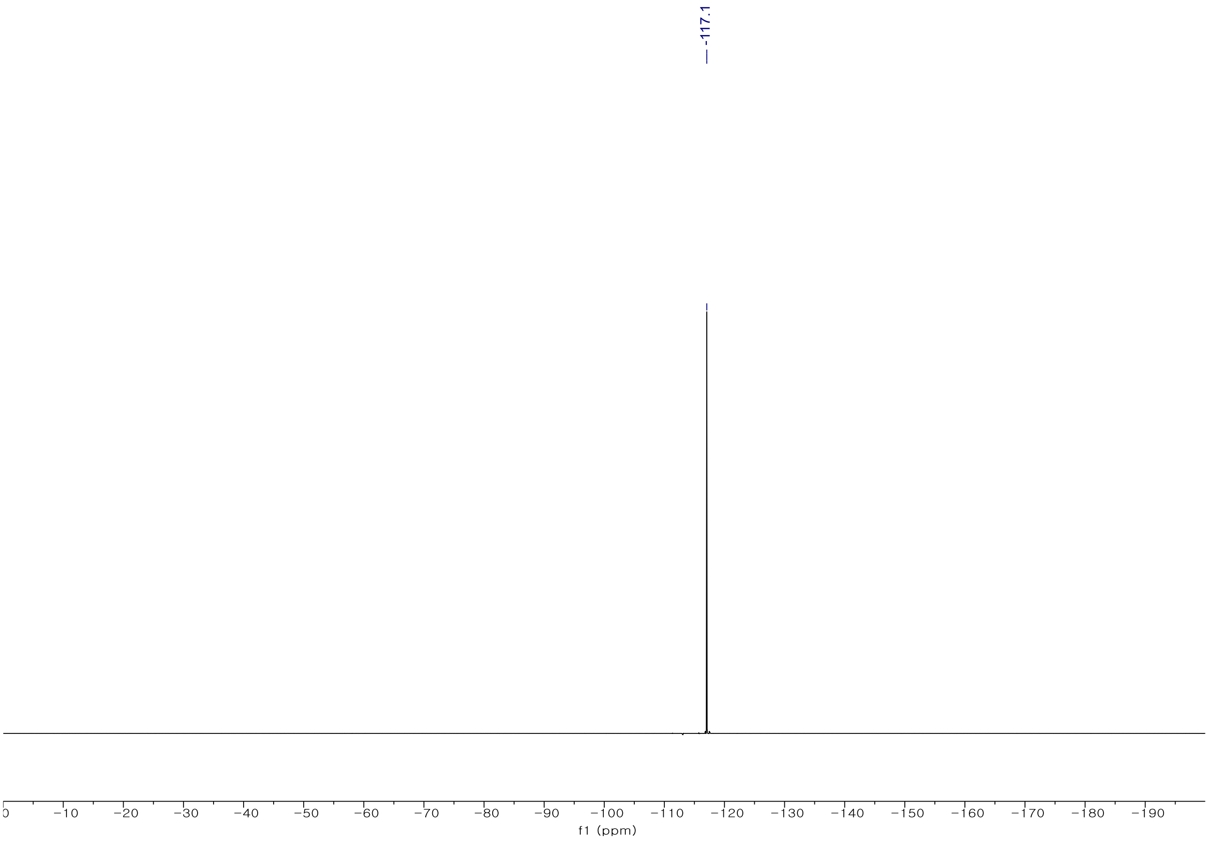

-117.1
0
-10
-20
-30
-40
-50
-60
-70
-80
-90
-100
-110
-120
-130
-140
-150
-160
-170
-180
-190
f1 (ppm)

(*E*)-5-Methyl-2-(piperidin-1-yldiazenyl)benzaldehyde (1i)

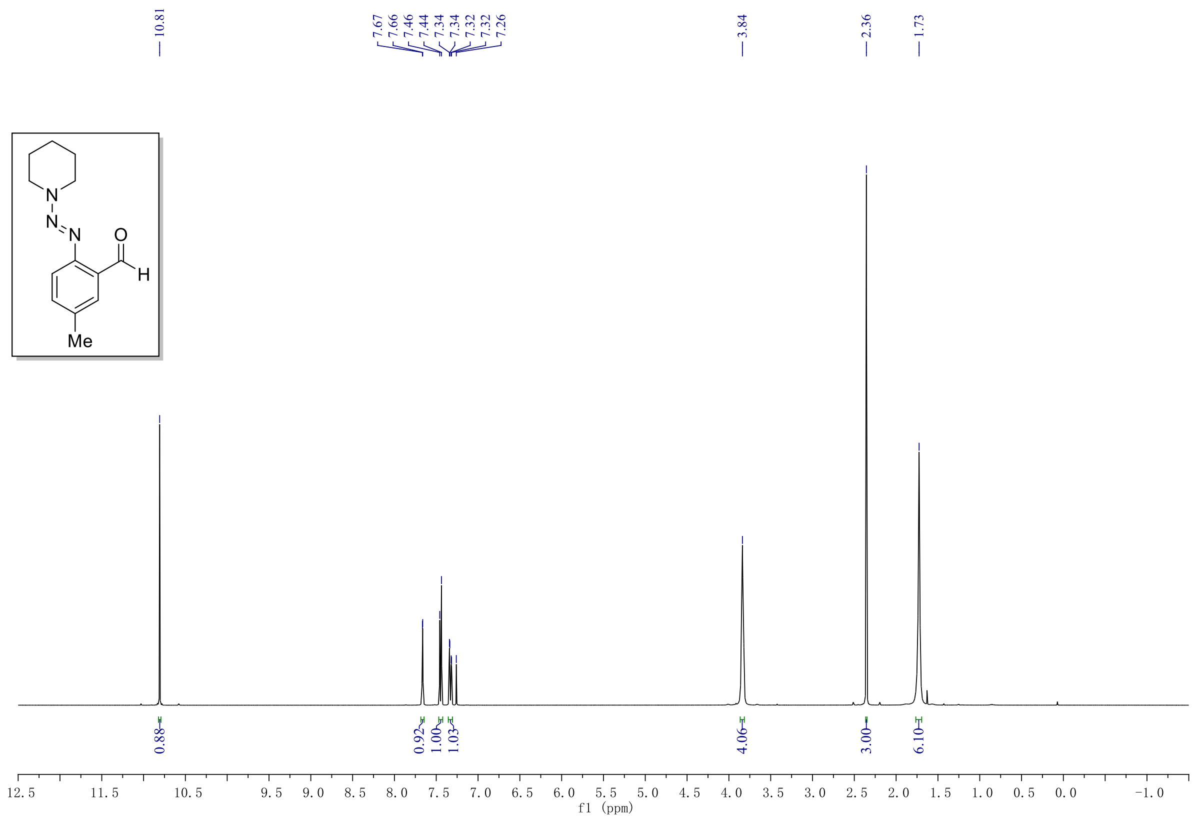

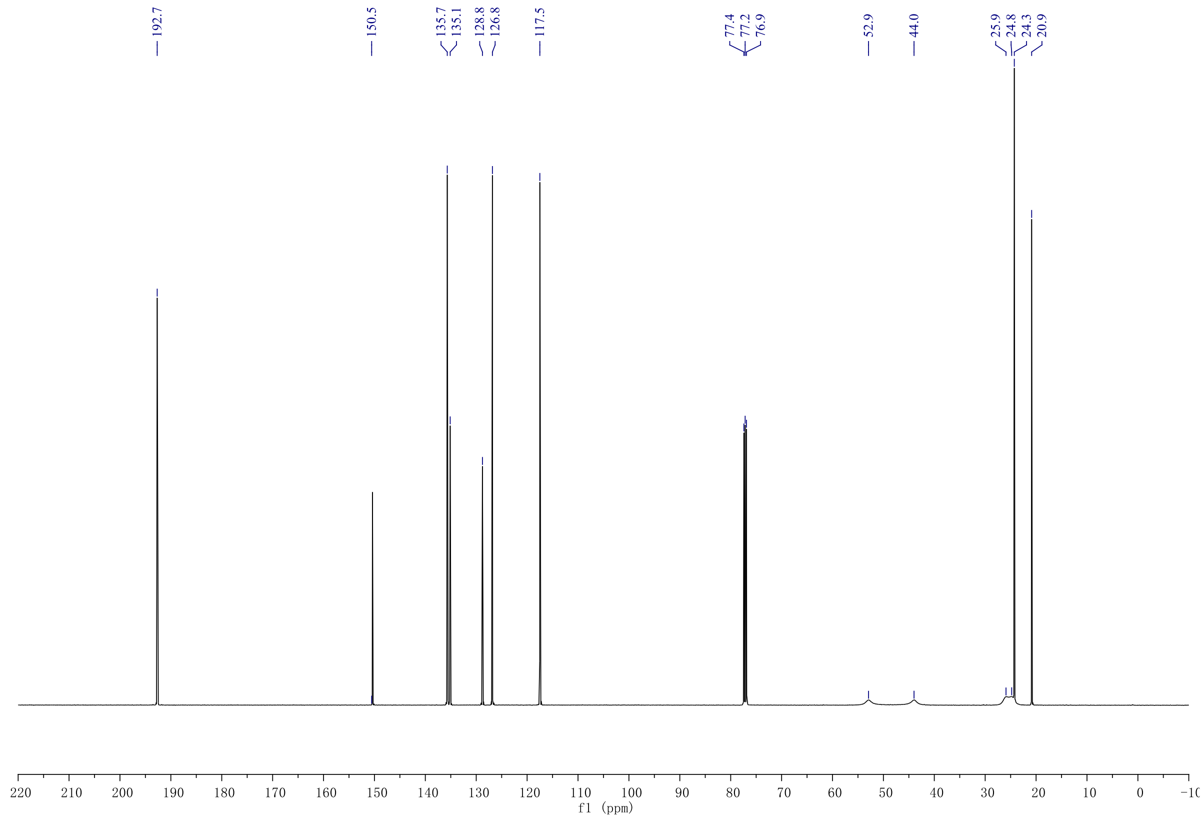

(*E*)-3-(Piperidin-1-yldiazenyl)-2-naphthaldehyde (1j)

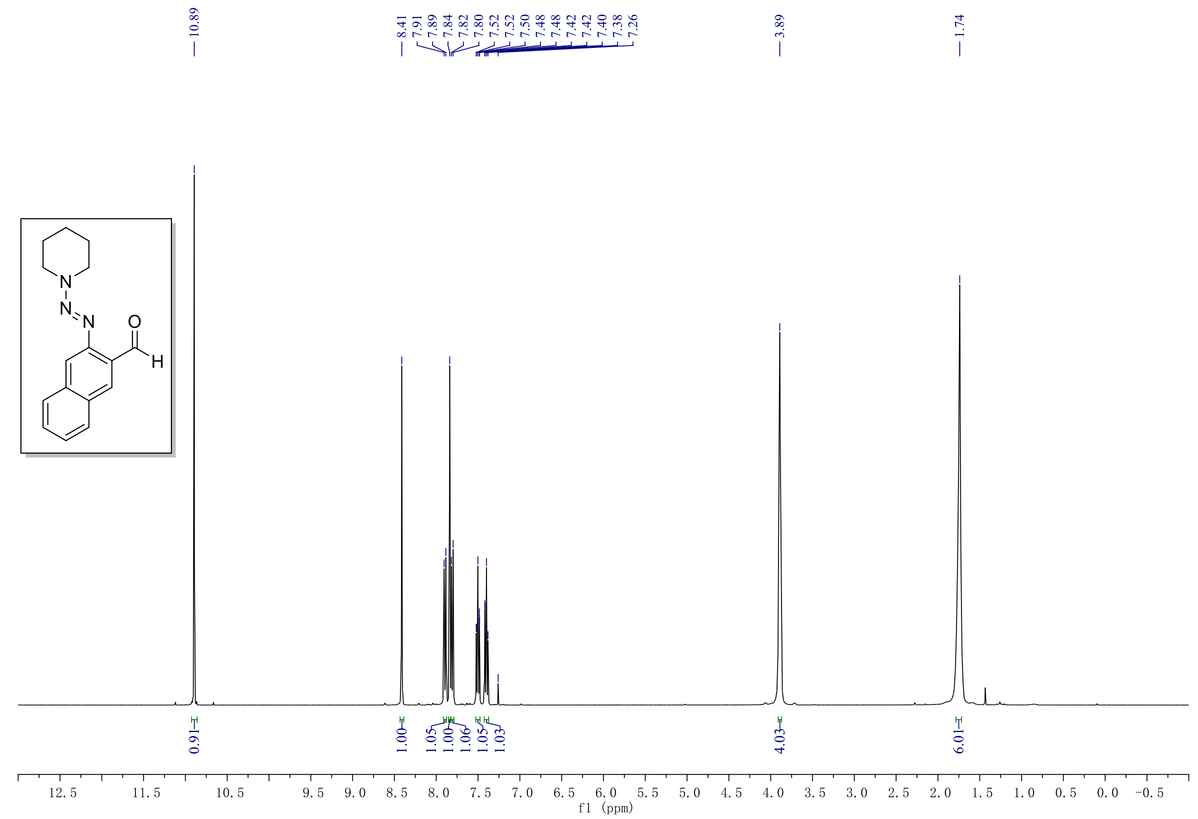

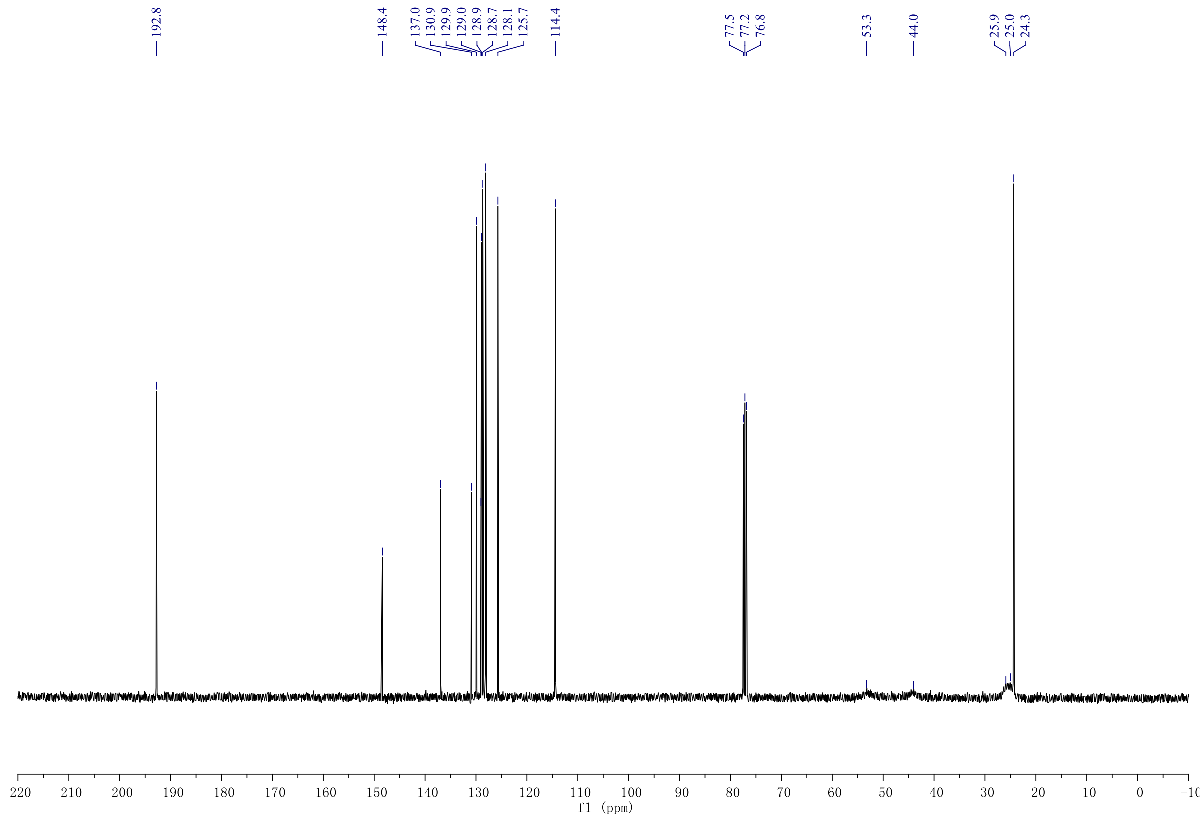

(*E*)-[2-Fluoro-6-(piperidin-1-yldiazenyl)phenyl]methanol

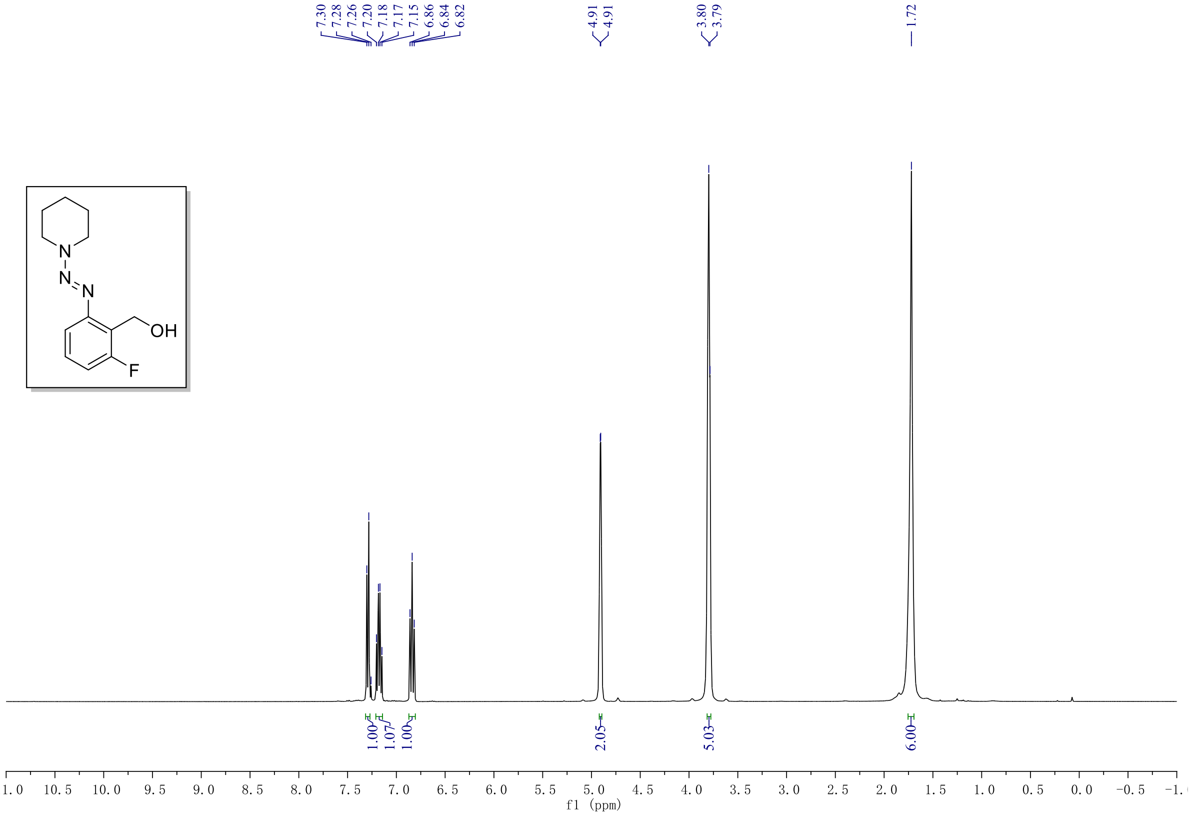

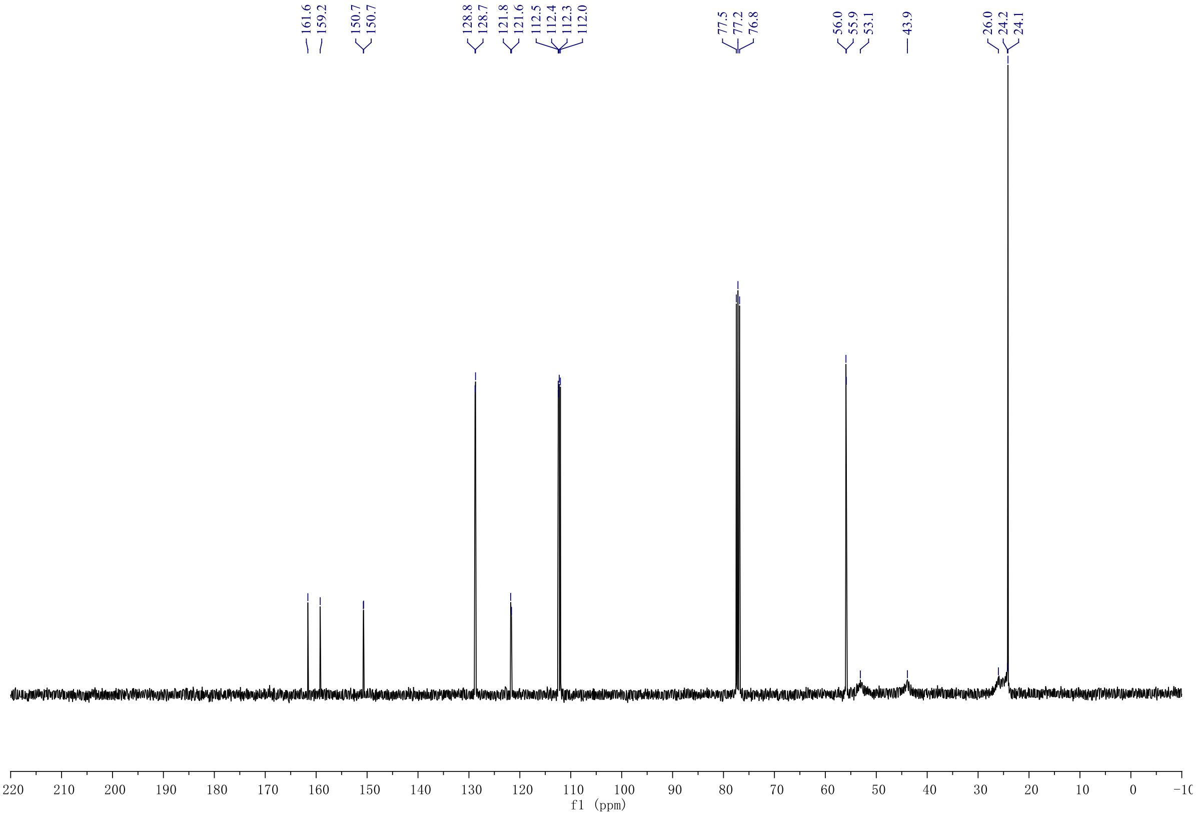

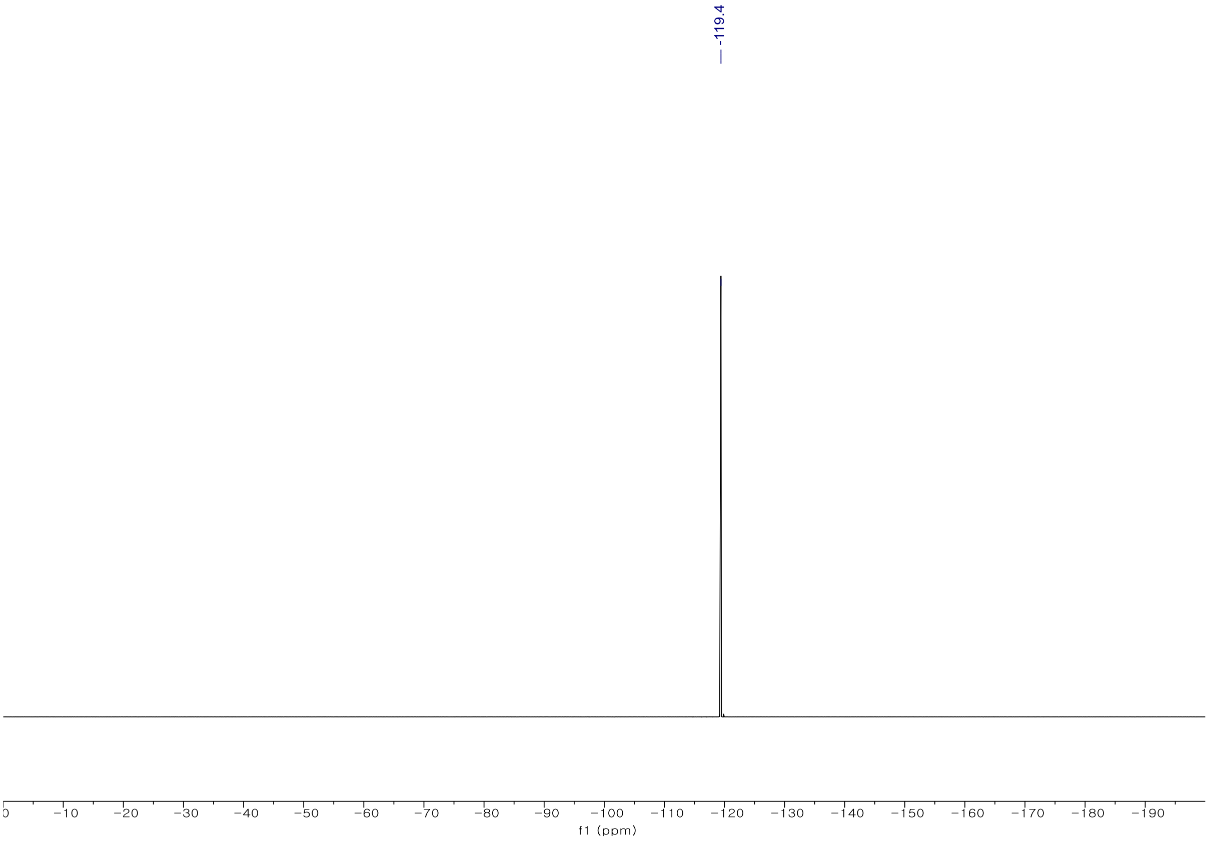
-119.4
0
−10
−20
−30
−40
−50
−60
−70
−80
−90
−100
−110
−120
−130
−140
−150
−160
−170
−180
−190
f1 (ppm)

(*E*)-2-Fluoro-6-(piperidin-1-yldiazenyl)benzaldehyde (1k)

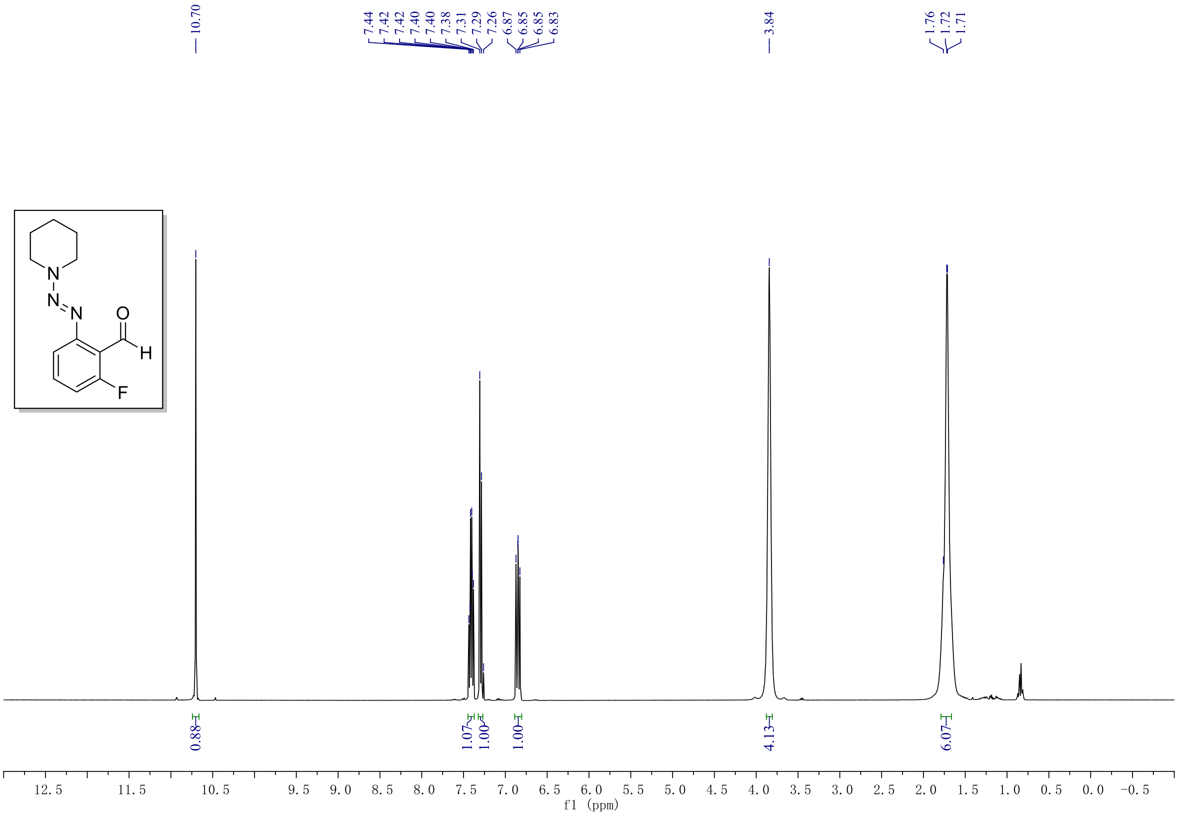

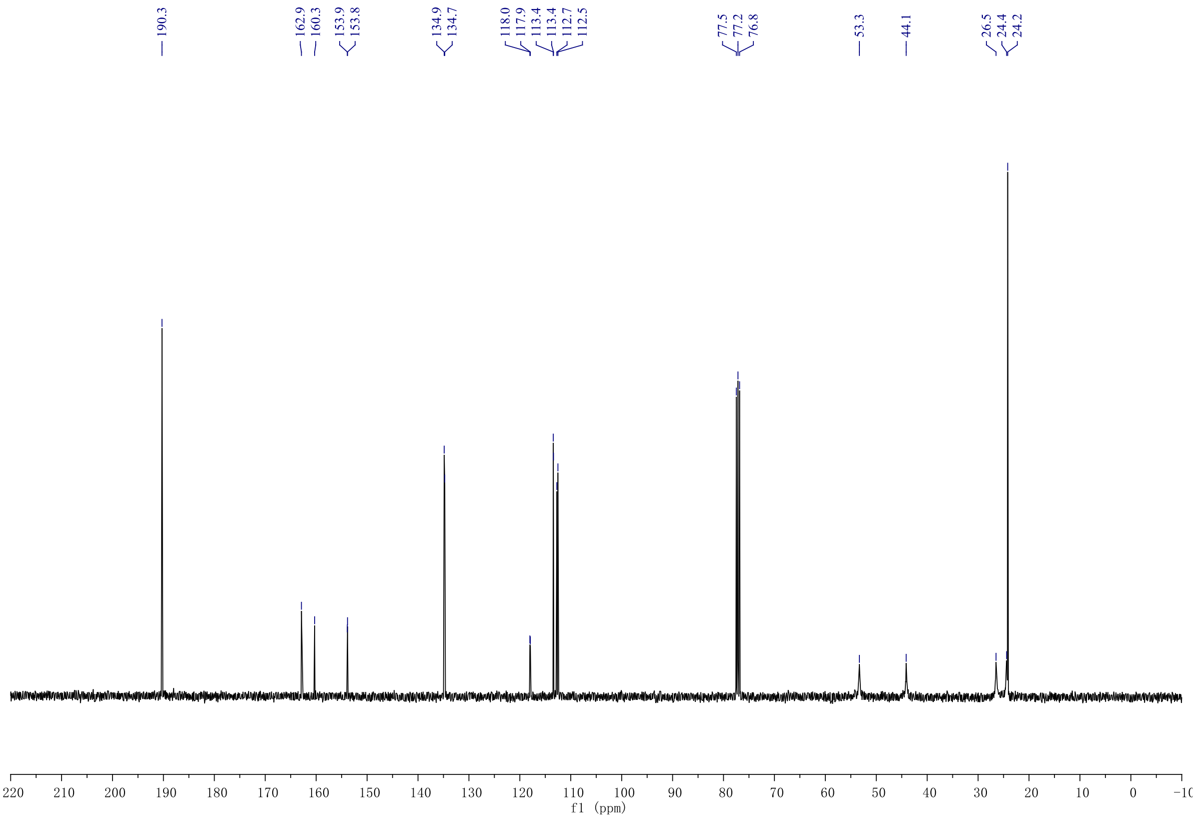

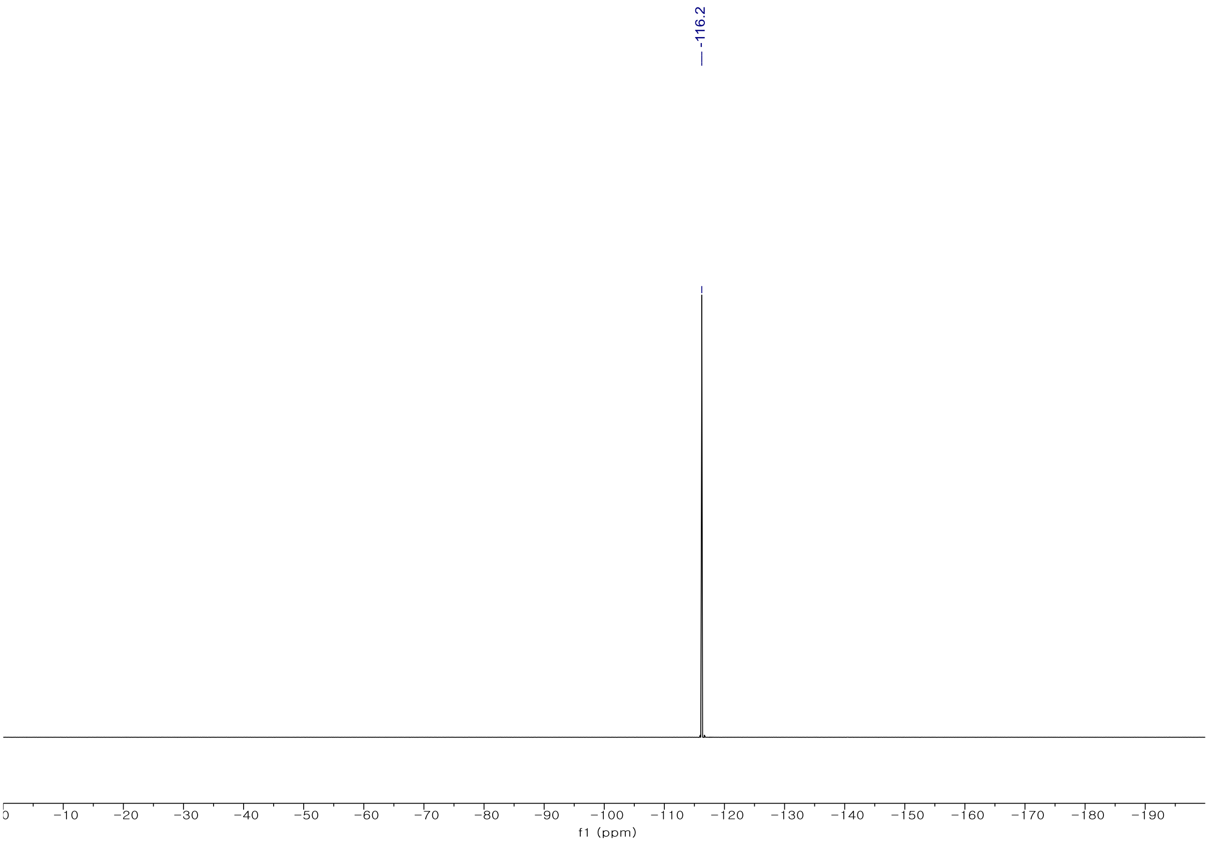
-116.2
0
−10
−20
−30
−40
−50
−60
−70
−80
−90
−100
−110
−120
−130
−140
−150
−160
−170
−180
−190
f1 (ppm)

(*E*)-1-{2-[(*E*)-3,3-diethyltriaz-1-en-1-yl]phenyl}non-2-en-1-one (3a’)

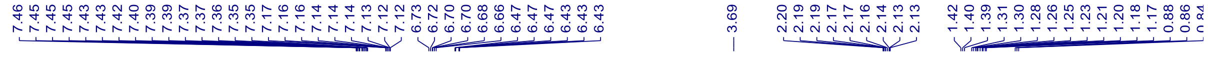

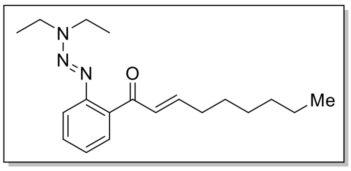

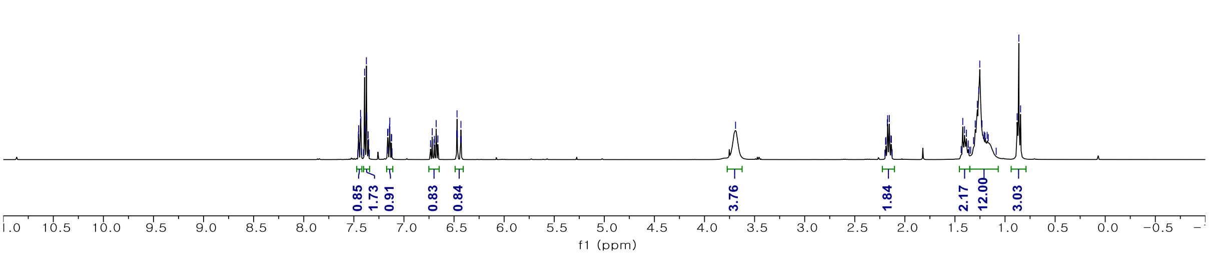

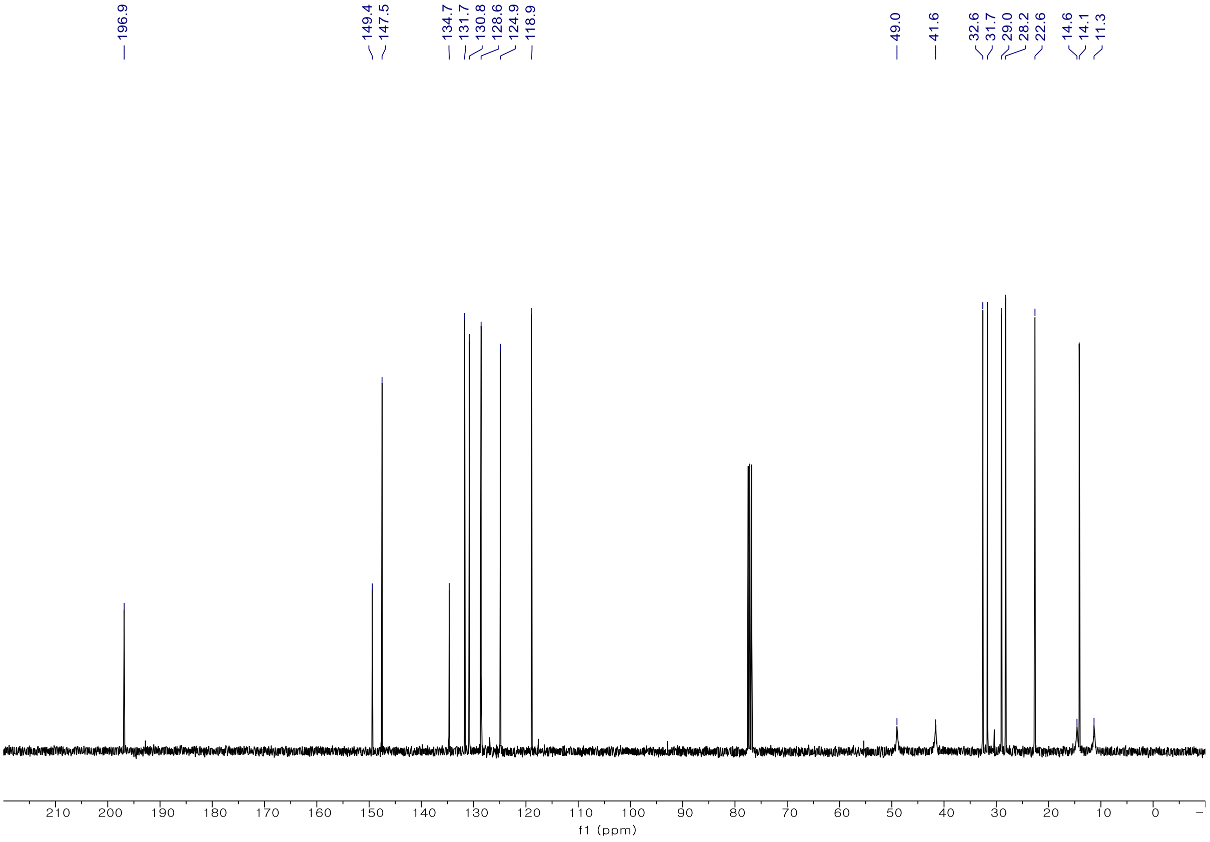

(*E*)-1-{2-[(*E*)-Piperidin-1-yldiazenyl]phenyl}non-2-en-1-one (3a)

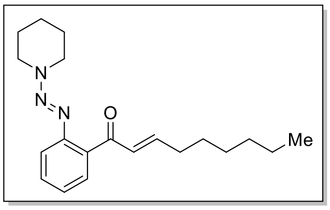

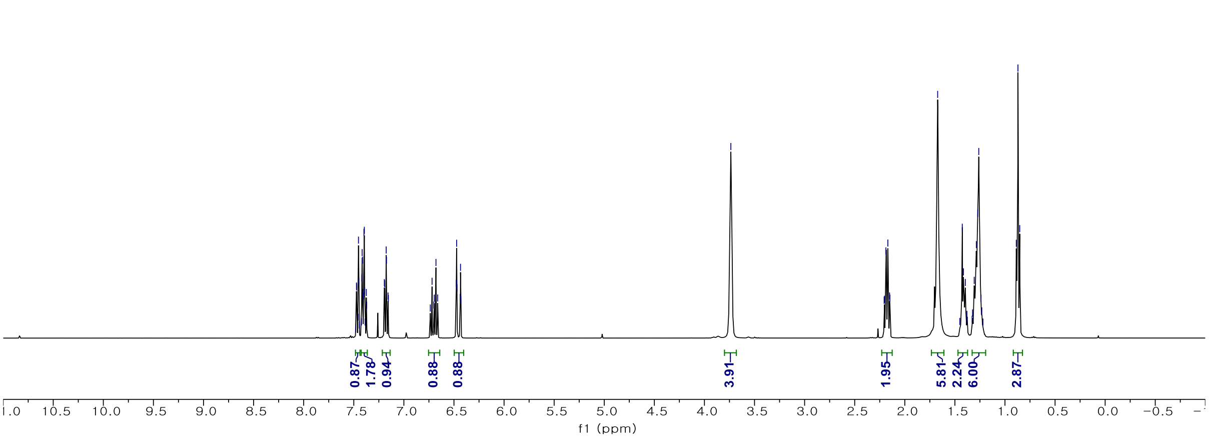

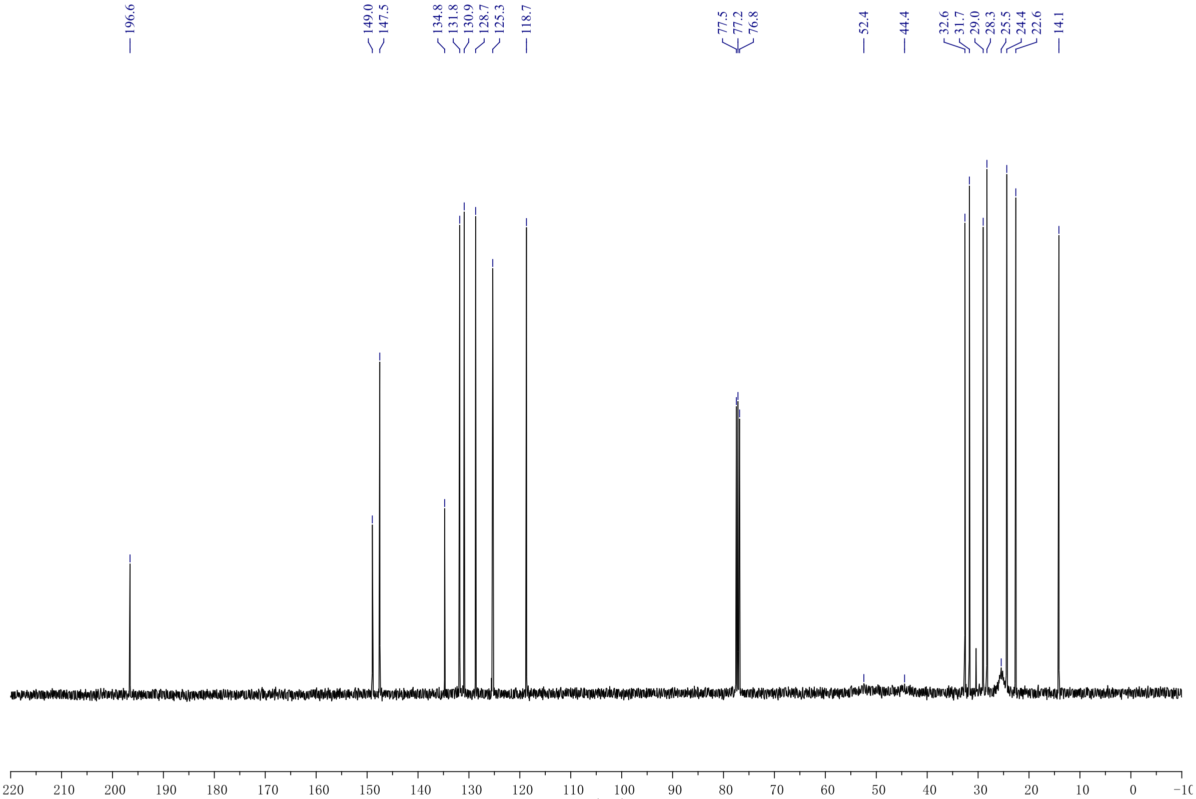

(*E*)-4,4-Dimethyl-1-{2-[(*E*)-piperidin-1-yldiazenyl]phenyl}pent-2-en-1-one (3b)

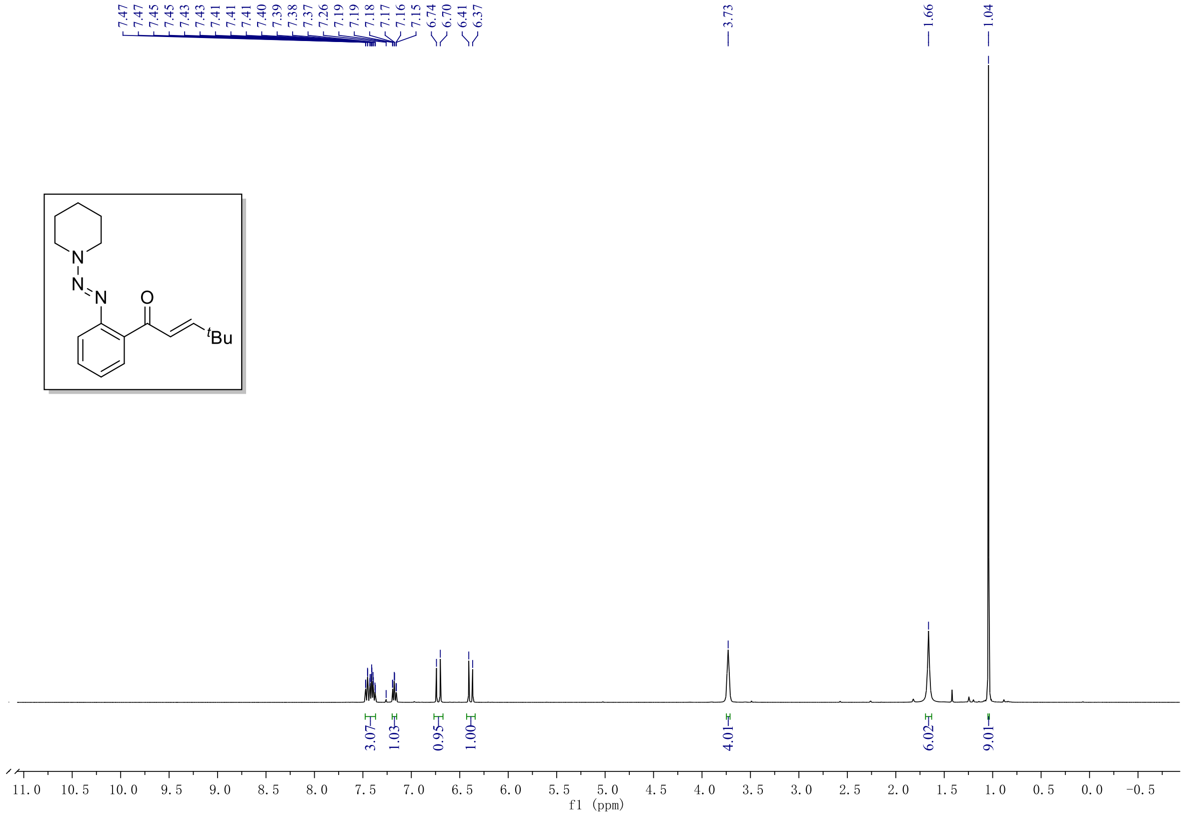

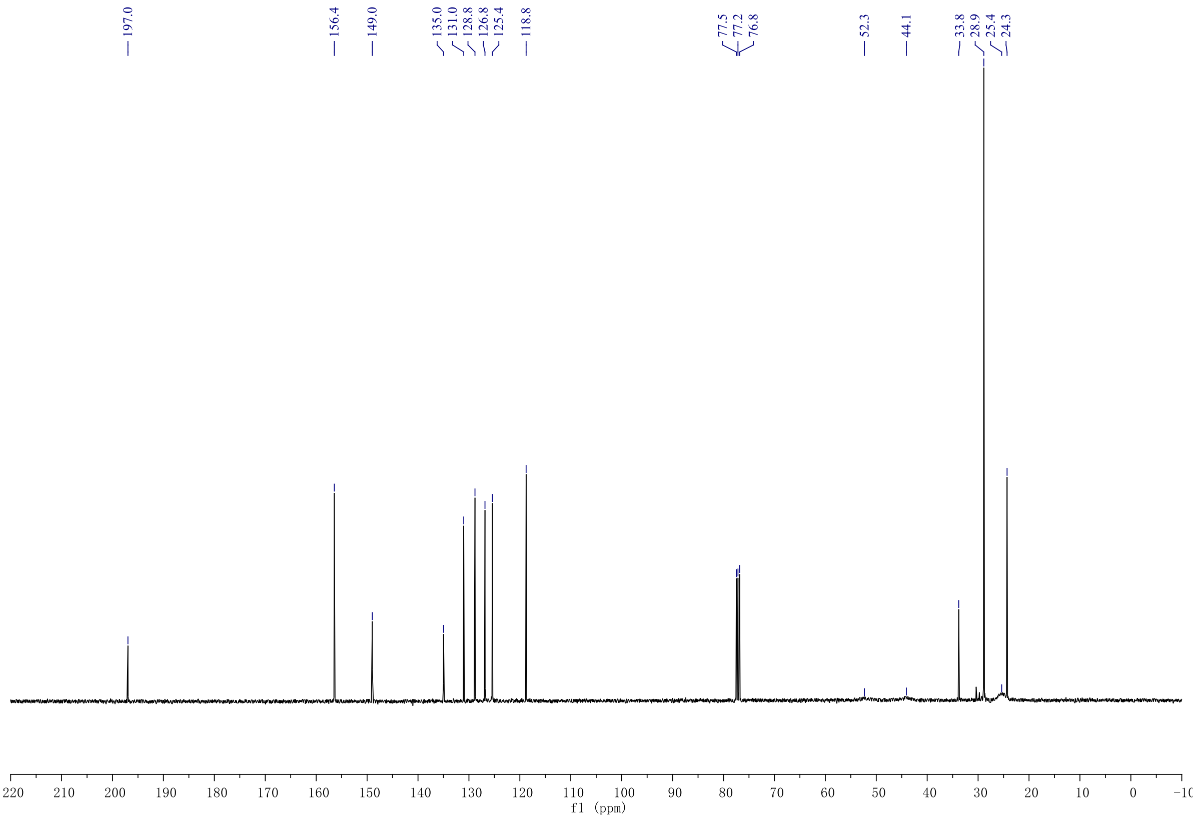

(*E*)-3-Phenyl-1-{2-[(*E*)-piperidin-1-yldiazenyl]phenyl}prop-2-en-1-one (3c)

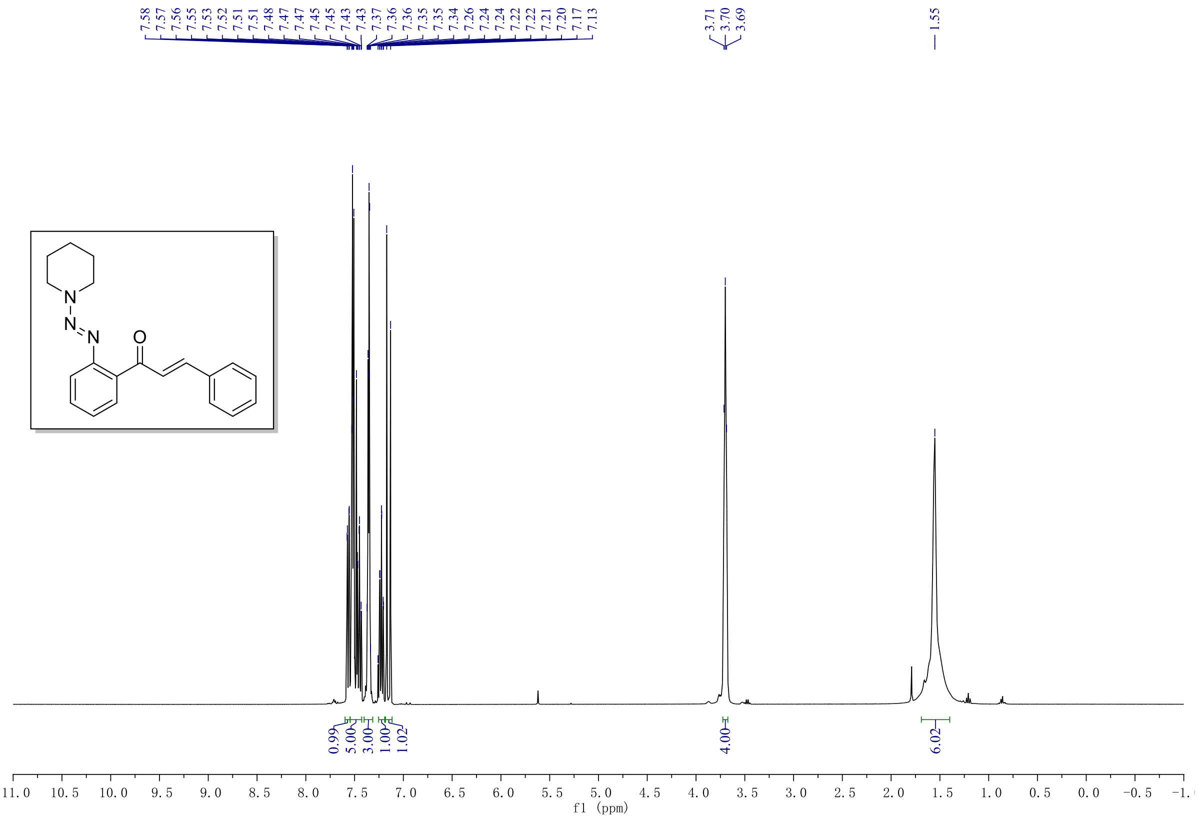

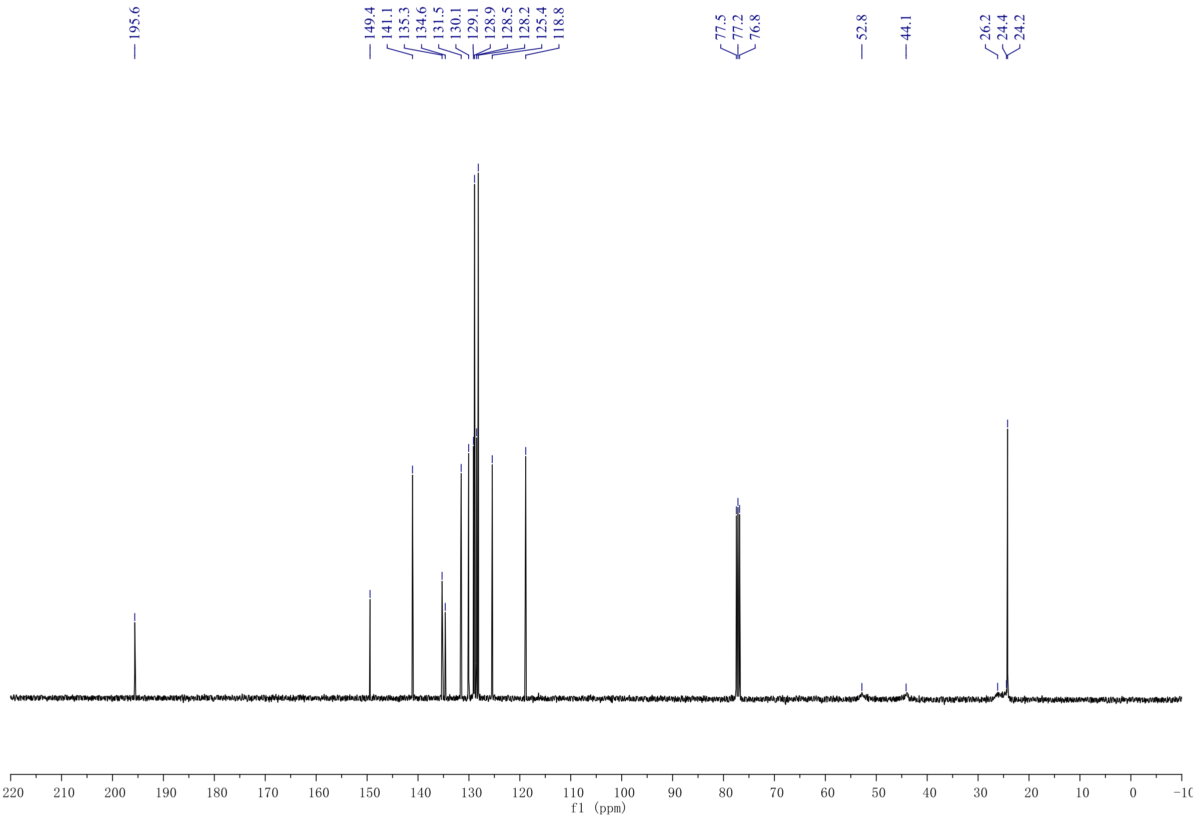

(*E*)-3-Ferrocenyl-1-phenylprop-2-en-1-one (4h)

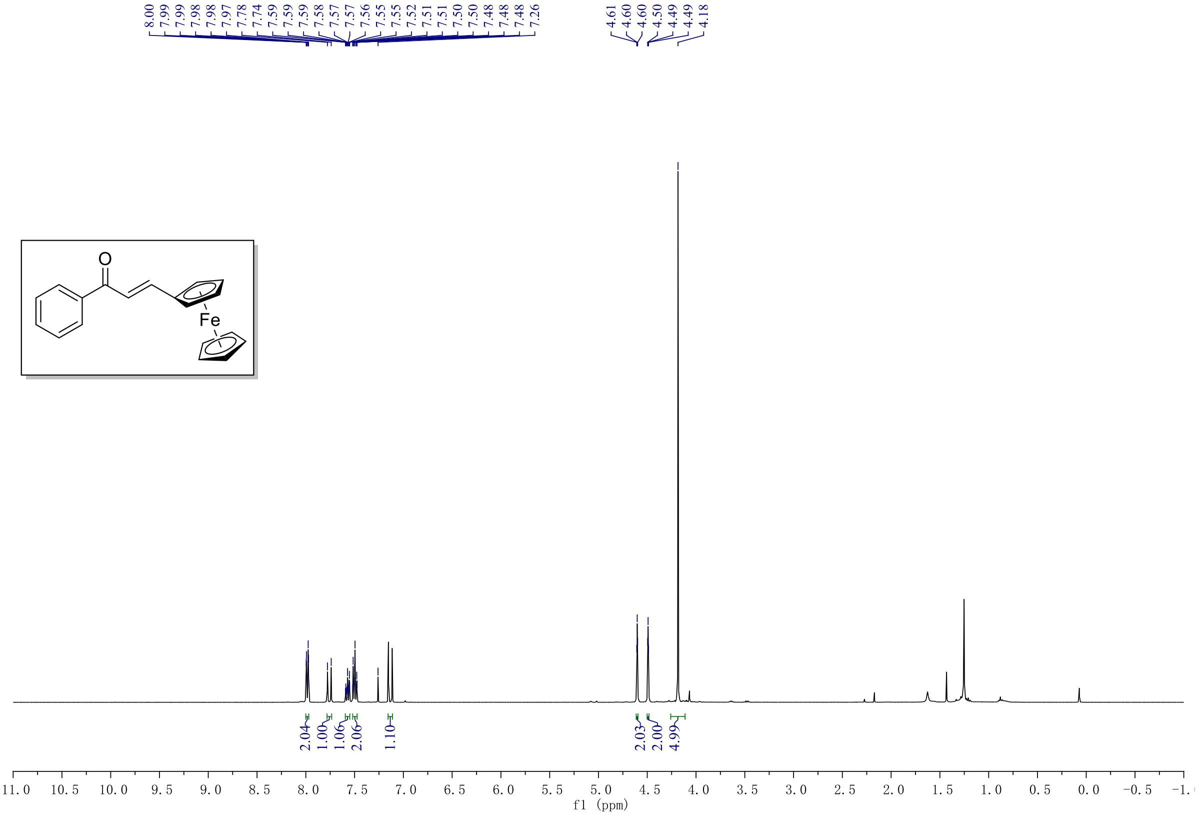

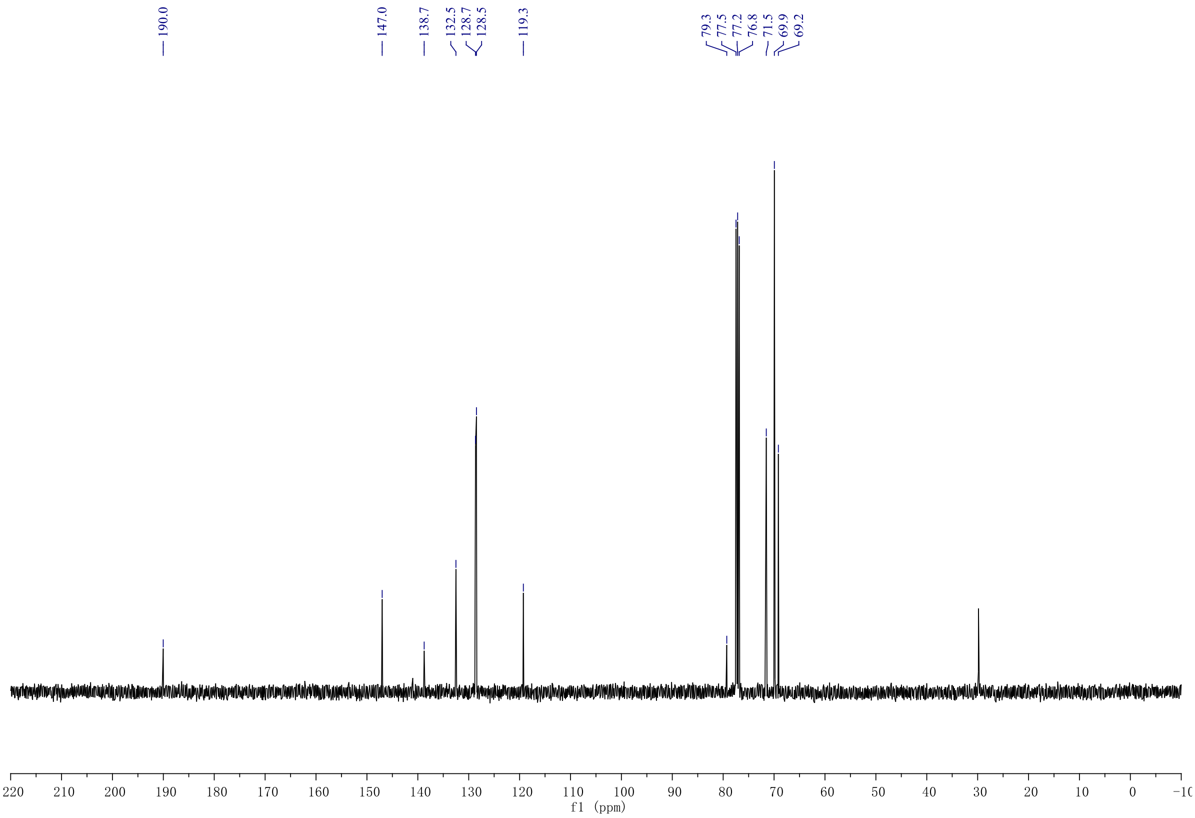

(*E*)-3-Cyclopentyl-1-[4-(trifluoromethyl)phenyl]prop-2-en-1-one (4j)

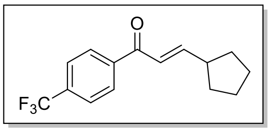

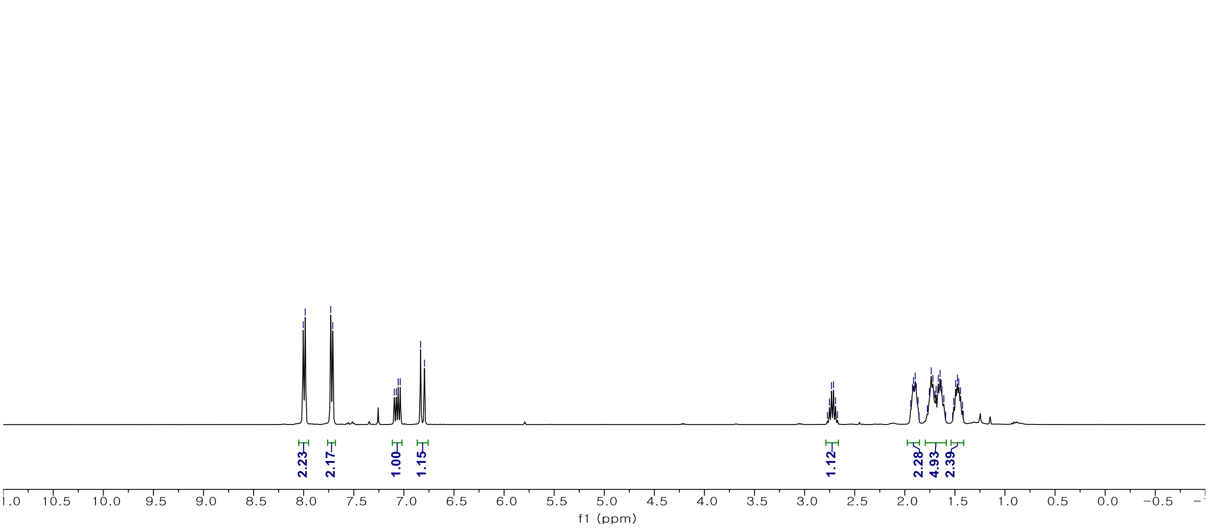

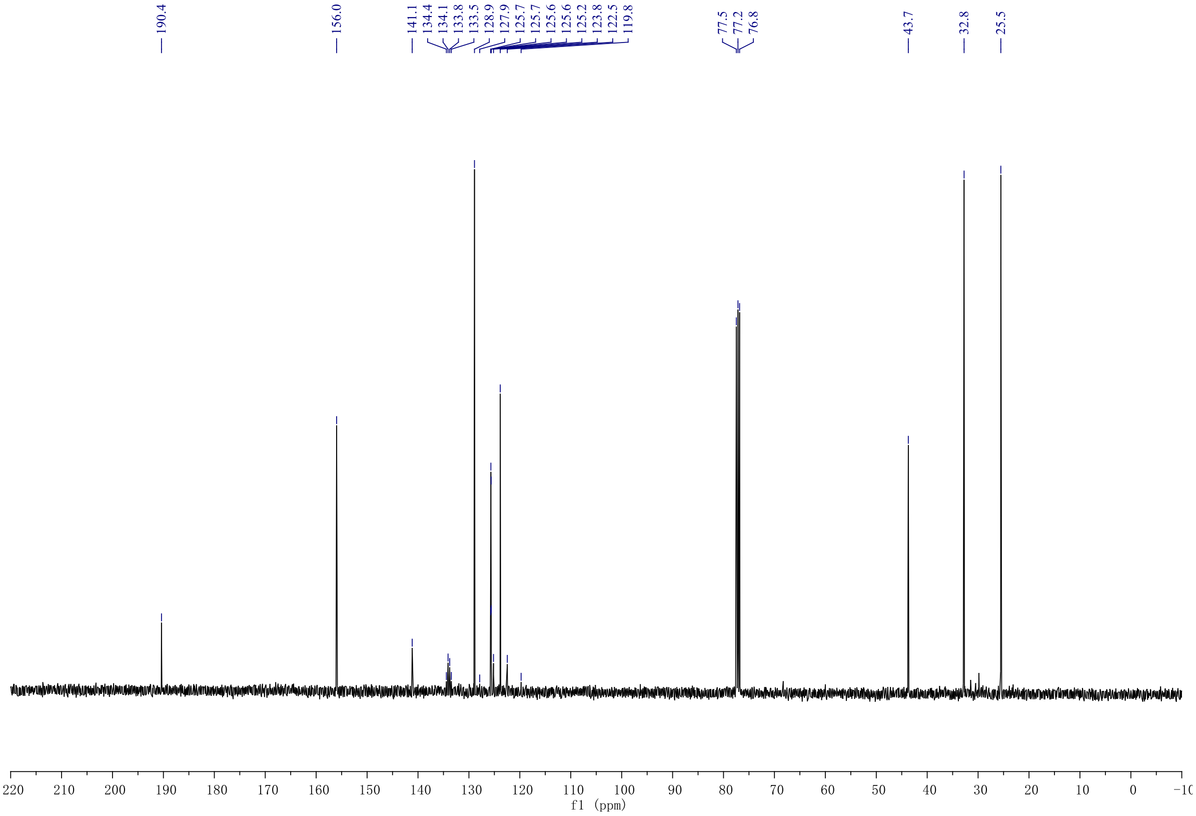

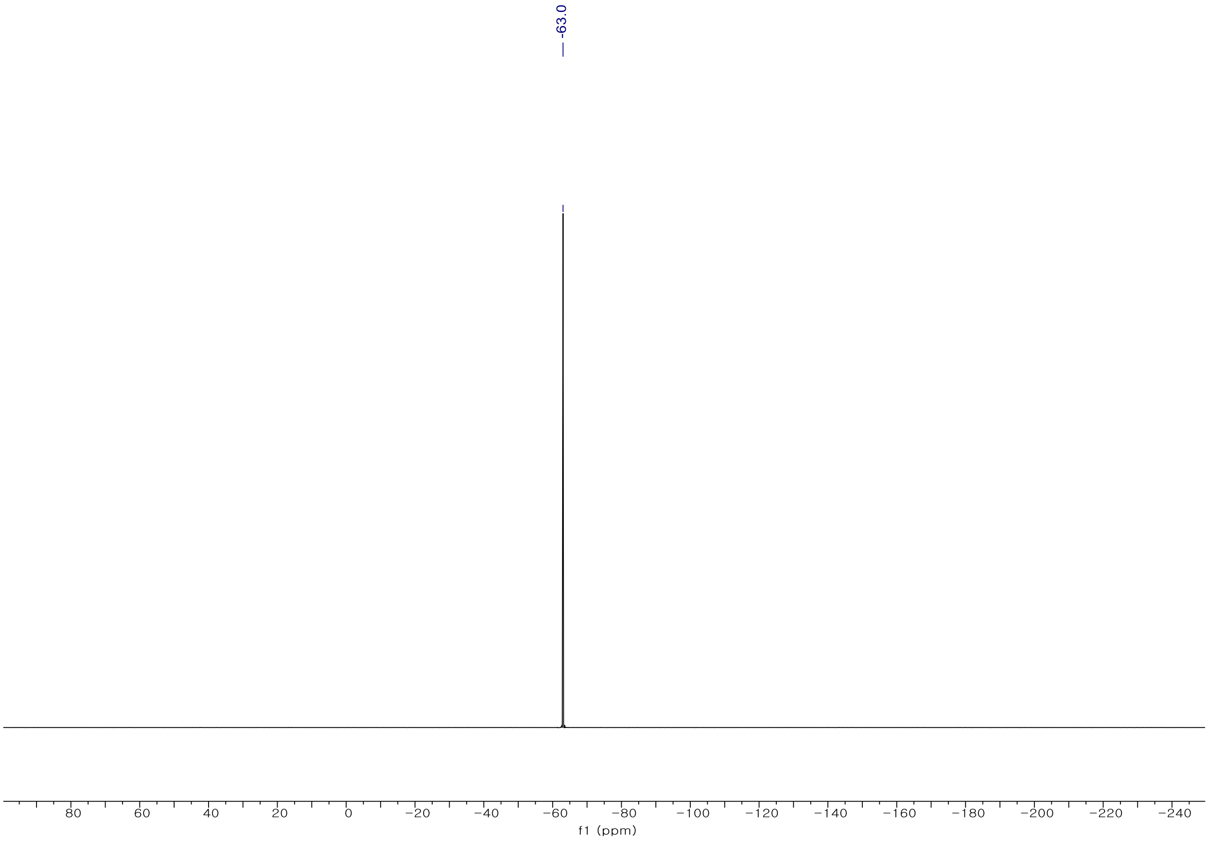
-63.0
80
60
40
20
0
−20
−40
−60
−80
−100
−120
−140
−160
−180
−200
−220
−240
f1 (ppm)

(*E*)-3-Cyclopentyl-1-[3-(trifluoromethoxy)phenyl]prop-2-en-1-one (4k)

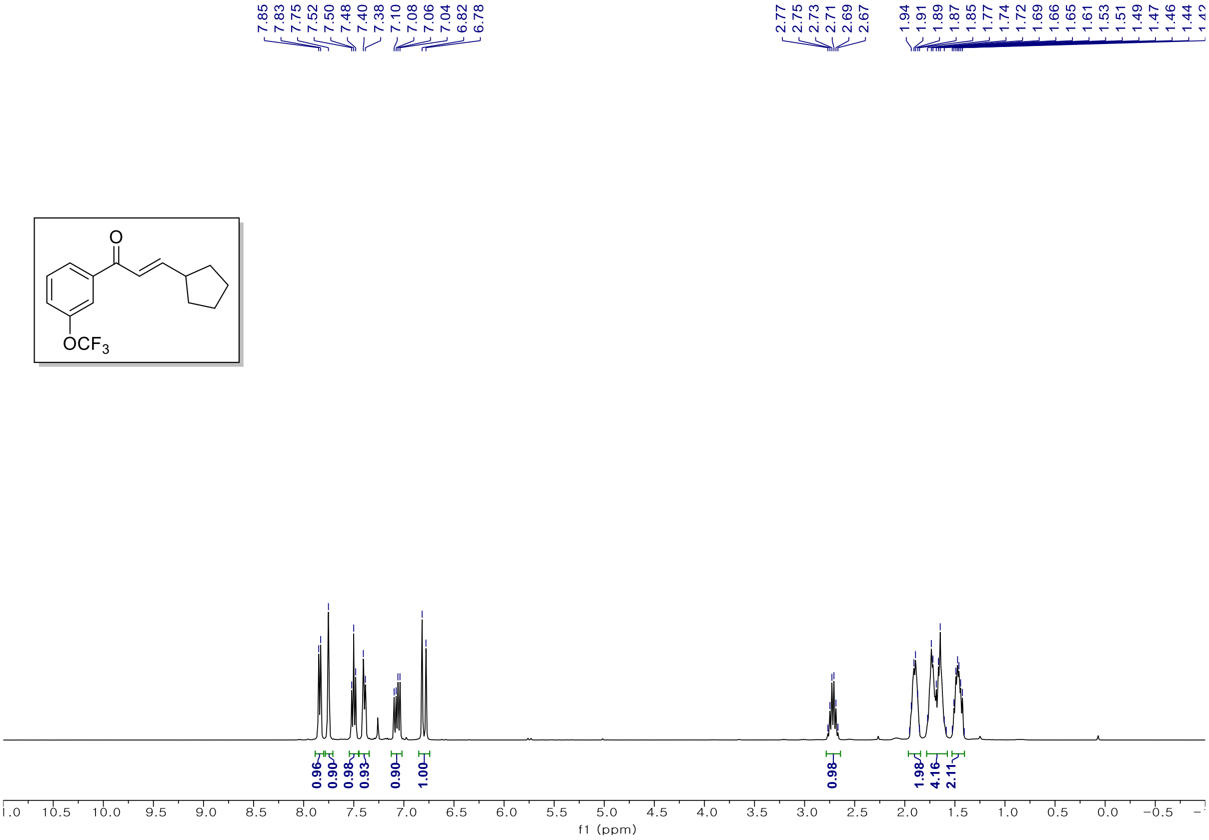

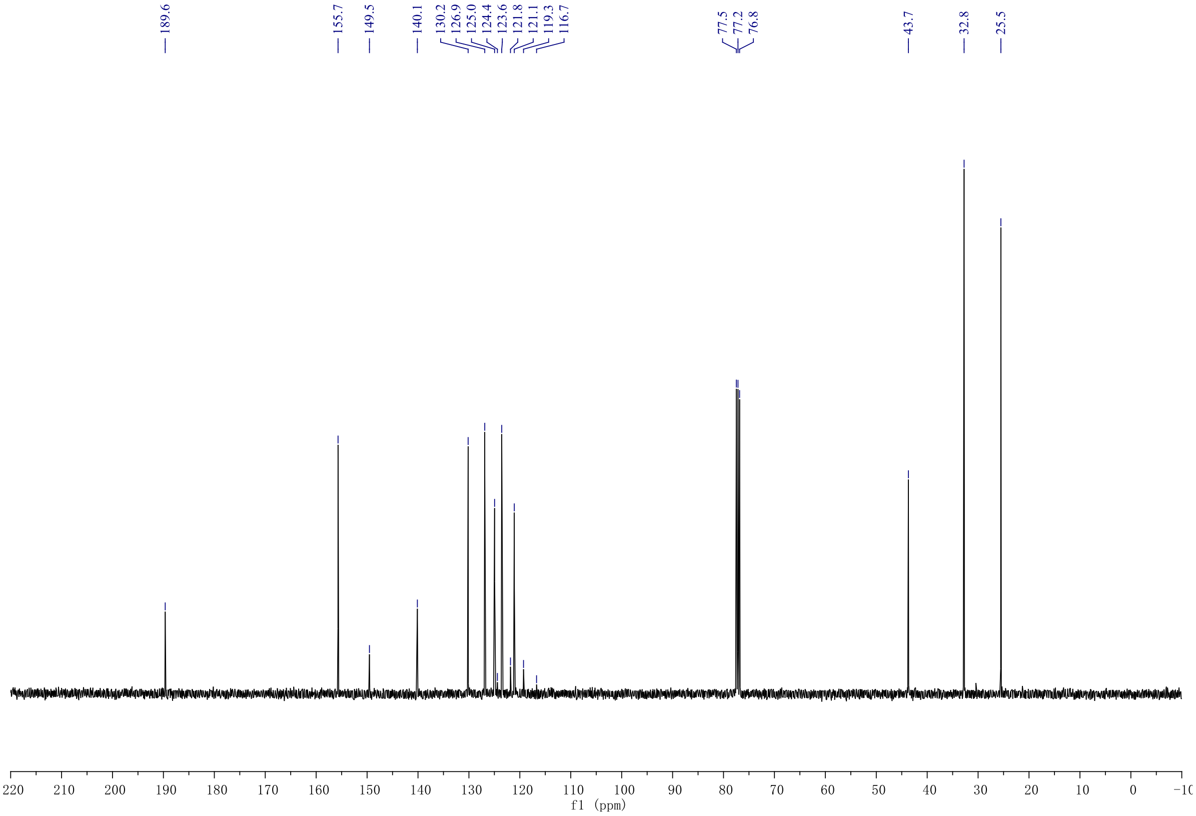

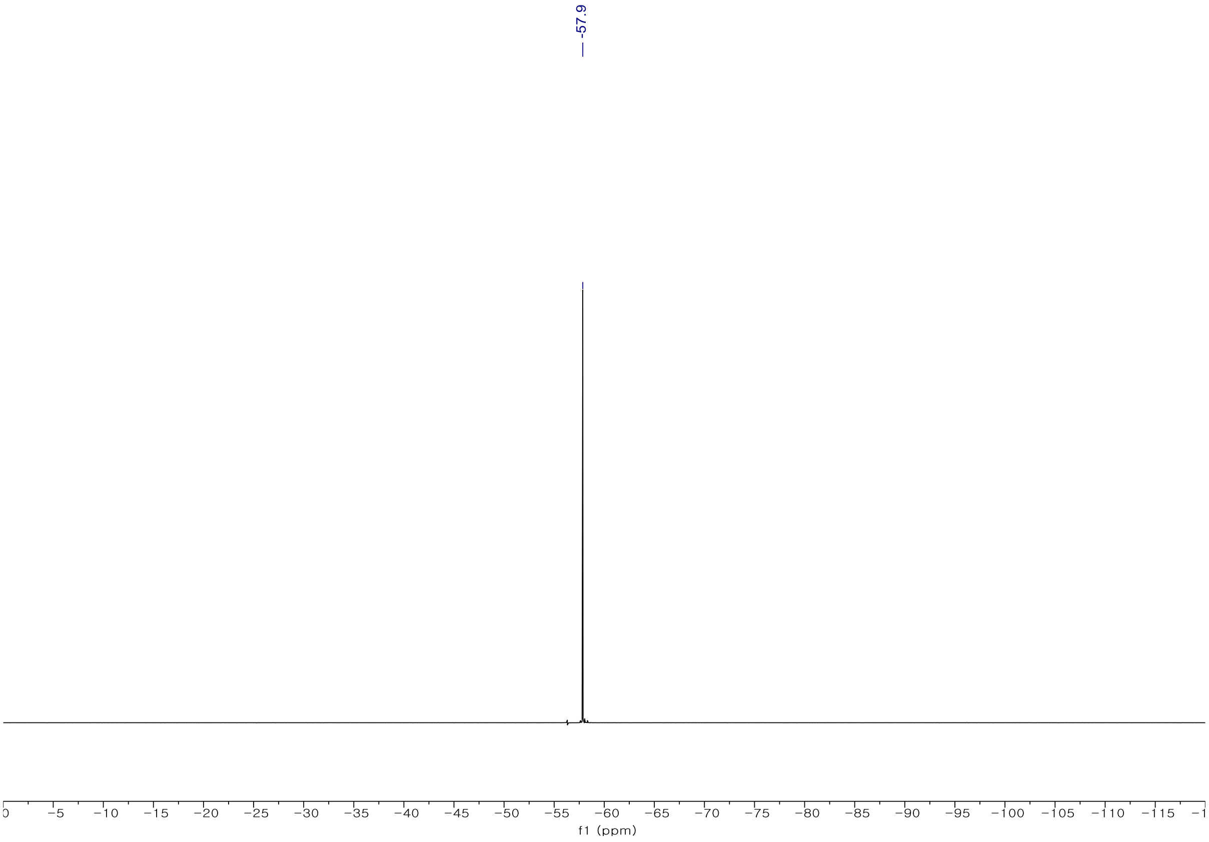

-57.9
0
−5
−10
−15
−20
−25
−30
−35
−40
−45
−50
−55
−60
−65
−70
−75
−80
−85
−90
−95
−100
−105
−110
−115
f1 (ppm)

(*E*)-3-Cyclopentyl-1-(3-fluorophenyl)prop-2-en-1-one (4l)

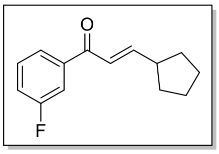

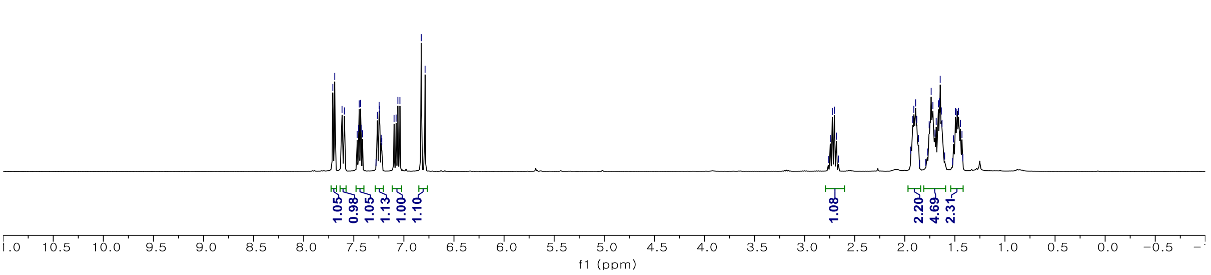

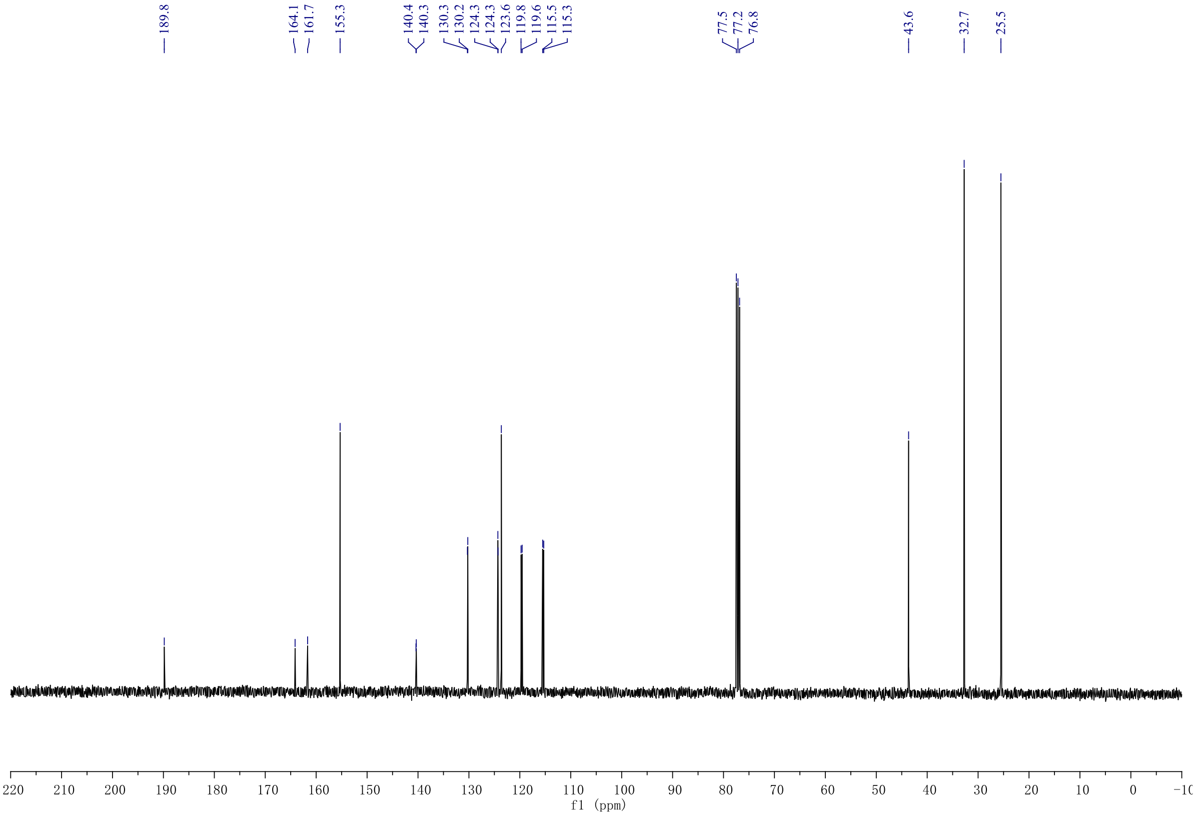

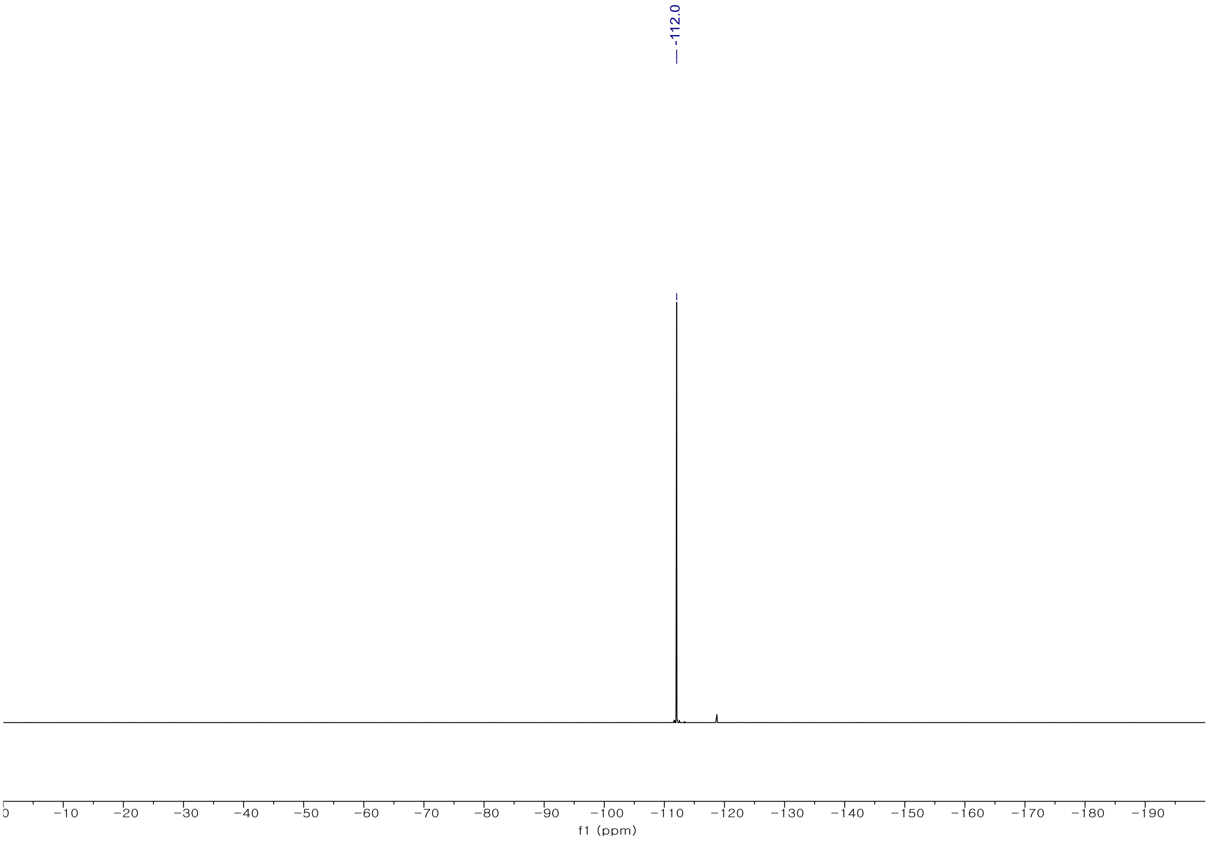

-112.0
0
-10
-20
-30
-40
-50
-60
-70
-80
-90
-100
-110
-120
-130
-140
-150
-160
-170
-180
-190
f1 (ppm)

(*E*)-Butyl 3-{3-[(*E*)-4,4-dimethylpent-2-enoyl]-2-[(*E*)-piperidin-1-yldiazenyl]phenyl}acrylate (5a)

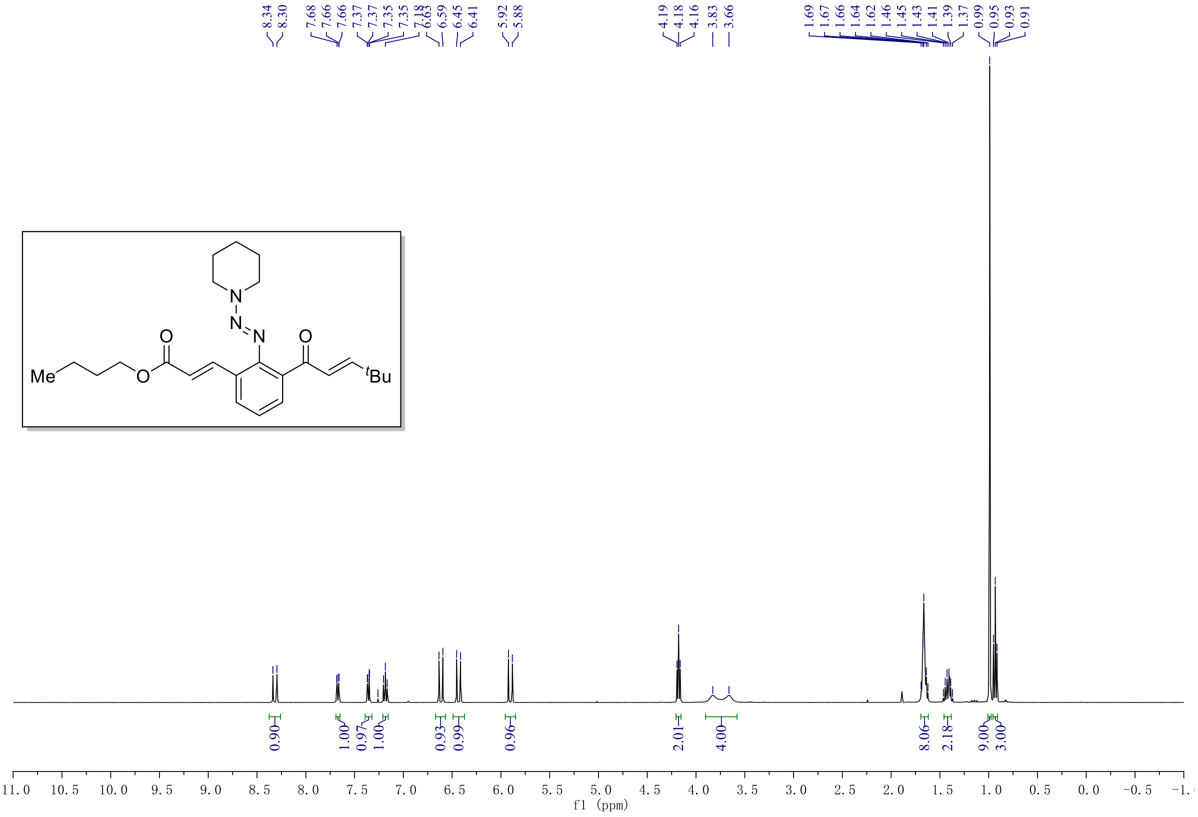

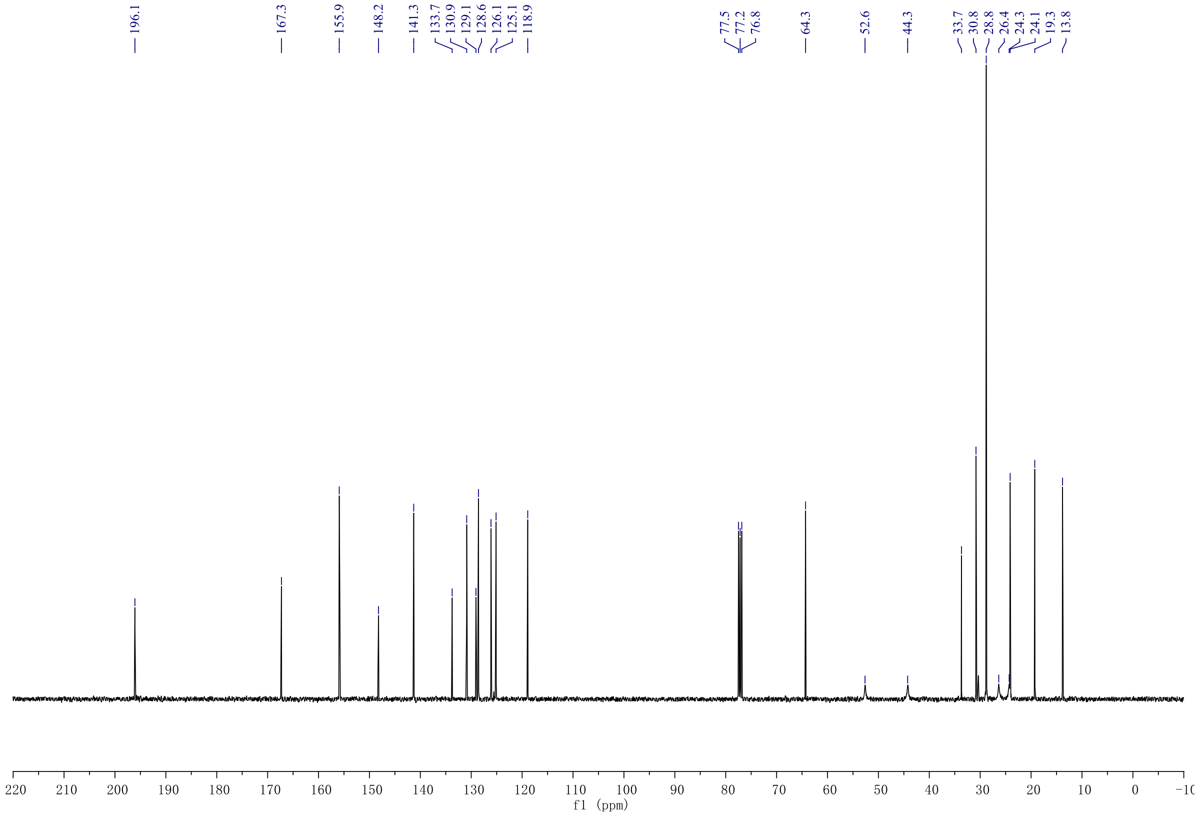

(*E*)-Butyl 3-{3-[(*E*)-non-2-enoyl]-2-[(*E*)-piperidin-1-yldiazenyl]phenyl}acrylate (5b)

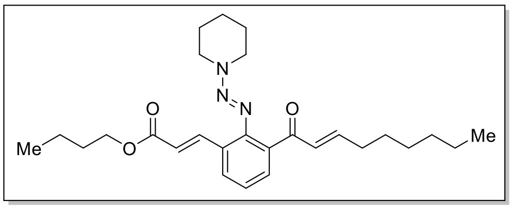

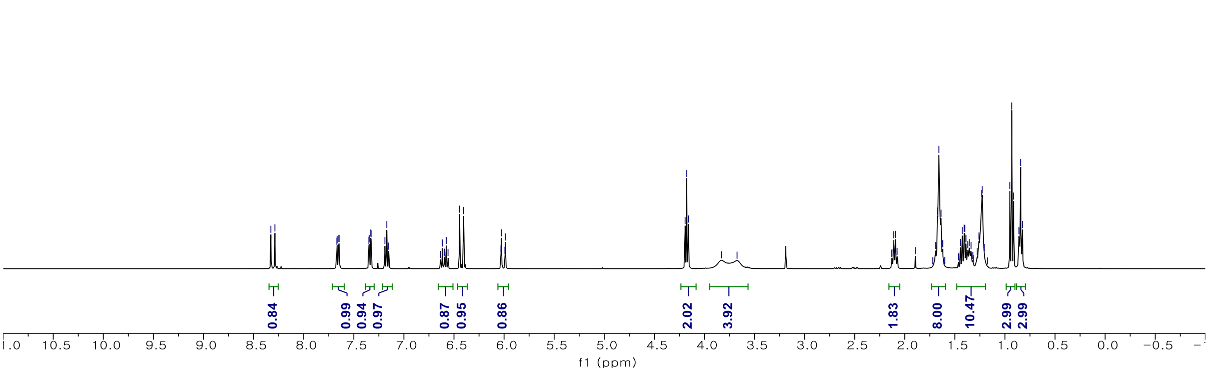

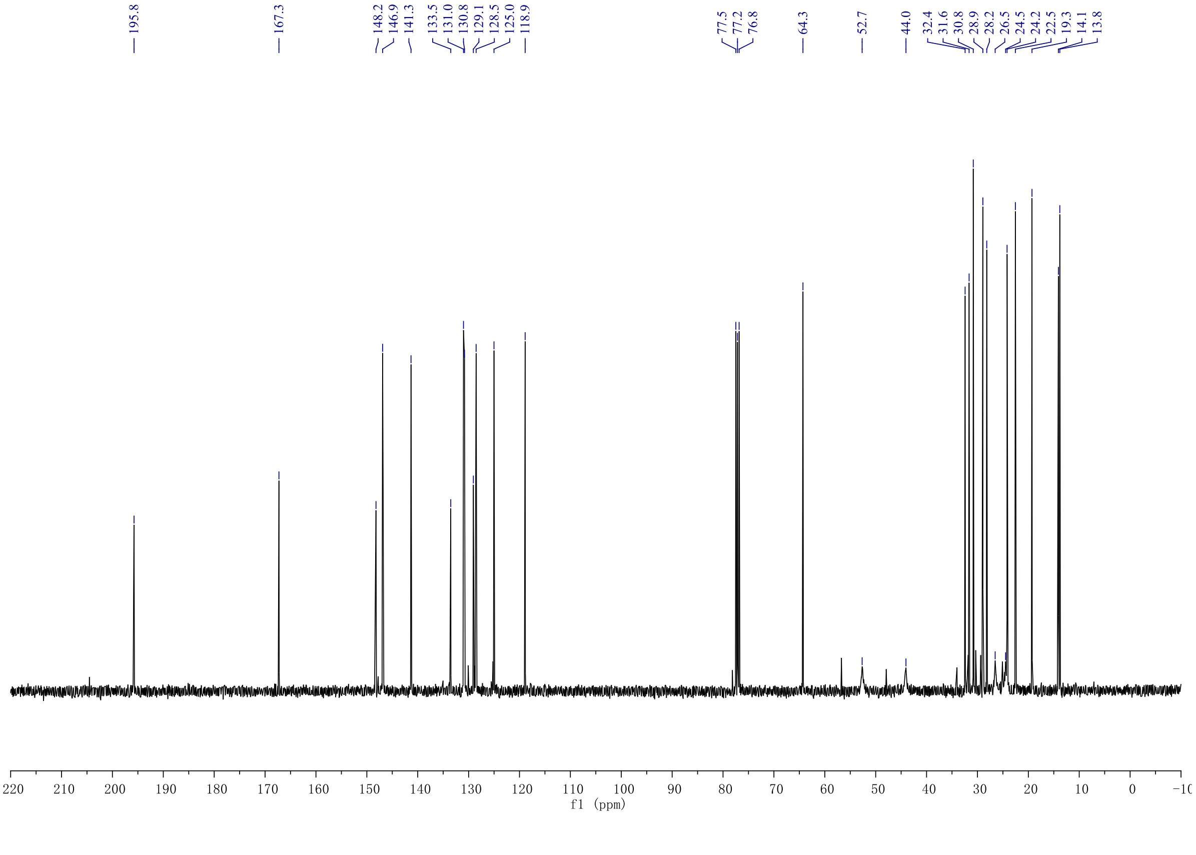

(*E*)-Butyl 3-{3-[(*E*)-6-chlorohex-2-enoyl]-2-[(*E*)-piperidin-1-yldiazenyl]phenyl}acrylate (5c)

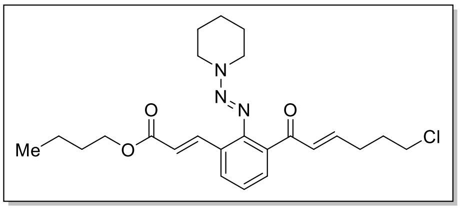

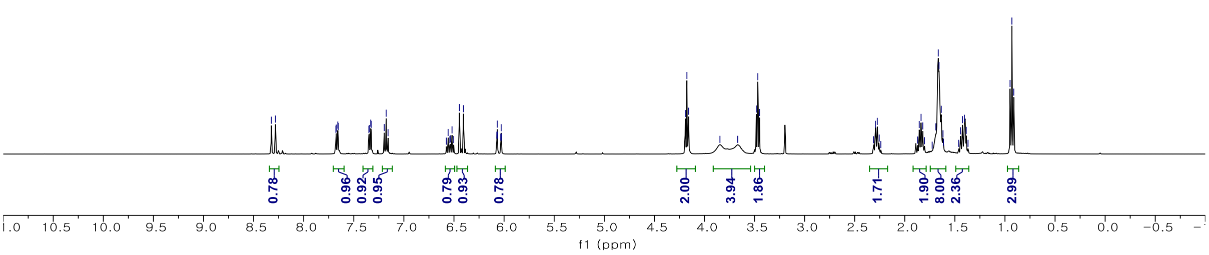

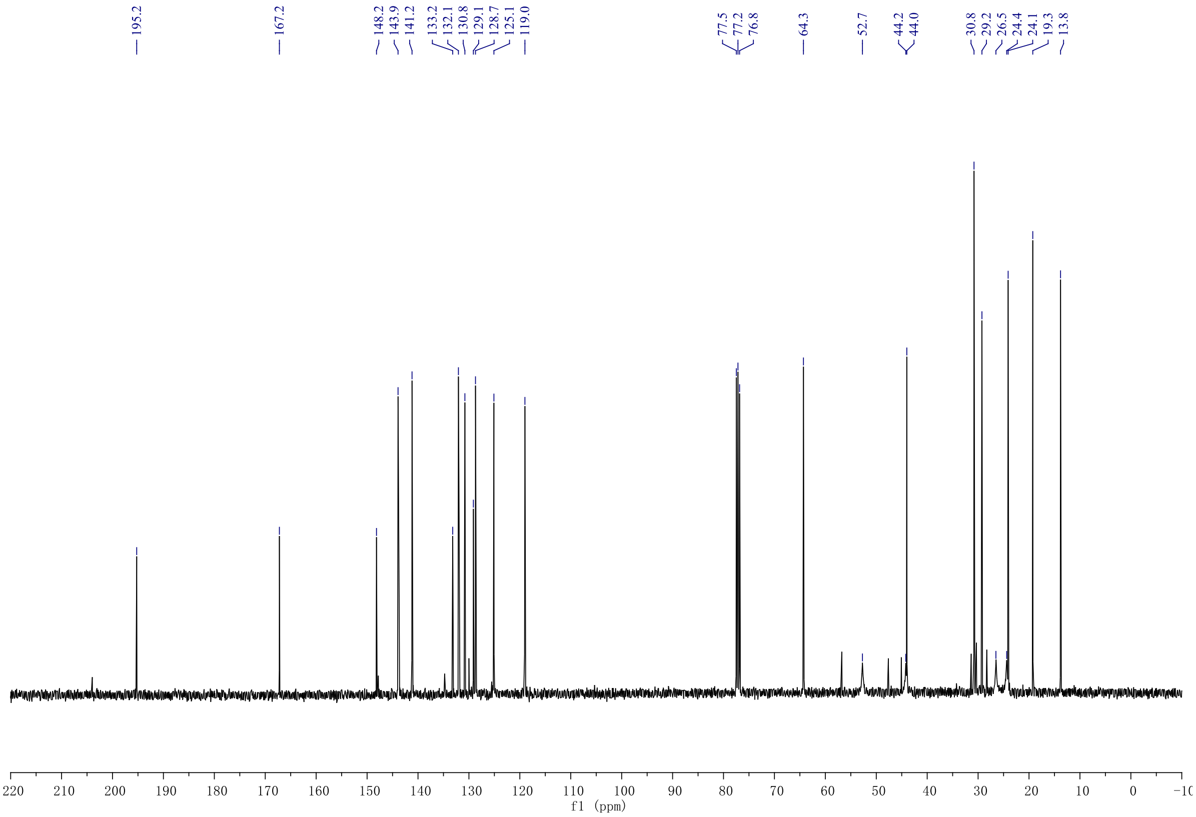

(*E*)-Butyl 3-{3-cinnamoyl-2-[(*E*)-piperidin-1-yldiazenyl]phenyl}acrylate (5d)

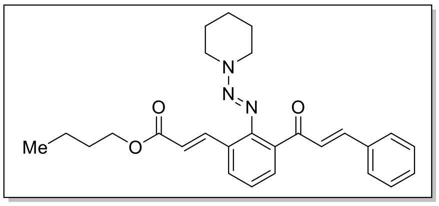

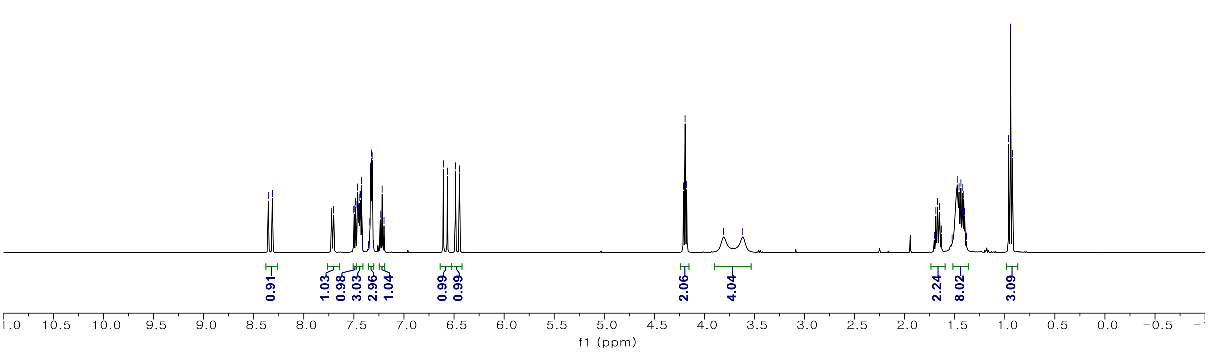

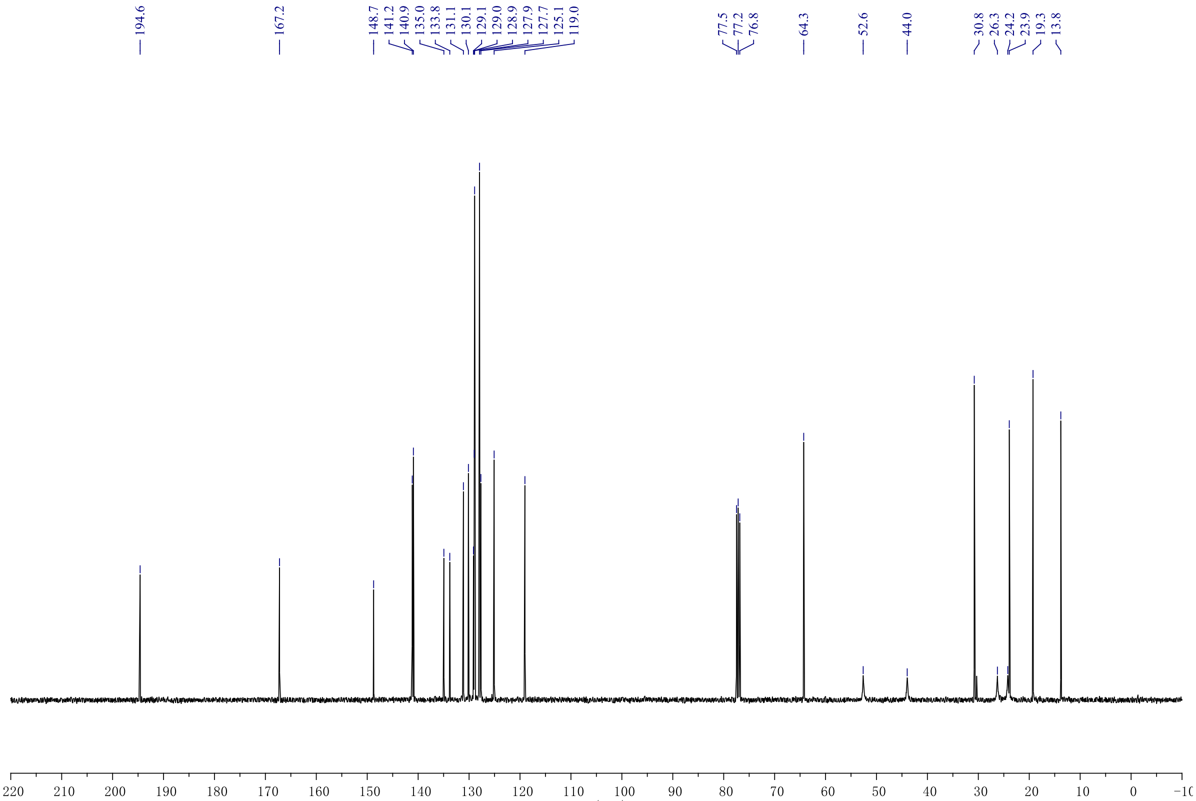

(*E*)-Butyl 3-{2-[(*E*)-piperidin-1-yldiazenyl]-3-[(*E*)-3-(thiophen-3-yl)acryloyl]phenyl}acrylate (5e)

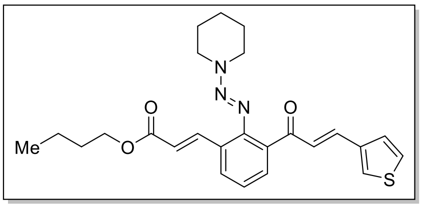

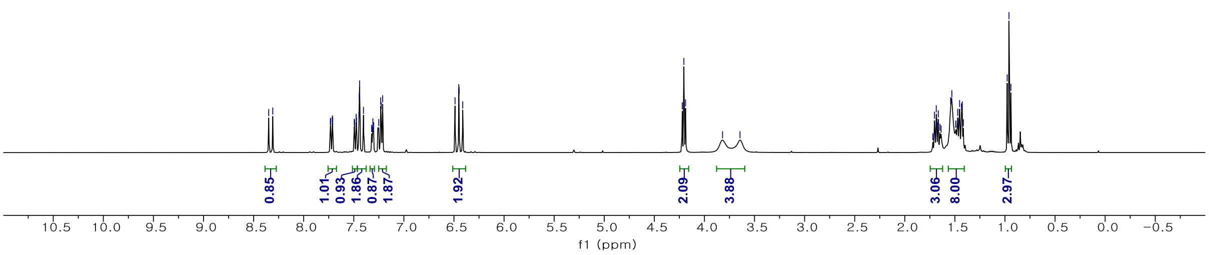

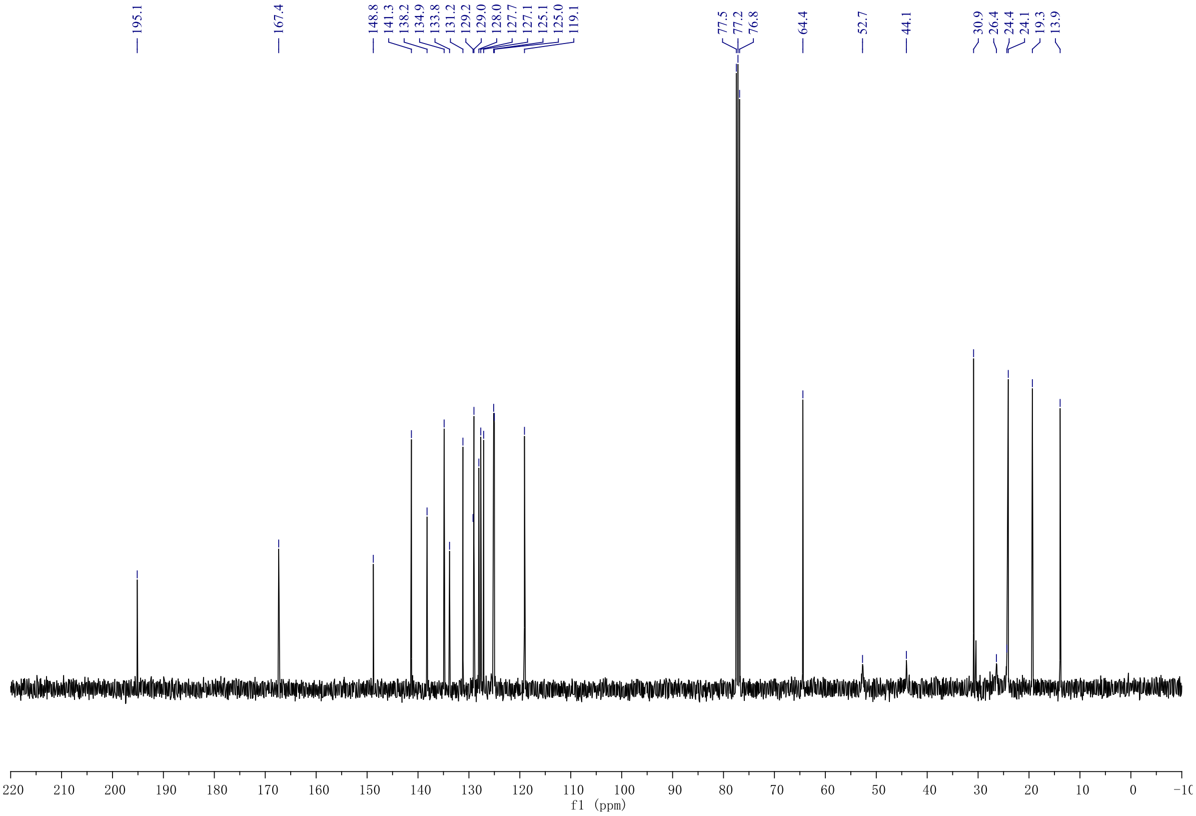

(*E*)-Butyl 3-{3-[(*E*)-4,4-dimethylpent-2-enoyl]-4-fluoro-2-[(*E*)-piperidin-1-yl diazenyl]phenyl}-acrylate (5f)

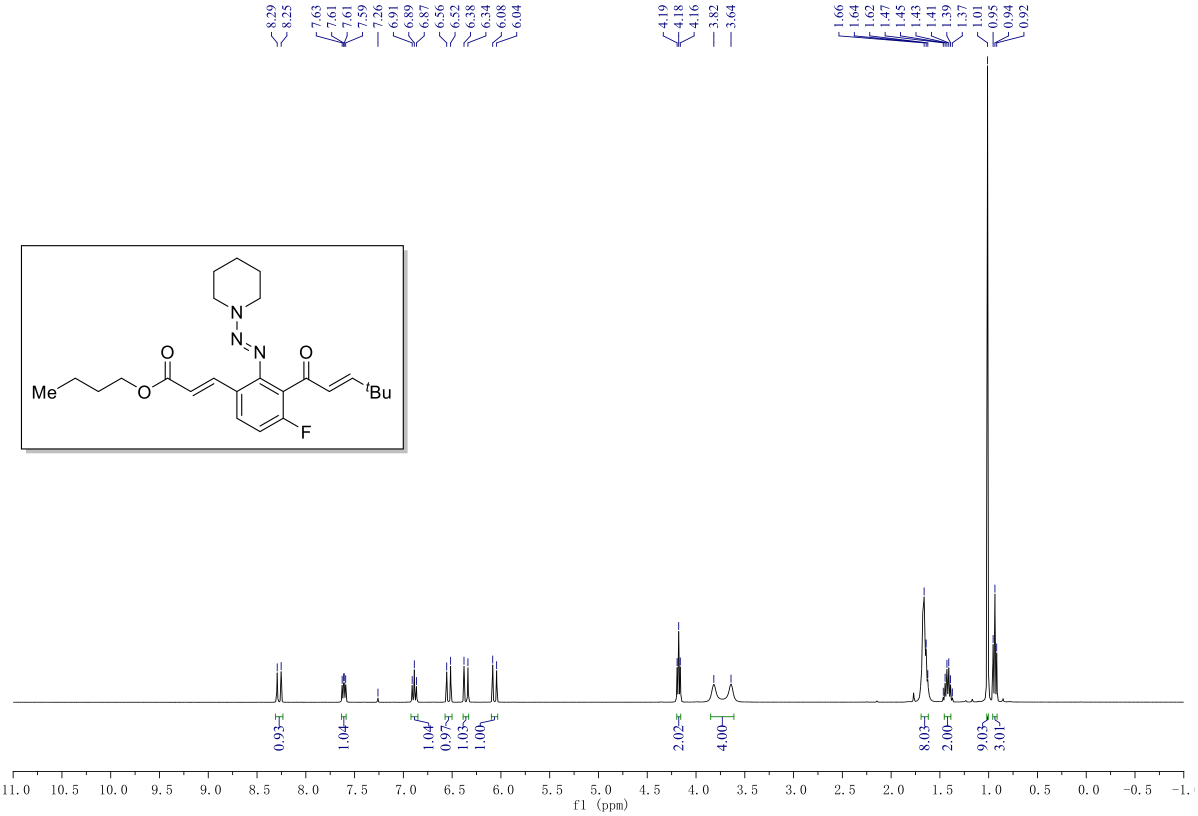

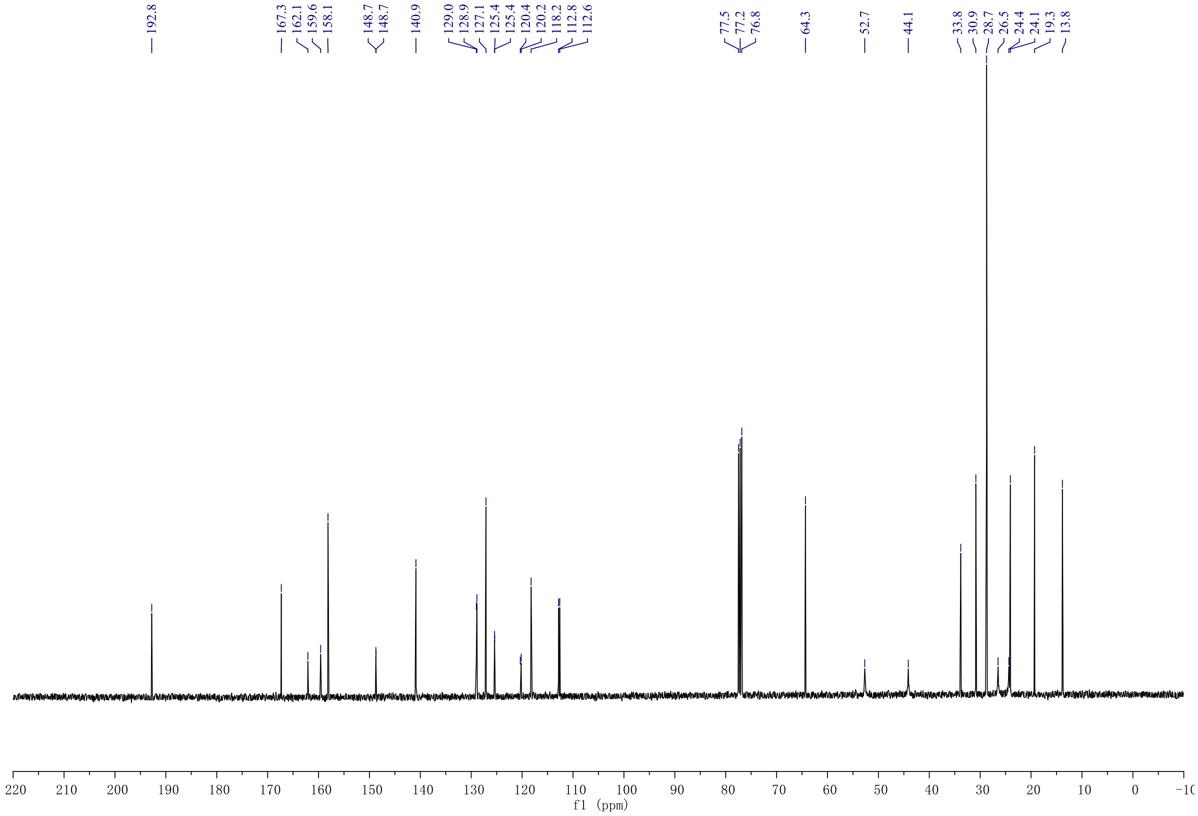

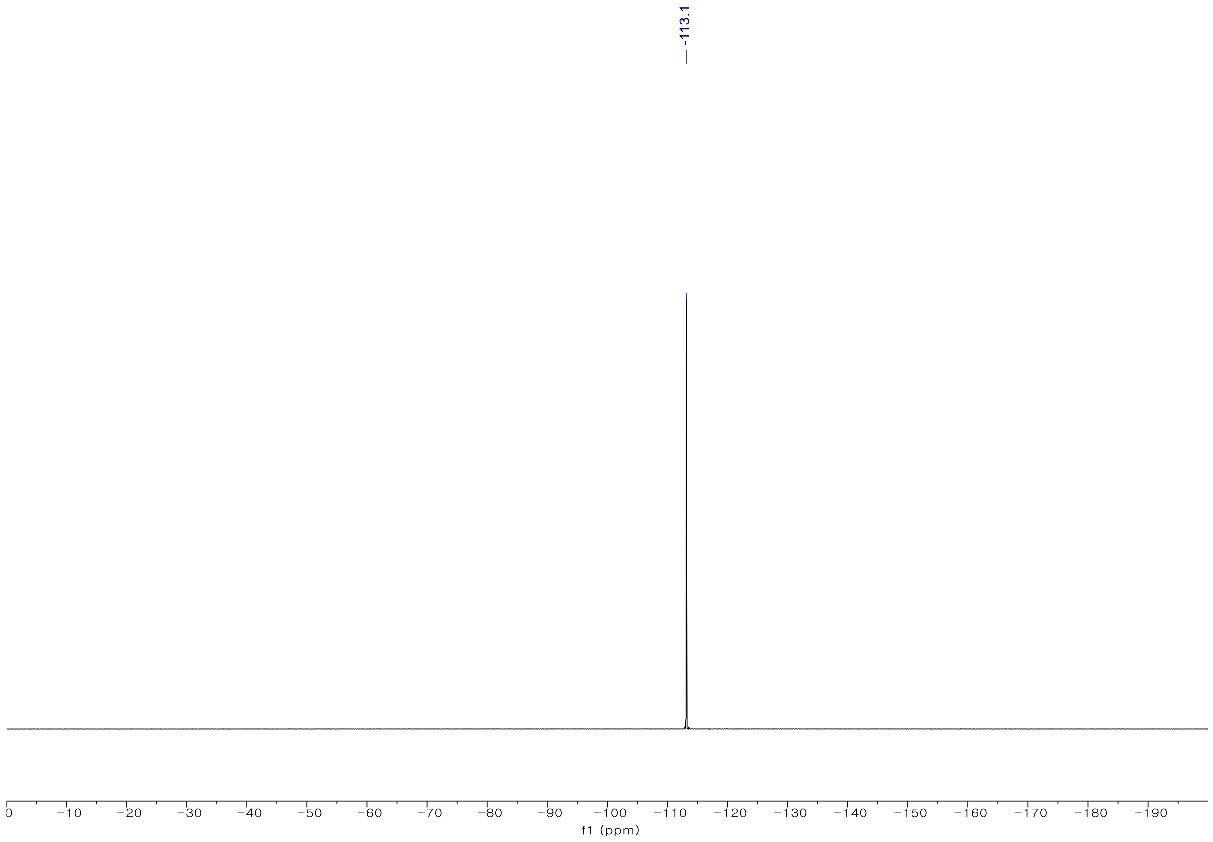
-113.1
0
−10
−20
−30
−40
−50
−60
−70
−80
−90
−100
−110
−120
−130
−140
−150
−160
−170
−180
−190
f1 (ppm)

(*E*)-Butyl 3-{3-[(*E*)-4,4-dimethylpent-2-enoyl]-5-methyl-2-[(*E*)-piperidin-1-yl diazenyl]phenyl}-acrylate (5g)

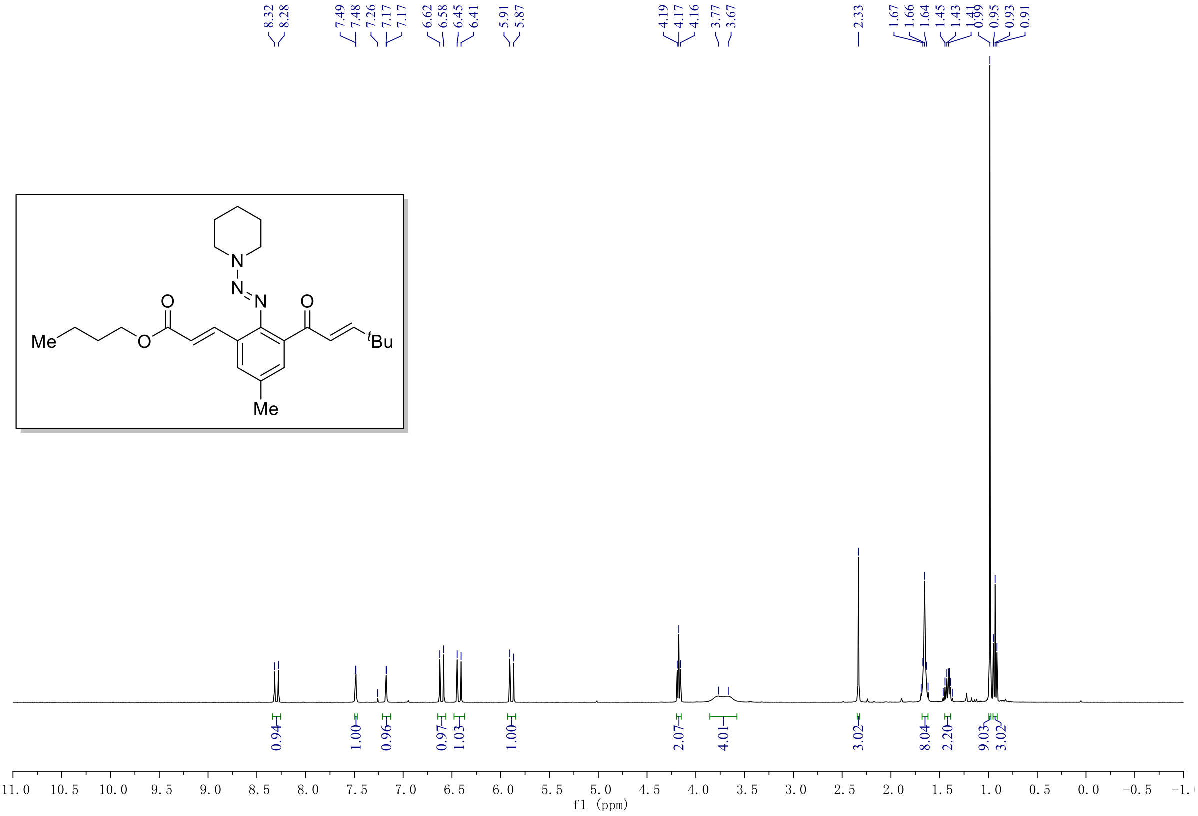

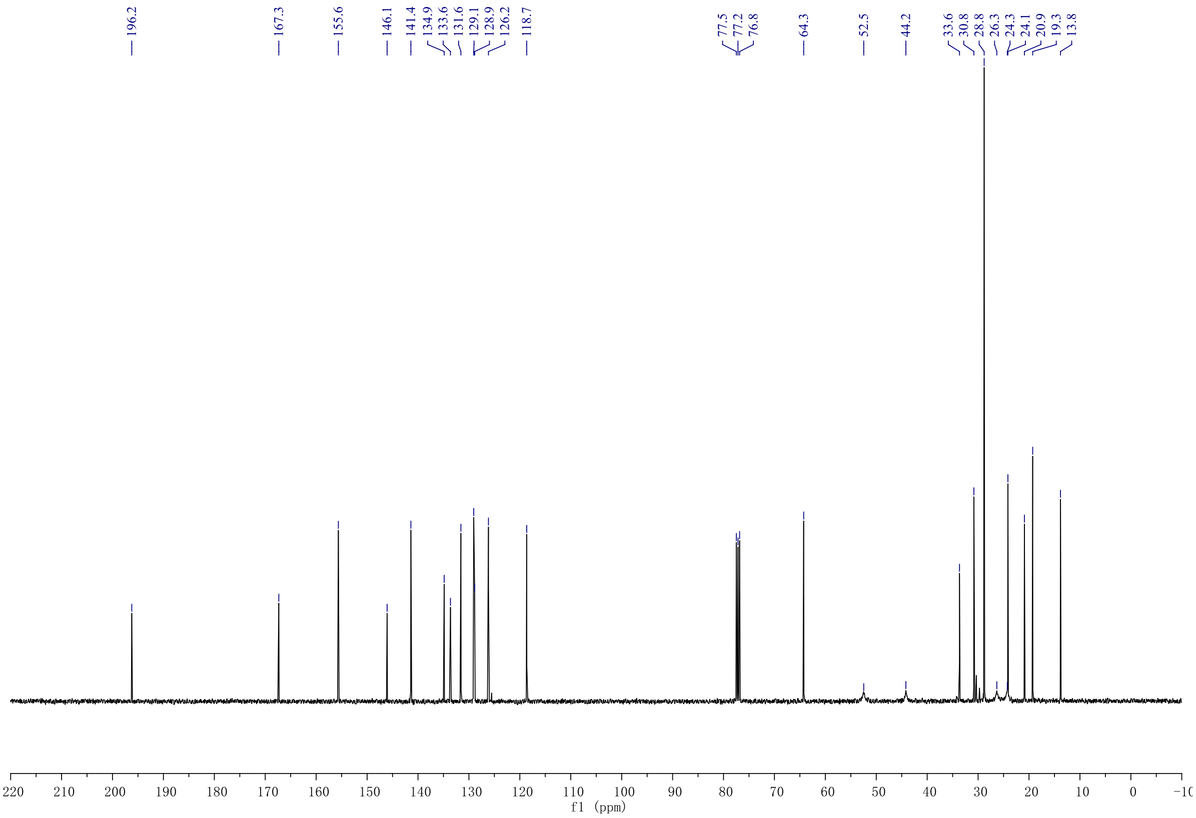

(*E*)-Butyl 3-{3-[(*E*)-4,4-dimethylpent-2-enoyl]-5-fluoro-2-[(*E*)-piperidin-1-yl diazenyl]phenyl}-acrylate (5h)

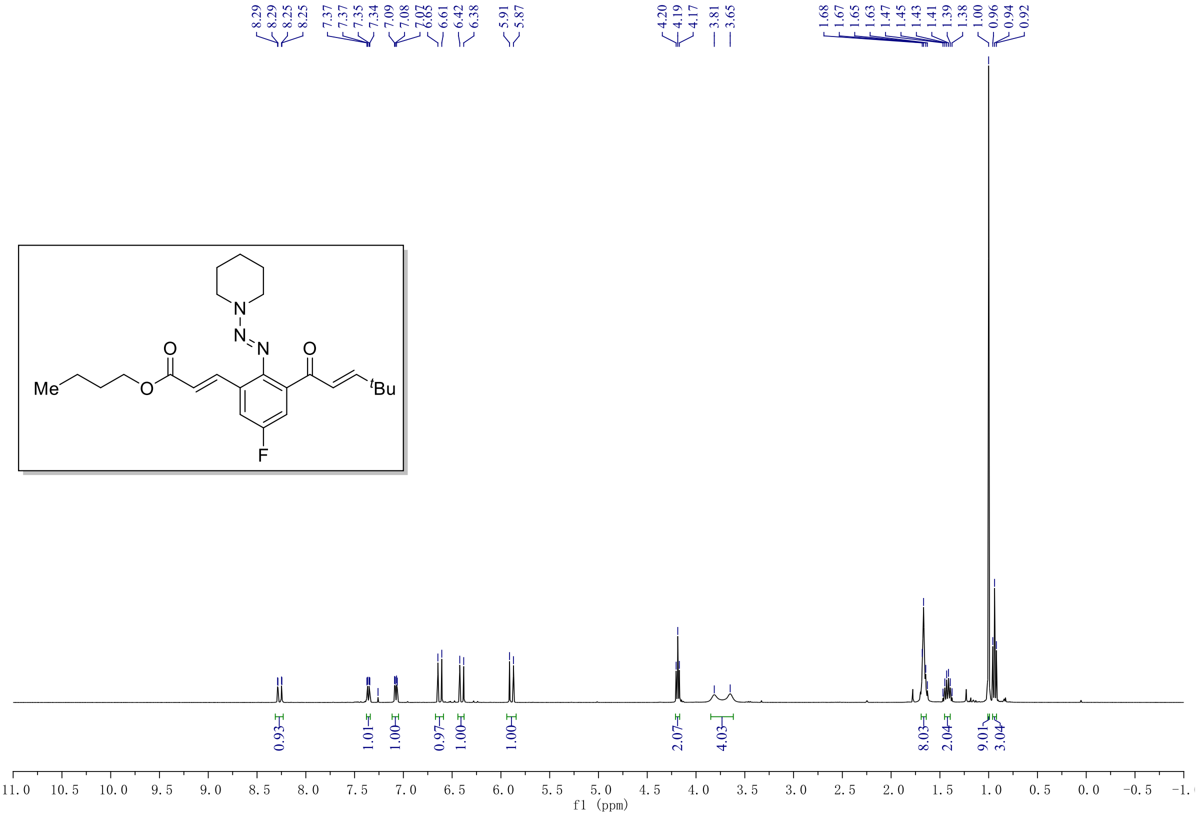

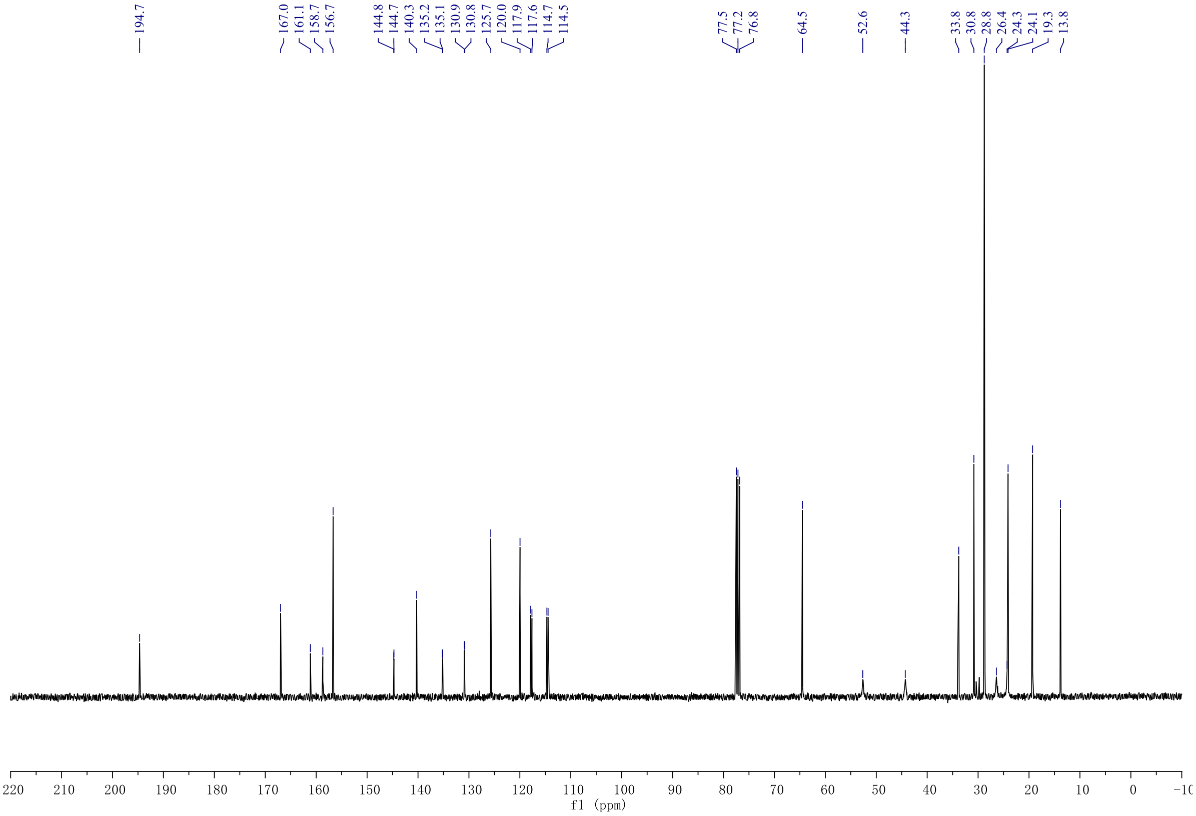

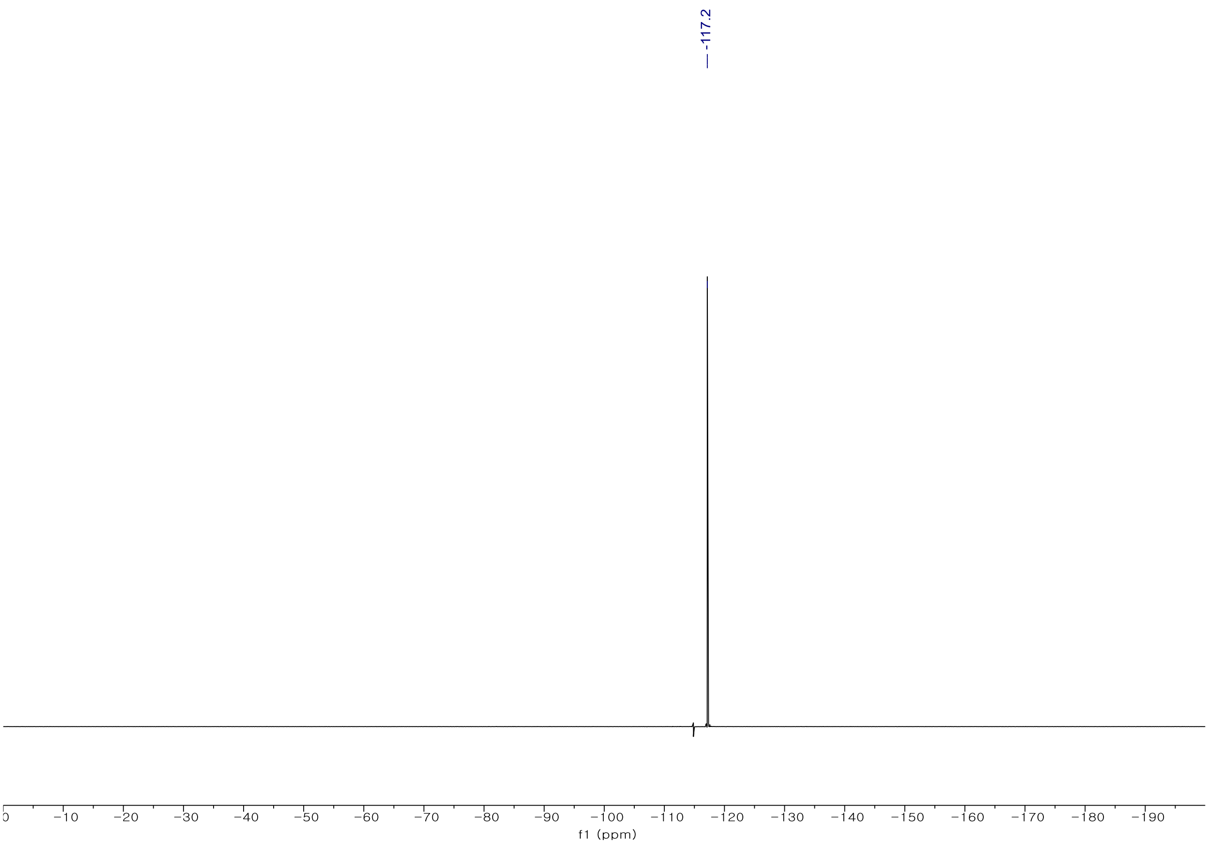
-117.2
0 −10 −20 −30 −40 −50 −60 −70 −80 −90 −100 −110 −120 −130 −140 −150 −160 −170 −180 −190
f1 (ppm)

(*E*)-Butyl 3-{6-chloro-3-[(*E*)-4,4-dimethylpent-2-enoyl]-2-[(*E*)-piperidin-1-yl diazenyl]phenyl}-acrylate (5i)

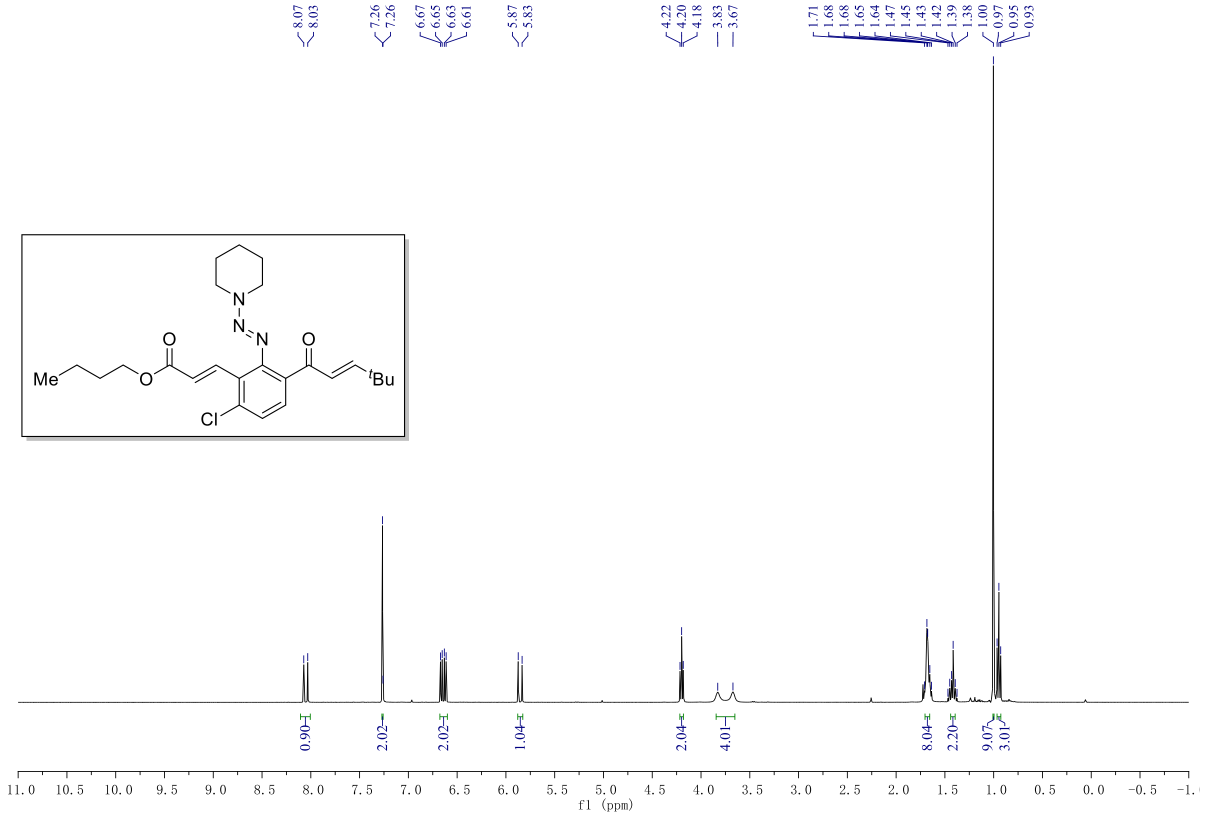

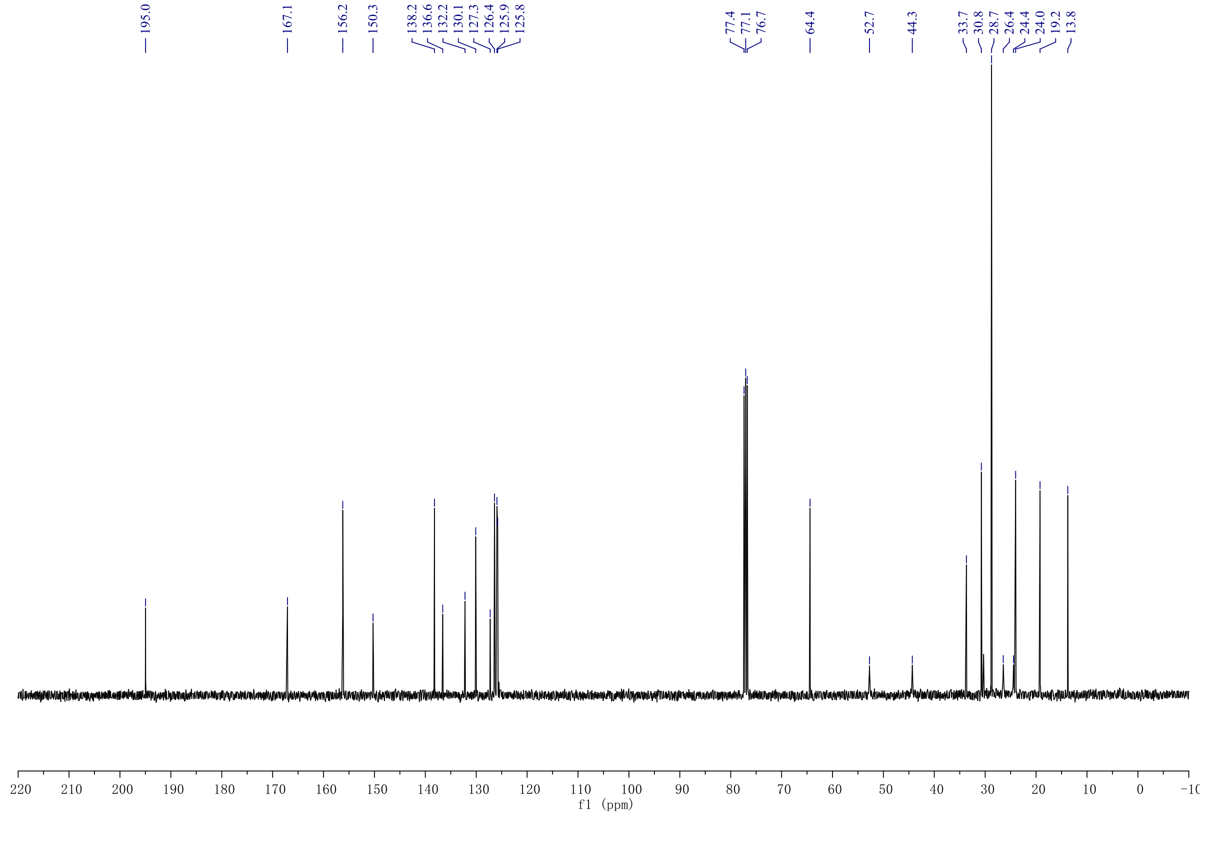

(*E*)-Butyl 3-{3-[(*E*)-4,4-dimethylpent-2-enoyl]-2-[(*E*)-piperidin-1-yldiazenyl]naphthalen-1-yl}-acrylate (5j)

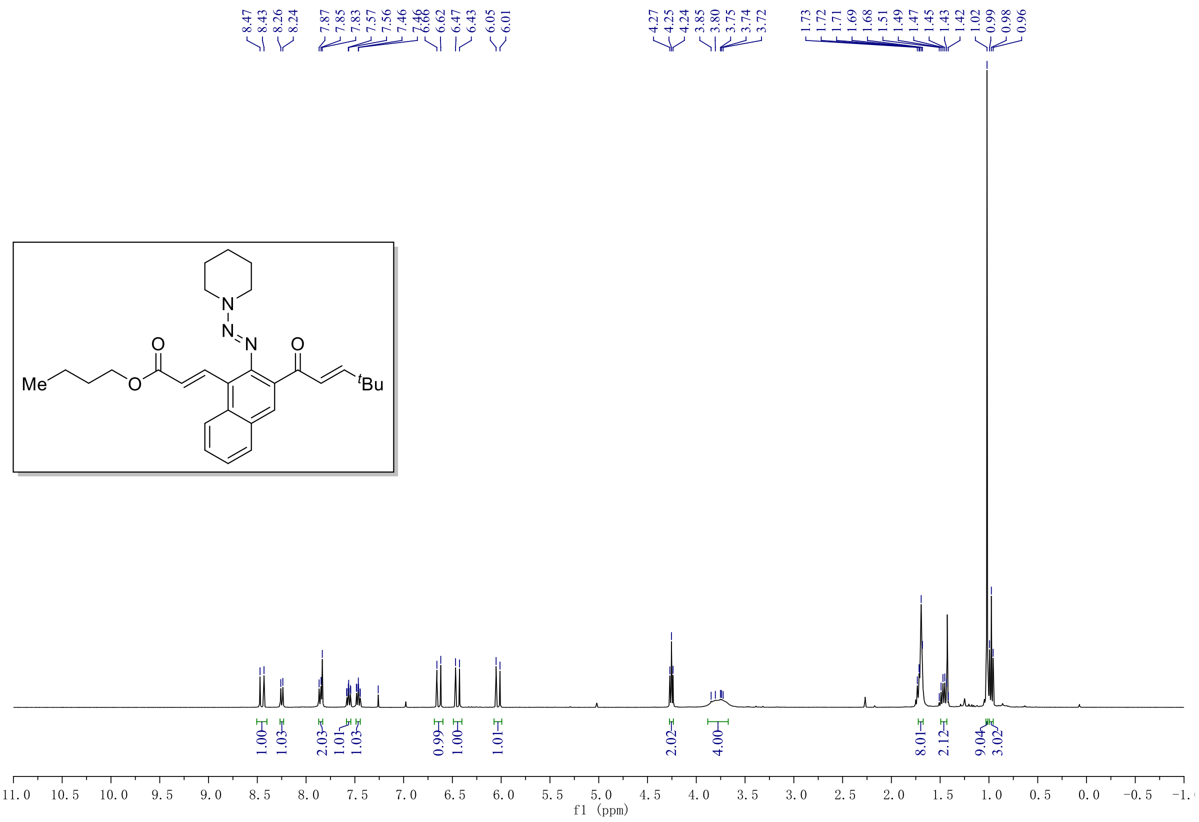

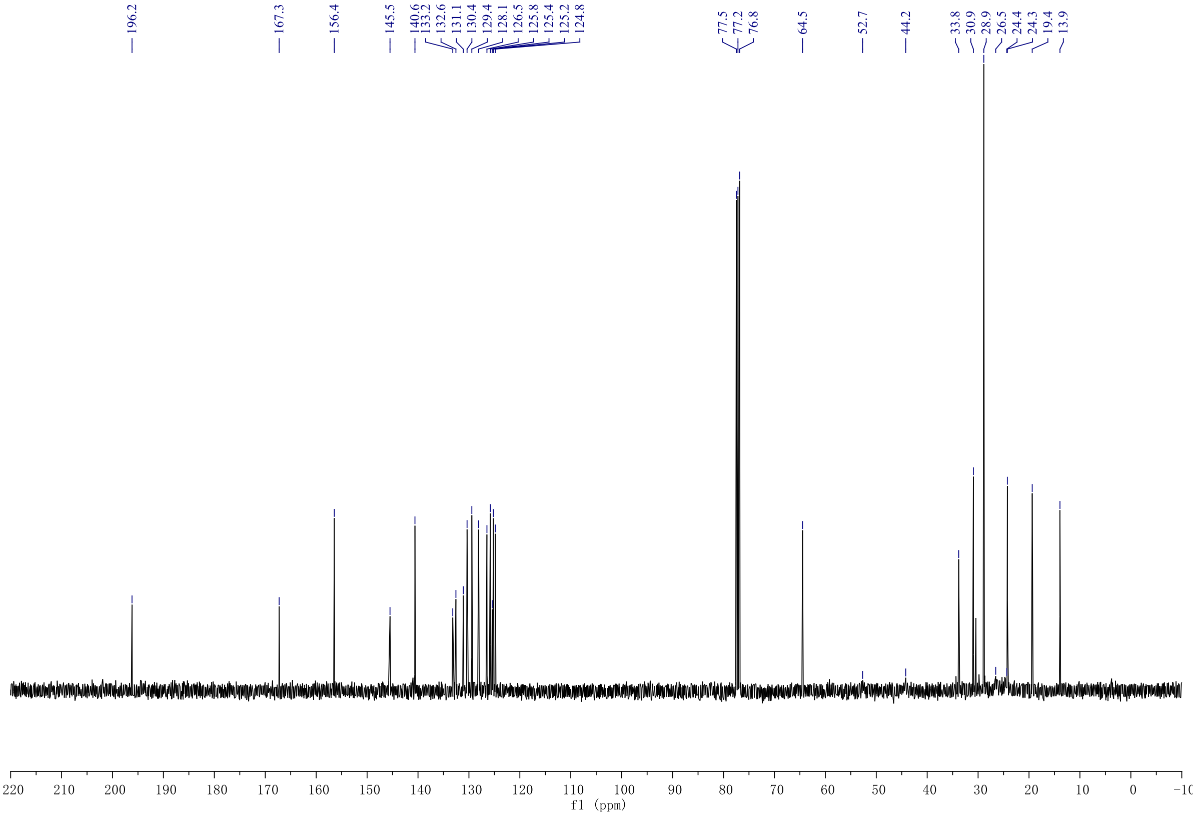

(*E*)-*tert*-Butyl 3-{3-[(*E*)-4,4-dimethylpent-2-enoyl]-2-[(*E*)-piperidin-1-yldiazenyl] phenyl}acrylate (5k)

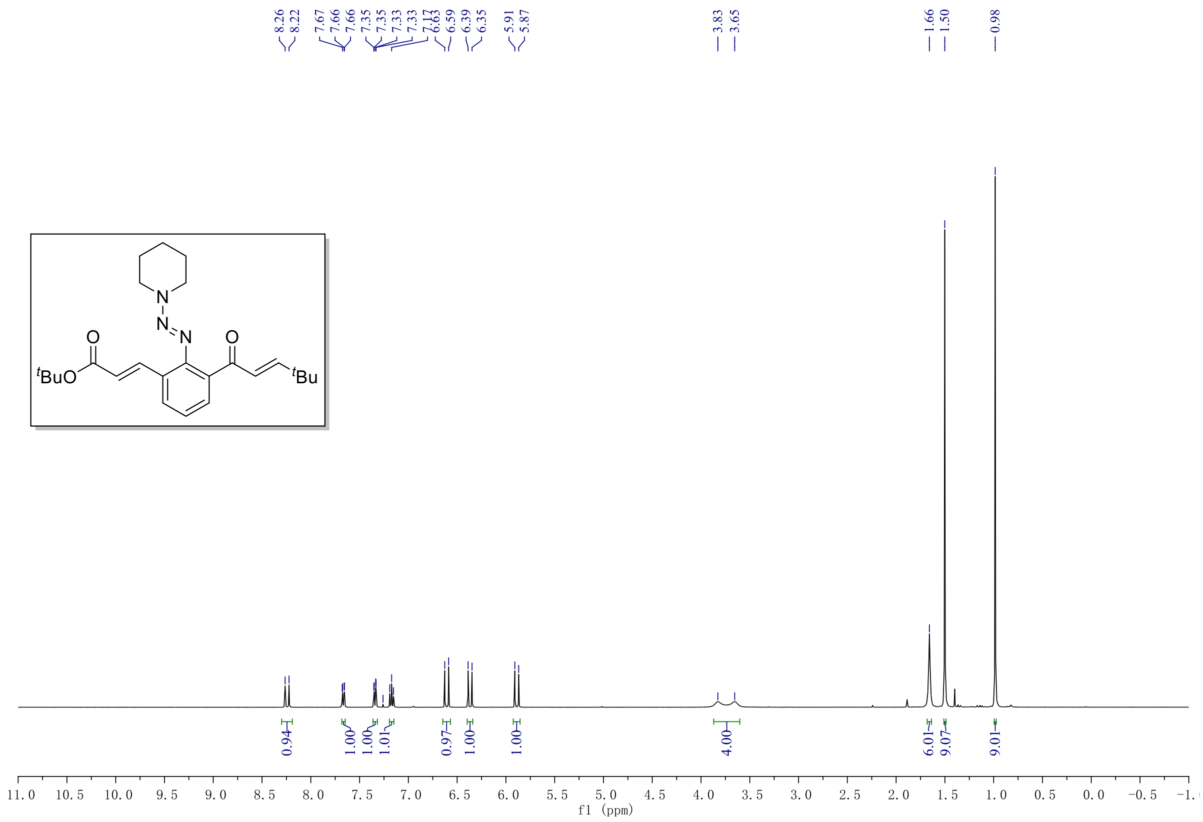

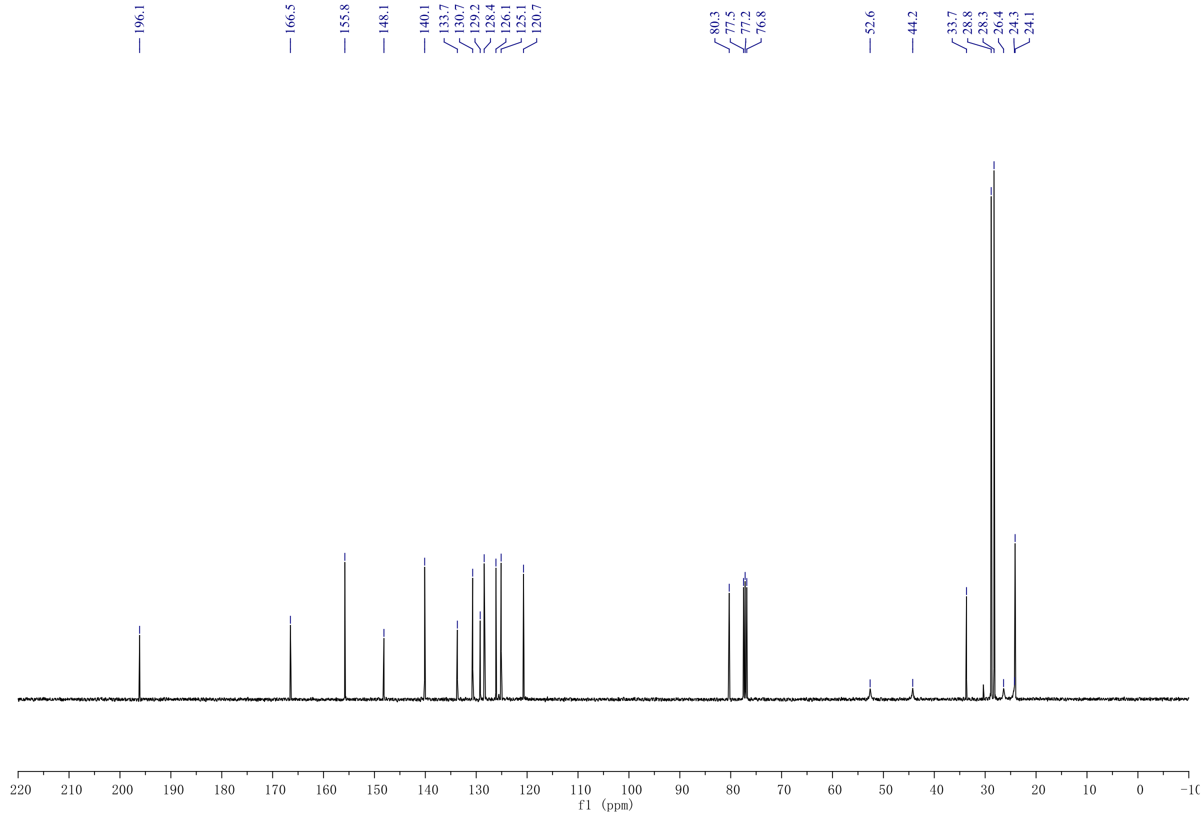

(*E*)-3-{3-[(*E*)-4,4-Dimethylpent-2-enoyl]-2-[(*E*)-piperidin-1-yldiazenyl]phenyl}-*N*,*N*-dimethyl-acrylamide (5l)

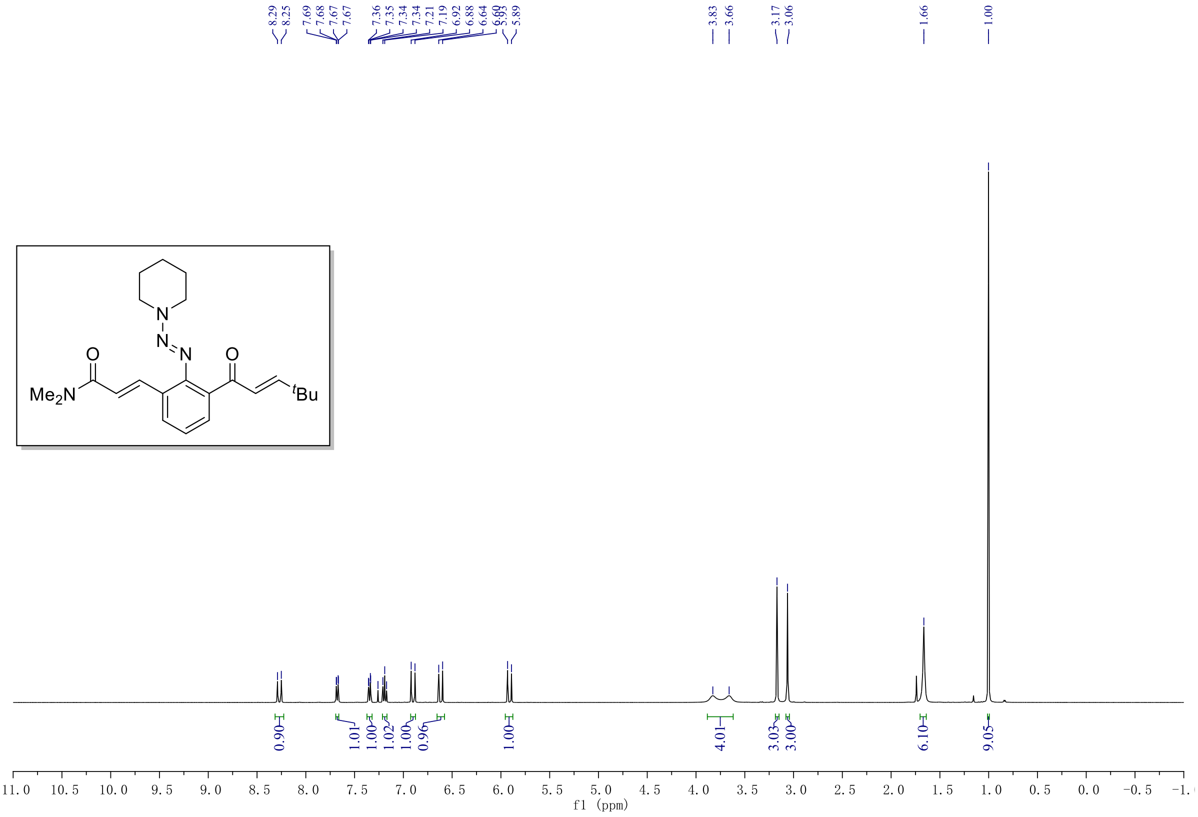

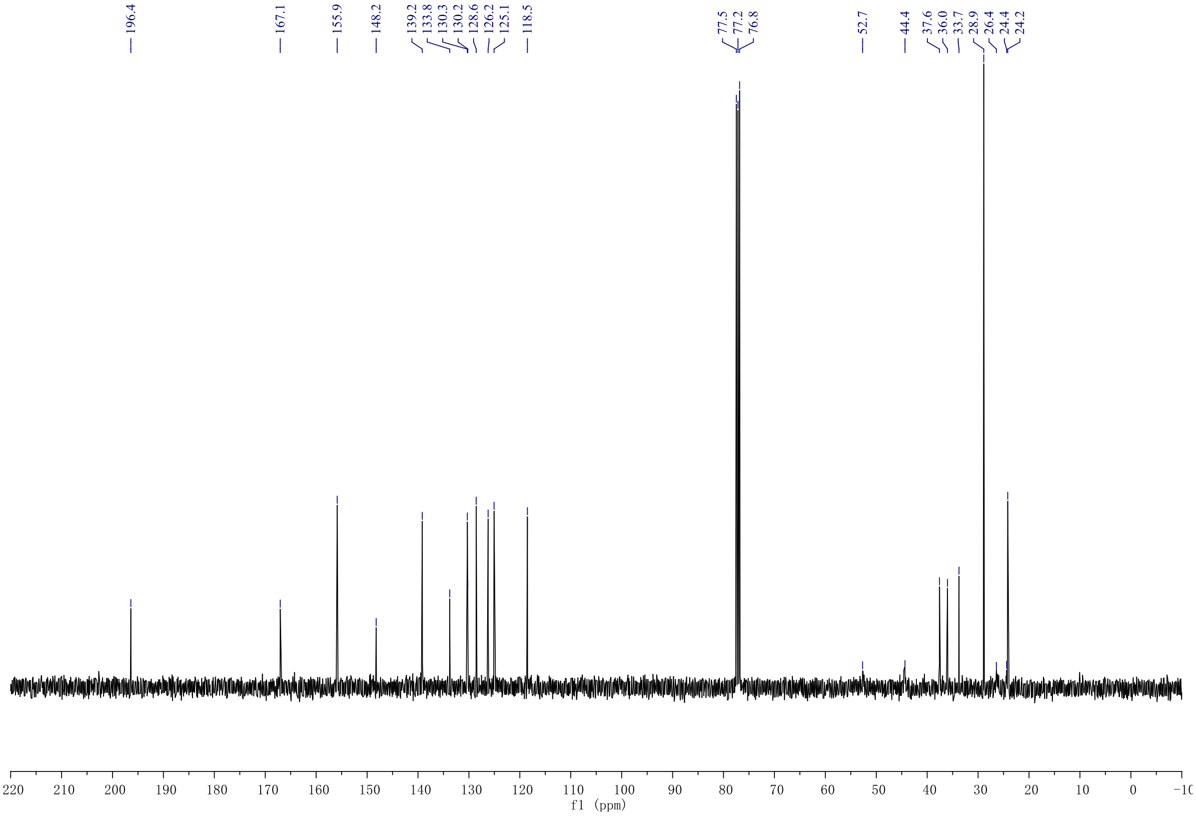

(*E*)-4,4-Dimethyl-1-{3-[(*E*)-2-(phenylsulfonyl)vinyl]-2-[(*E*)-piperidin-1-yldiazenyl]phenyl}pent-2-en-1-one (5m)

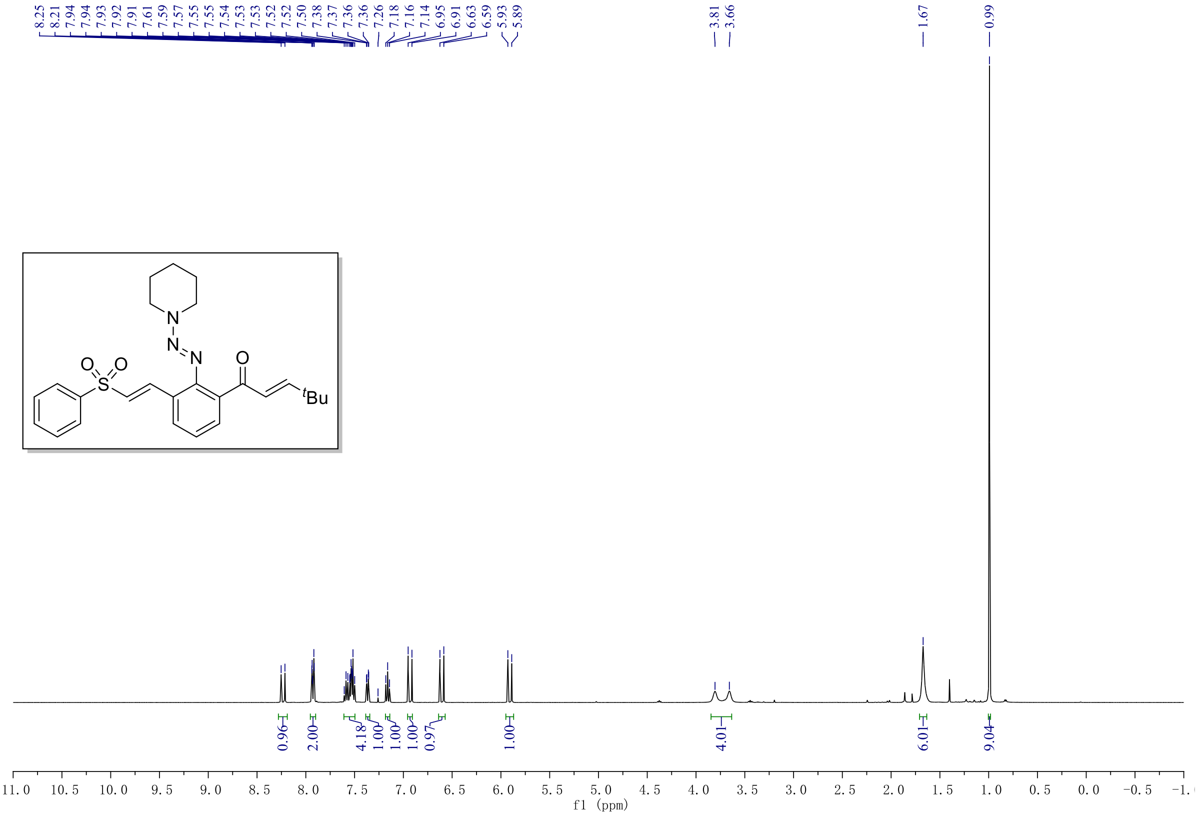

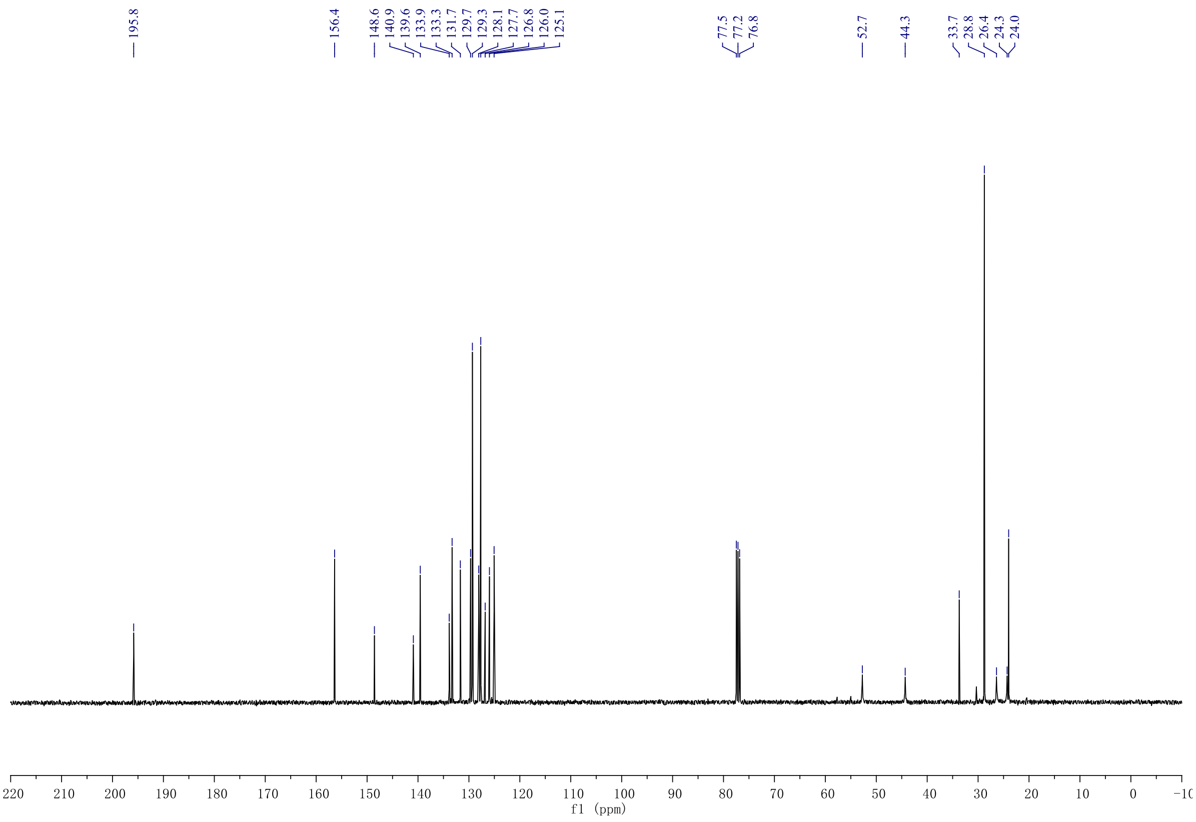

(*E*)-4,4-Dimethyl-1-{2-[(*E*)-piperidin-1-yldiazenyl]-3-[(*E*)-4-(trifluoromethyl)styryl]phenyl}pent-2-en-1-one (5n)

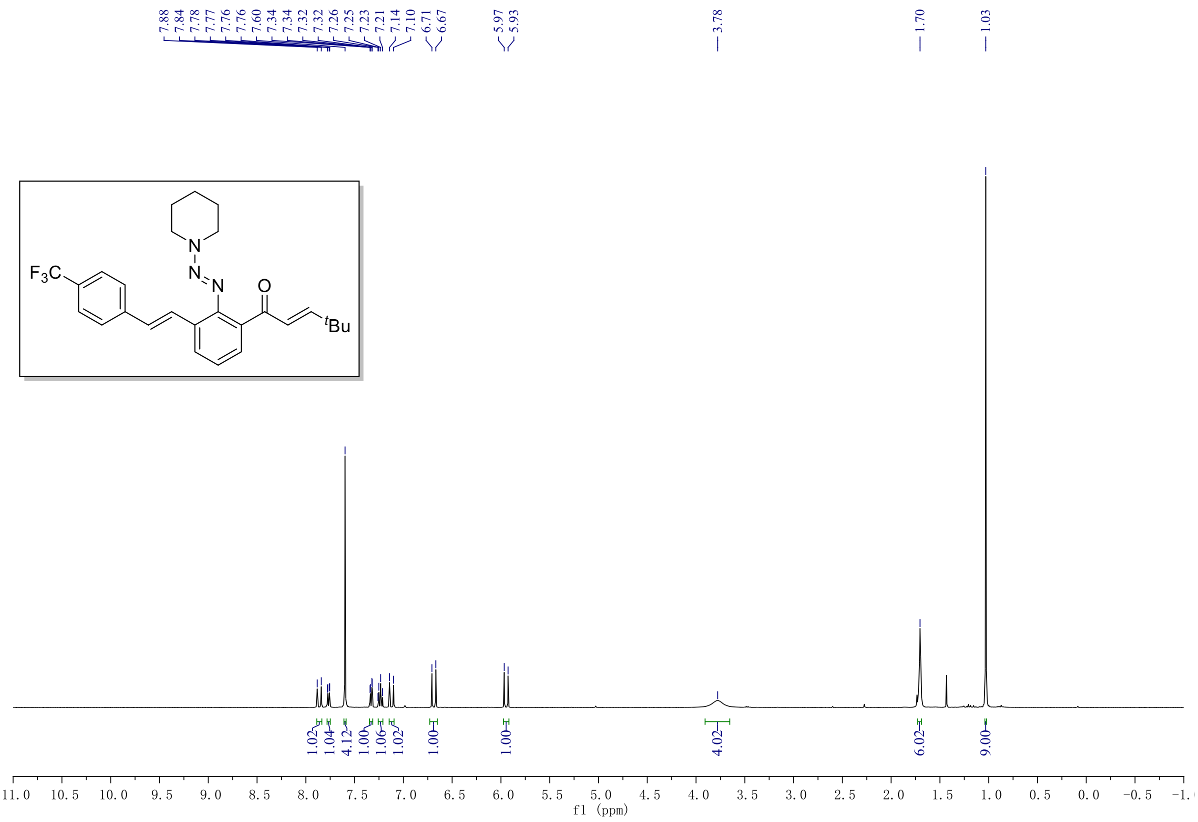

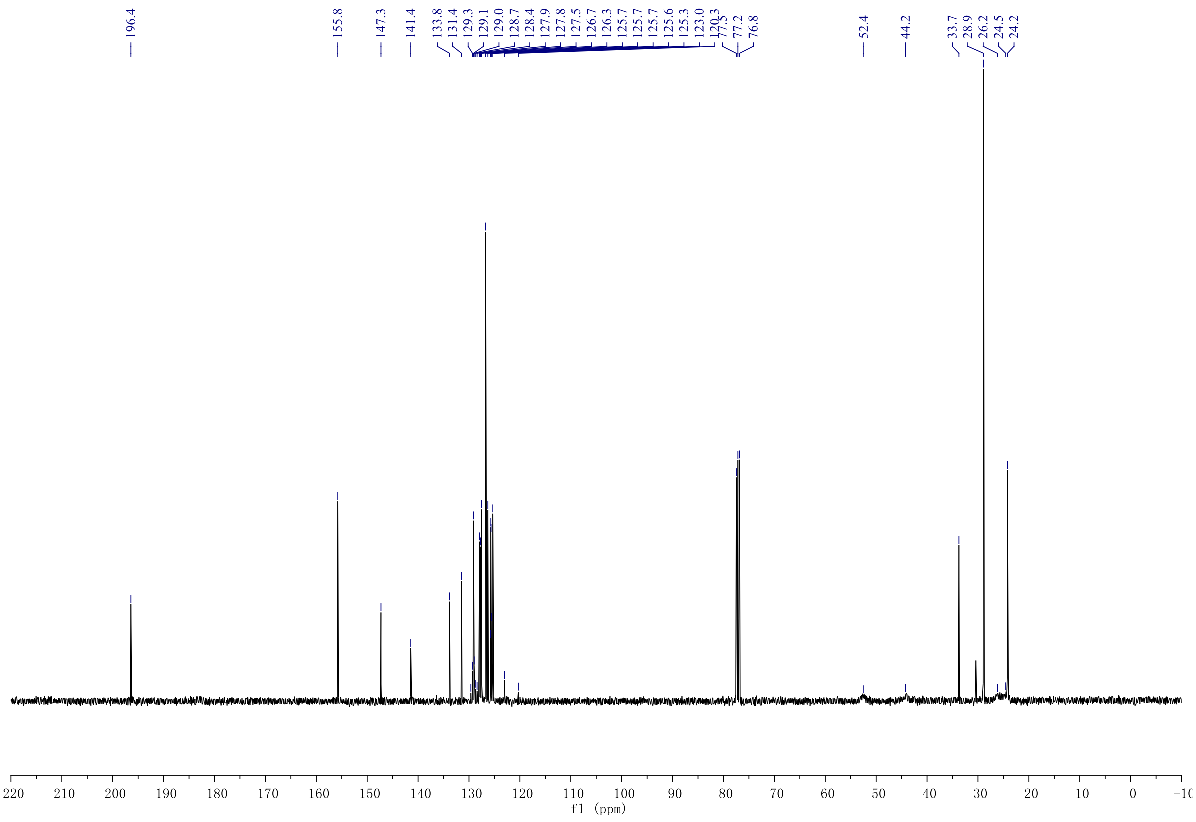

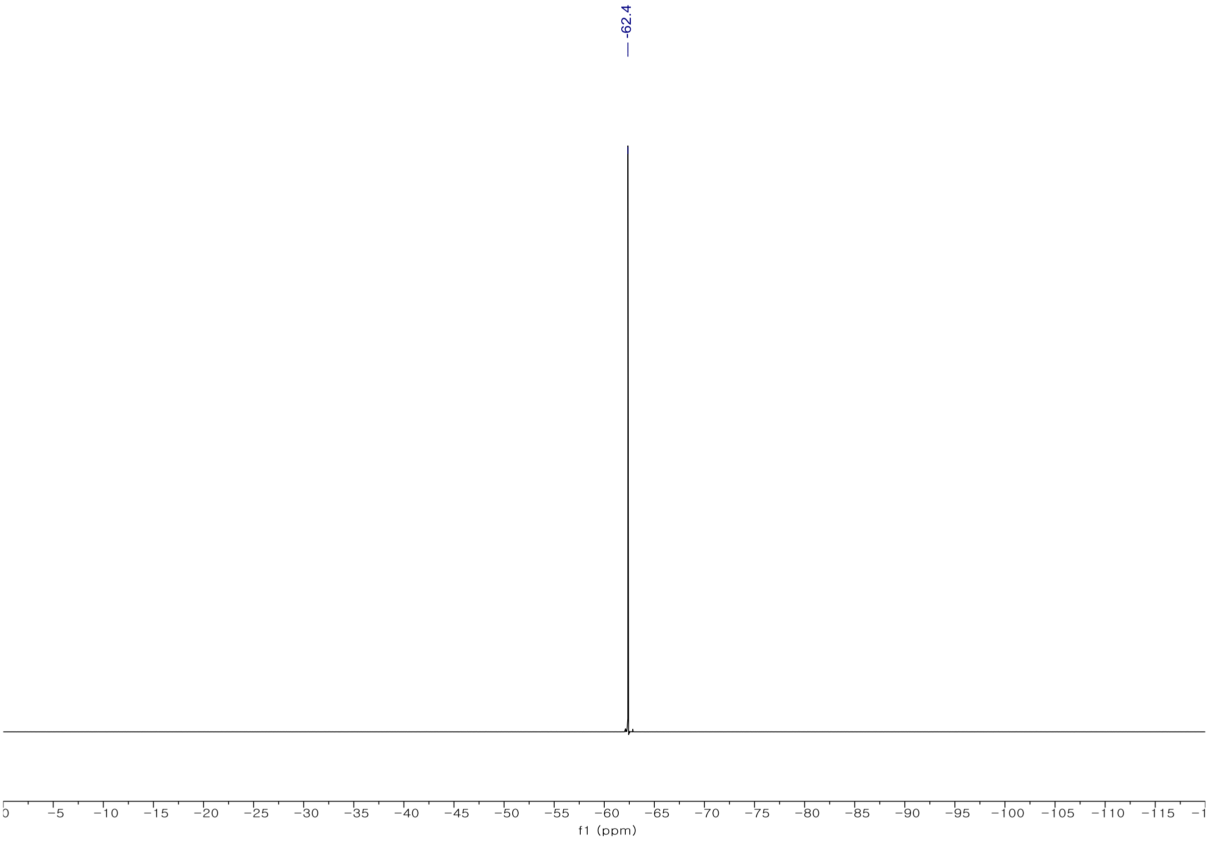
-62.4
0 −5 −10 −15 −20 −25 −30 −35 −40 −45 −50 −55 −60 −65 −70 −75 −80 −85 −90 −95 −100 −105 −110 −115
f1 (ppm)

(*E*)-Butyl 3-{3-[(*E*)-4,4-dimethylpent-2-enoyl]-5-methylphenyl}acrylate (6a)

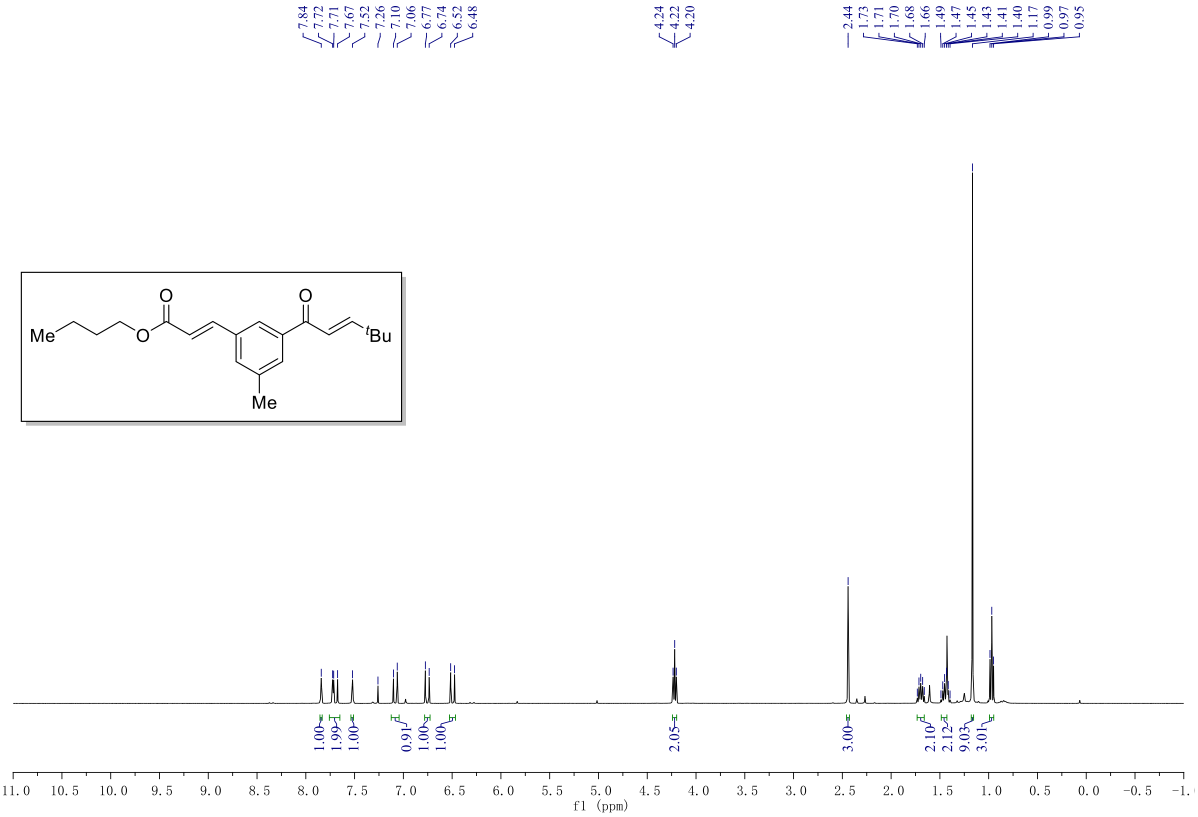

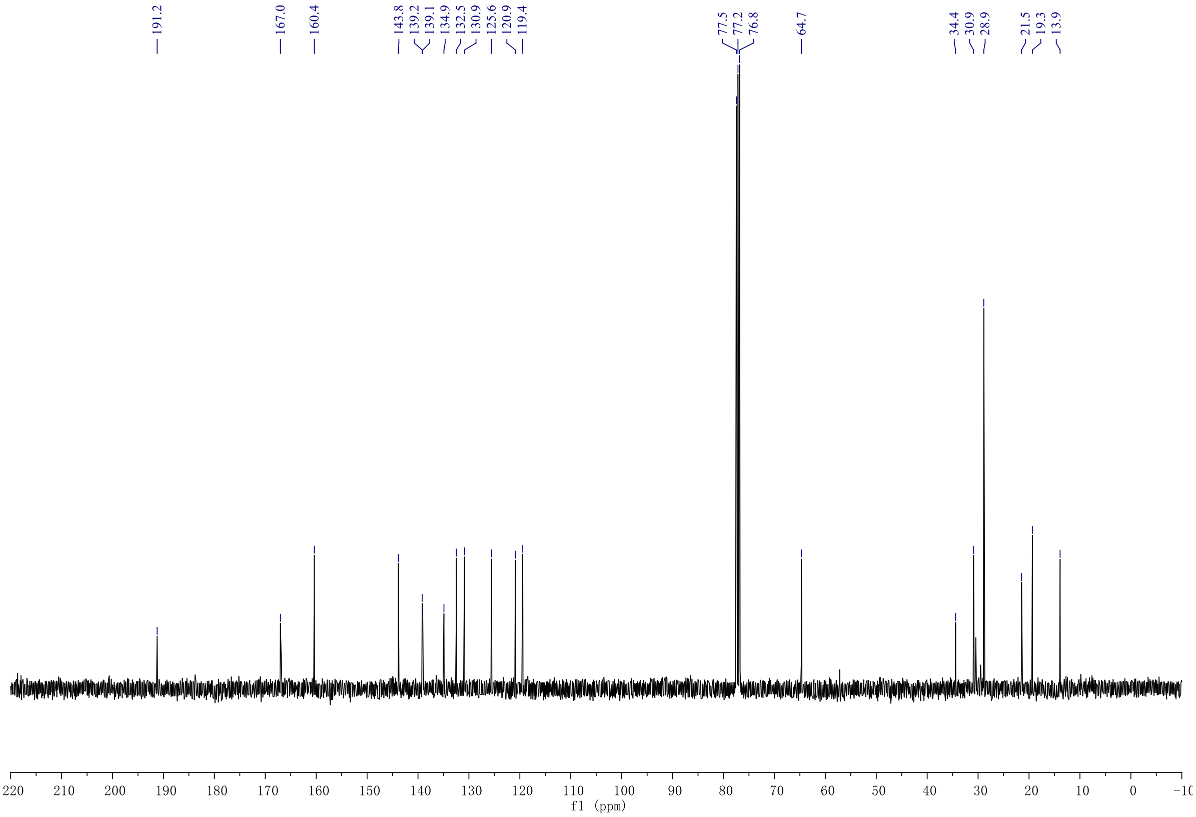

(*E*)-Butyl 3-(3-cinnamoylphenyl)acrylate (6b)

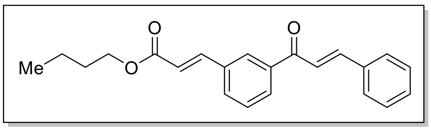

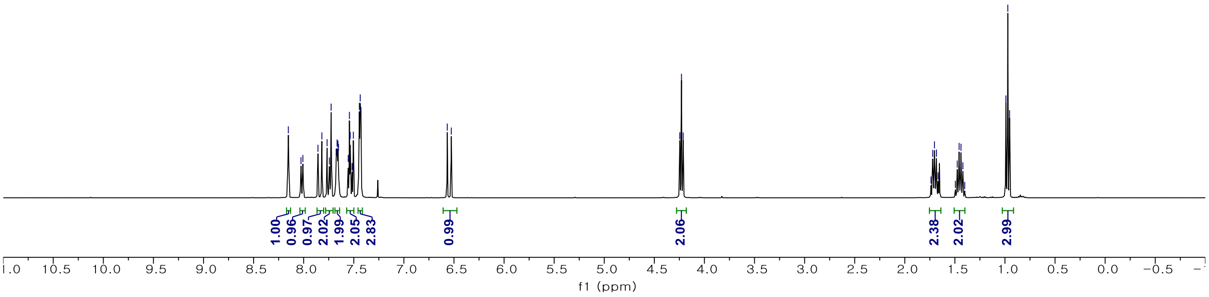

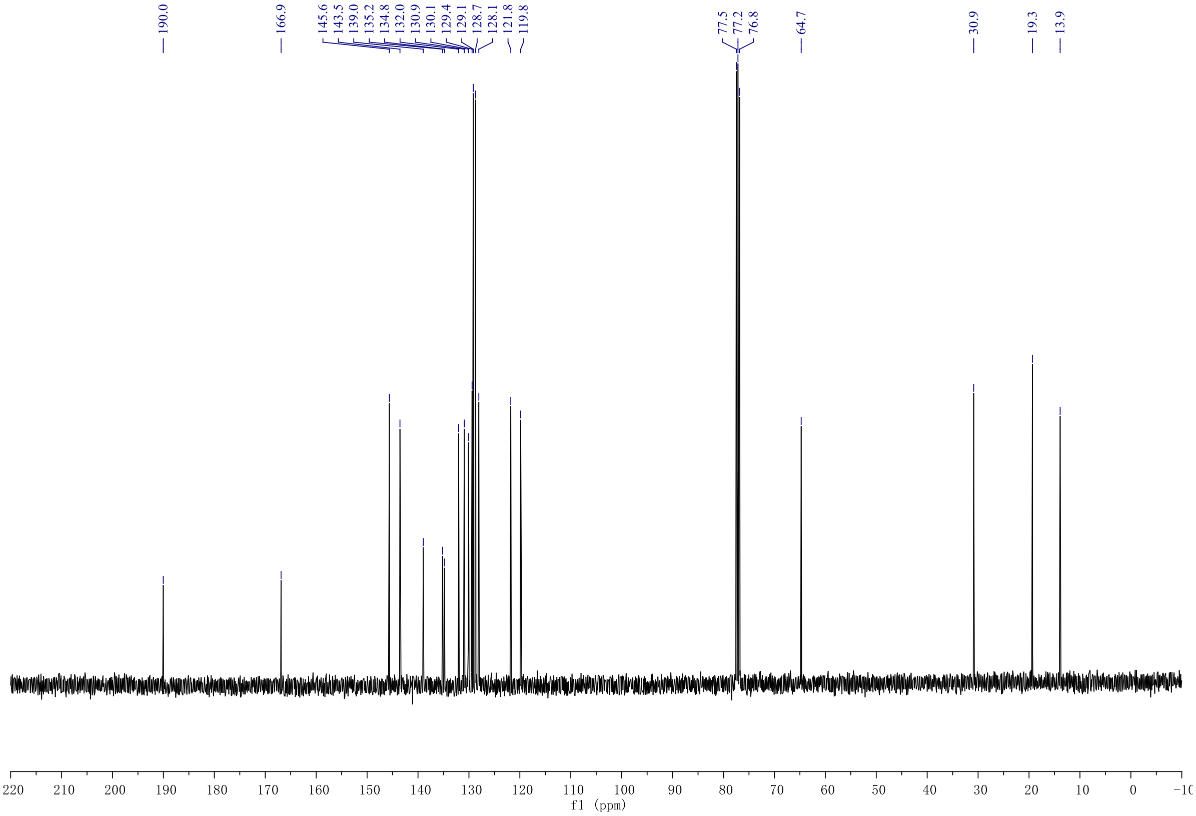

(*E*)-1-{5-Bromo-2-[(*E*)-piperidin-1-yldiazenyl]phenyl}-4,4-dimethylpent-2-en-1-one (S2)

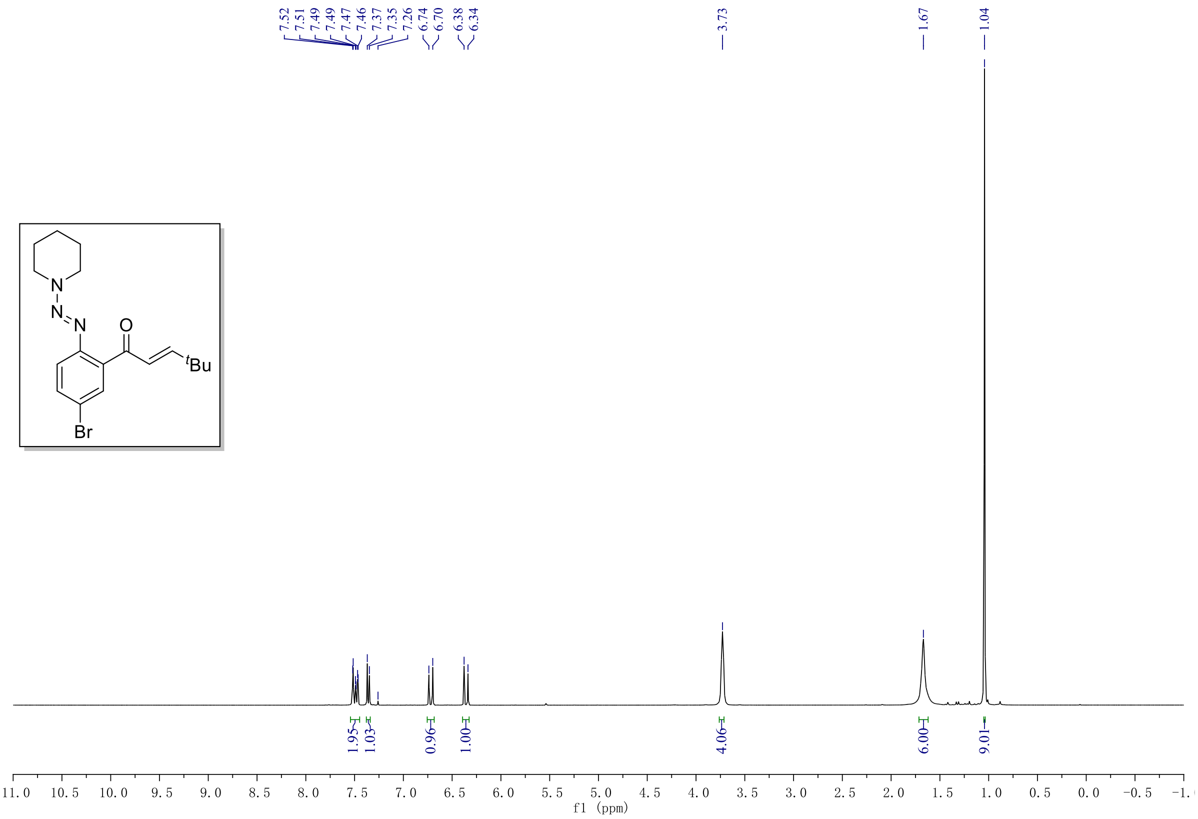

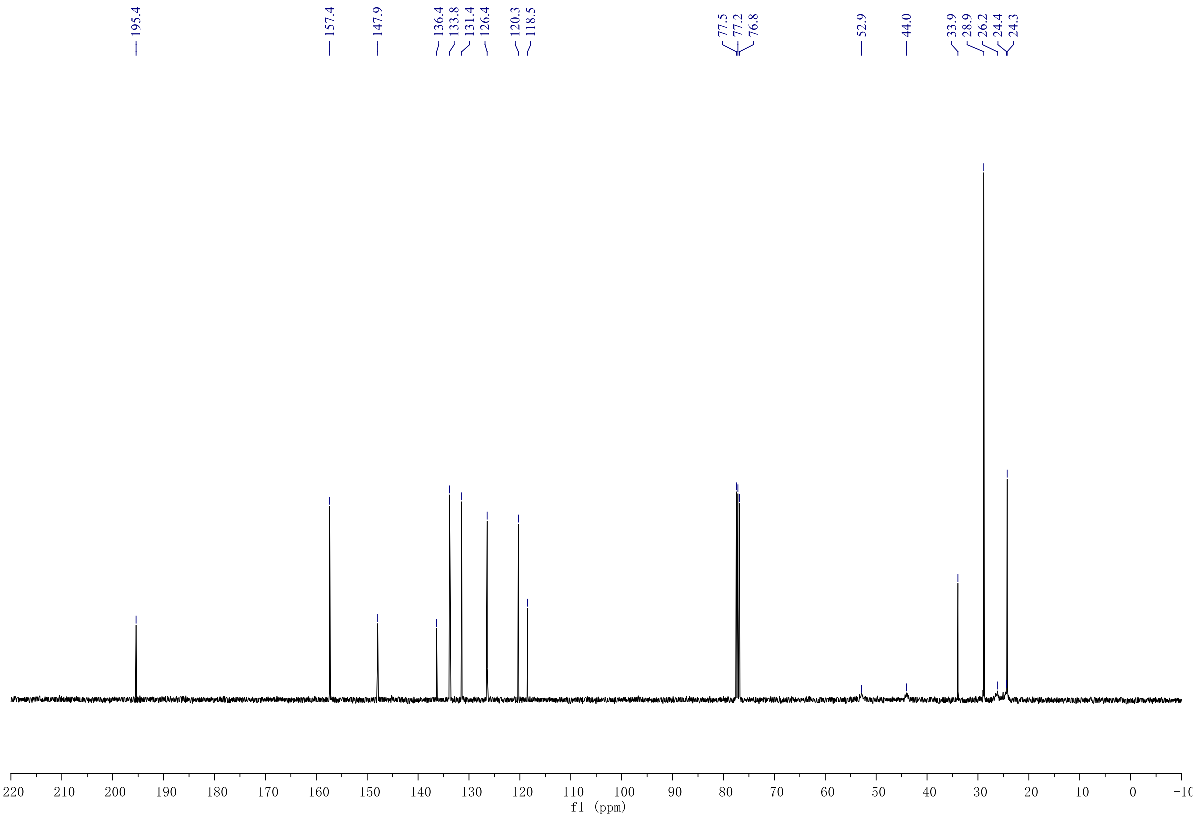

(*E*)-1-(3-Bromophenyl)-4,4-dimethylpent-2-en-1-one (7a)

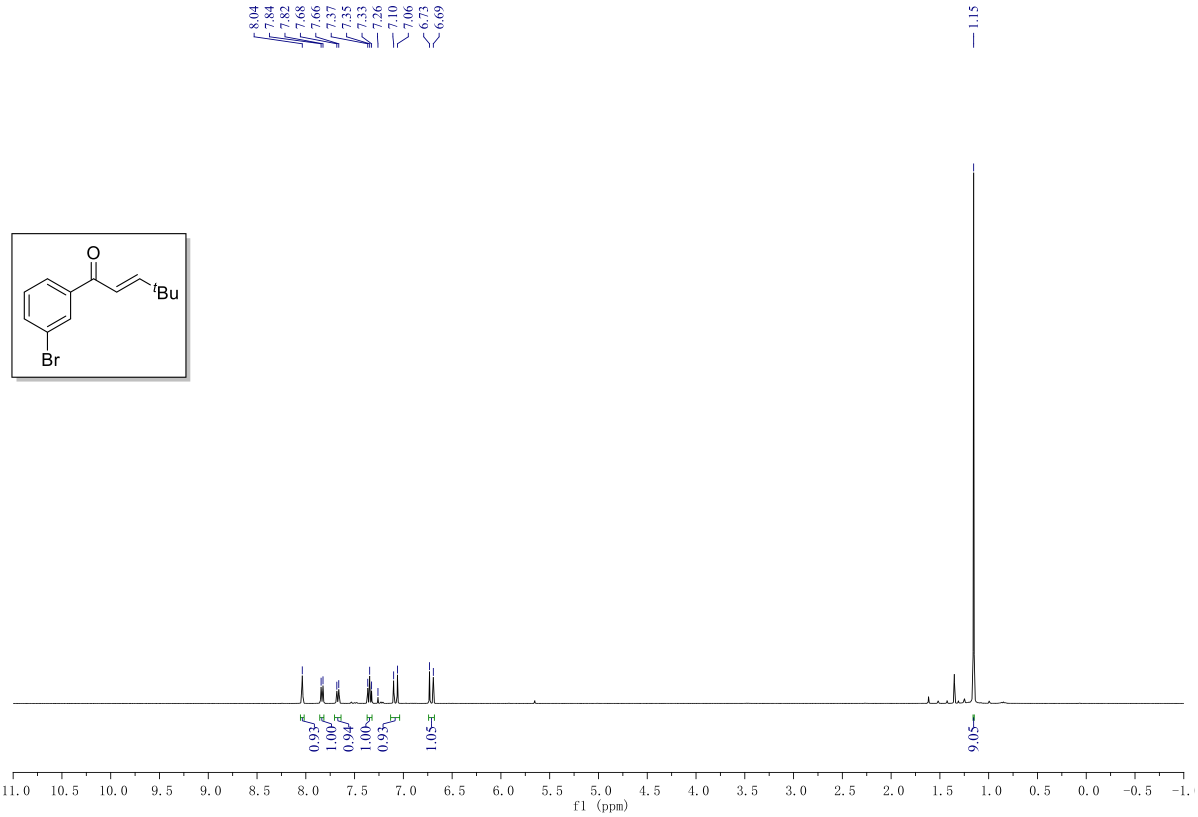

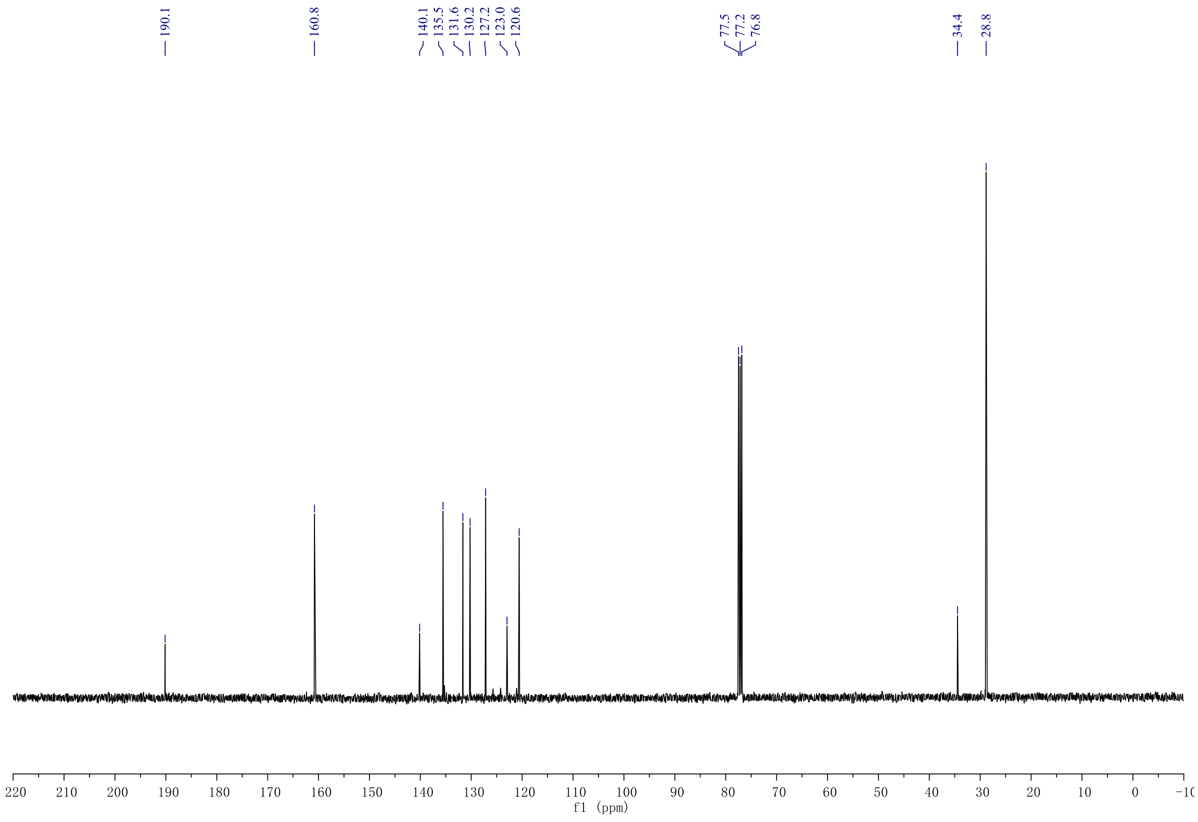

(*E*)-1-(3-Bromophenyl)non-2-en-1-one (7b)

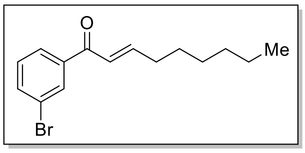

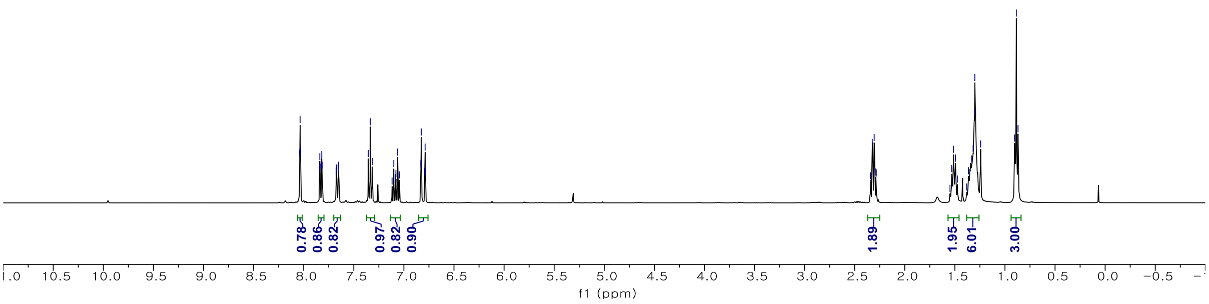

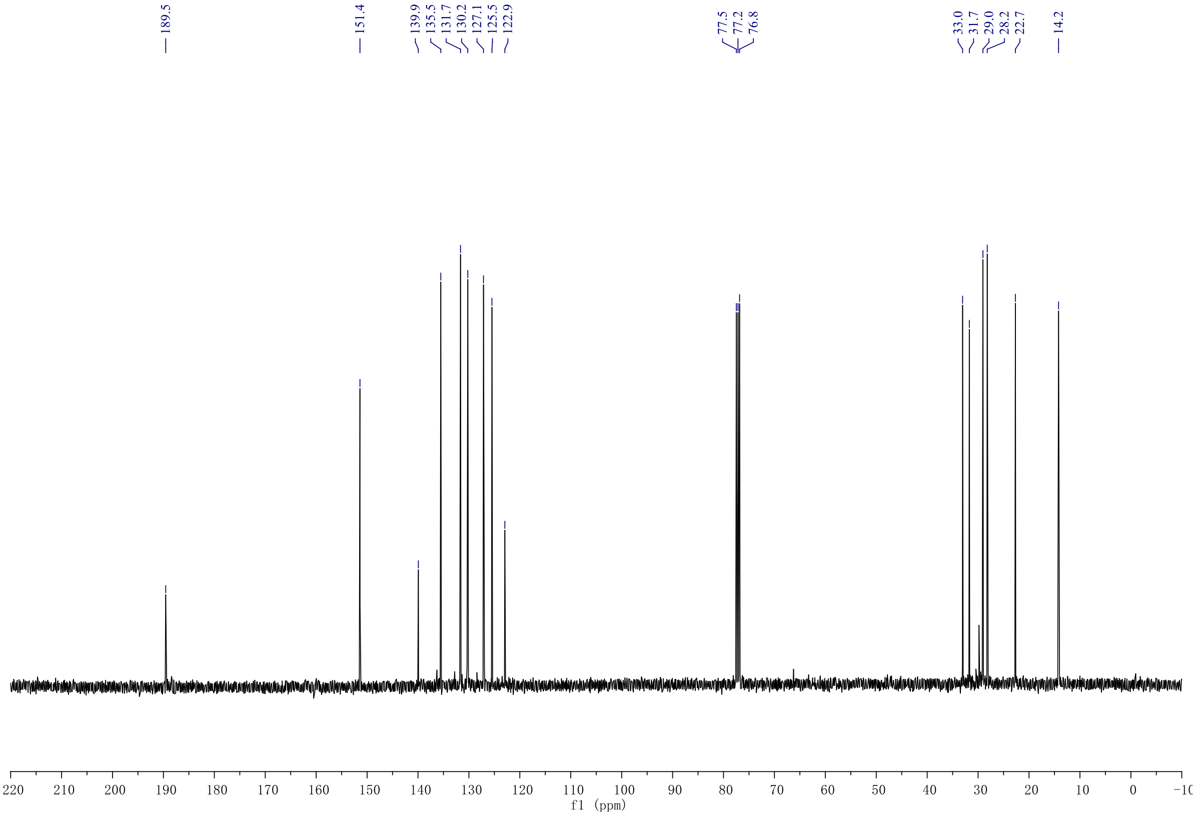

(*E*)-1-{5-Bromo-2-[(*E*)-piperidin-1-yldiazenyl]phenyl}-3-cyclopentylprop-2-en-1-one (S3)

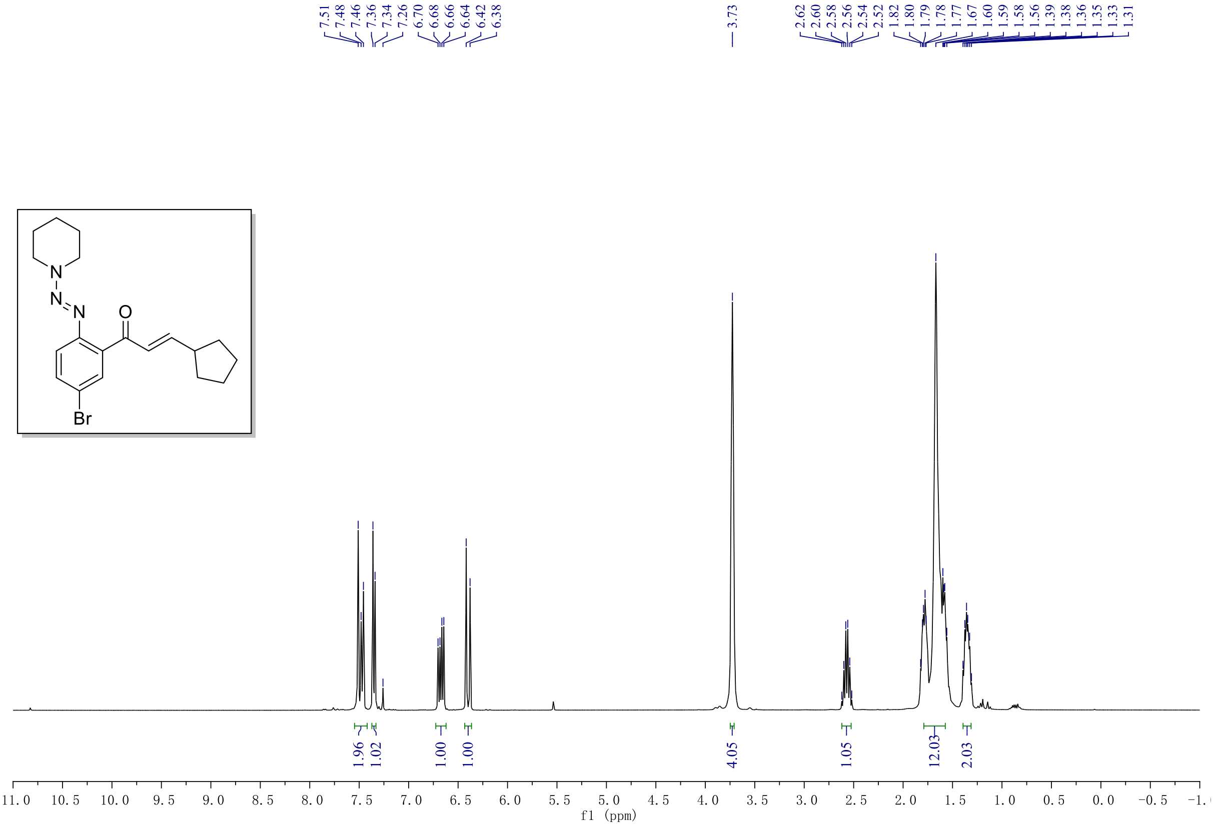

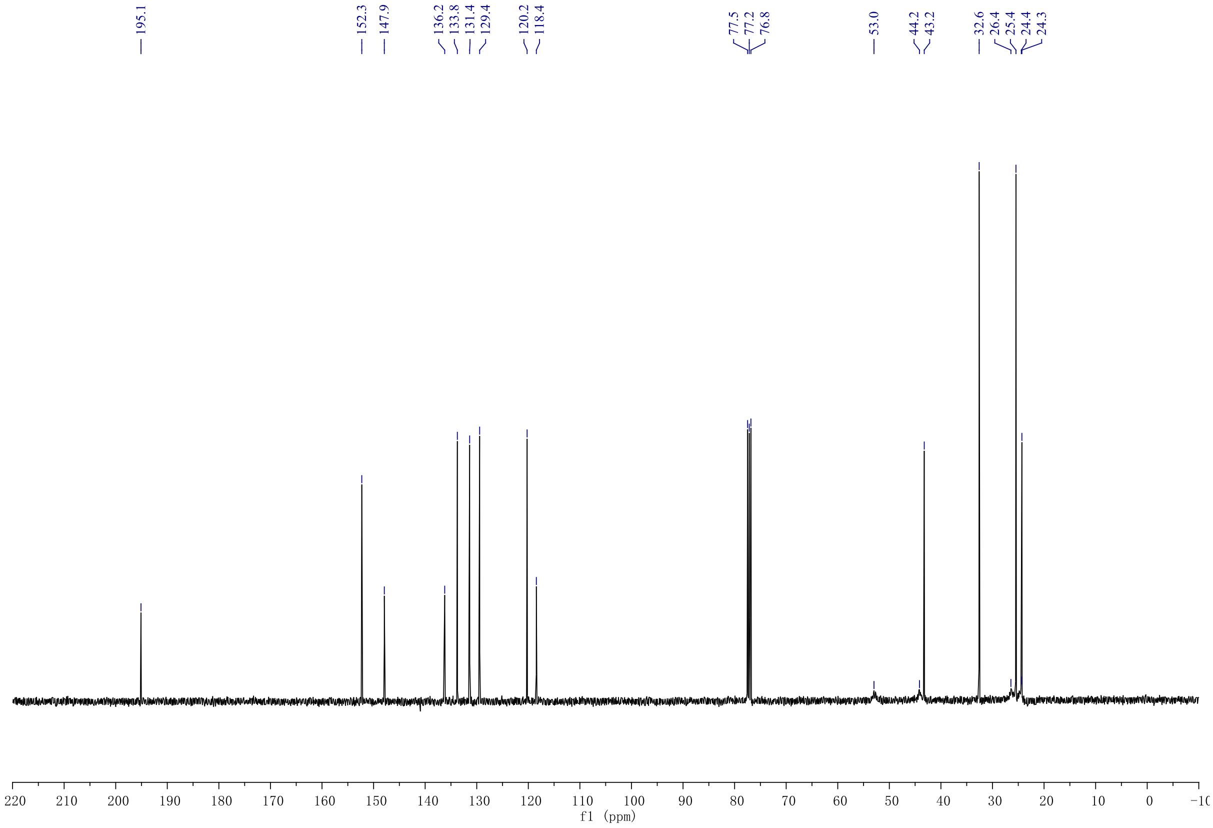

(*E*)-1-(3-Bromophenyl)-3-cyclopentylprop-2-en-1-one (7c)

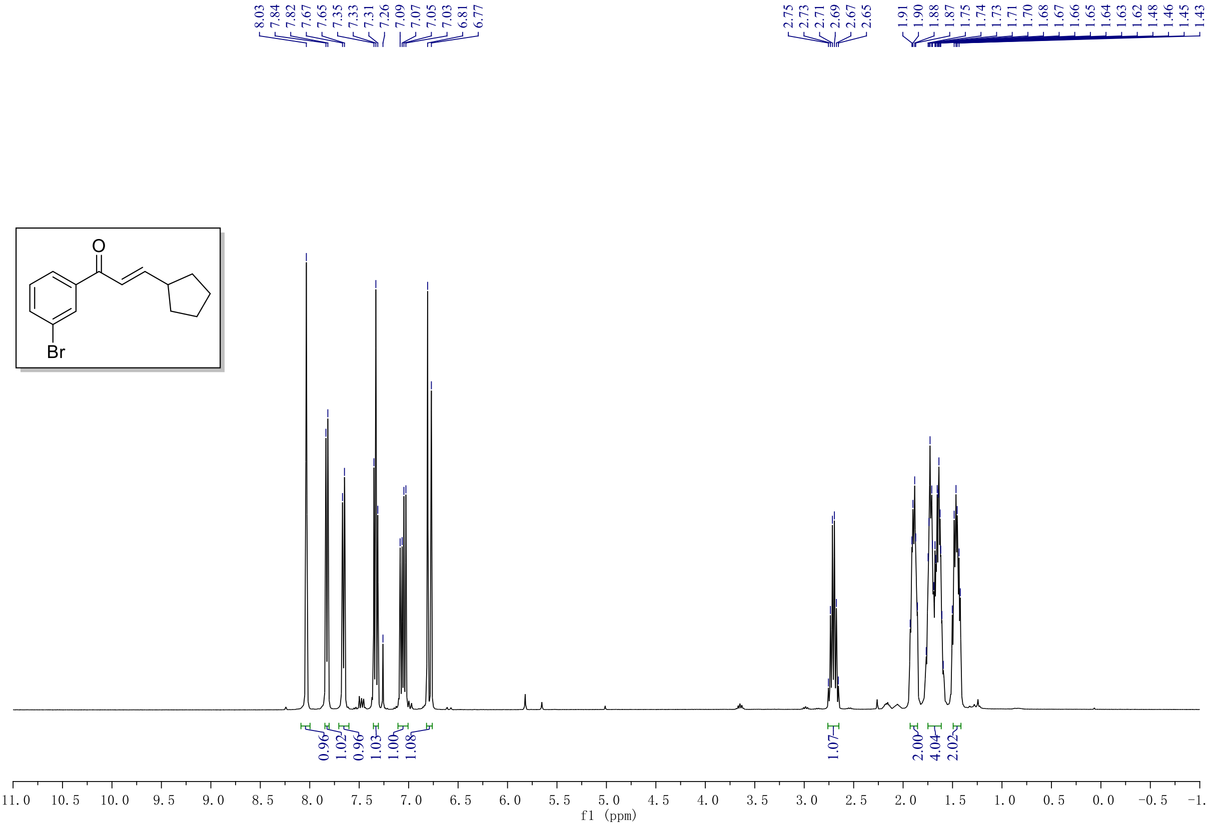

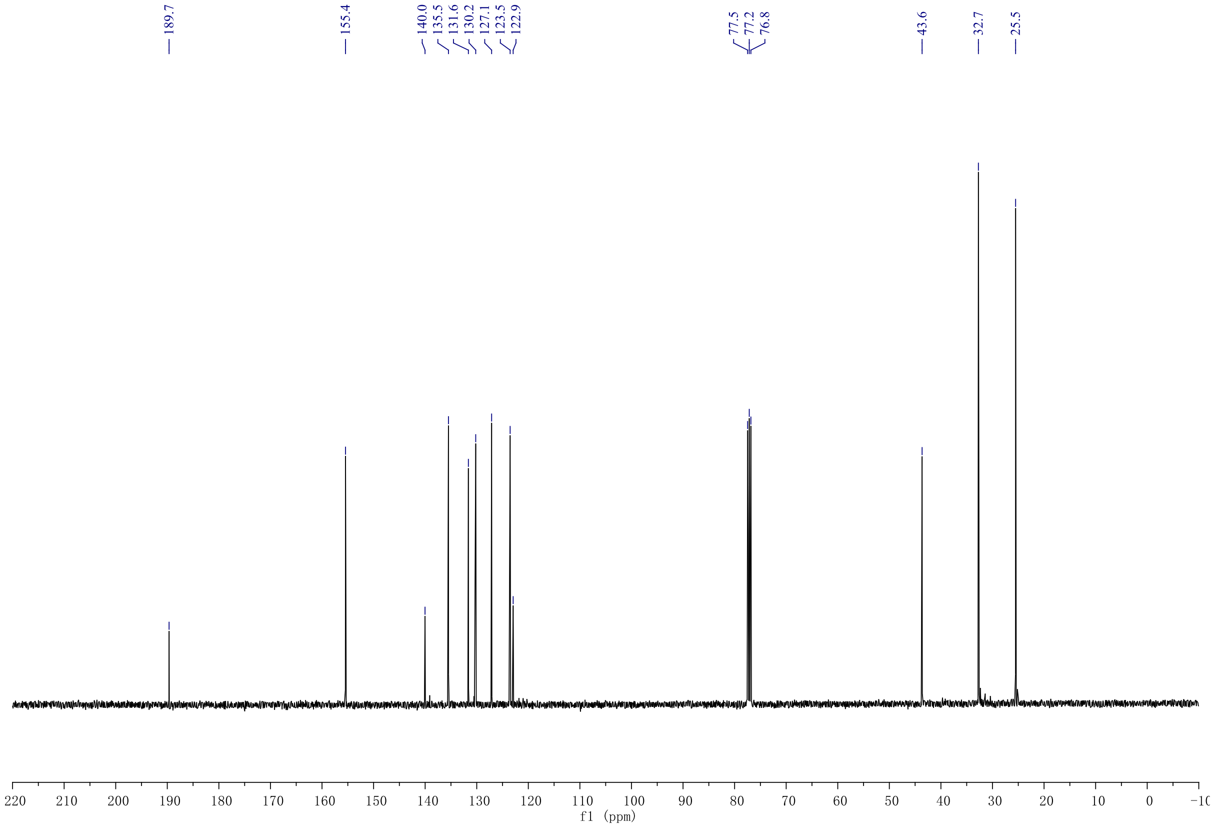

(*E*)-1-(3-Bromophenyl)-5-phenylpent-2-en-1-one (7d)

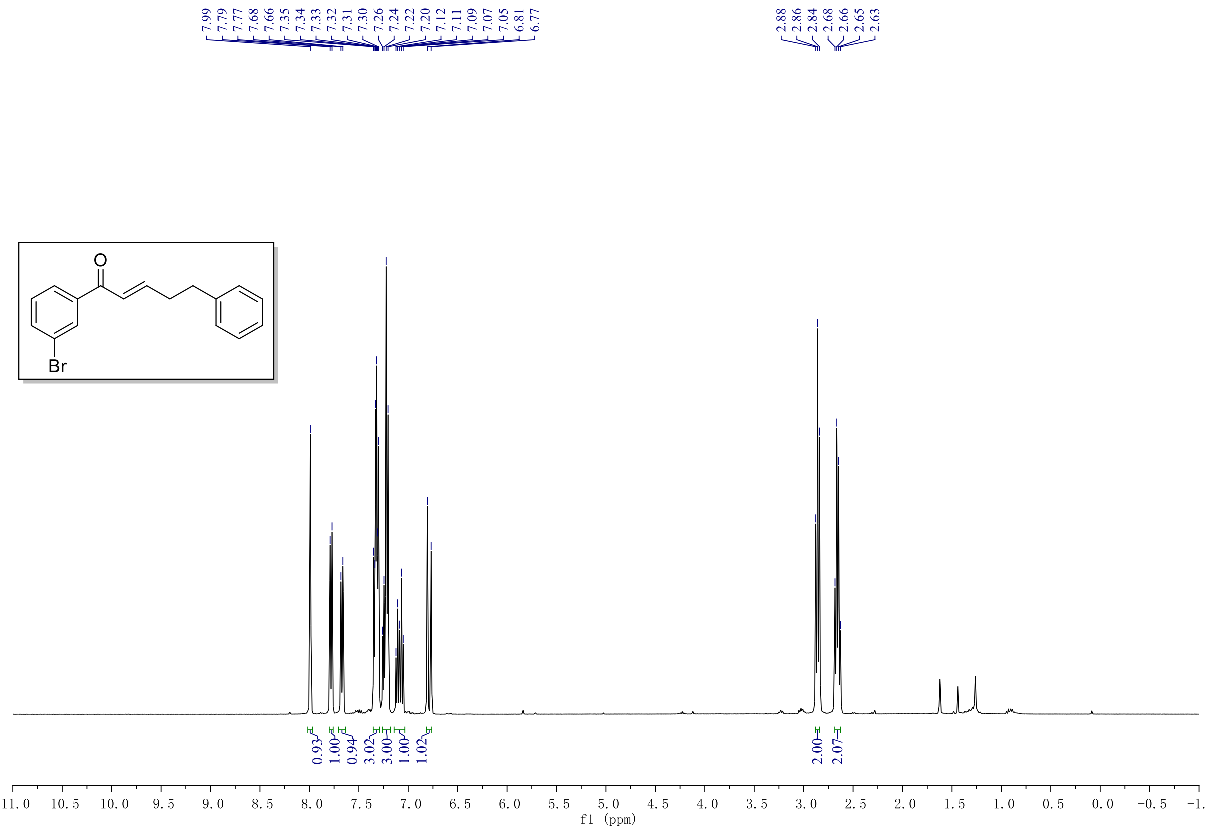

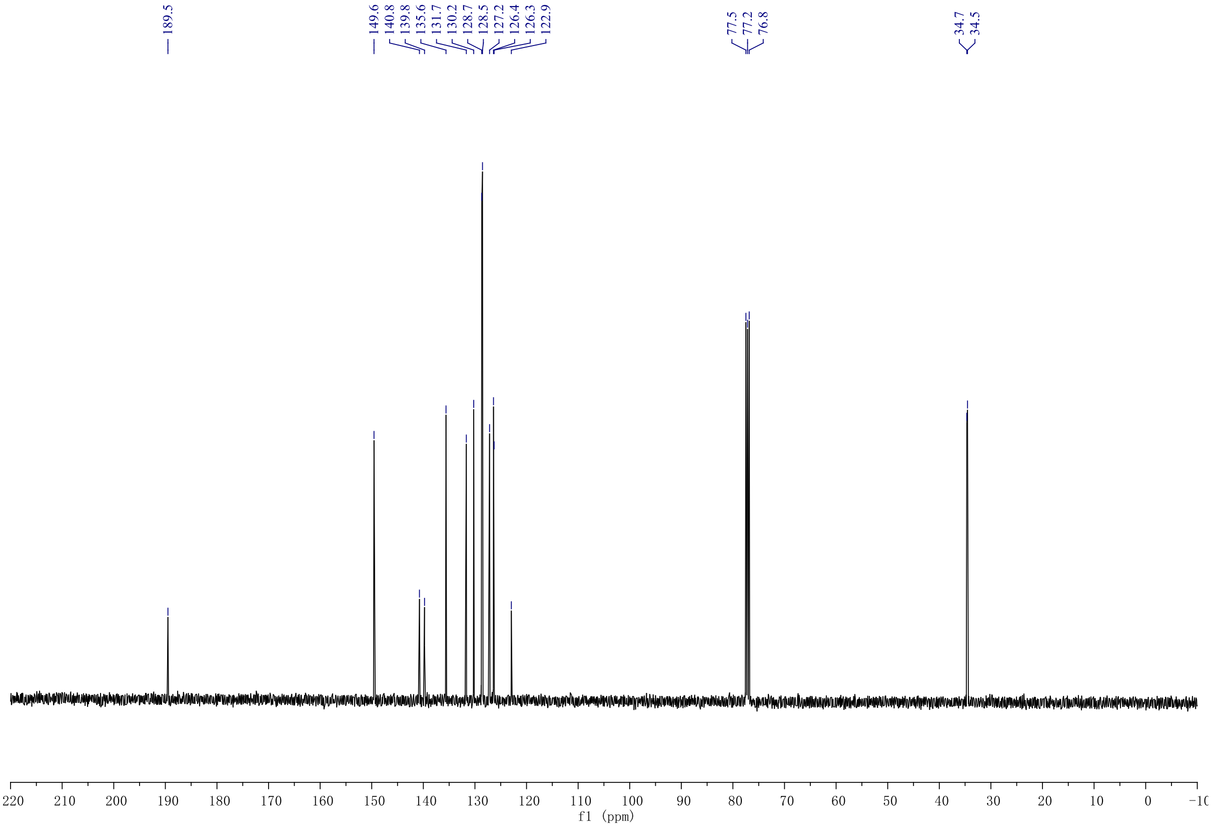

2-{(*E*)-7-[5-Bromo-2-((*E*)-piperidin-1-yldiazenyl)phenyl]-7-oxohept-5-en-1-yl}isoindoline-1,3-dione (S4)

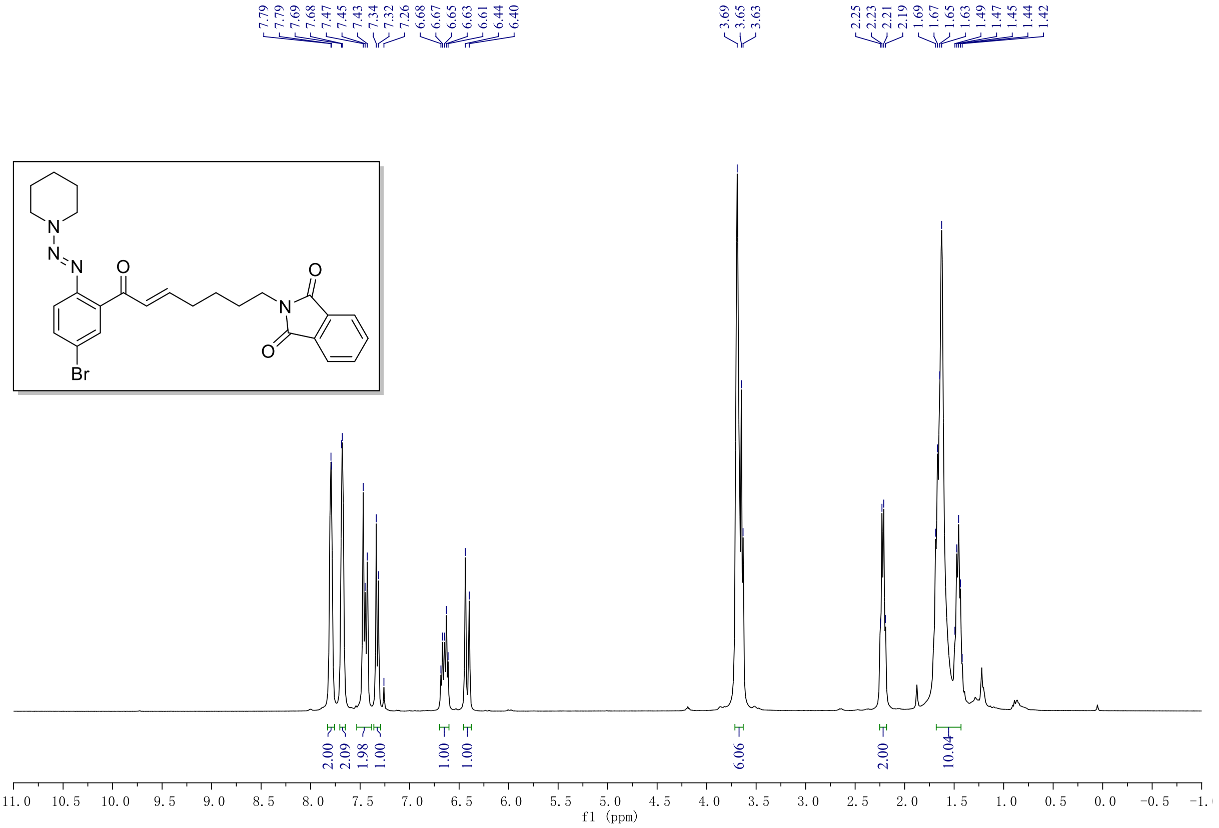

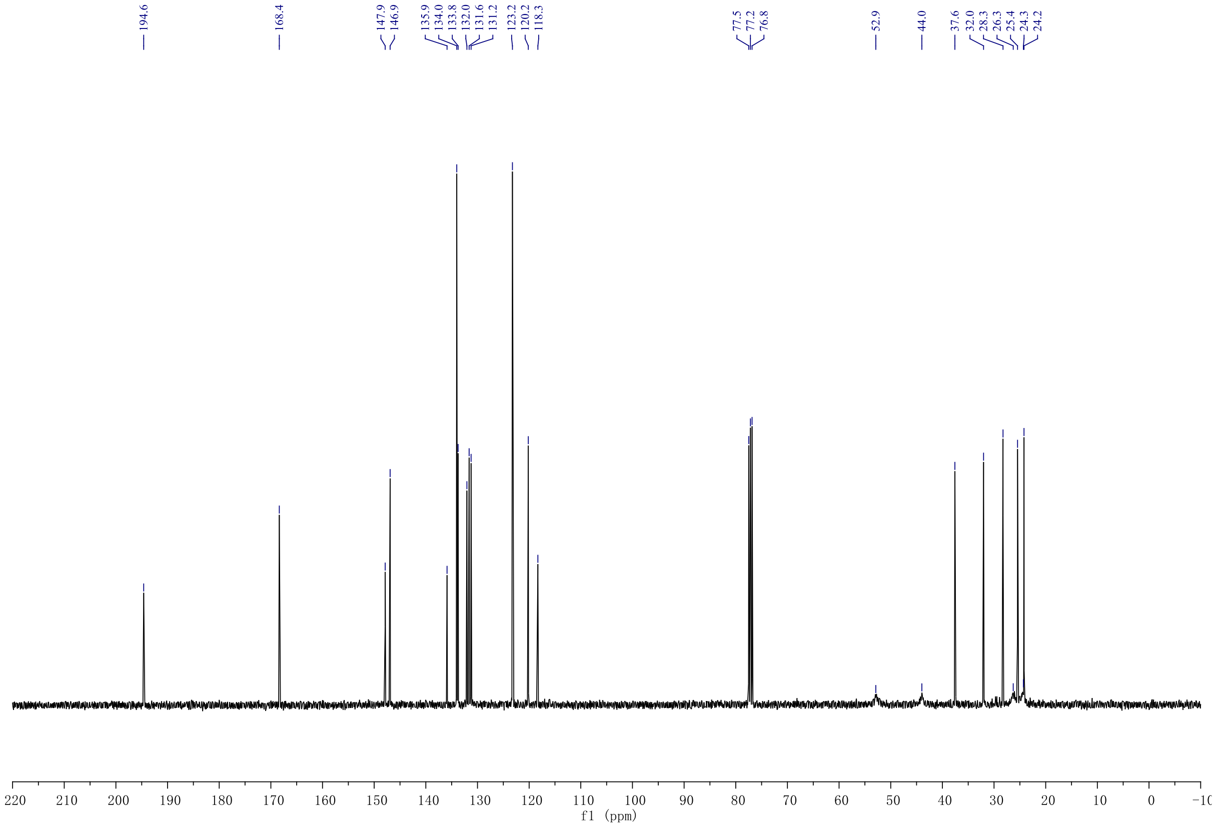

(*E*)-2-[7-(3-Bromophenyl)-7-oxohept-5-en-1-yl]isoindoline-1,3-dione (7e)

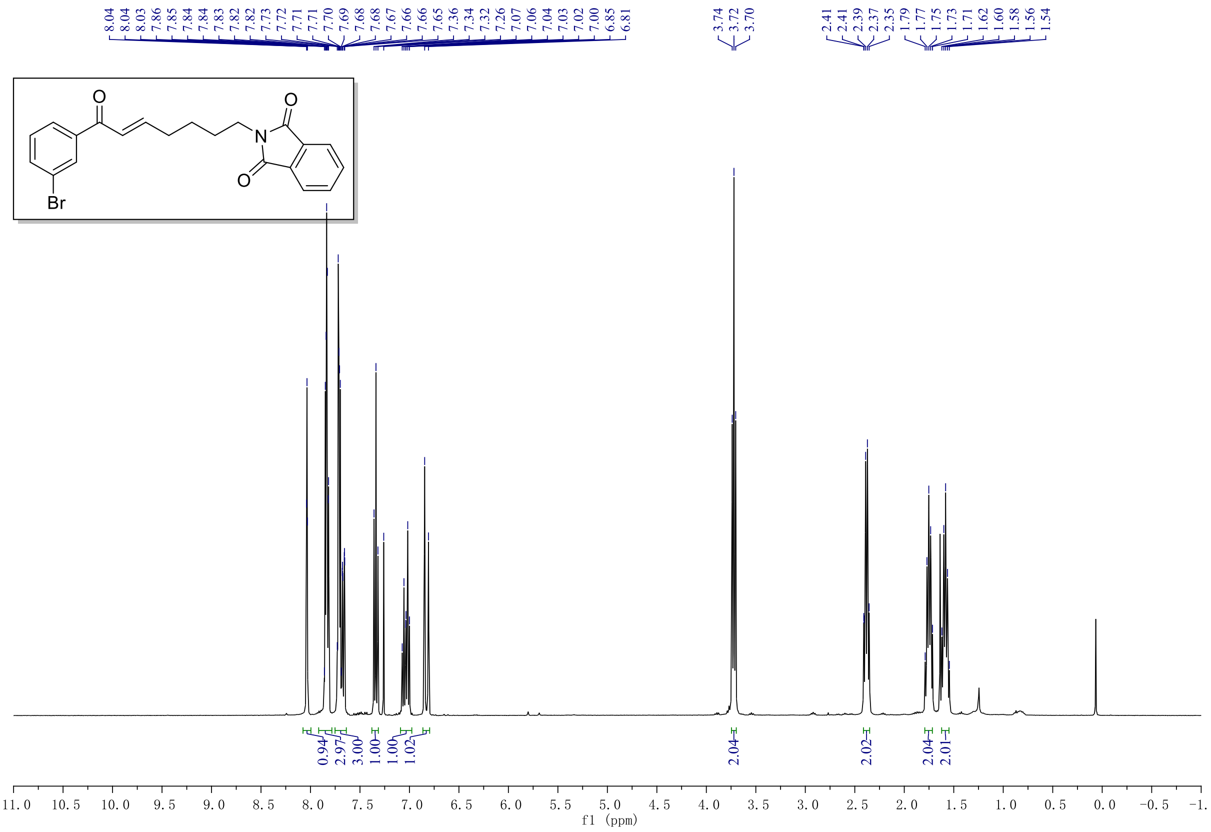

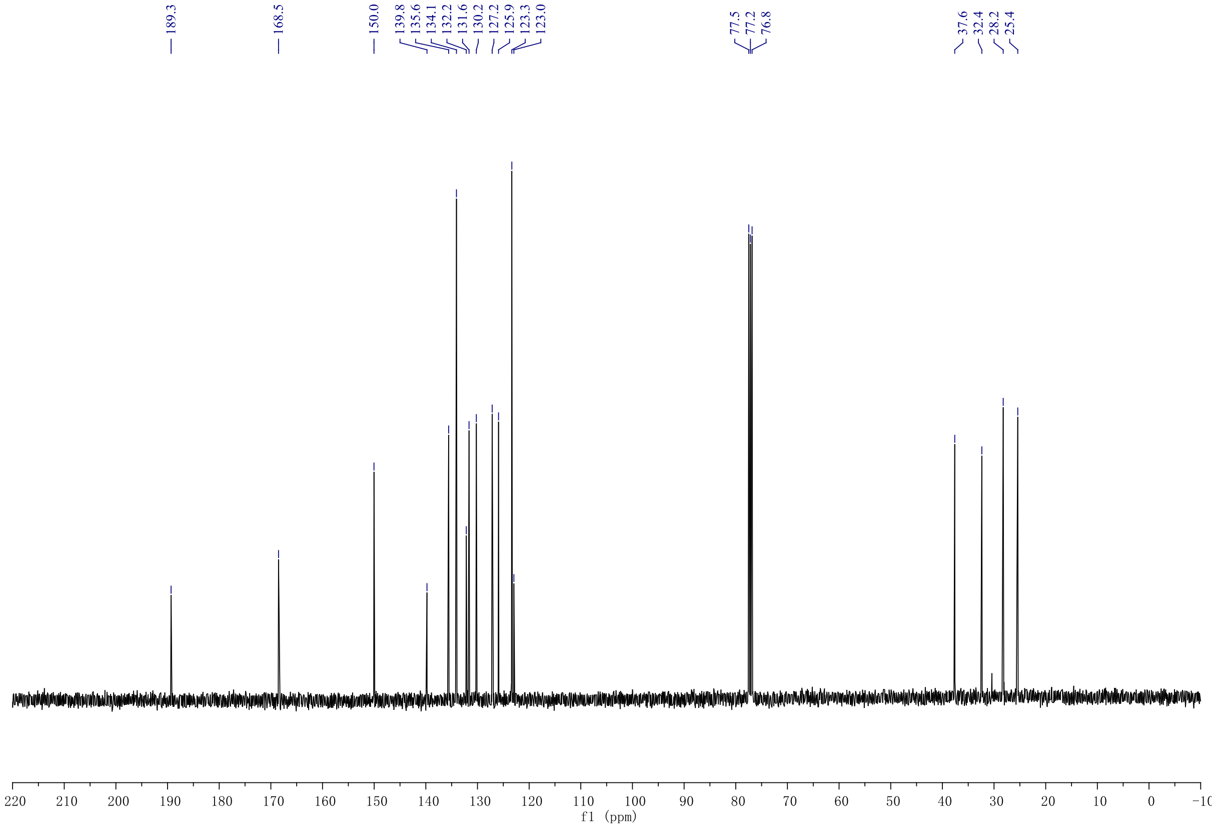

(*E*)-1-{5-Bromo-4-fluoro-2-[(*E*)-piperidin-1-yldiazenyl]phenyl}-4,4-dimethylpent -2-en-1-one (S5)

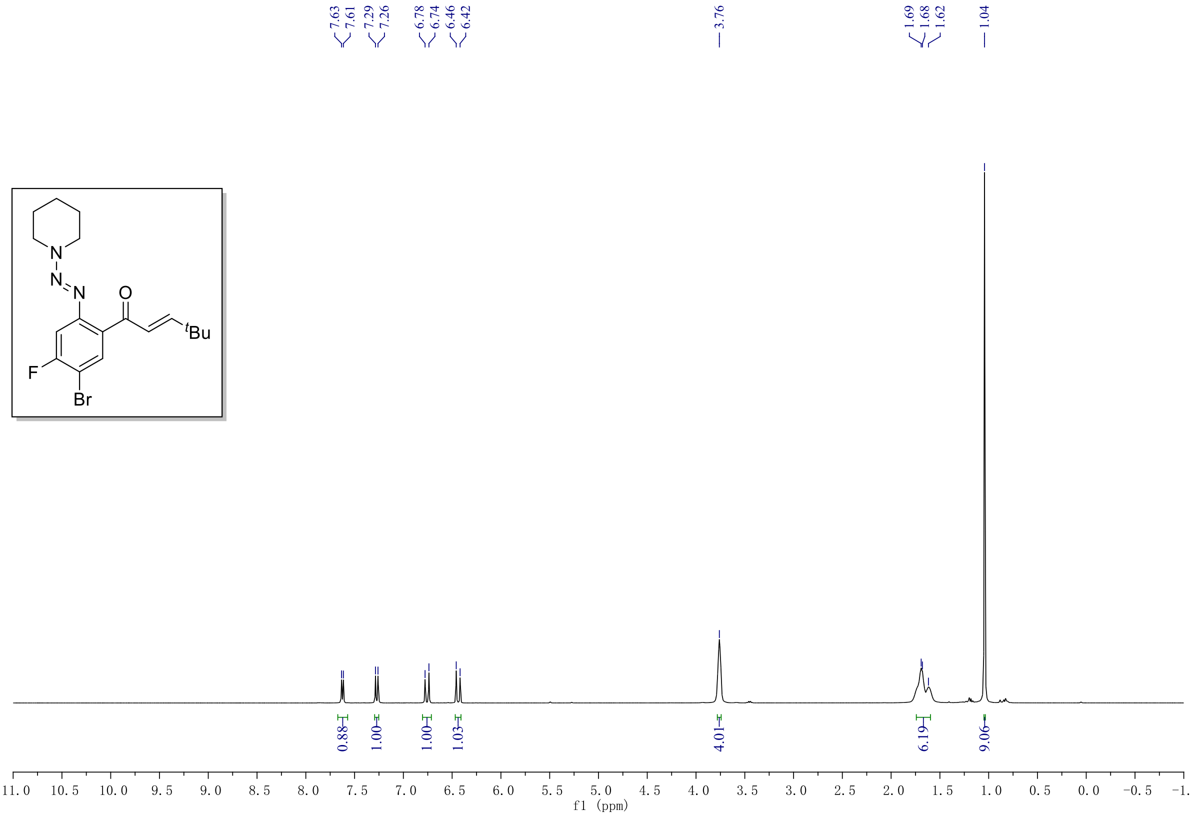

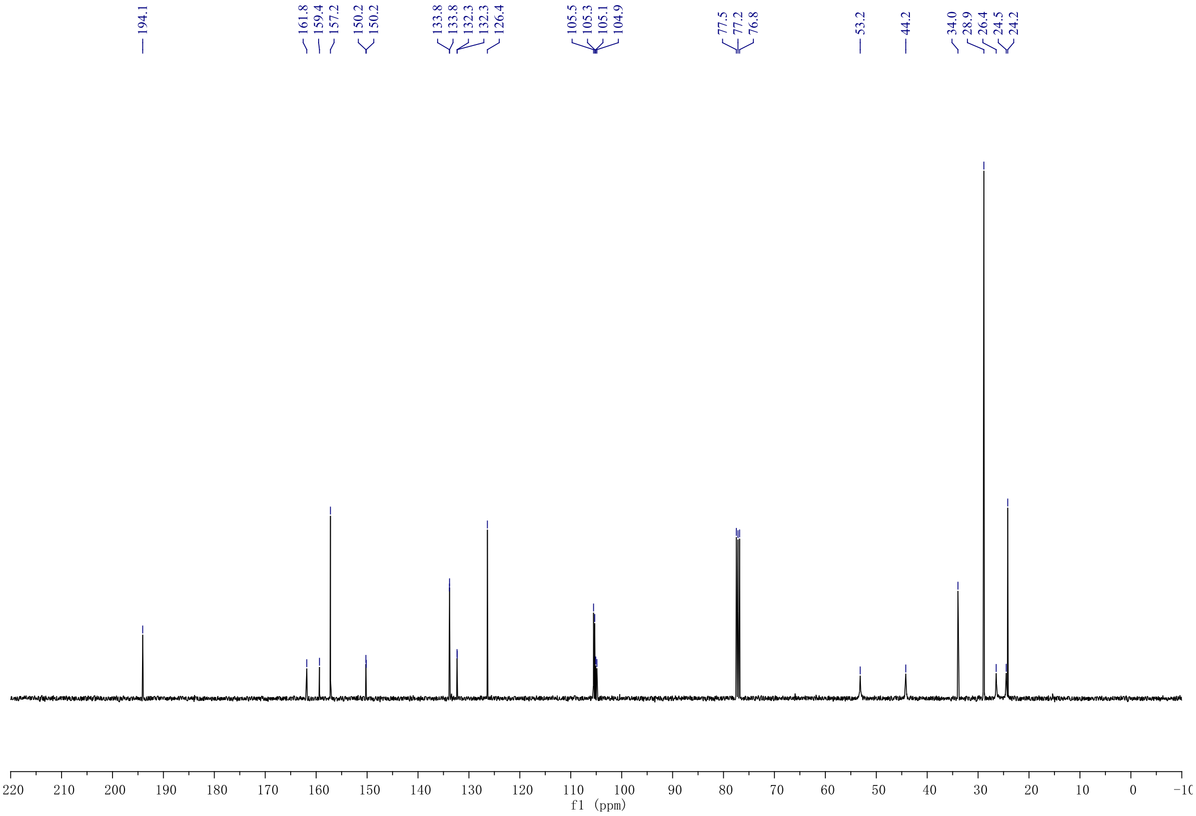

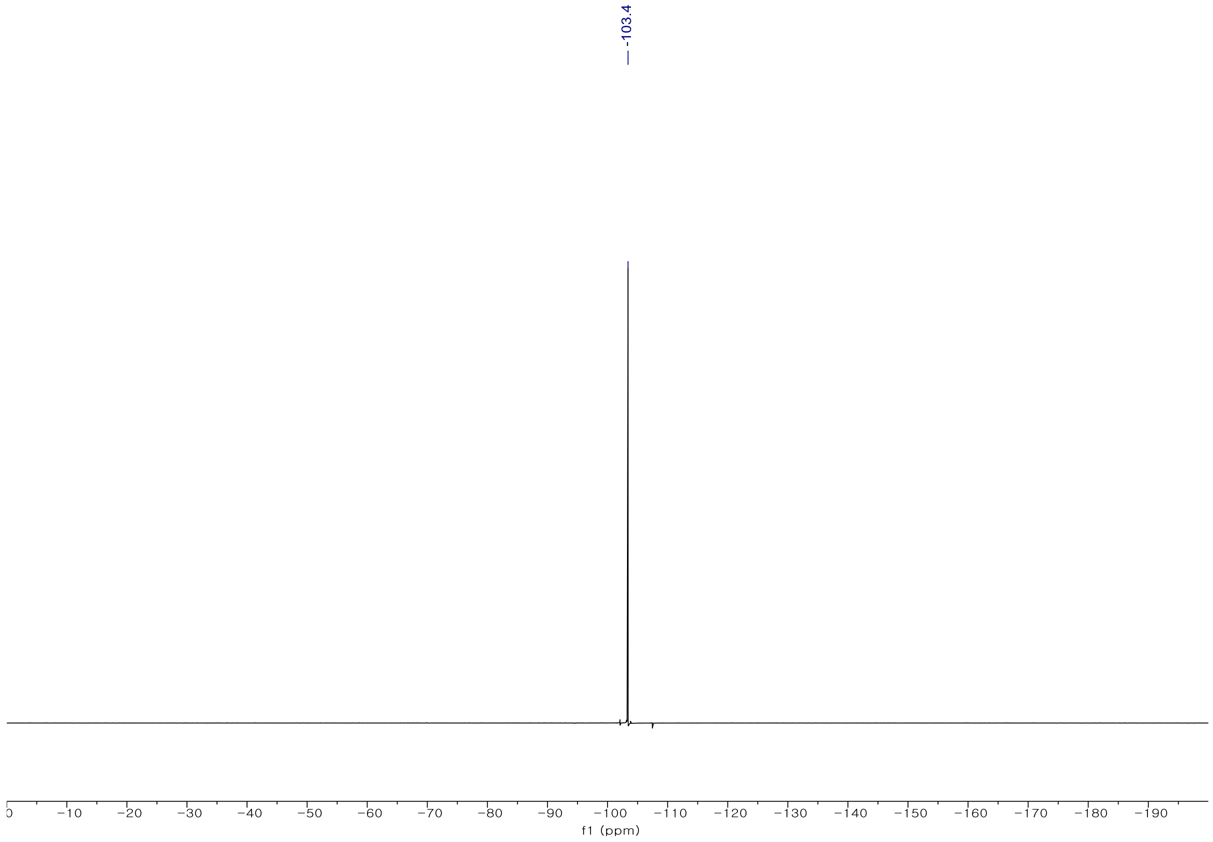
-103.4
0
−10
−20
−30
−40
−50
−60
−70
−80
−90
−100
−110
−120
−130
−140
−150
−160
−170
−180
−190
f1 (ppm)

(*E*)-1-(3-Bromo-4-fluorophenyl)-4,4-dimethylpent-2-en-1-one (7f)

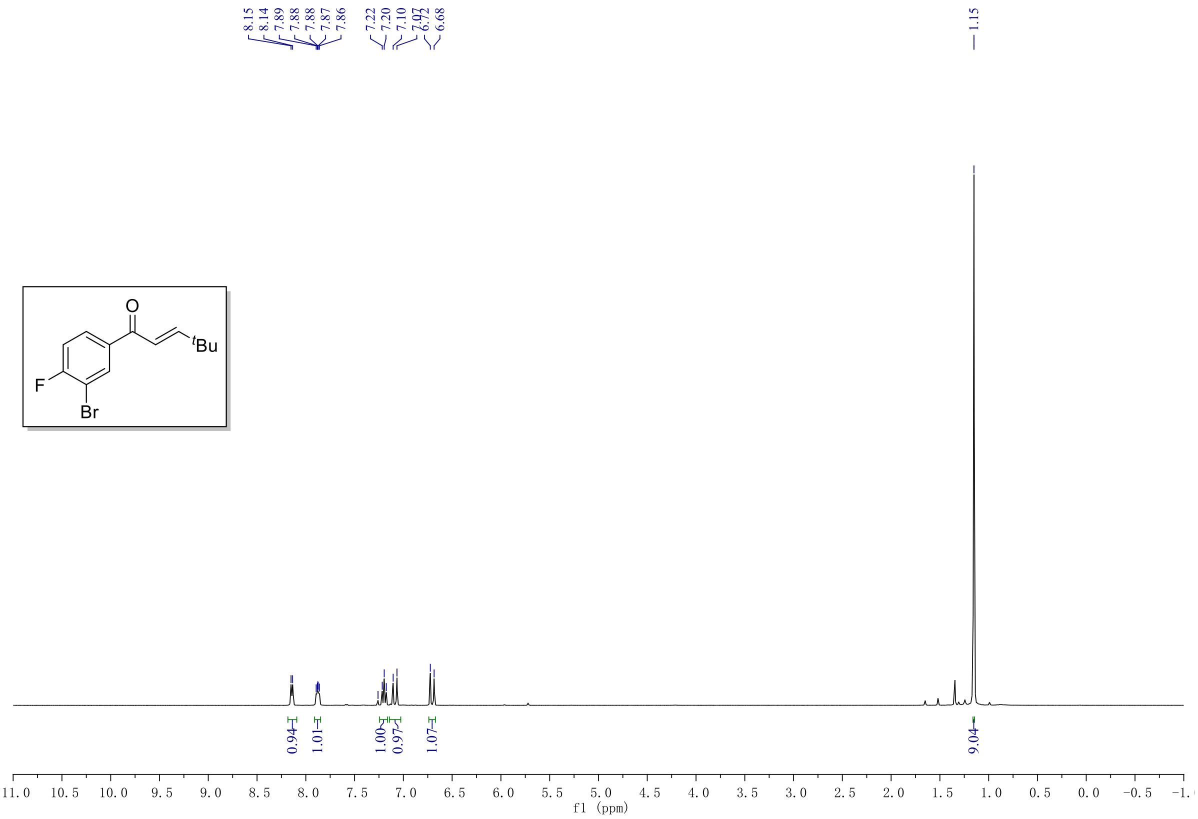

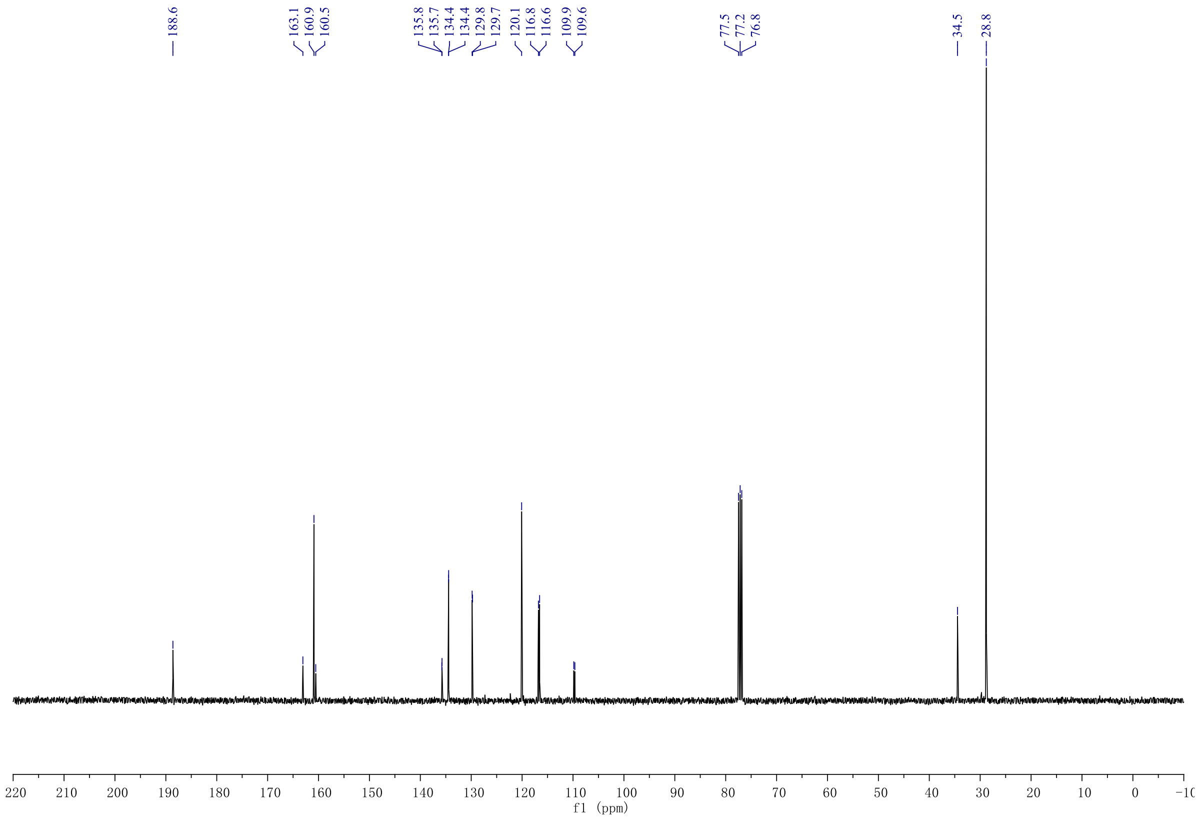

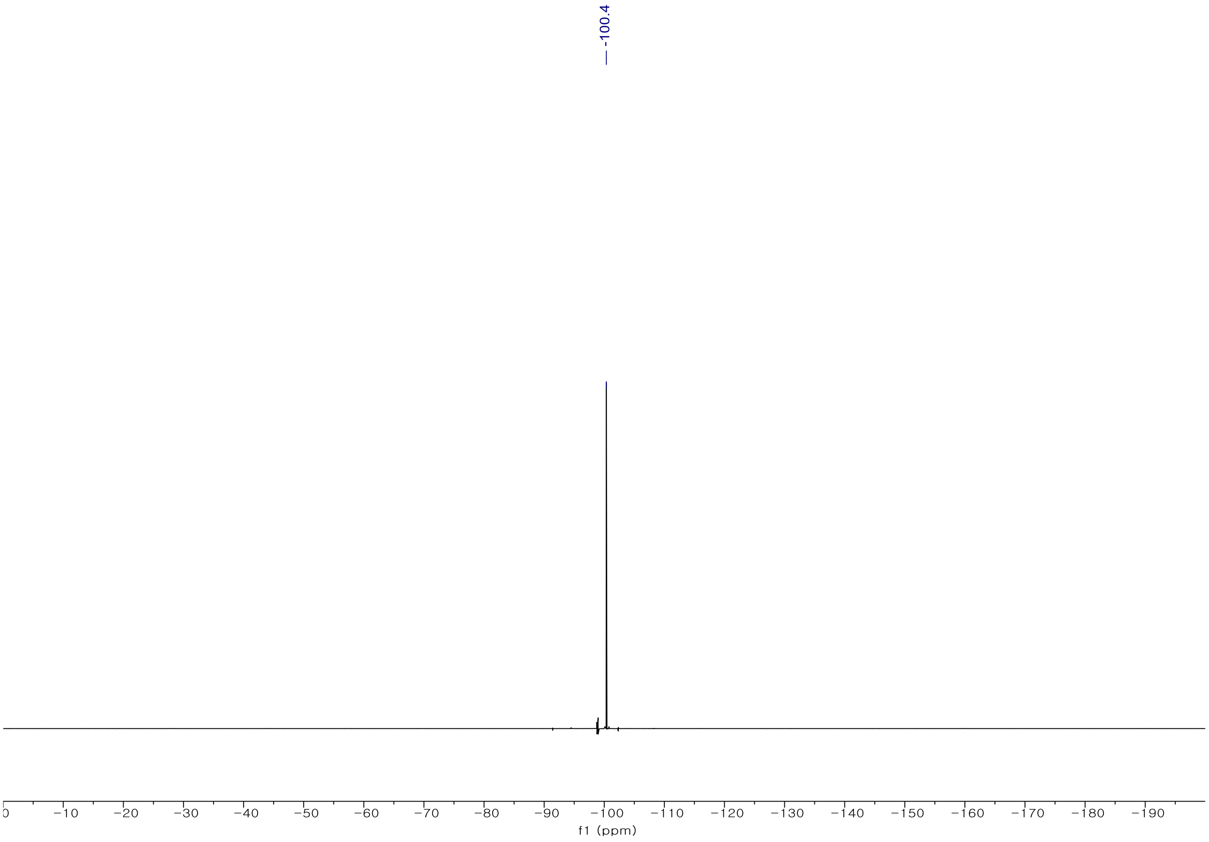
-100.4
0
-10
-20
-30
-40
-50
-60
-70
-80
-90
-100
-110
-120
-130
-140
-150
-160
-170
-180
-190
f1 (ppm)

(*E*)-1-{5-Bromo-4-chloro-2-[(*E*)-piperidin-1-yldiazenyl]phenyl}-4,4-dimethylpent-2-en-1-one (S6)

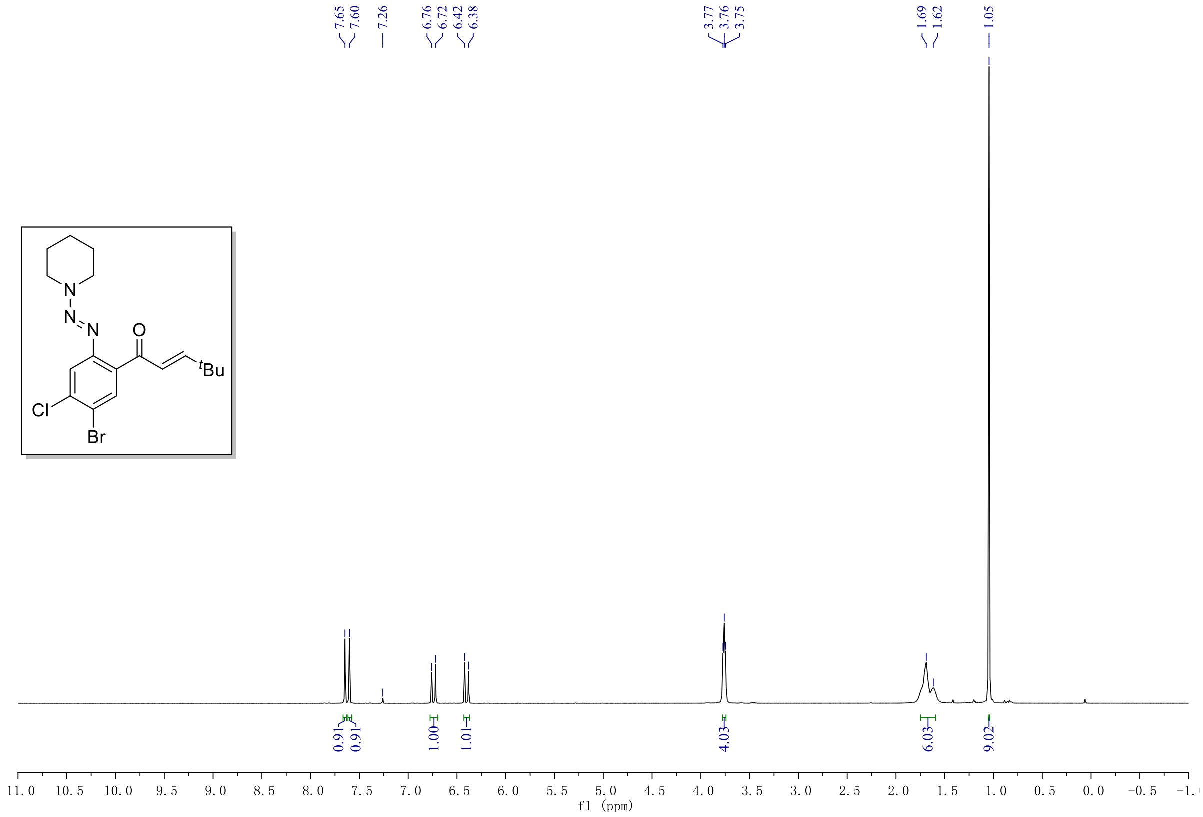

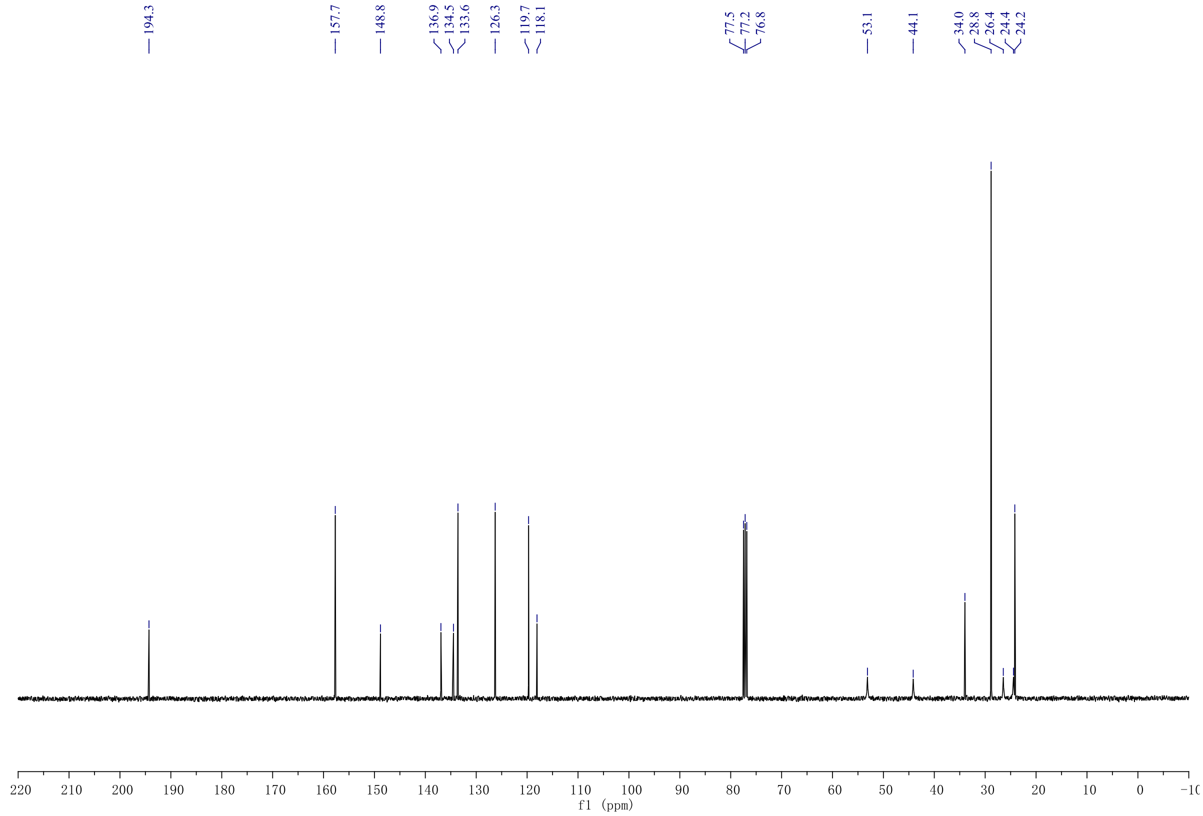

(*E*)-1-(3-Bromo-4-chlorophenyl)-4,4-dimethylpent-2-en-1-one (7g)

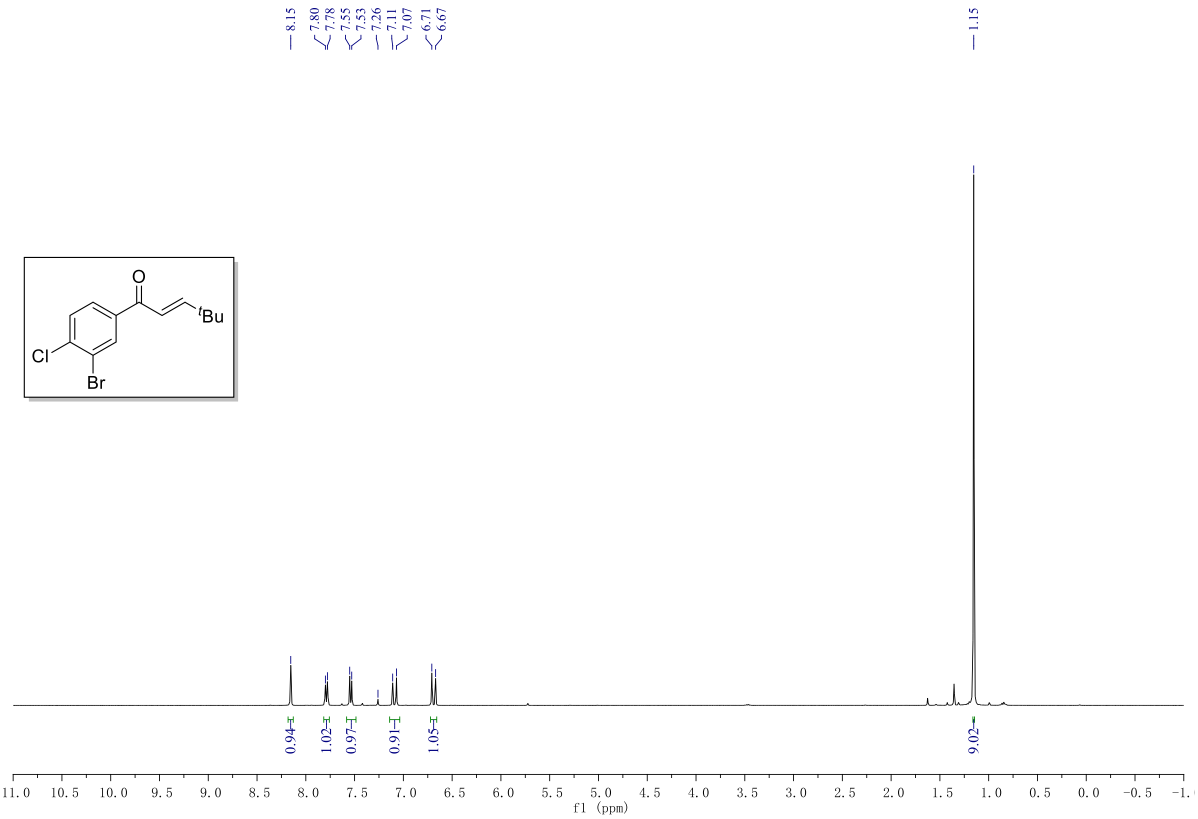

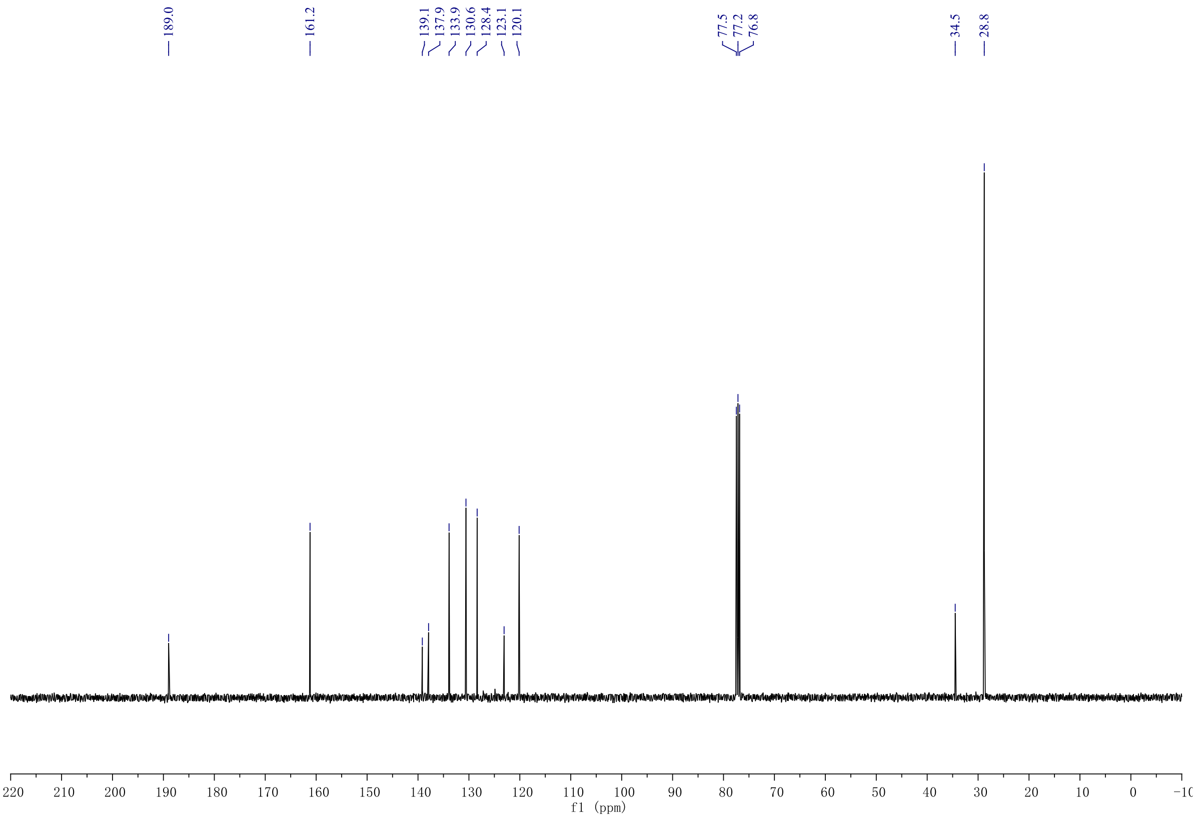

(*E*)-1-{5-Bromo-4-methyl-2-[(*E*)-piperidin-1-yldiazenyl]phenyl}-3-cyclopentyl prop-2-en-1-one (S7)

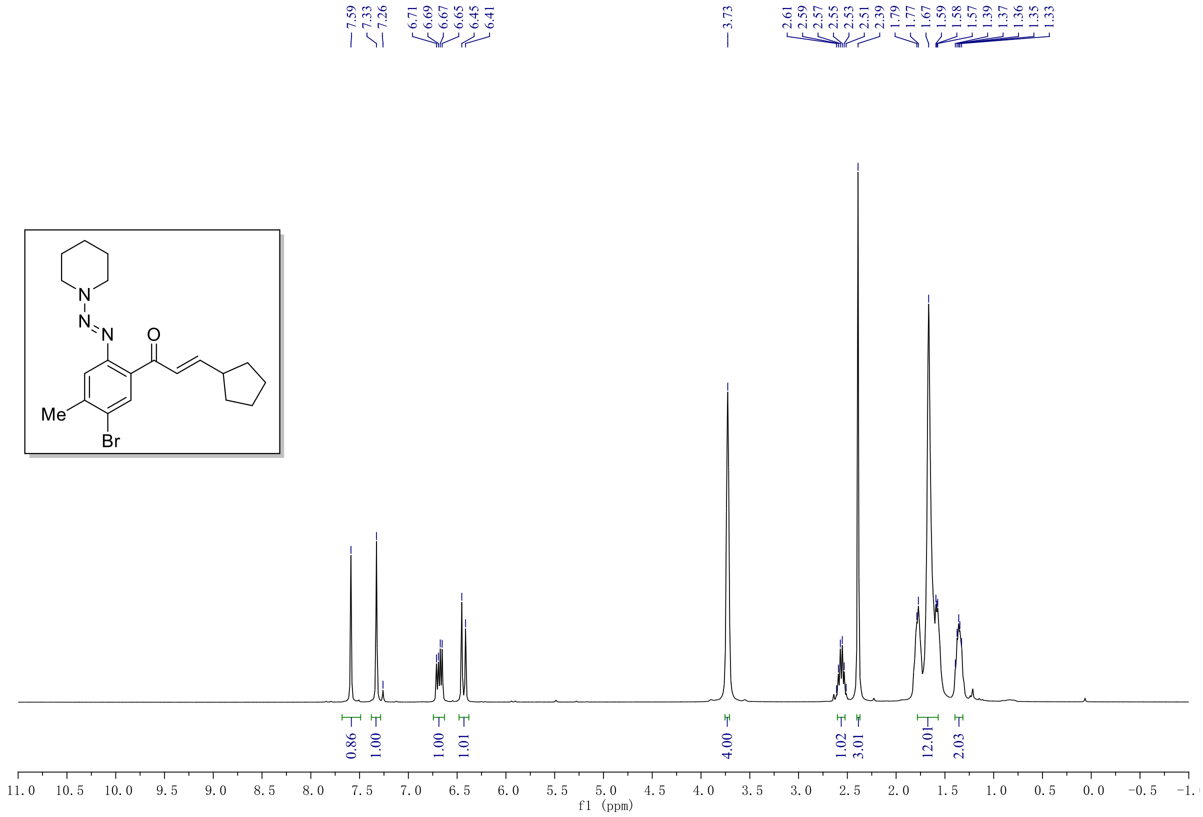

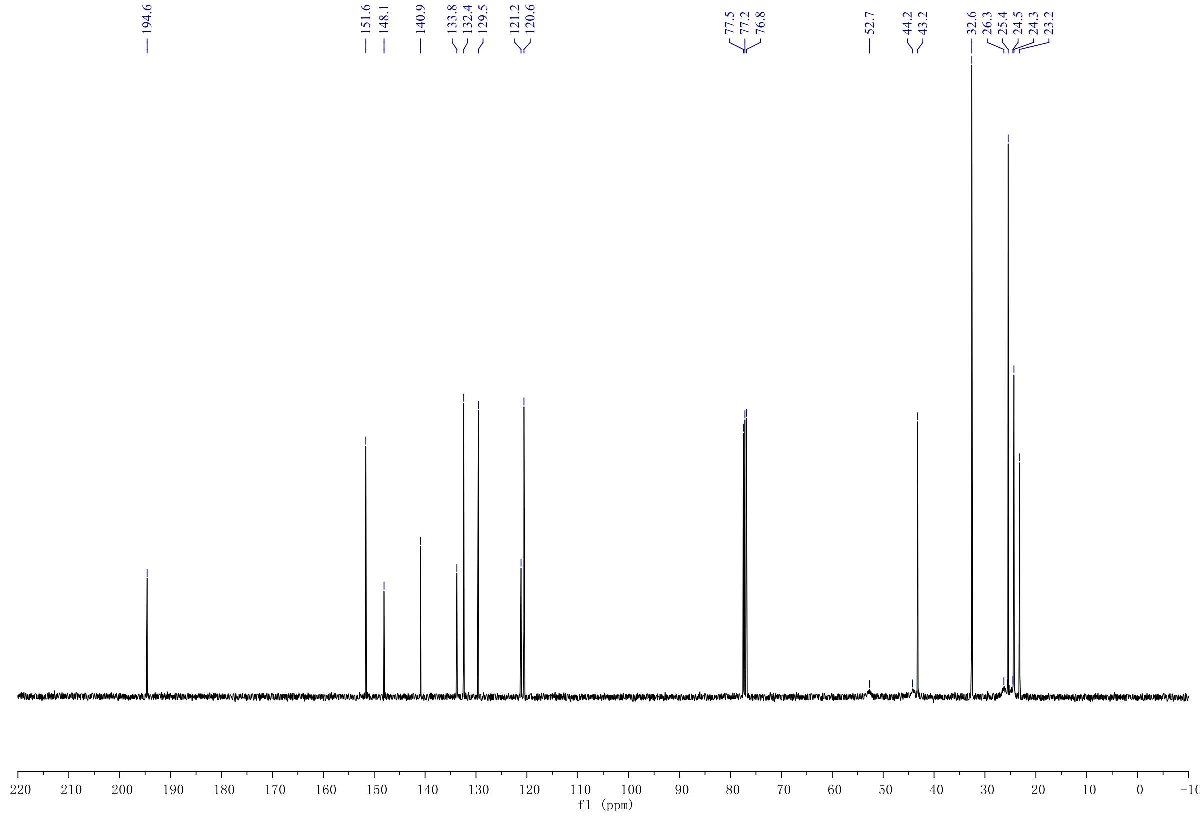

(*E*)-1-(3-Bromo-4-methylphenyl)-3-cyclopentylprop-2-en-1-one (7h)

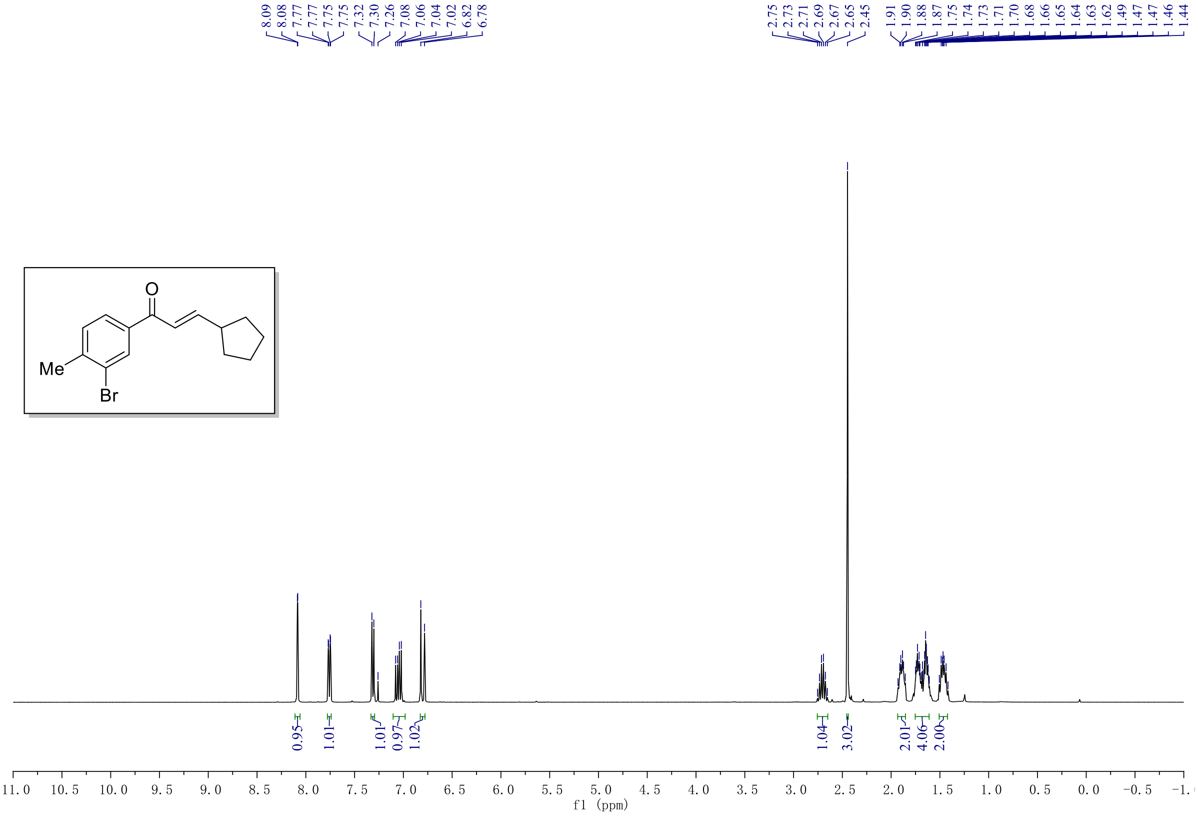

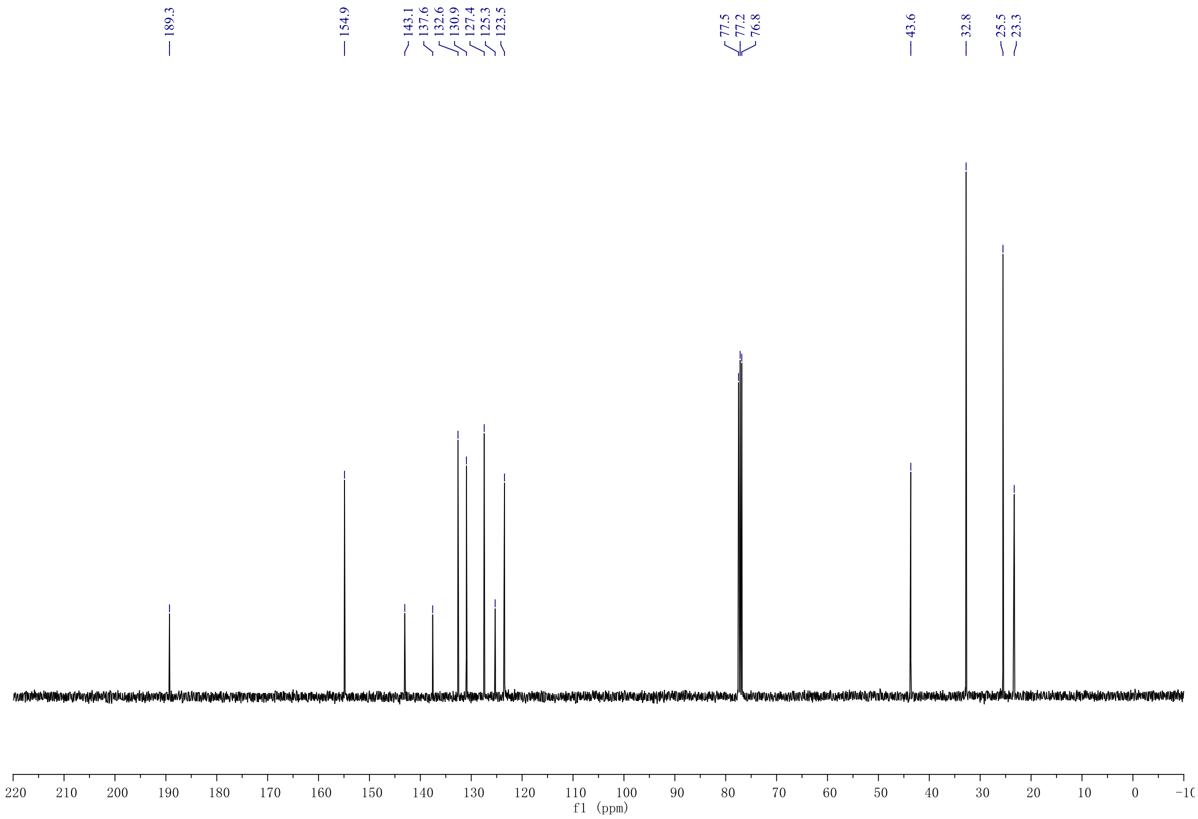

(*E*)-1-(3-Bromo-2-fluorophenyl)-4,4-dimethylpent-2-en-1-one (7i)

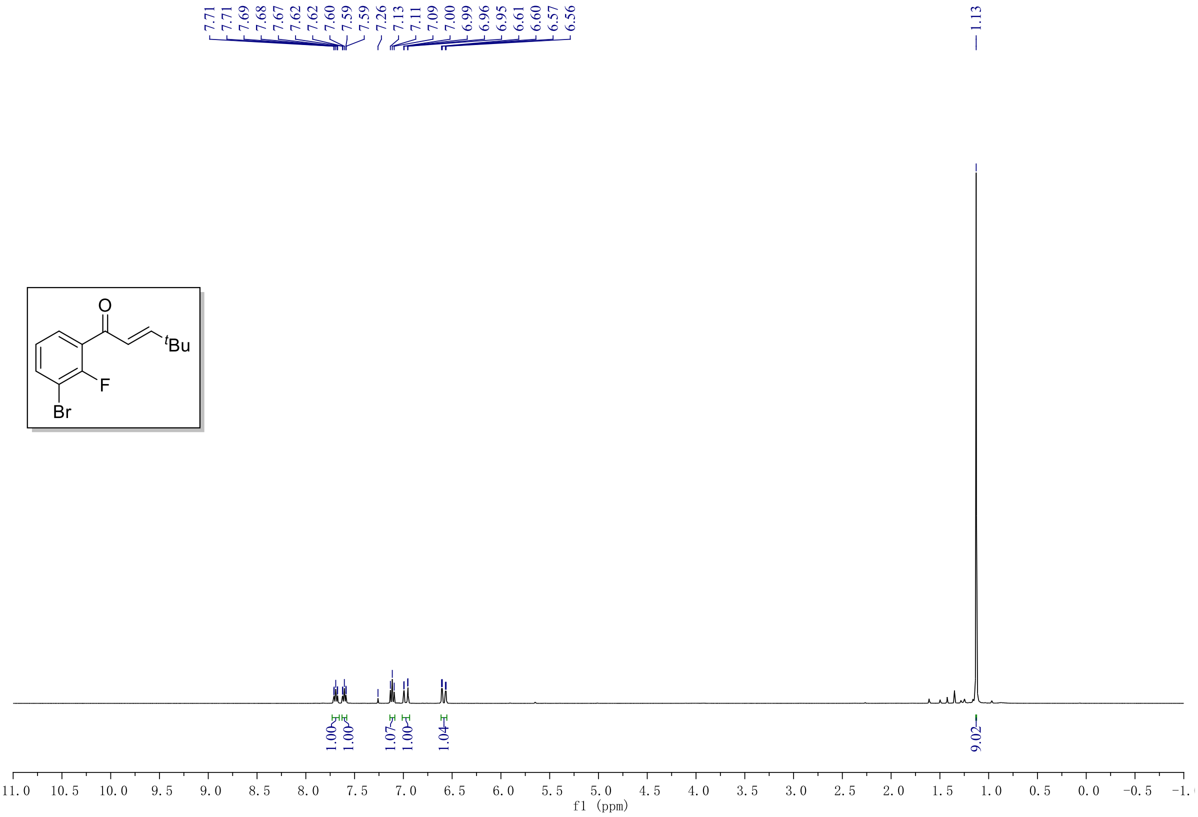

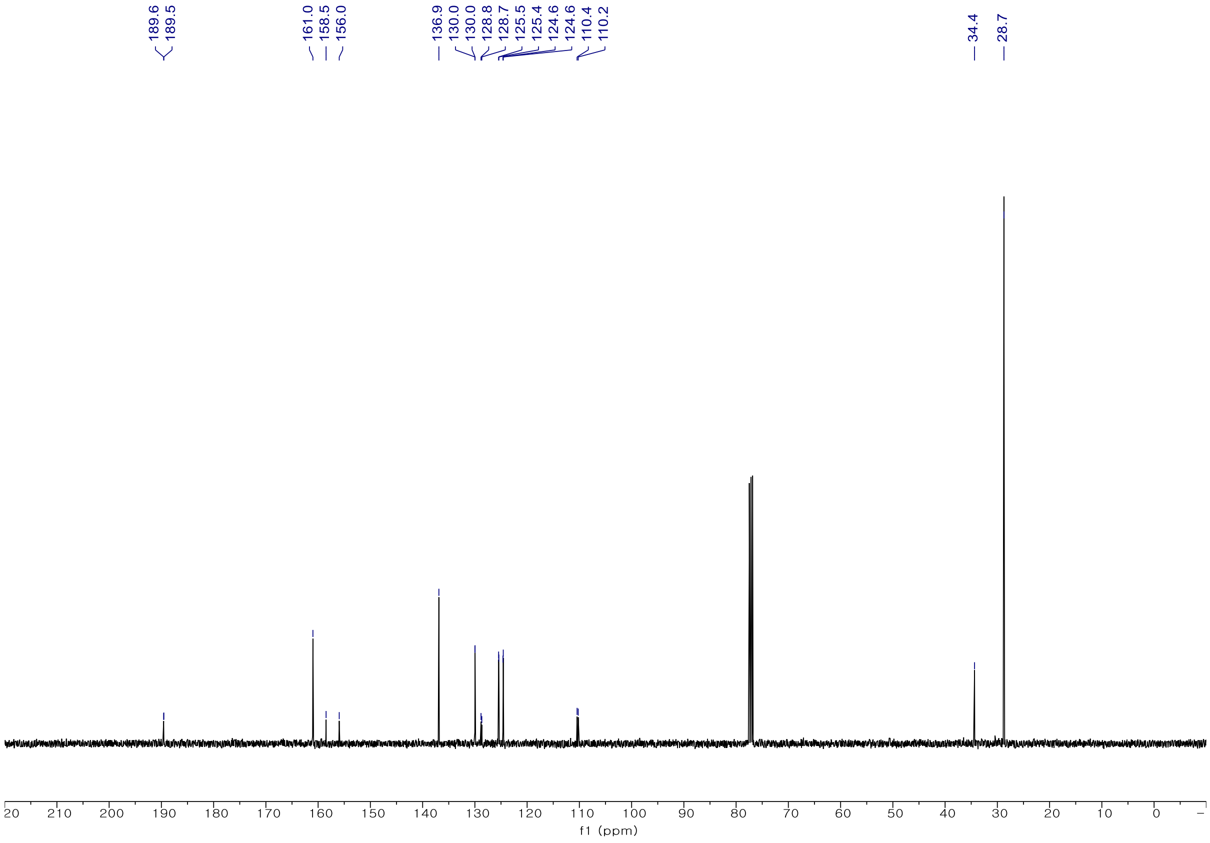

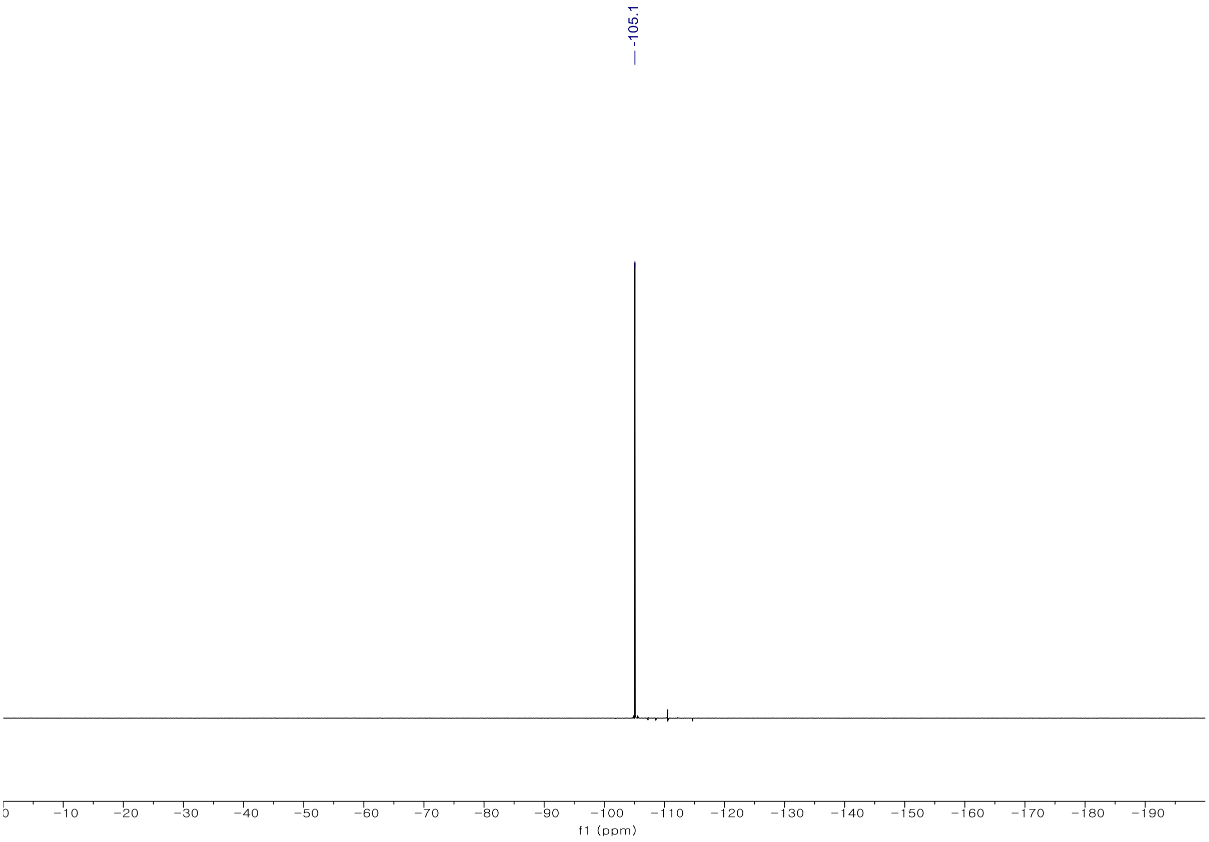

-105.1
0
−10
−20
−30
−40
−50
−60
−70
−80
−90
−100
−110
−120
−130
−140
−150
−160
−170
−180
−190
f1 (ppm)

(*E*)-1-{5-Bromo-3-fluoro-2-[(*E*)-piperidin-1-yldiazenyl]phenyl}-4,4-dimethylpent -2-en-1-one (S8)

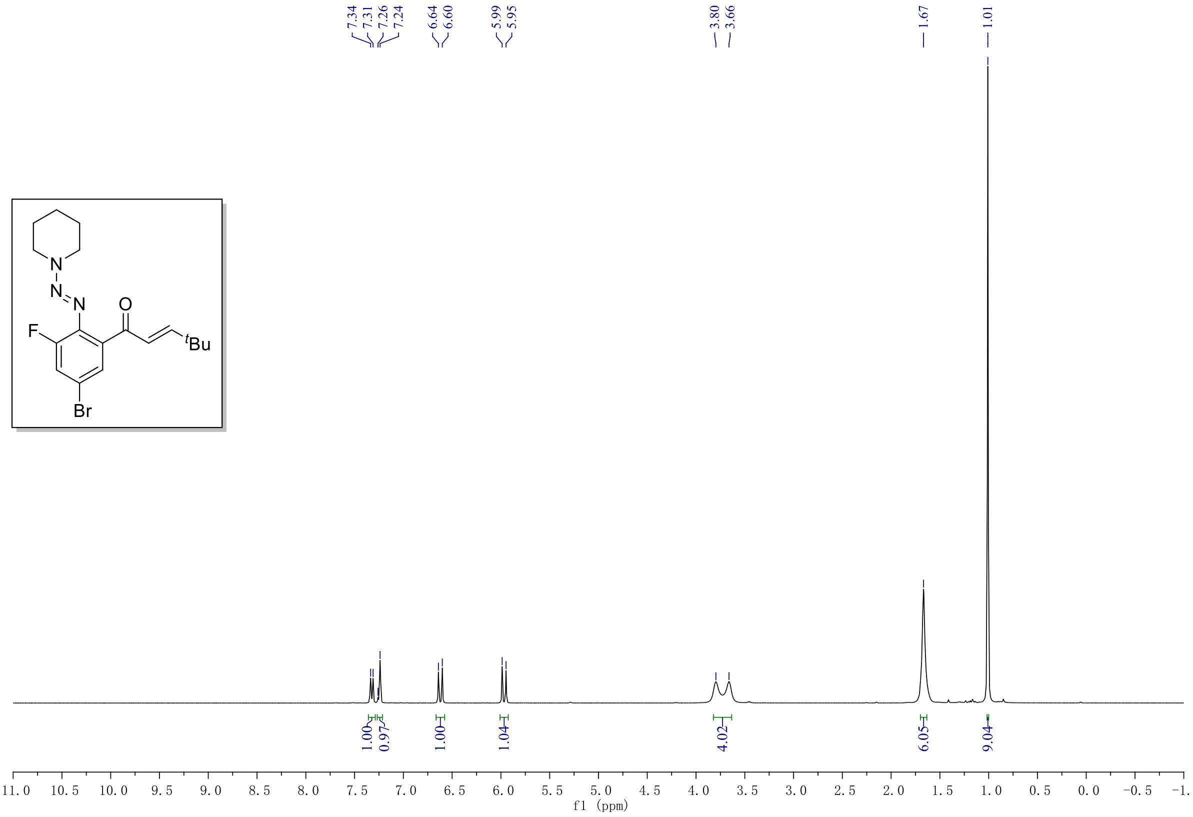

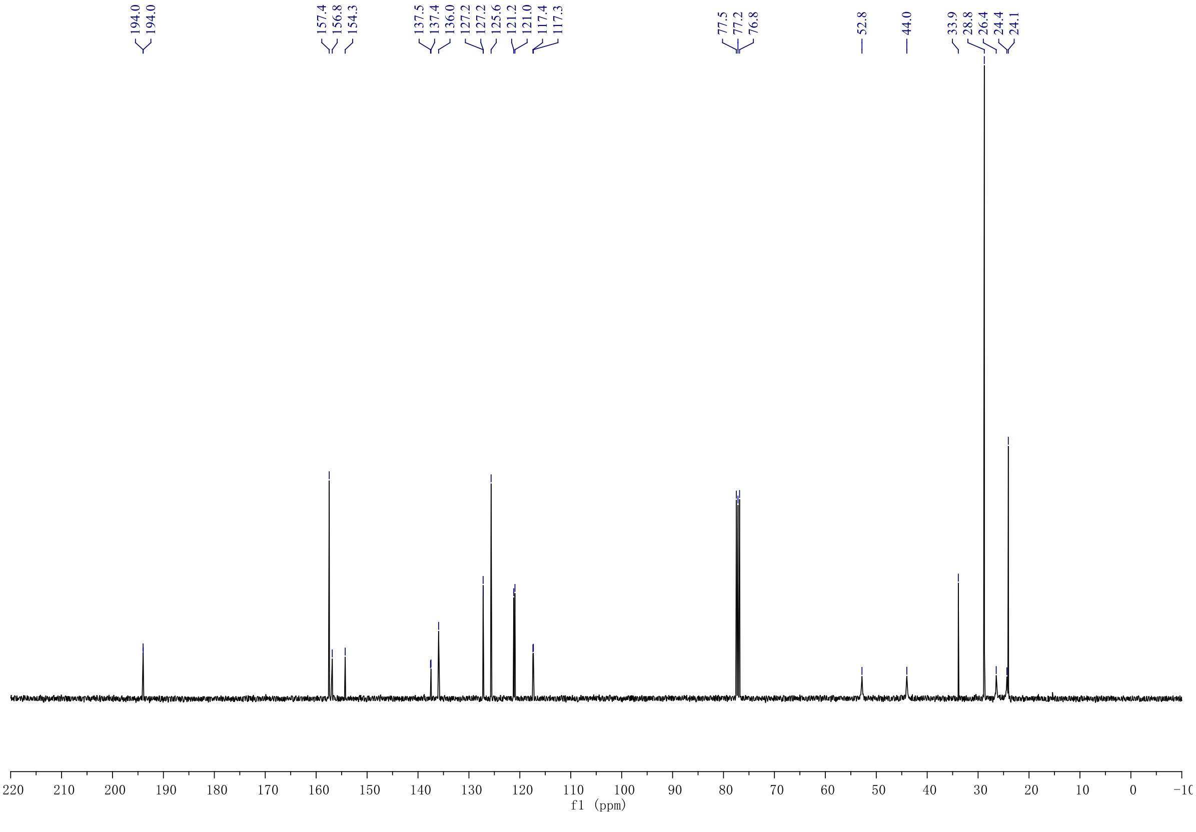

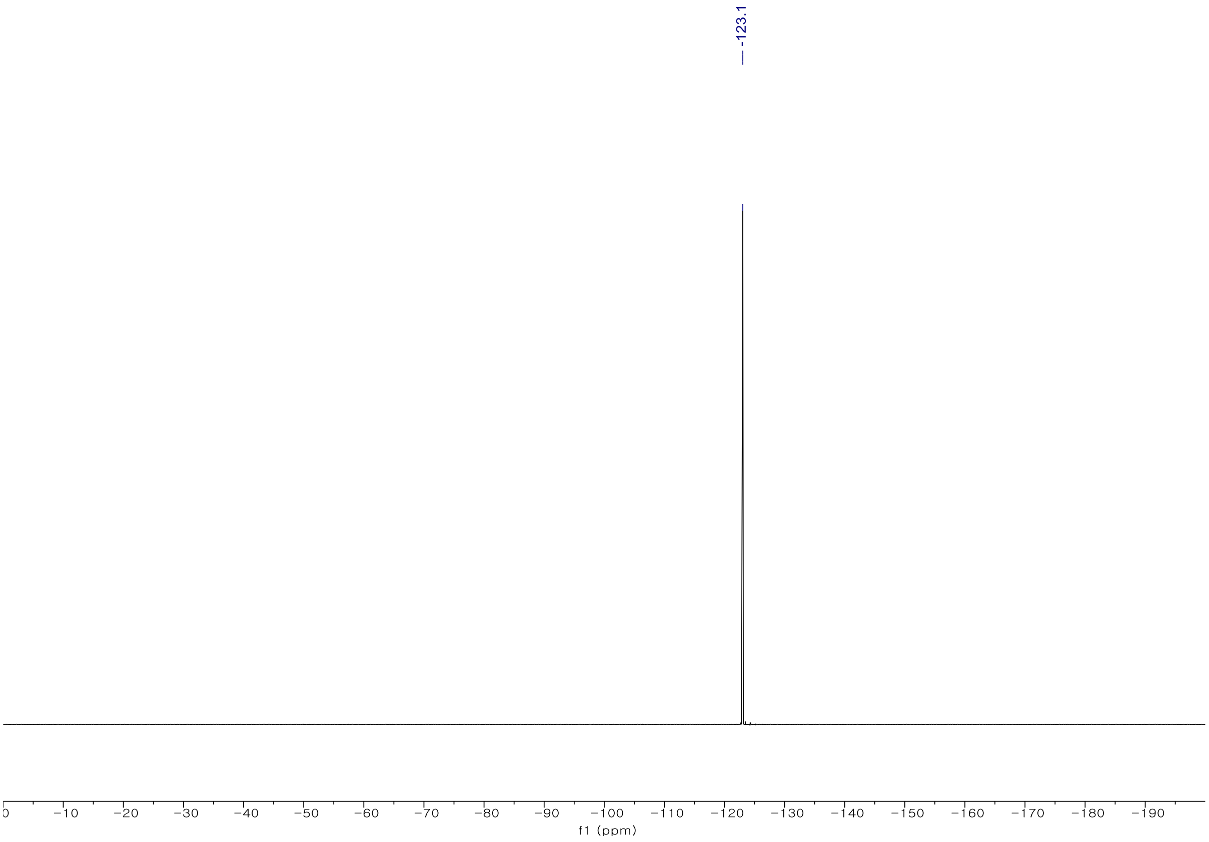
-123.1
0
-10
-20
-30
-40
-50
-60
-70
-80
-90
-100
-110
-120
-130
-140
-150
-160
-170
-180
-190
f1 (ppm)

(*E*)-1-(3-Bromo-5-fluorophenyl)-4,4-dimethylpent-2-en-1-one, (7j)

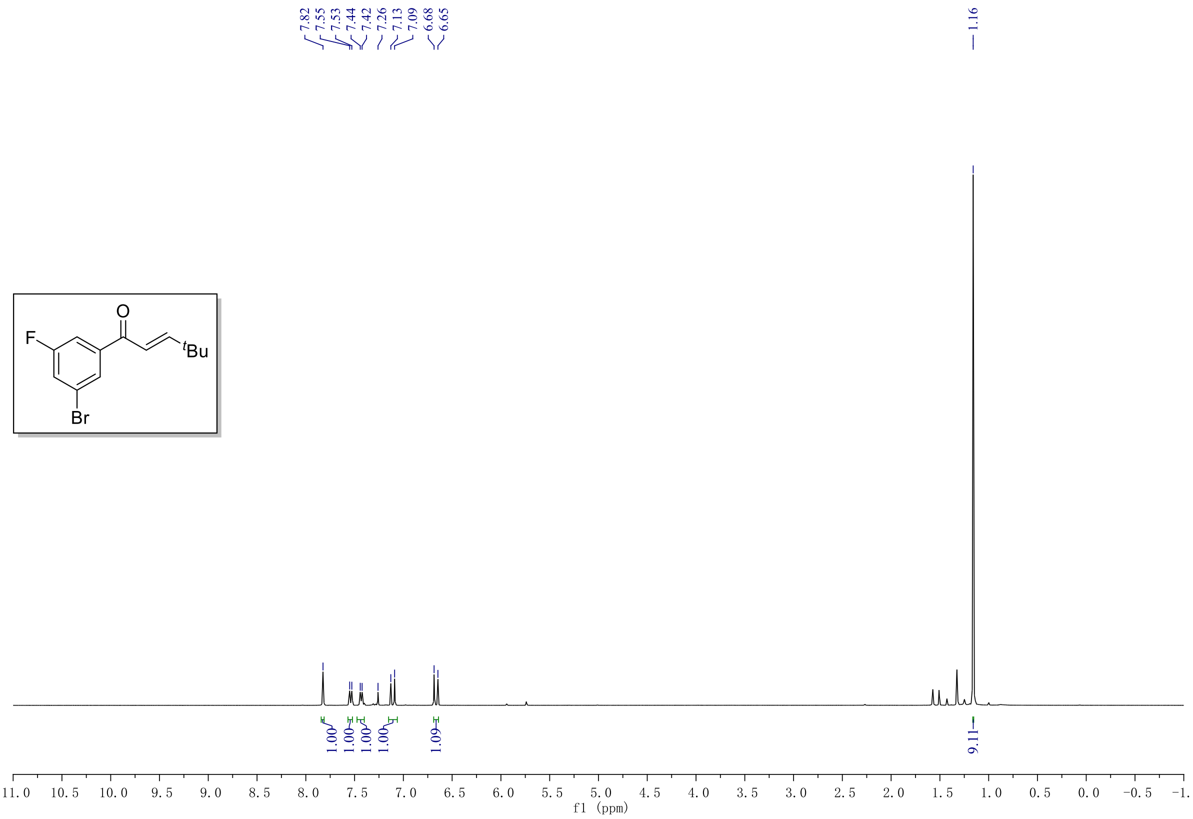

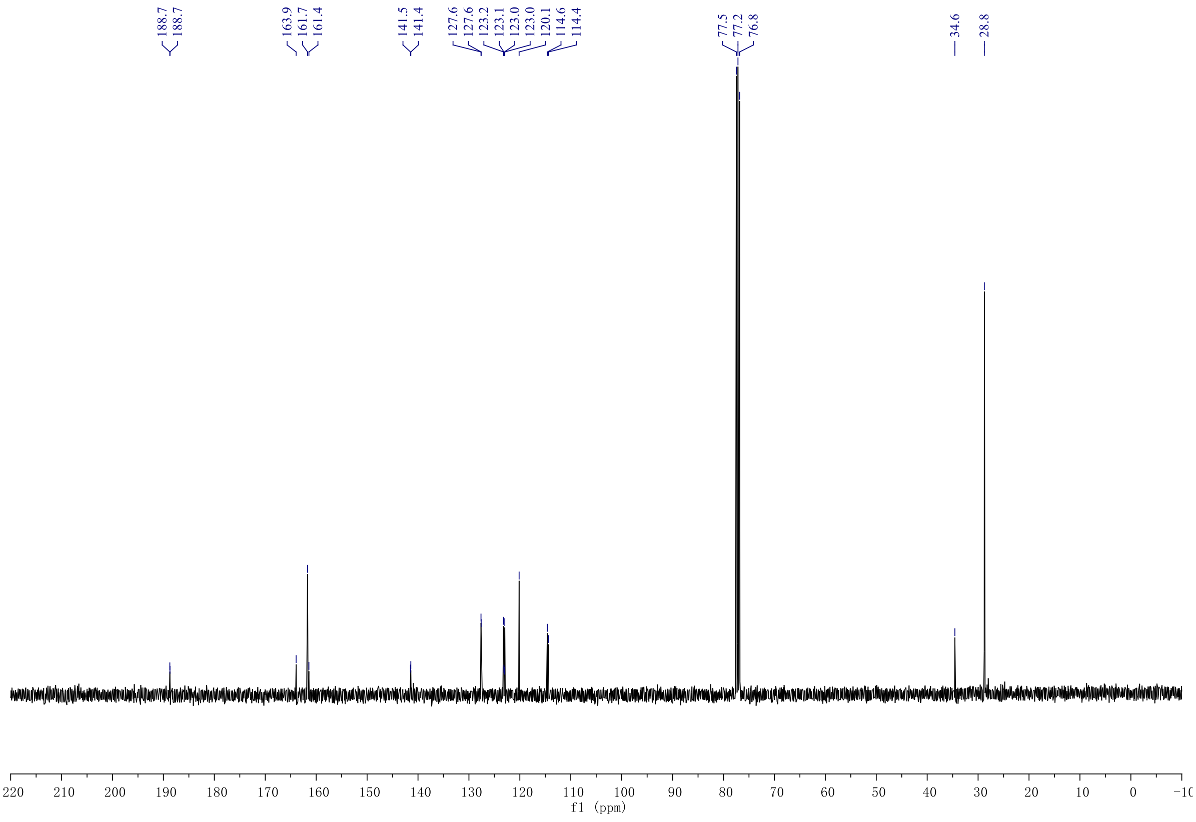

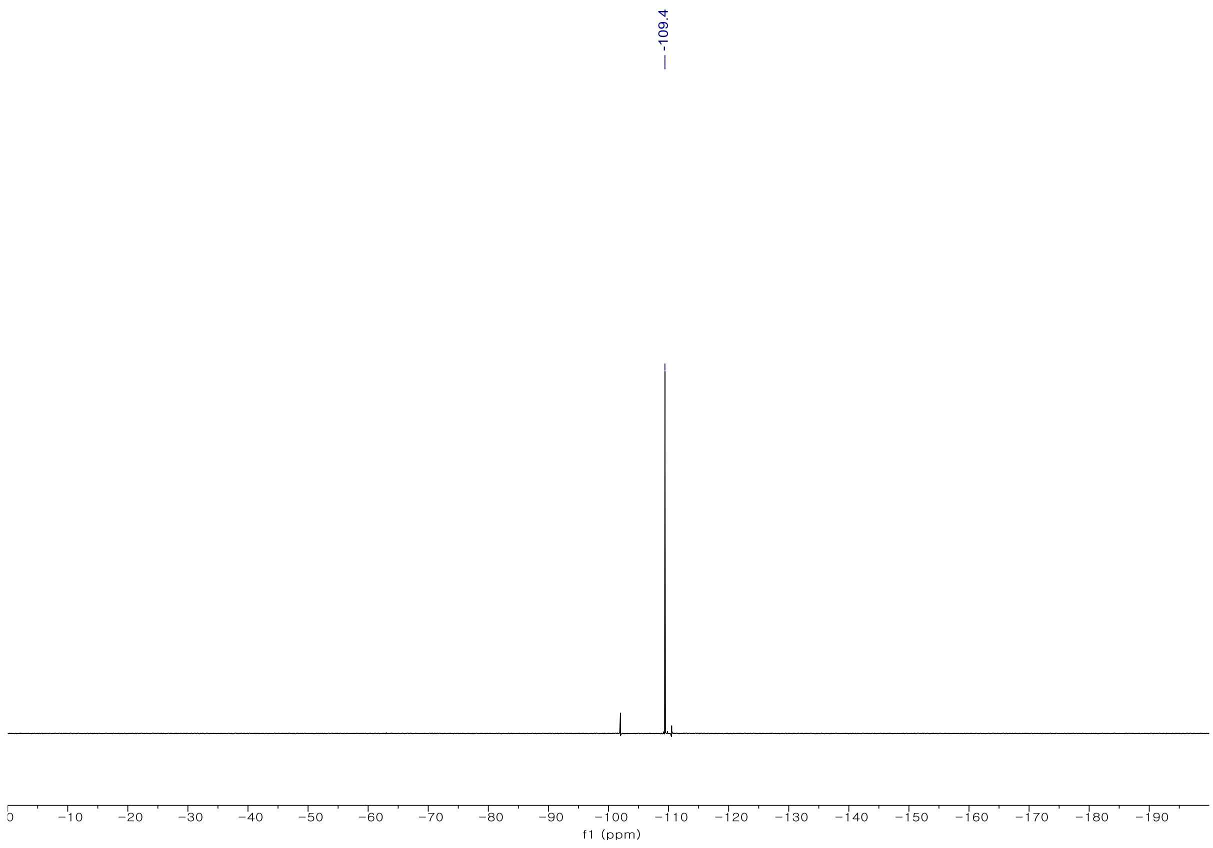

-109.4
0
-10
-20
-30
-40
-50
-60
-70
-80
-90
-100
-110
-120
-130
-140
-150
-160
-170
-180
-190
f1 (ppm)

(*E*)-1-{5-Chloro-4-fluoro-2-[(*E*)-piperidin-1-yldiazenyl]phenyl}-4,4-dimethylpent -2-en-1-one (S9)

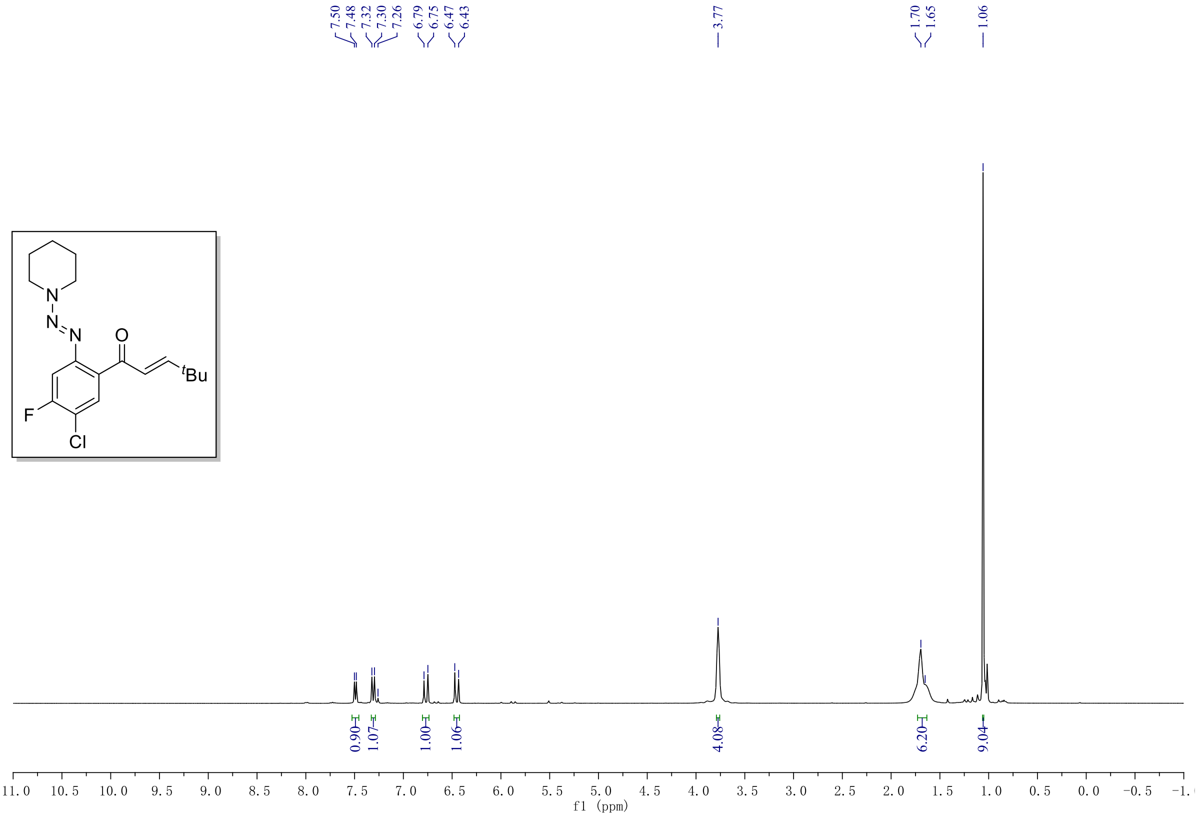

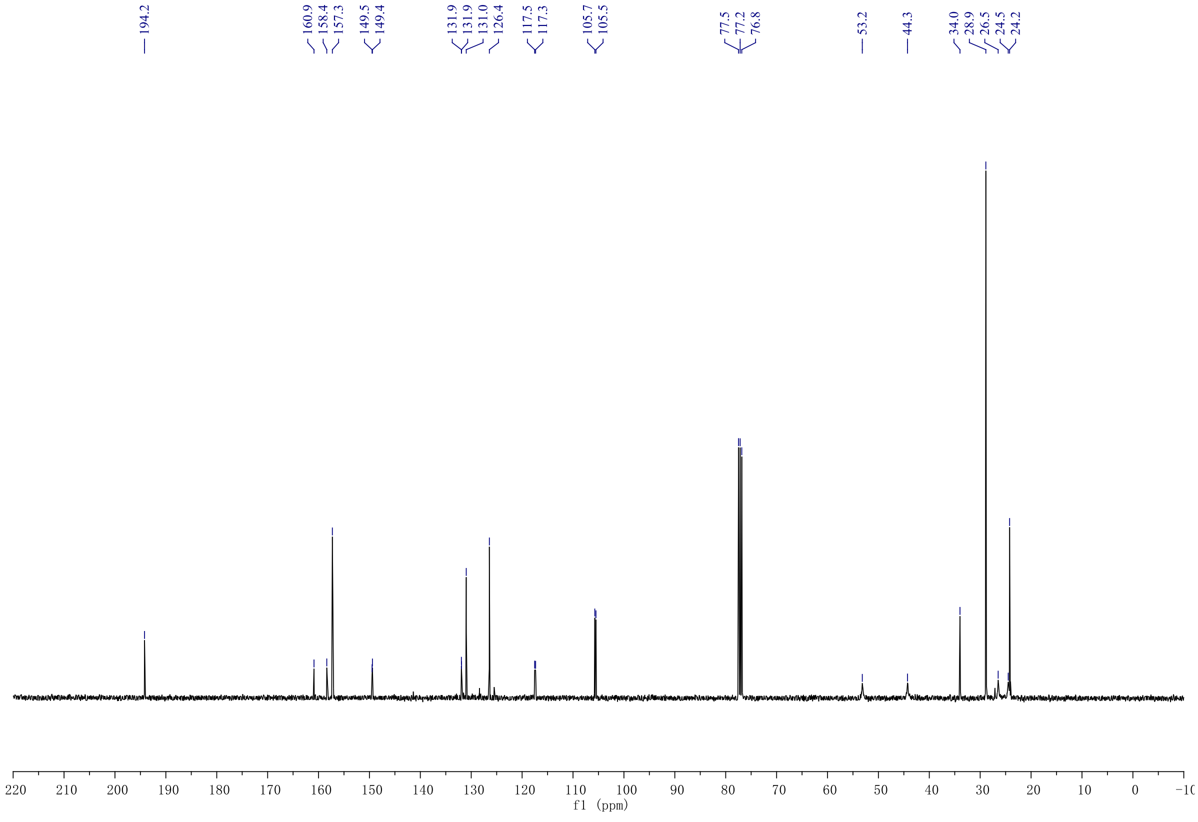

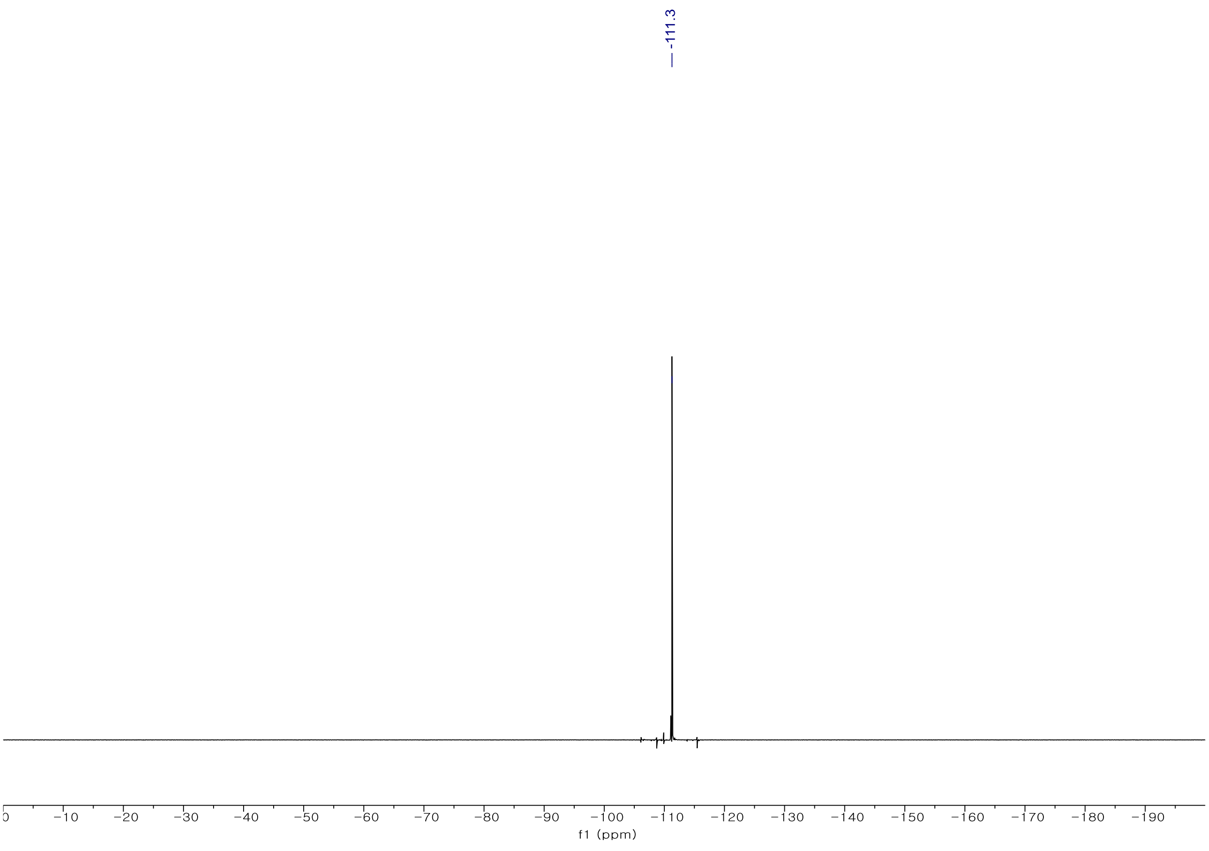

-111.3
0
-10
-20
-30
-40
-50
-60
-70
-80
-90
-100
-110
-120
-130
-140
-150
-160
-170
-180
-190
f1 (ppm)

(*E*)-1-(3-Chloro-4-fluorophenyl)-4,4-dimethylpent-2-en-1-one (7k)

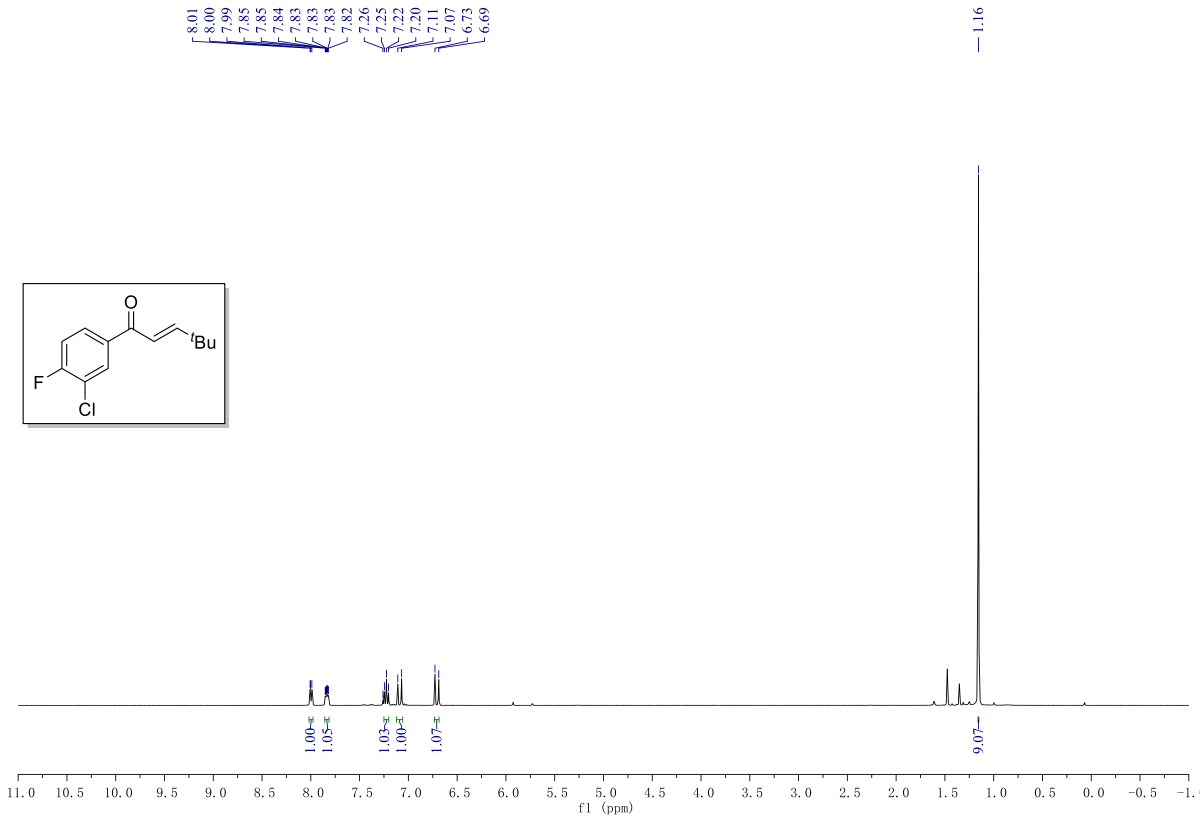

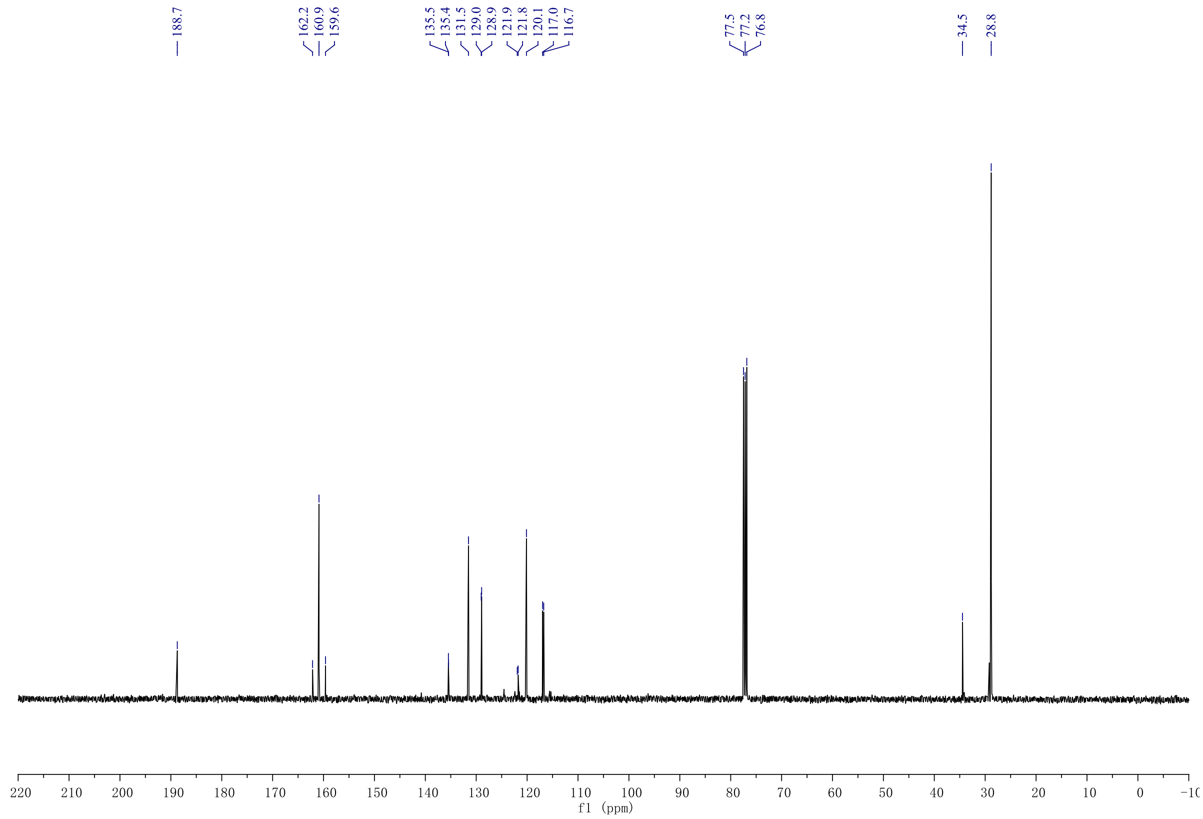

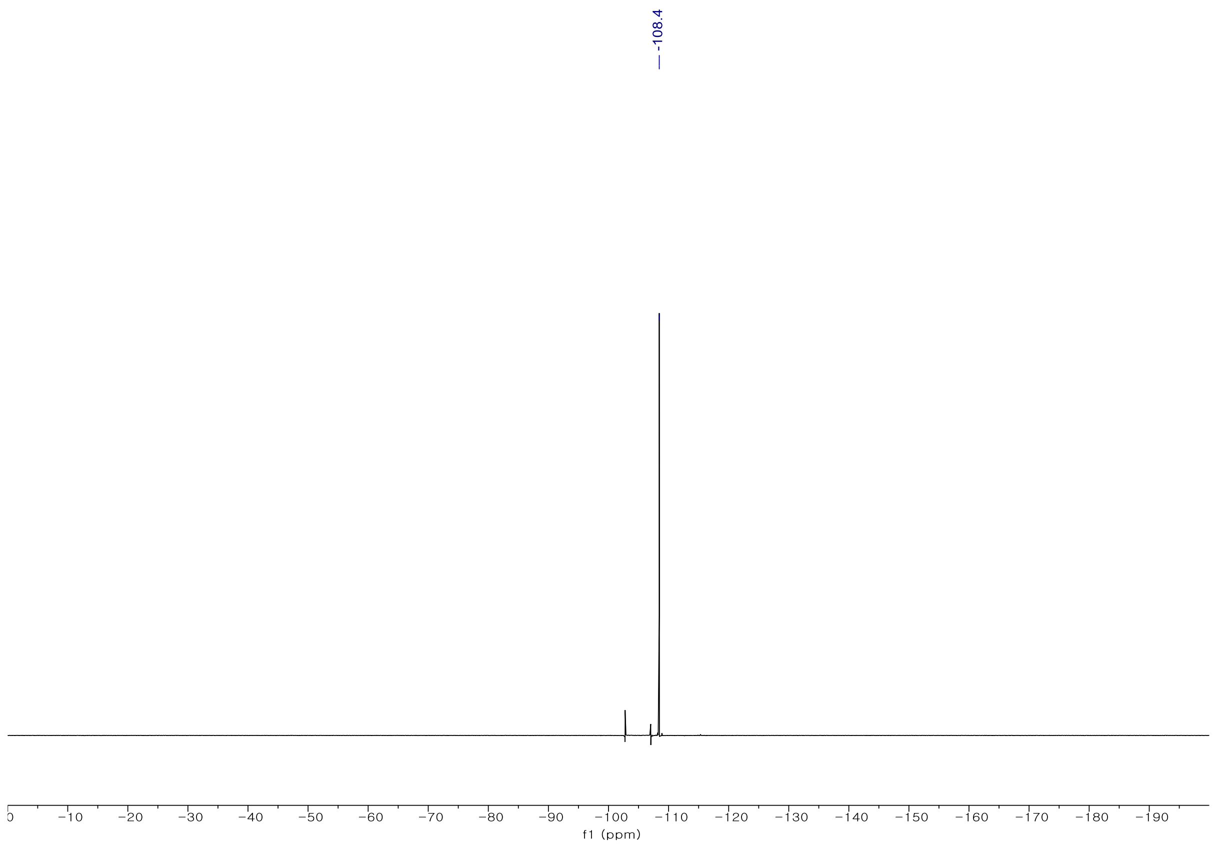
-108.4
0
−10
−20
−30
−40
−50
−60
−70
−80
−90
−100
−110
−120
−130
−140
−150
−160
−170
−180
−190
f1 (ppm)

(*E*)-1-[4'-Methoxy-(1,1'-biphenyl)-2-yl]-3-phenylprop-2-en-1-one (8)

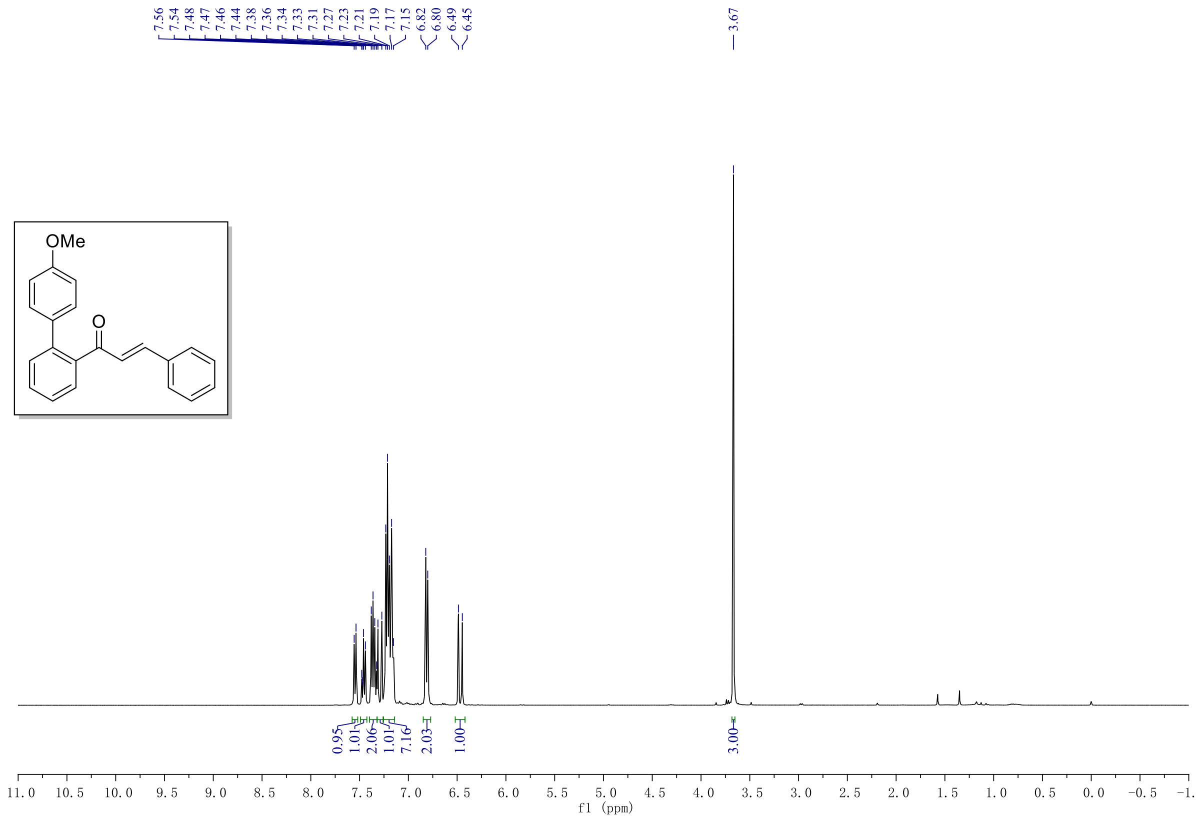

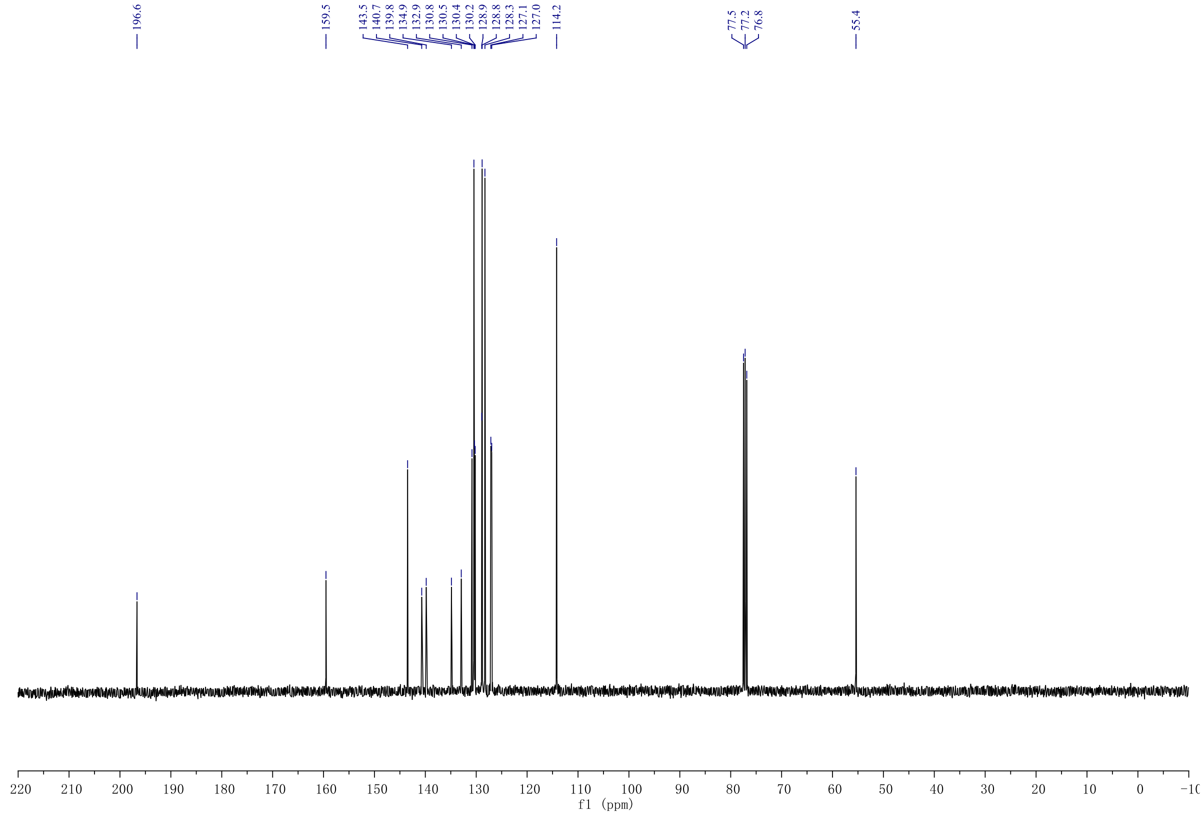

(*E*)-3-Phenyl-1-{2-[(trimethylsilyl)ethynyl]phenyl}prop-2-en-1-one (10)

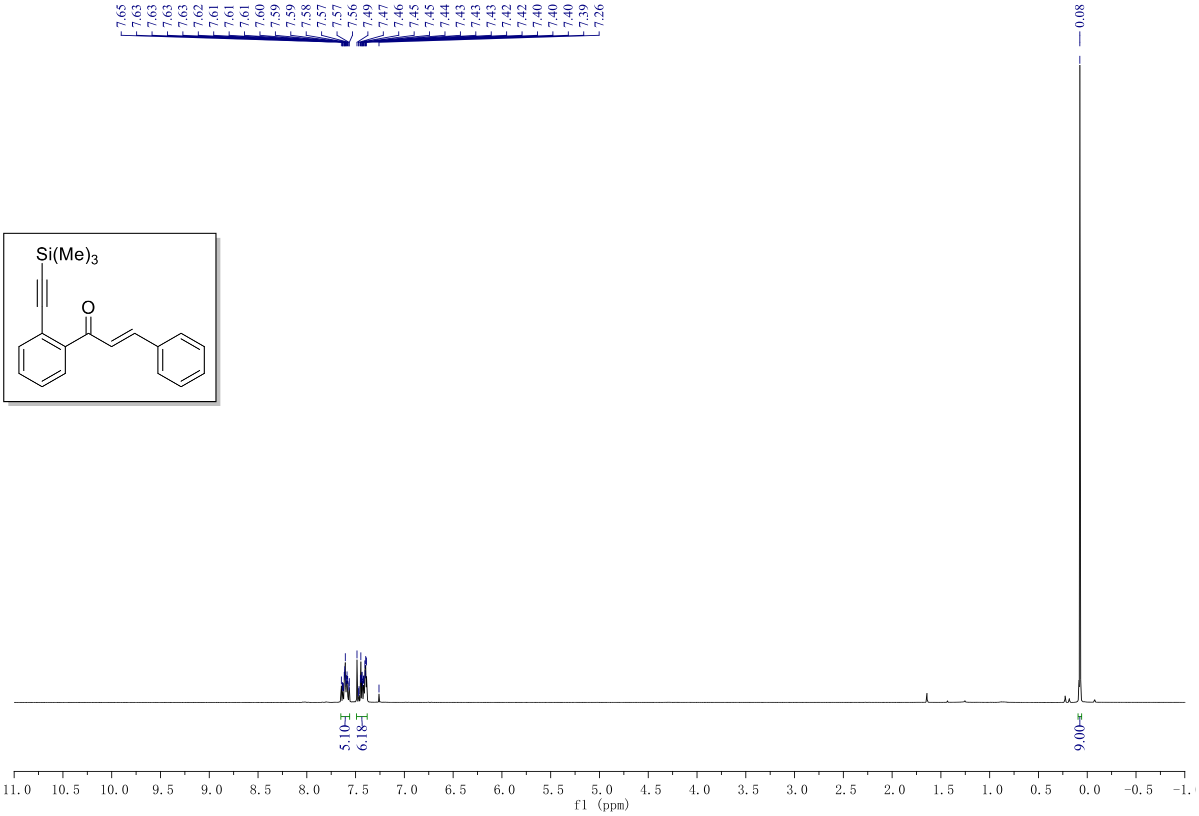

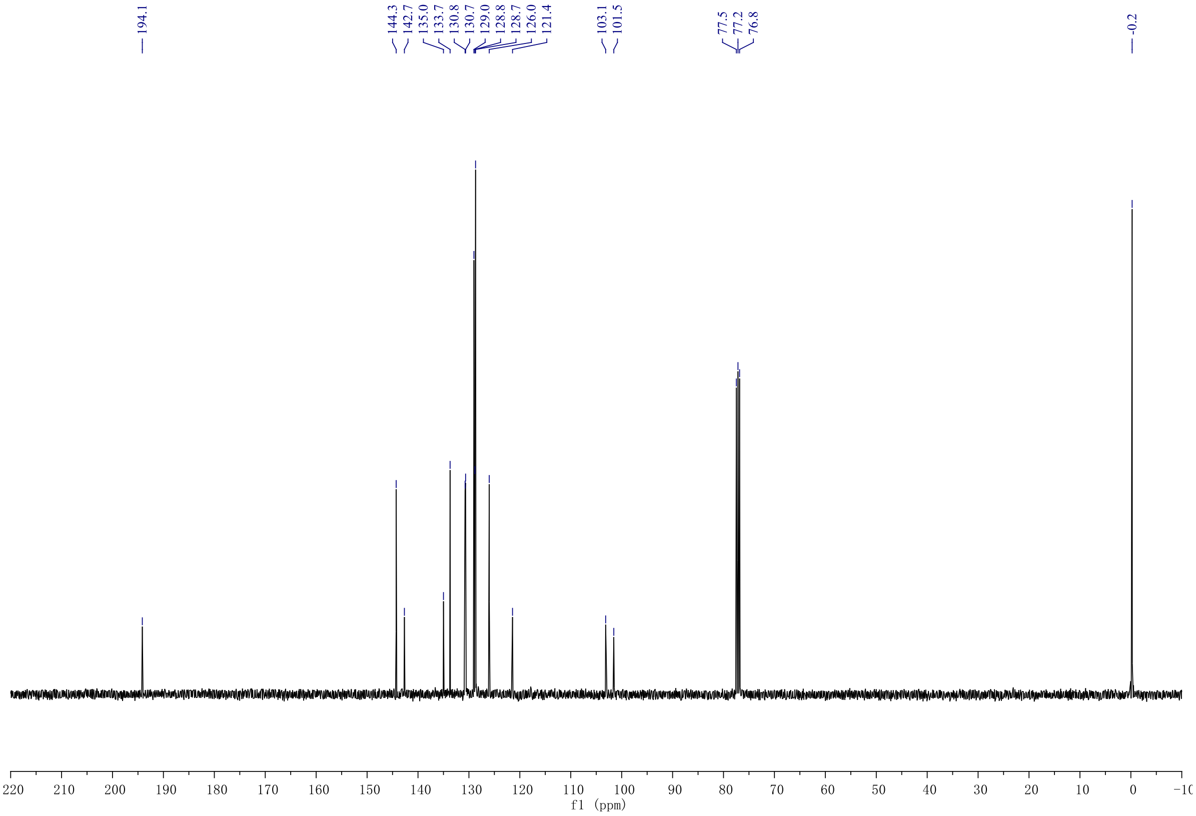

(*E*)-1-(2-Azidophenyl)-3-phenylprop-2-en-1-one (11)

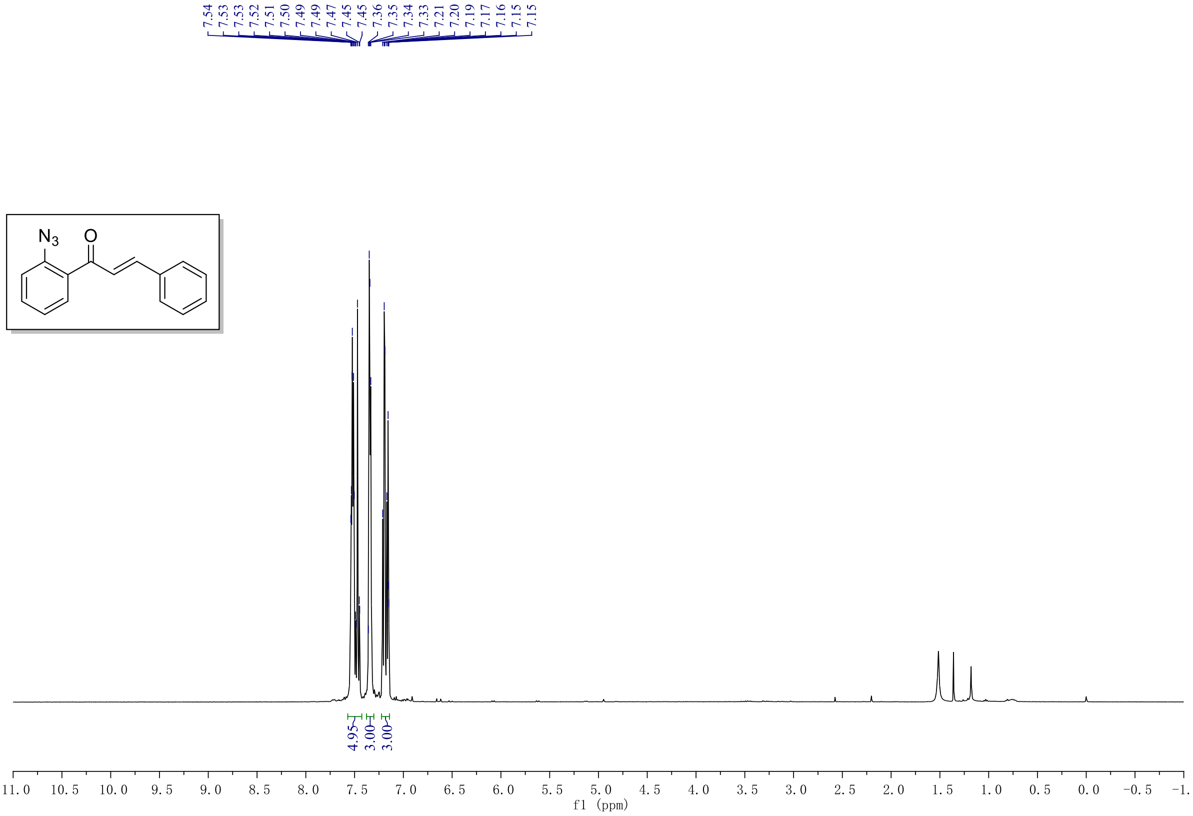

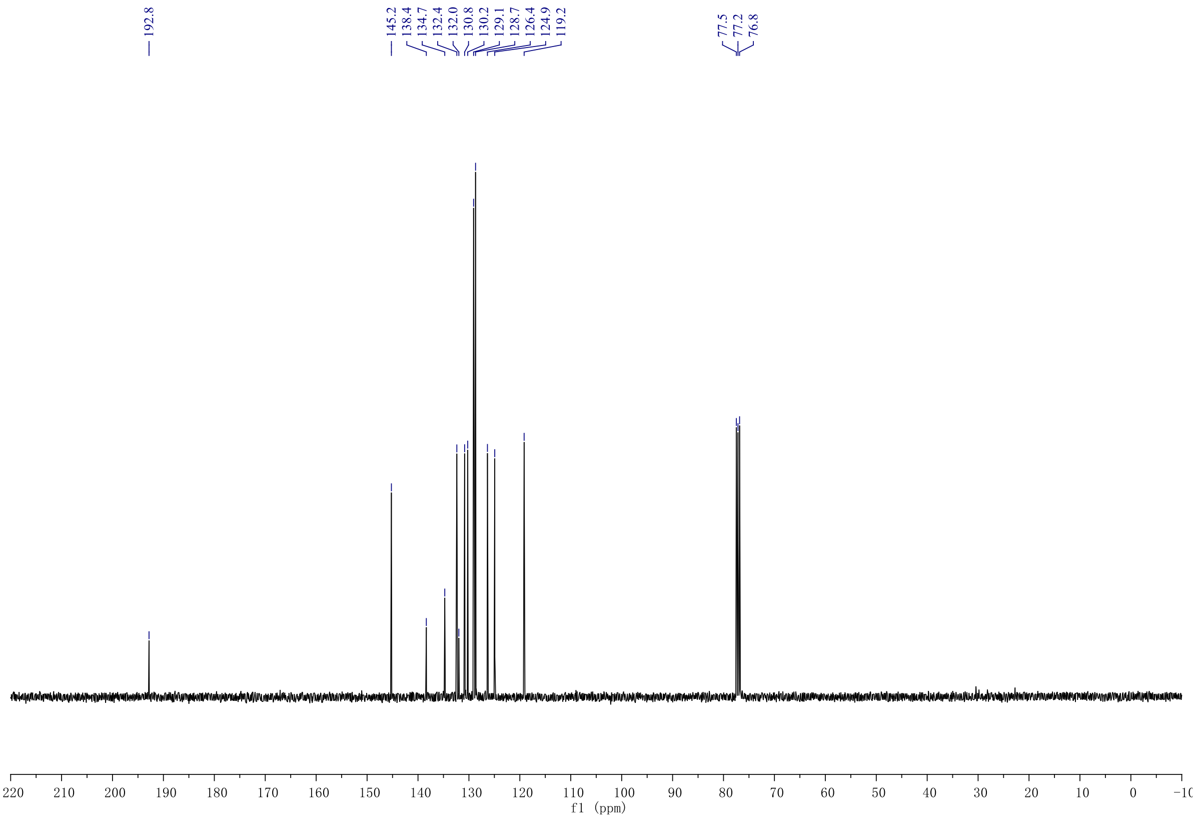

(*E*)-1-(2-Deuteriophenyl)-3-phenylprop-2-en-1-one (12)

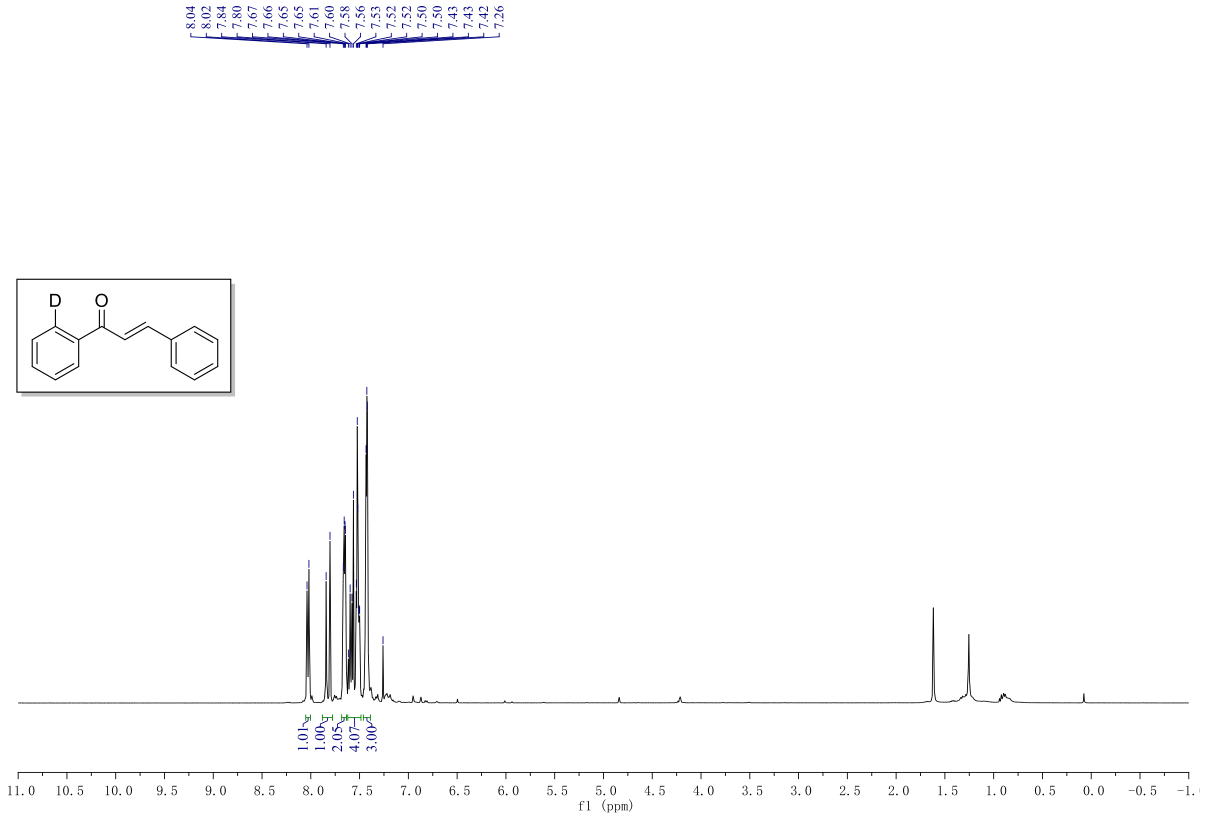

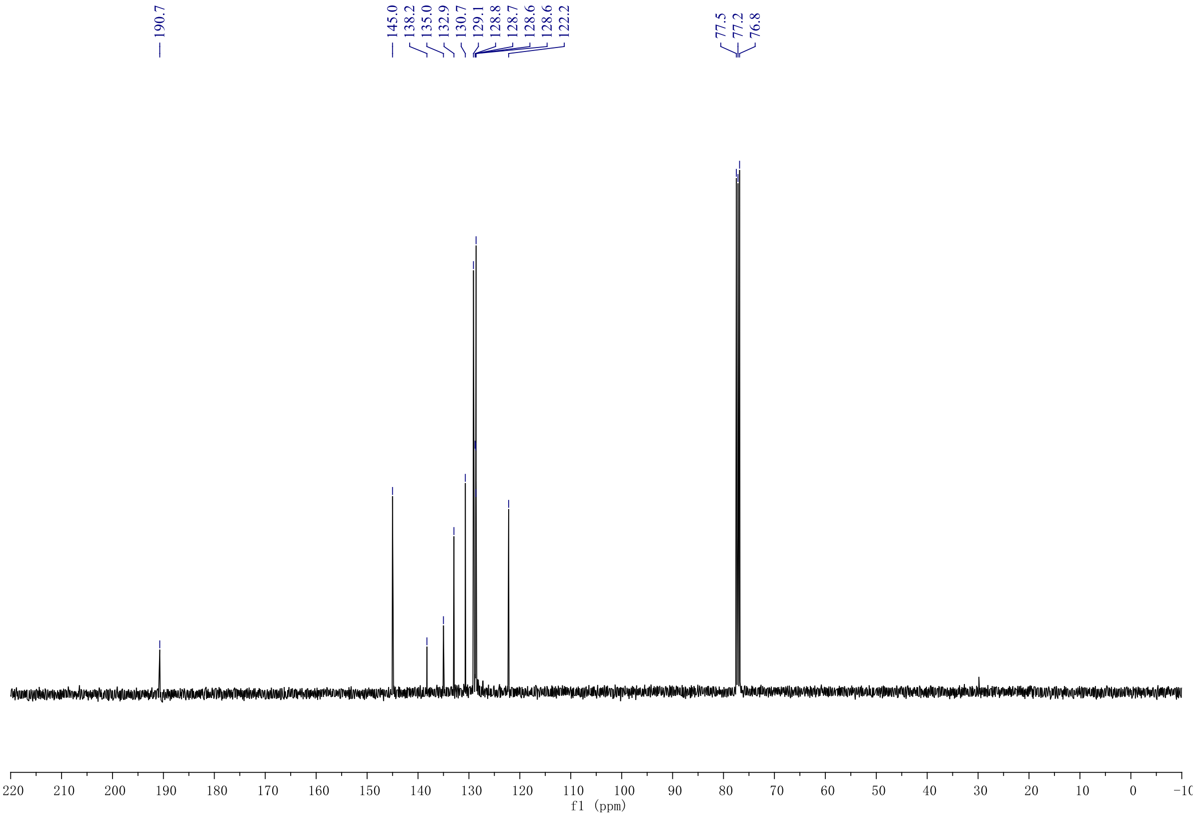

(*E*)-1-(6-Fluoro-4'-methyl-[1,1'-biphenyl]-2-yl)-4,4-dimethylpent-2-en-1-one (13a)

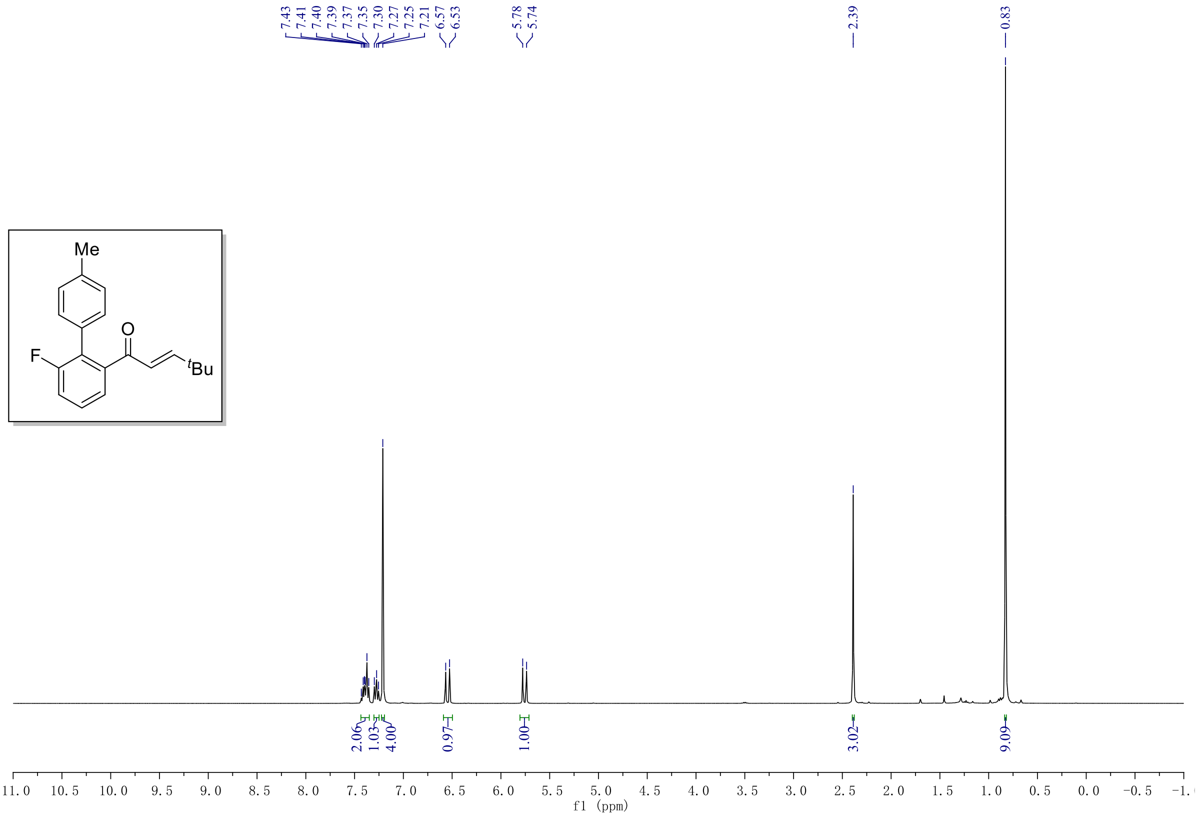

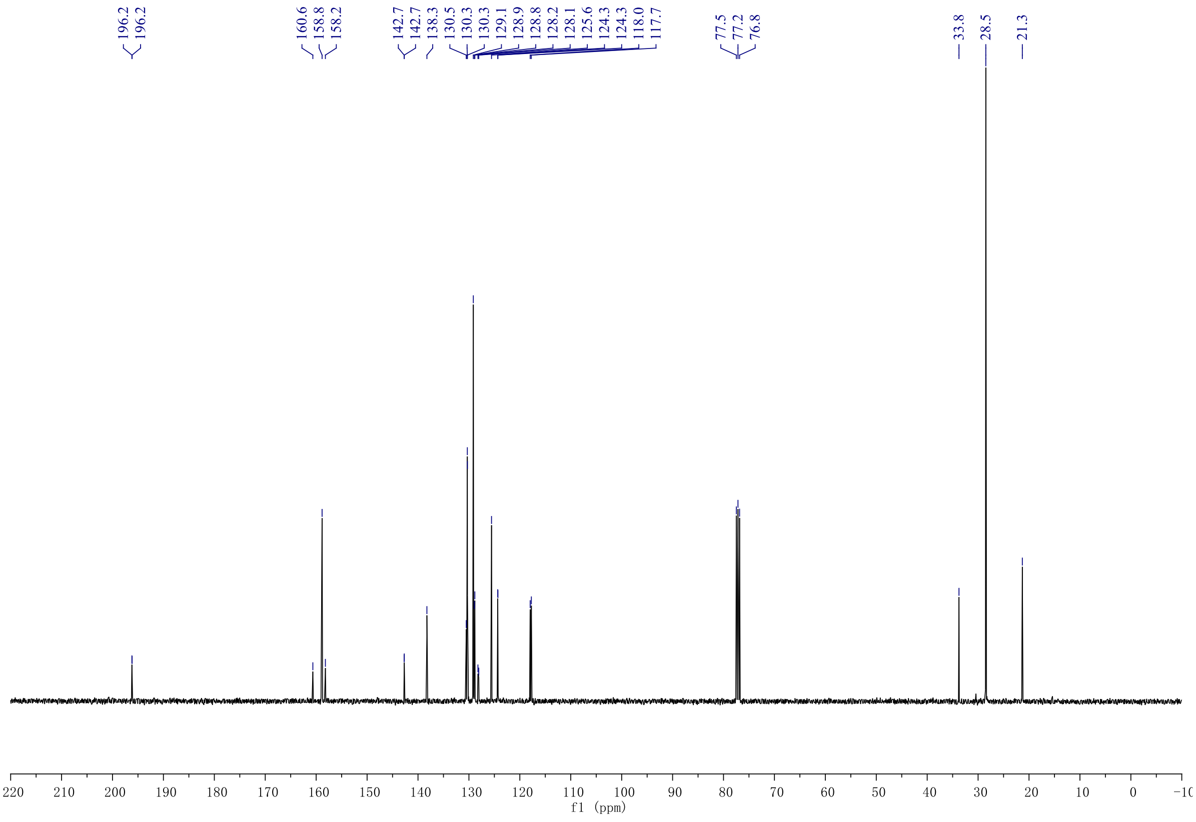

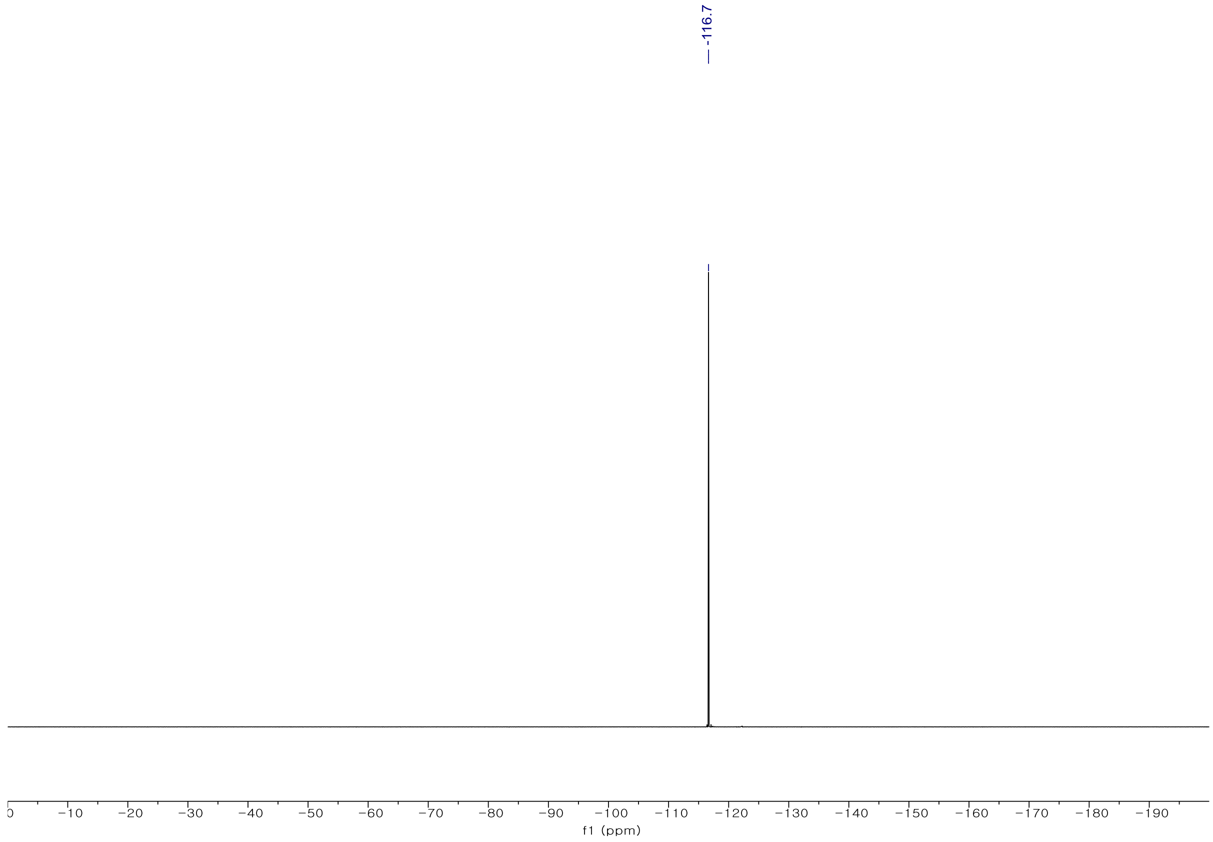
-116.7
0
-10
-20
-30
-40
-50
-60
-70
-80
-90
-100
-110
-120
-130
-140
-150
-160
-170
-180
-190
f1 (ppm)

(*E*)-1-(5-Chloro-2'-methyl-[1,1'-biphenyl]-2-yl)-4,4-dimethylpent-2-en-1-one (13b)

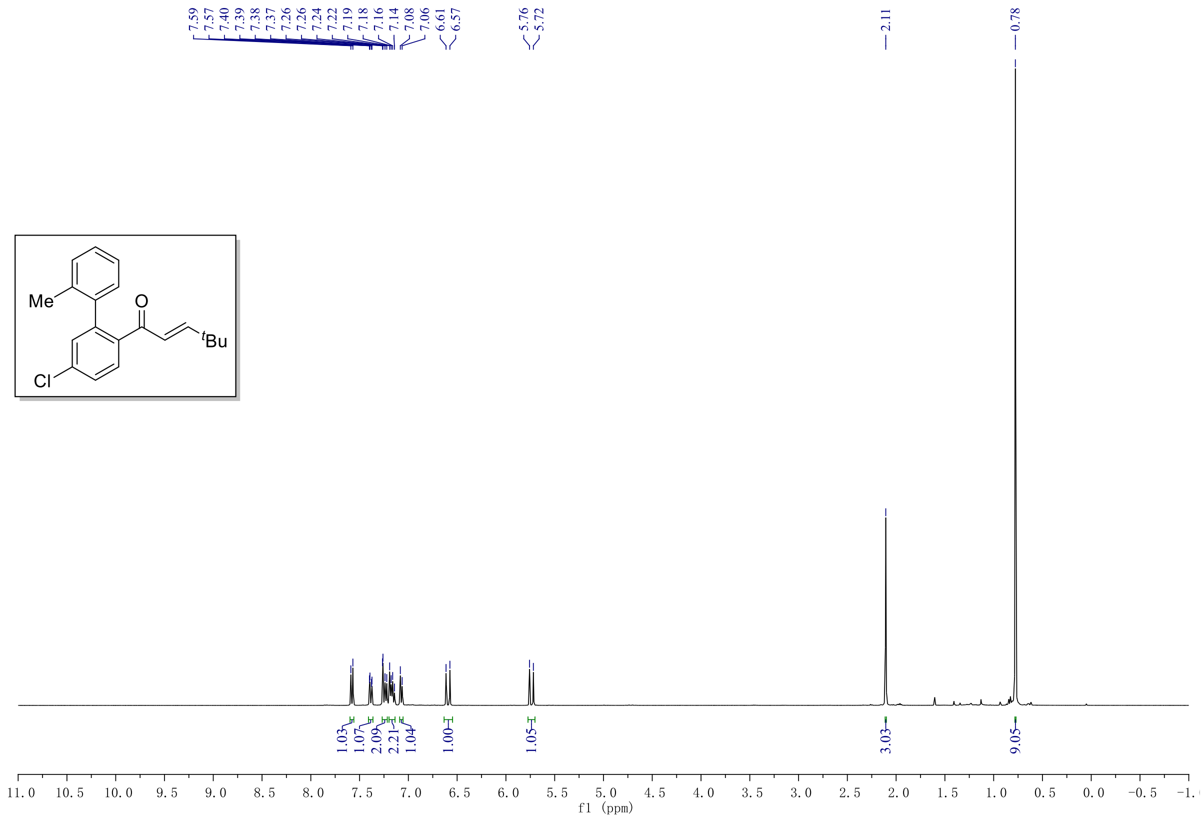

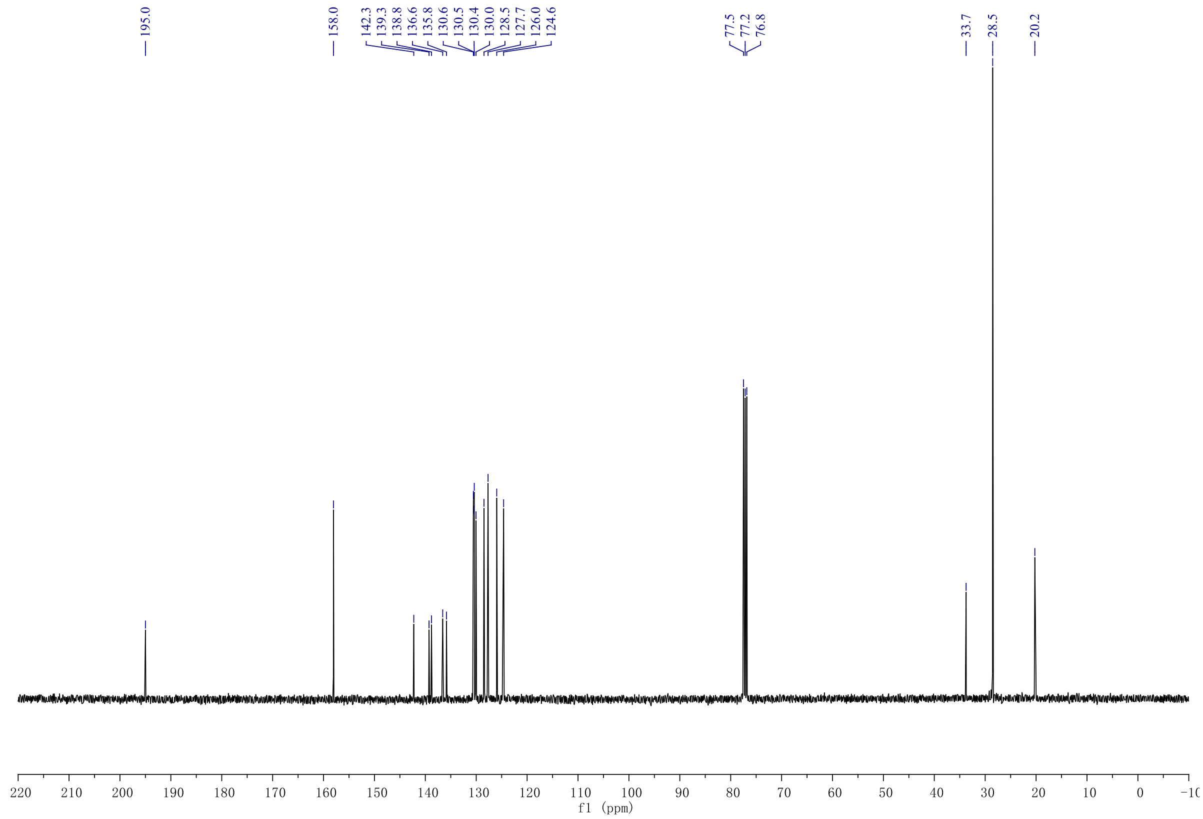

(*E*)-1-(3'-Chloro-3-fluoro-4'-methoxy-[1,1'-biphenyl]-2-yl)-4,4-dimethylpent-2-en-1-one (13c)

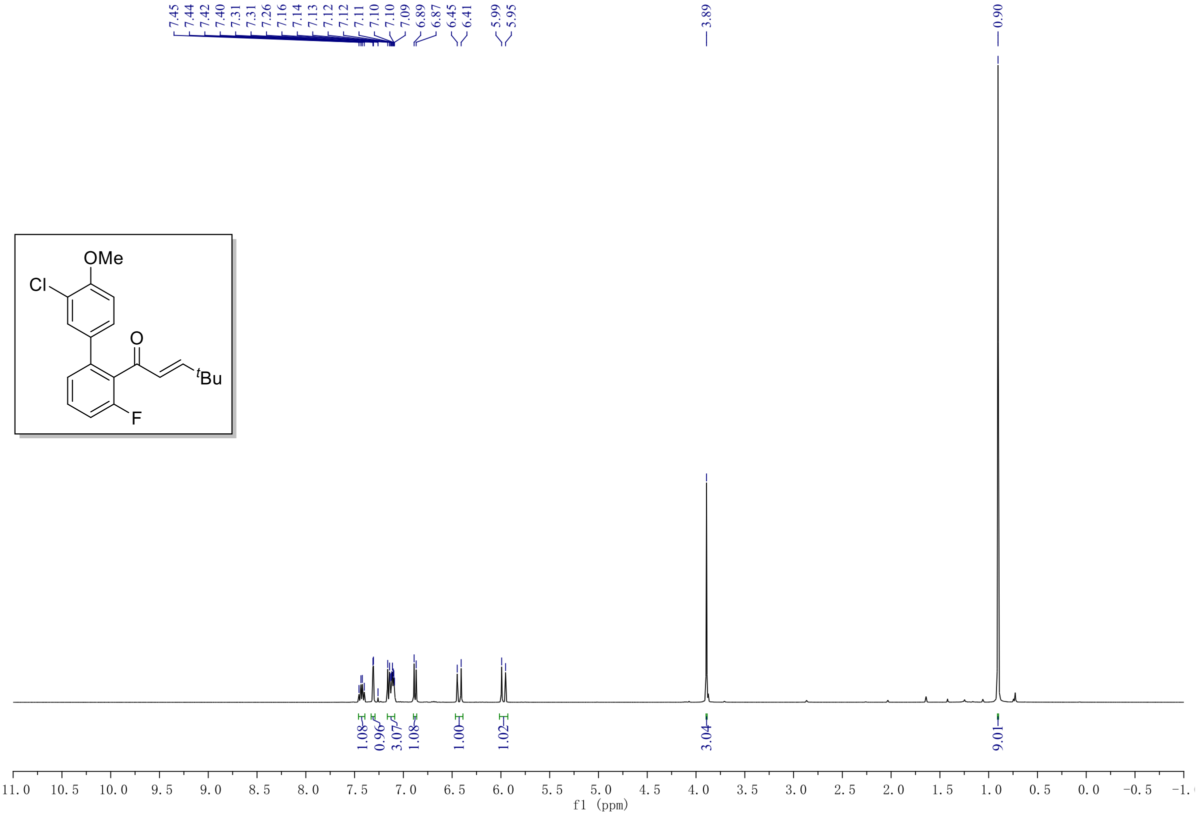

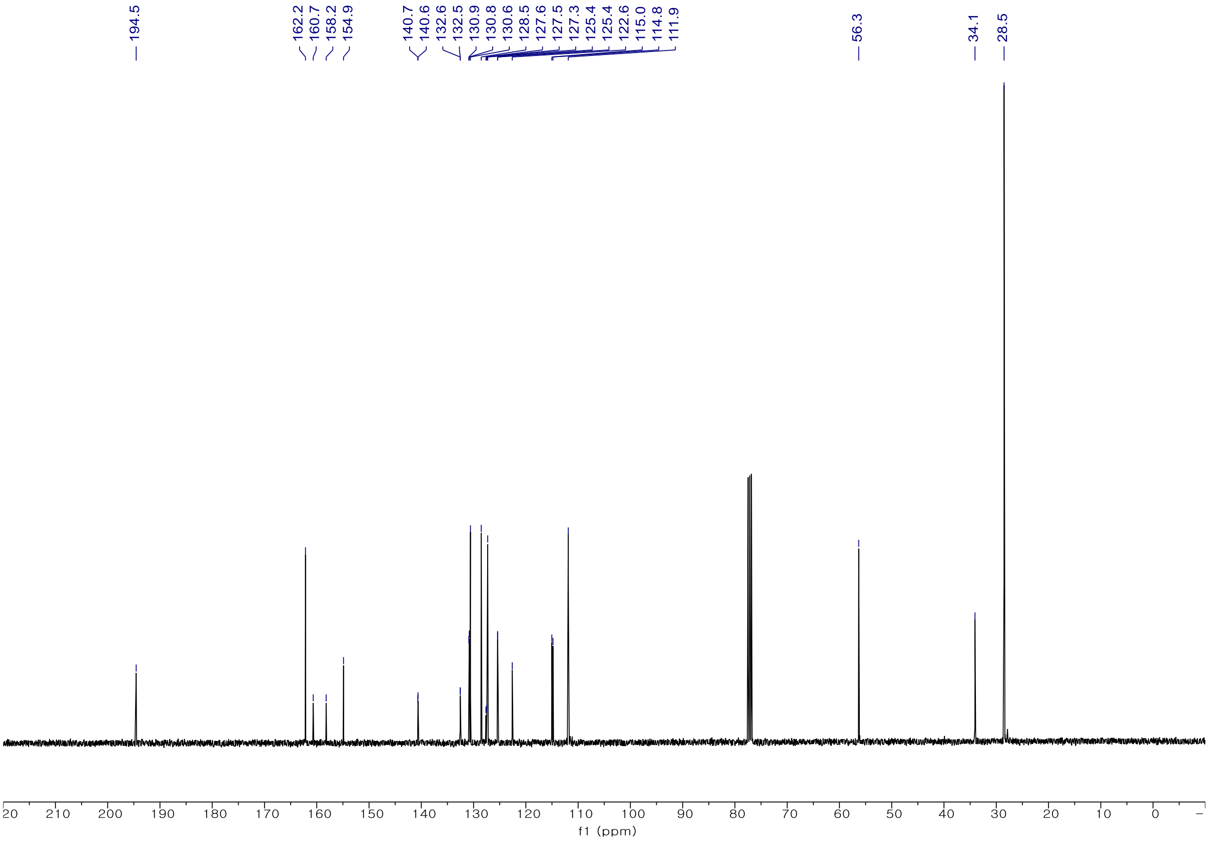

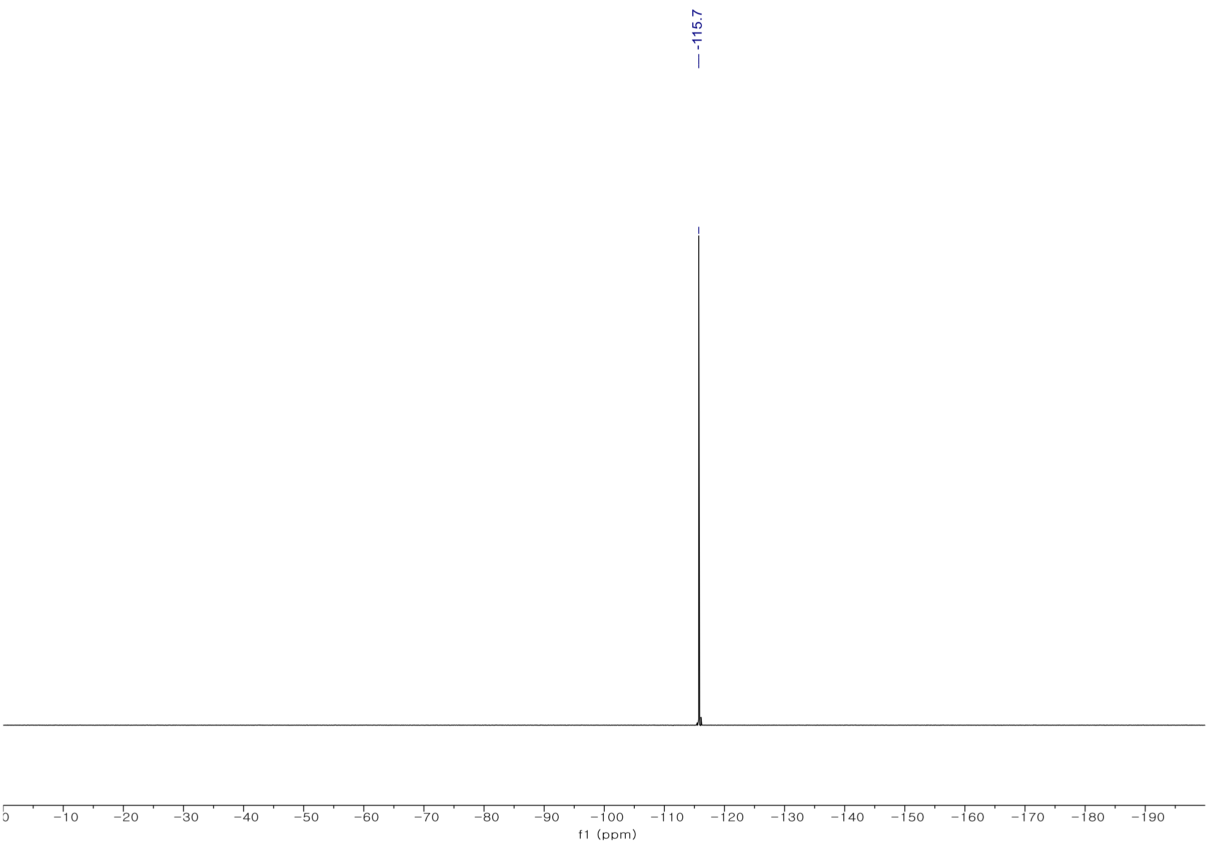
-115.7
0 −10 −20 −30 −40 −50 −60 −70 −80 −90 −100 −110 −120 −130 −140 −150 −160 −170 −180 −190
f1 (ppm)

(*E*)-3-(4-Methoxyphenyl)-3-phenyl-1-[2-(piperidin-1-yldiazenyl)phenyl]propan-1-one (S10)

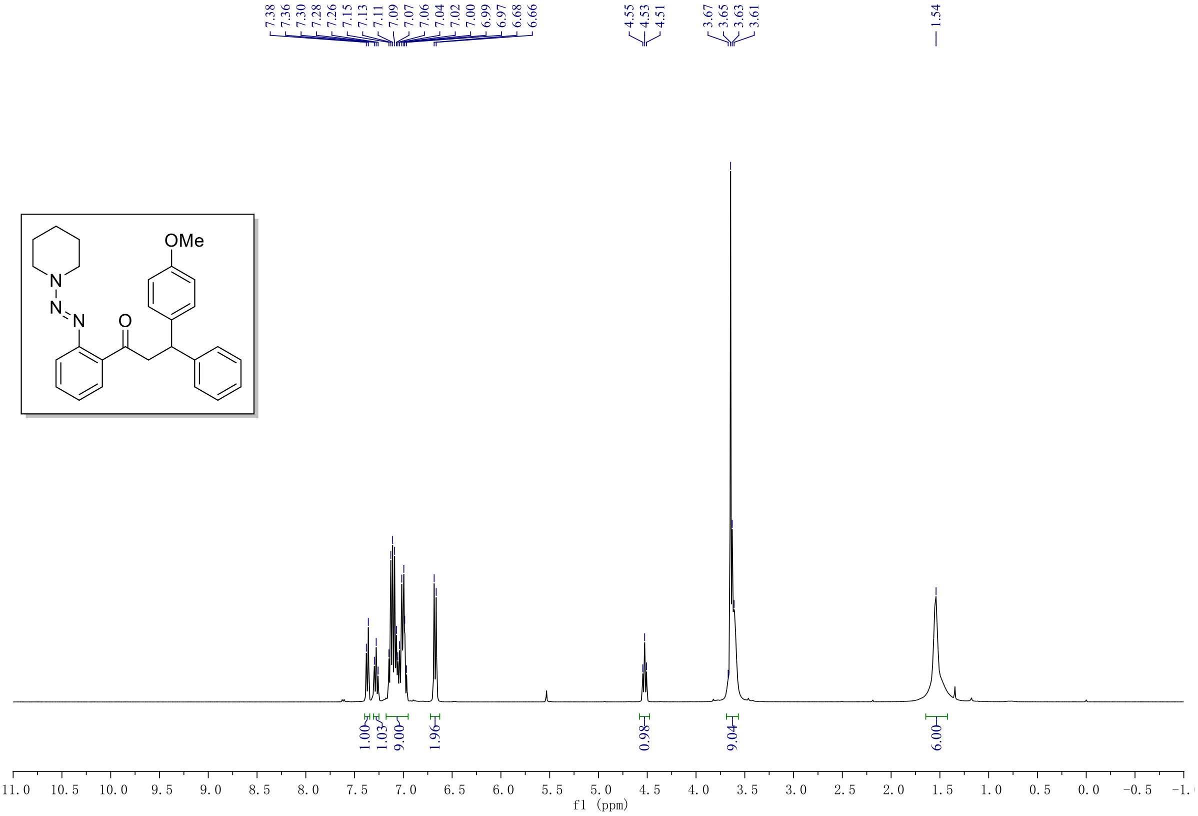

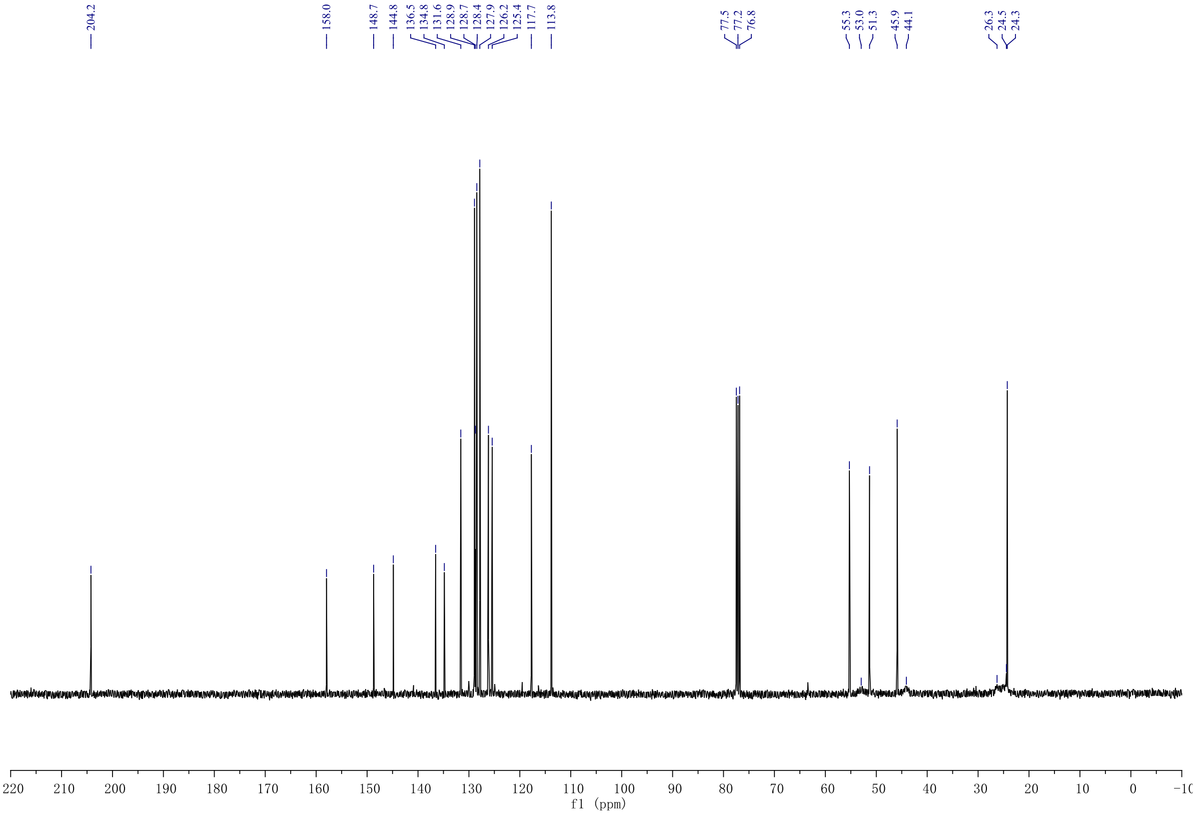

3-(4-Methoxyphenyl)-3-phenyl-1-[4'-(trifluoromethyl)-[1,1'-biphenyl]-2-yl] propan-1-one (14)

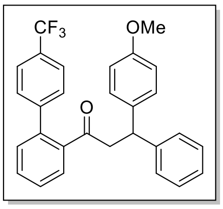

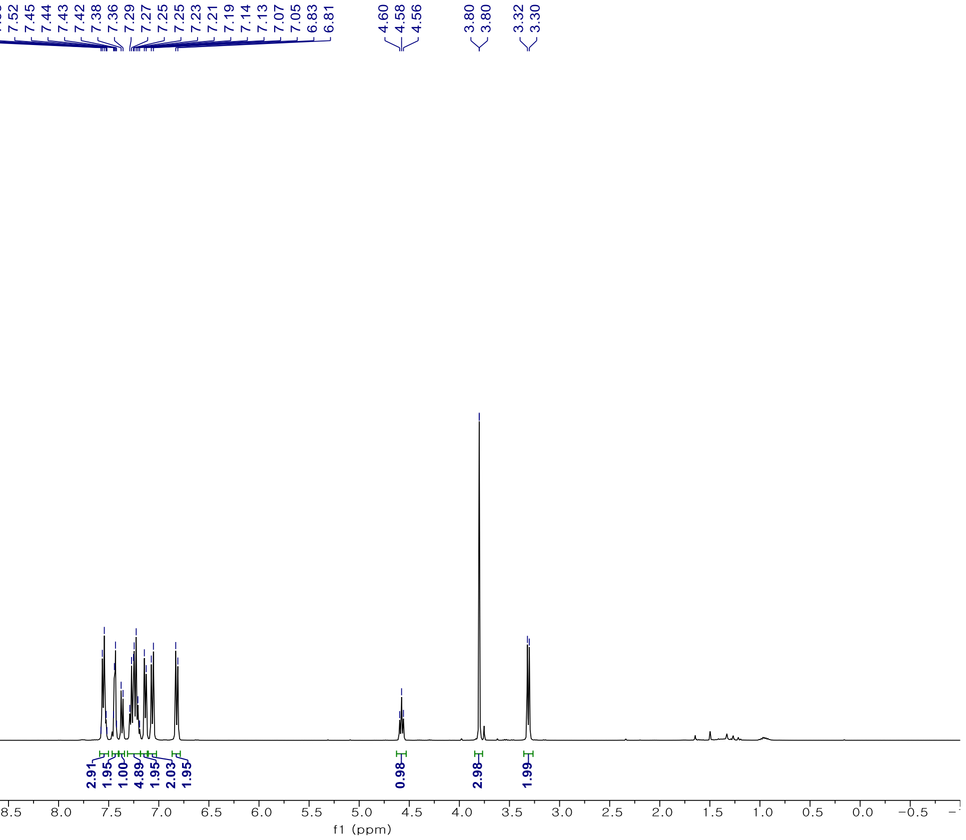

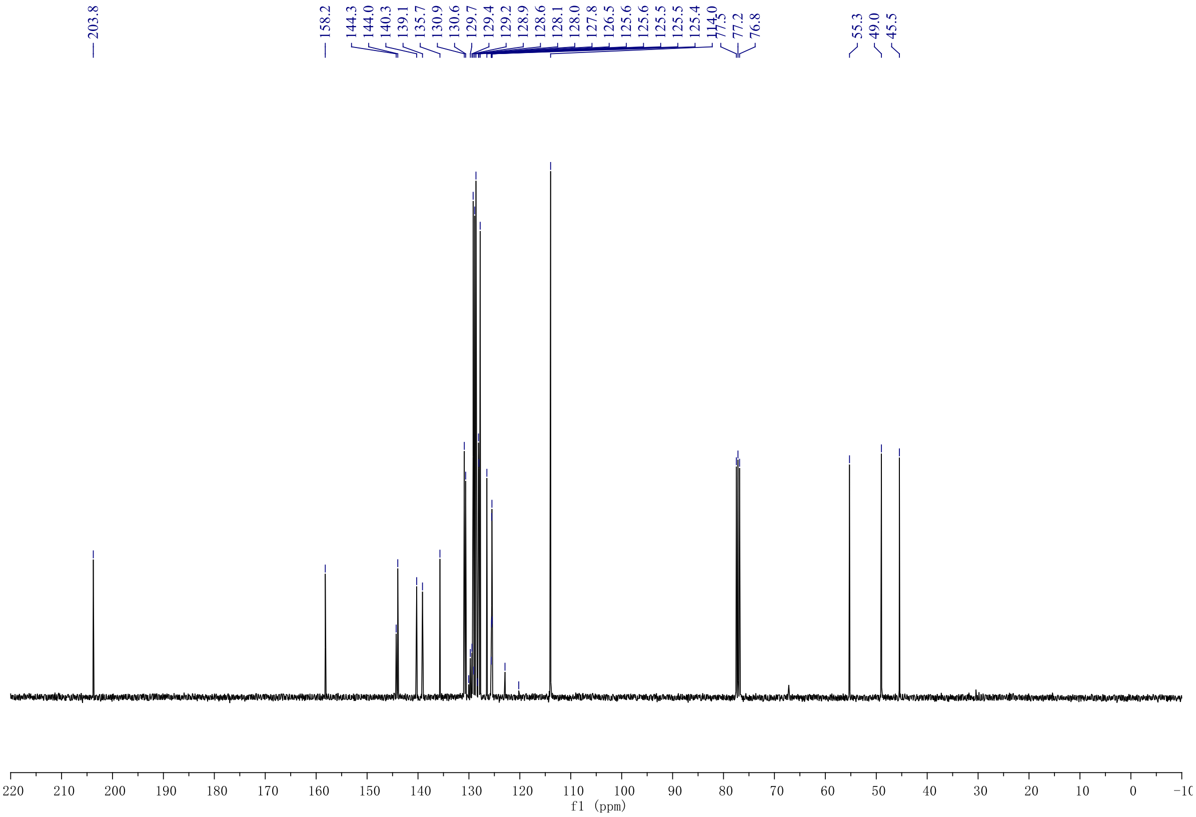

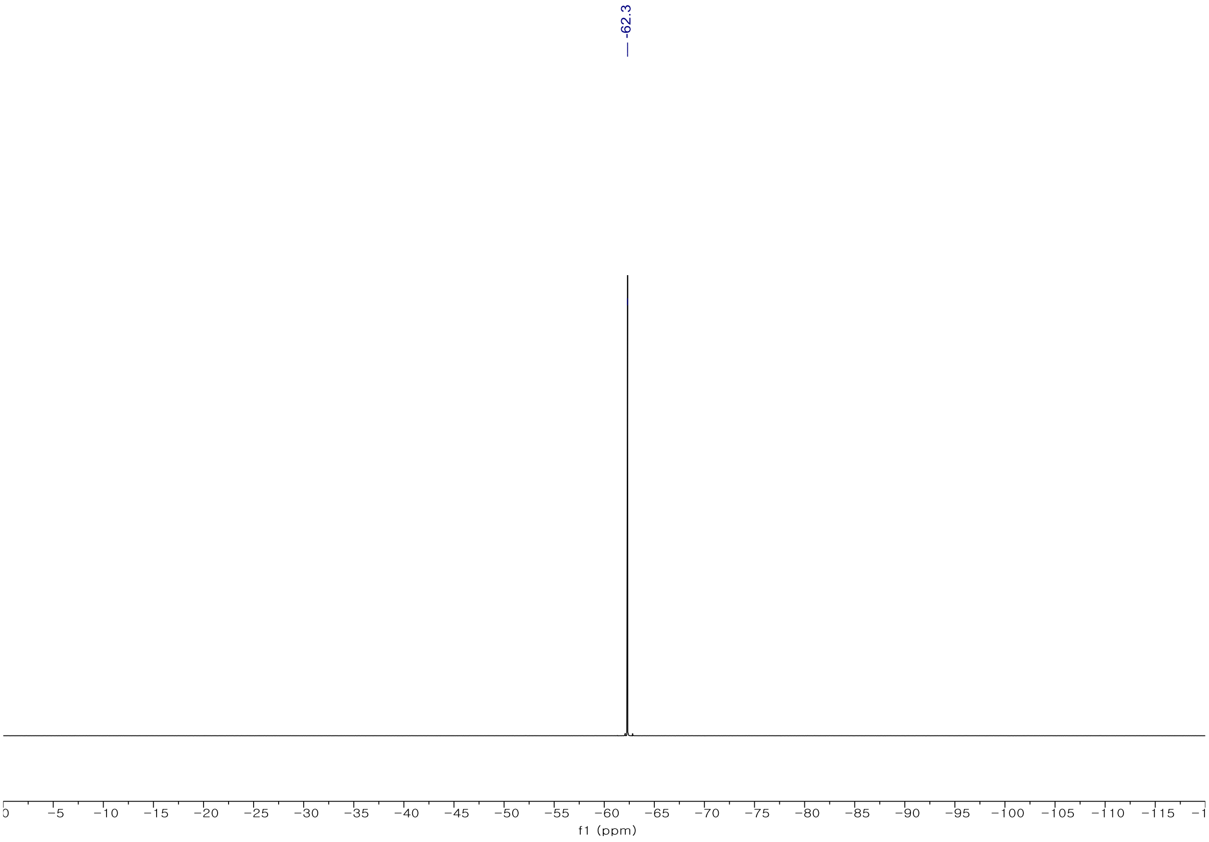
-62.3
0
-5
-10
-15
-20
-25
-30
-35
-40
-45
-50
-55
-60
-65
-70
-75
-80
-85
-90
-95
-100
-105
-110
-115
f1 (ppm)

(*E*)-Butyl 3-{3-bromo-5-[(*E*)-4,4-dimethylpent-2-enoyl]-2-fluoro-6-[(*E*)-piperidin-1-yldiazenyl]-phenyl}acrylate (15)

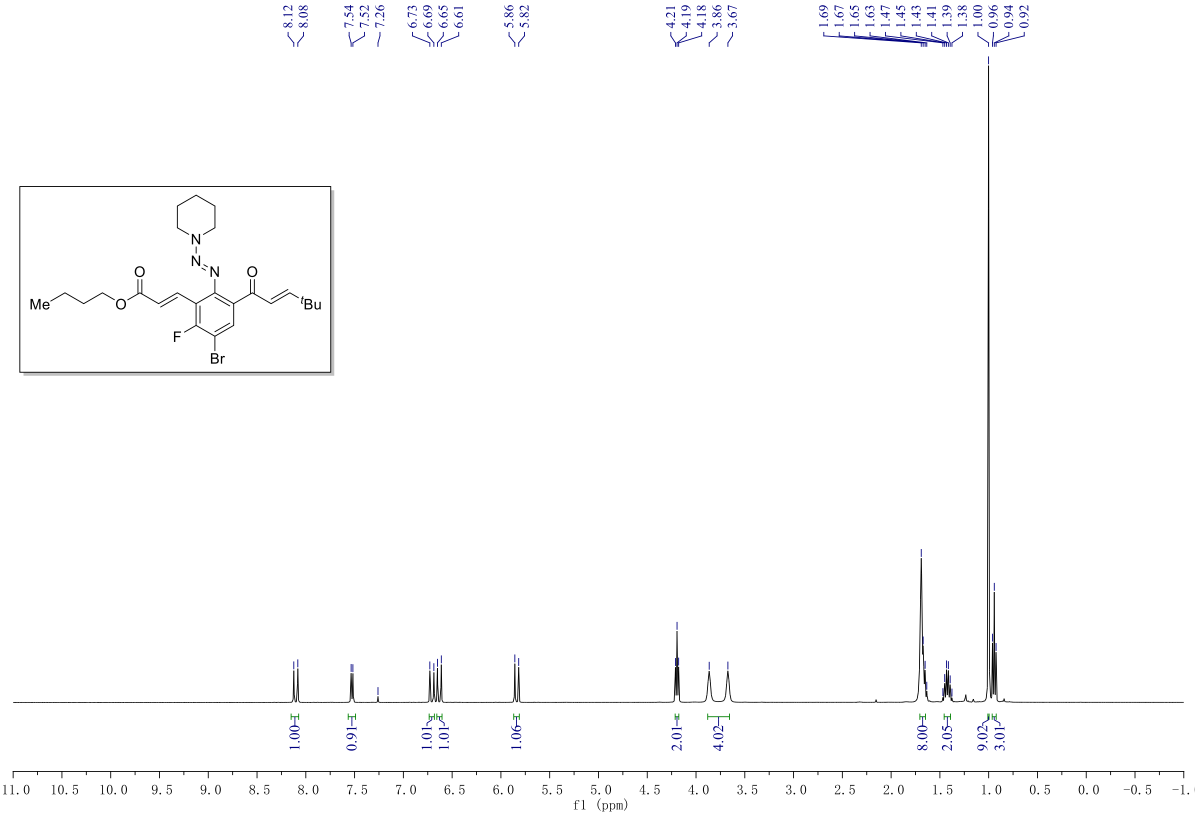

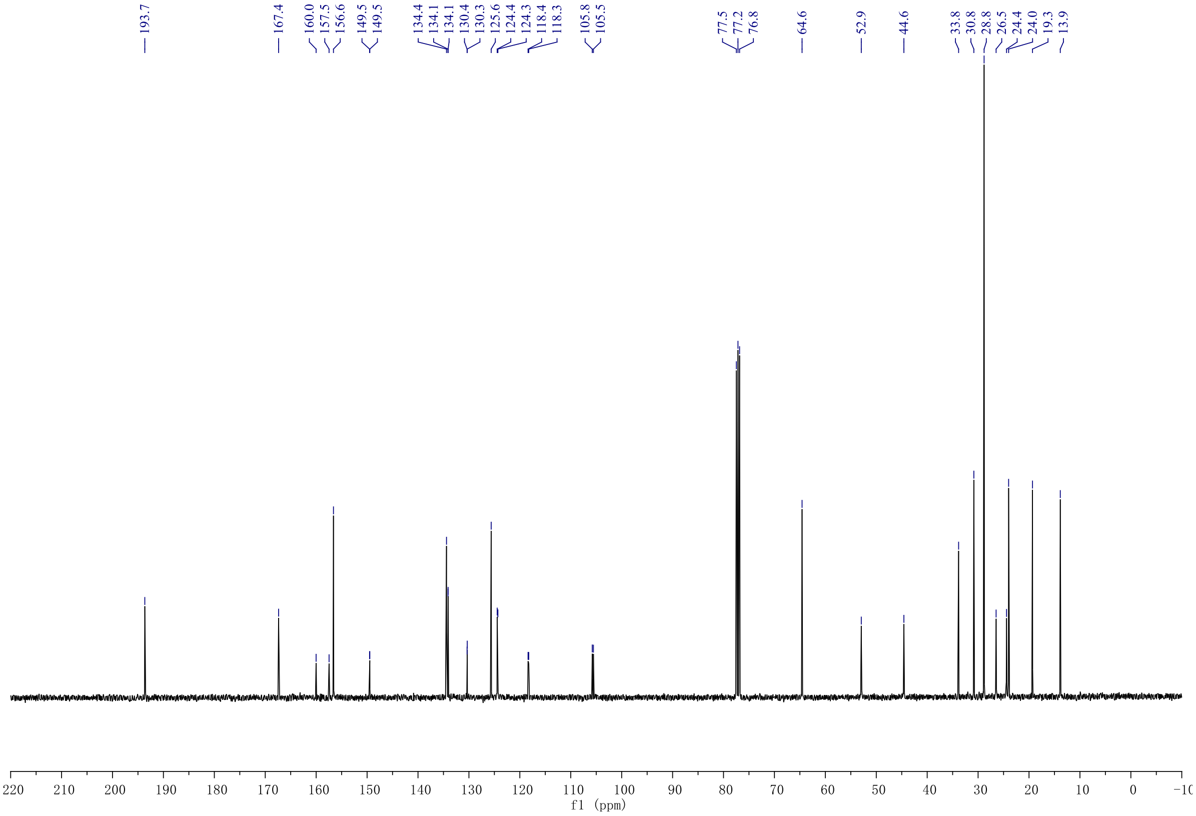

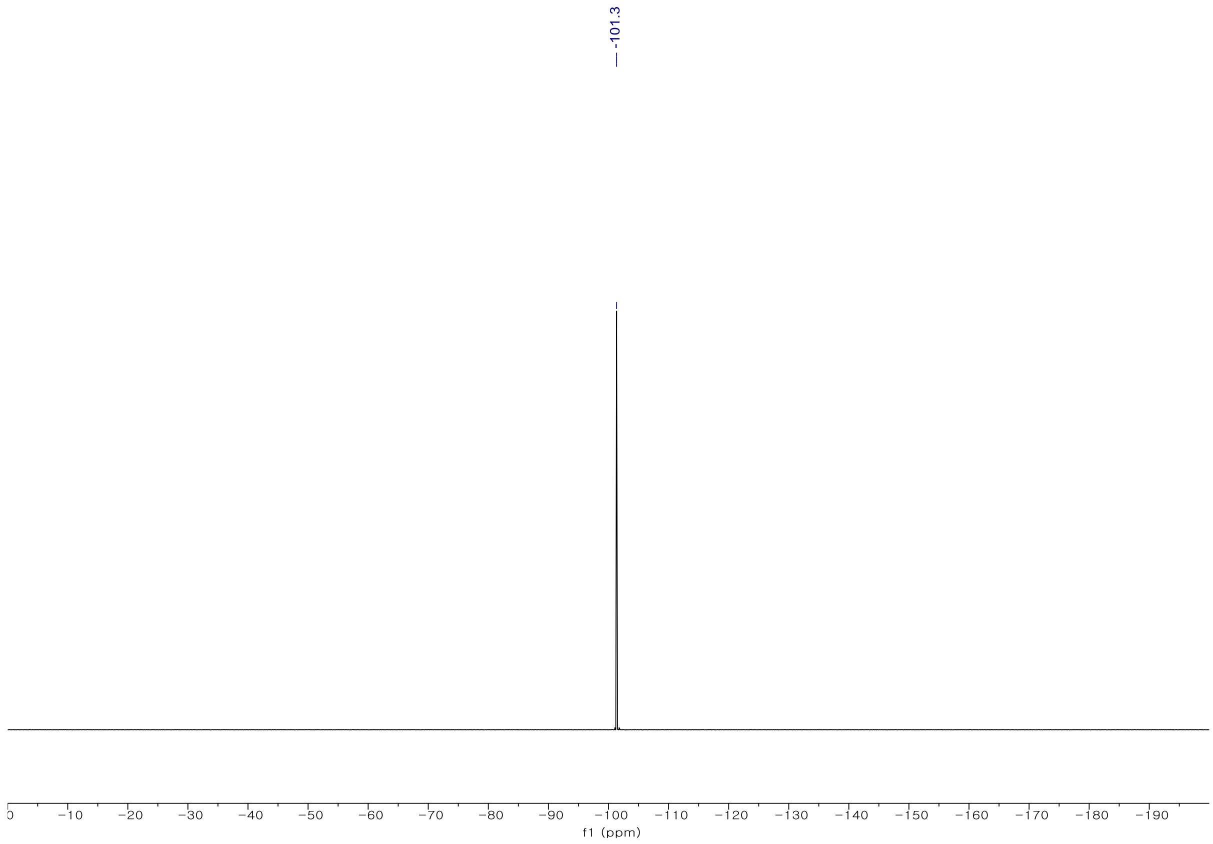
-101.3
0
-10
-20
-30
-40
-50
-60
-70
-80
-90
-100
-110
-120
-130
-140
-150
-160
-170
-180
-190
f1 (ppm)

(*E*)-Butyl 3-{4'-acetyl-5-[(*E*)-4,4-dimethylpent-2-enoyl]-2-fluoro-4-[(*E*)-piperidin-1-yldiazenyl]-[1,1'-biphenyl]-3-yl}acrylate (16)

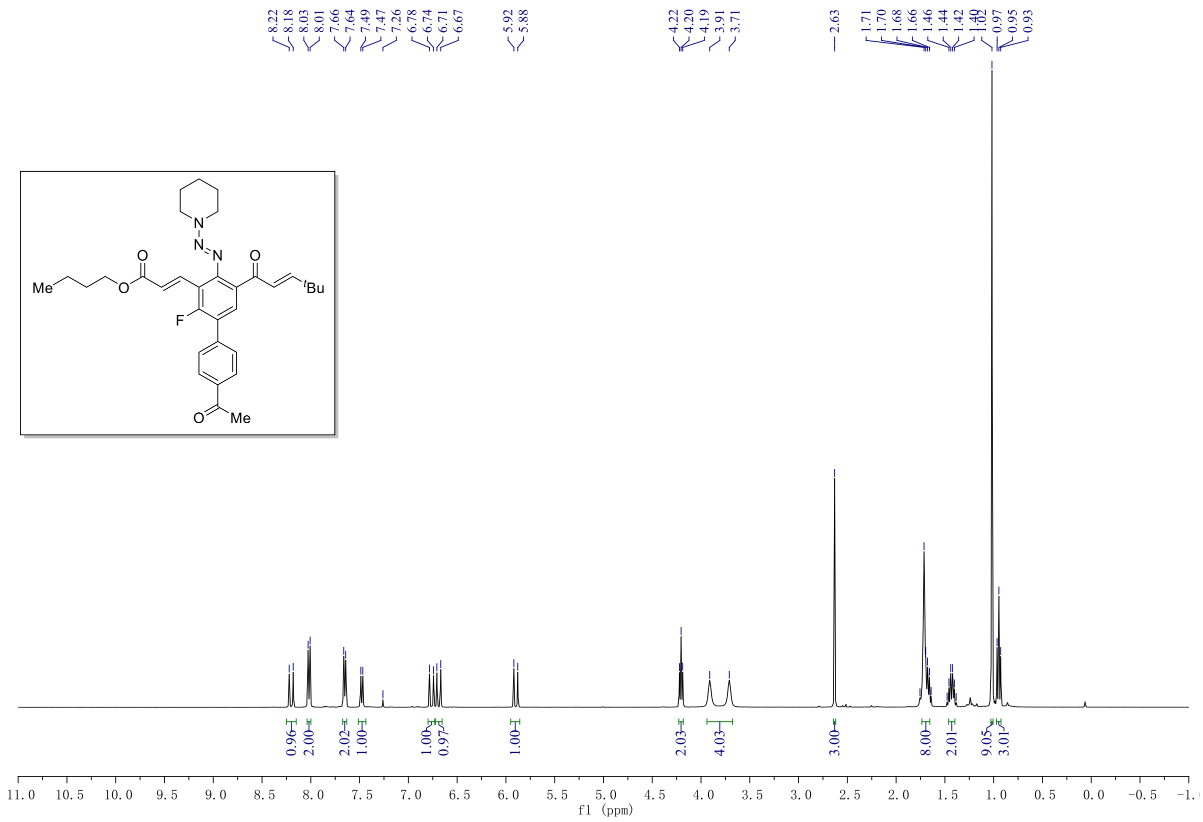

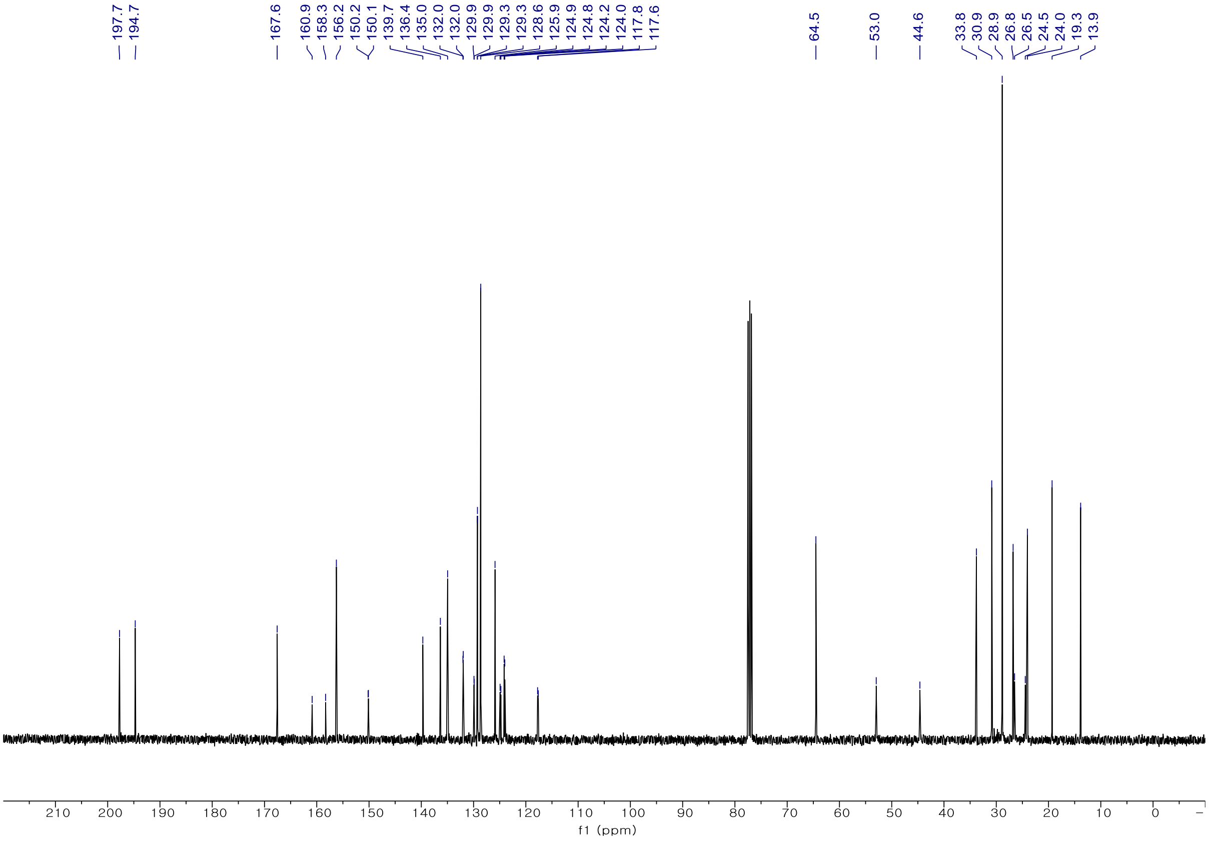

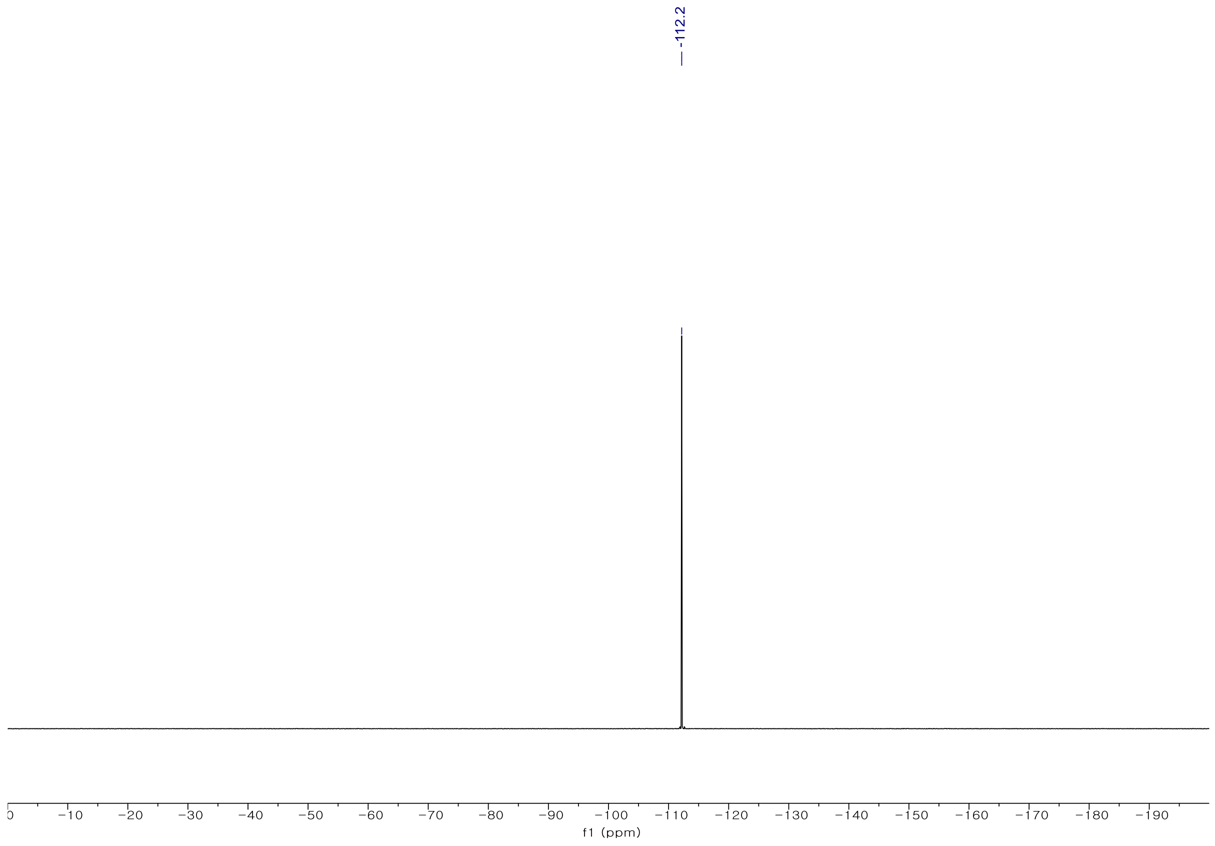
-112.2
0
-10
-20
-30
-40
-50
-60
-70
-80
-90
-100
-110
-120
-130
-140
-150
-160
-170
-180
-190
f1 (ppm)
